# A flexible open-source processing workflow for multiplexed fluorescence imaging based on cycles

**DOI:** 10.12688/f1000research.124990.1

**Published:** 2022-09-29

**Authors:** Guillaume Potier, Aurélie Doméné, Perrine Paul-Gilloteaux

**Affiliations:** 1 Nantes Université, Inserm UMR 1307, CNRS UMR 6075, Université d’Angers, CRCI2NA, Nantes, F-44000, France; 2 LabEx IGO “Immunotherapy, Graft, Oncology”, Nantes, France; 3 Nantes Université, CHU Nantes, CNRS, Inserm, BioCore, US16 UAR 3556, SFR Bonamy, Nantes, F-44000, France; 4 Nantes Université, , CNRS, INSERM, l’institut du thorax, Nantes, F-44000, France

**Keywords:** Bio image analysis, workflow, registration, signal processing, segmentation, fluorescence microscopy, multiplexing

## Abstract

**Background**

Multiplexing tissue imaging is developing as a complement for single cell analysis, bringing the spatial information of cells in tissue in addition to multiple parameters measurements. More and more commercial or home-made systems are available. These techniques allow the imaging of tens of fluorescent reporters, where the spectral overlap is solved by imaging by cycles the fluorophores using microfluidics to change the reporters between each cycle.

**Methods**

For several systems, the acquisition system coupled to the microfluidic system is a wide field microscope, and the acquisition process is done by mosaicking to cover a large field of view, relying on image processing to obtain the data set to be analysed in intensity. The processed data set allows the identification of different populations, quite similarly to cytometry analysis, but with spatial information in addition. To obtain the final image for analysis from the raw acquisitions, several preprocessing steps are needed for inter-cycle registration, tissue autofluorescence correction or mosaicking. We propose a workflow for this preprocessing, implemented as an open source software (as a library, command line tool and standalone).

**Results**

We exemplify the workflow on the commercial system PhenoCycler *^TM^* (formerly named CODEX®) and provide a reduced size data set for testing.

**Conclusions**

We compare our processor with the commercially provided processor and show that we solve some problems also reported by other users.

## Introduction

Mammalian cells are organized in tissues and organs. They are assemblies of multiple cell types that can interact together. The tissue microenvironnment has been recognised as important during organisms’ development or for processes of deregulation such as cancer ^1– 5^. Understanding the spatial architecture or the heterogeneity in the tissue environment is key to understanding for example, the biology and progression of cancer or complex immune system disorders.

Single-cell technologies, like Next Generation Sequencing-based tools and flow or mass cytometry enable the detection of numerous parameters. However, biological samples are destroyed for the study and they don’t provide the associated spatial dimension.

In clinical practice, tissue samples are usually cut and then stained with conventional immunohistochemistry or immunofluorescence technologies but this is limited to measuring a few parameters simultaneously with the use of consecutive tissue sections, due in particular to the spectral overlap of fluorophores.

A number of technologies have emerged in the last years with different strategies for multiple epitope detection on a single slide. The commercial system PhenoCycler *^TM^* (formerly named CODEX® for co-detection by indexing) is a technology that uses DNA-conjugated antibodies with fluorescent nucleotides ^6, 7^. Associated to a fluorescence microscope, a multiplexed imaging device cycles through sample washing and marker substitution, allowing an important number of markers to be acquired using only a few fluorochromes. This technology solves the problem of spectral overlap and panel composition but requires an image processing software reconstituting the entire data from the raw acquired data.

**Figure 1.**
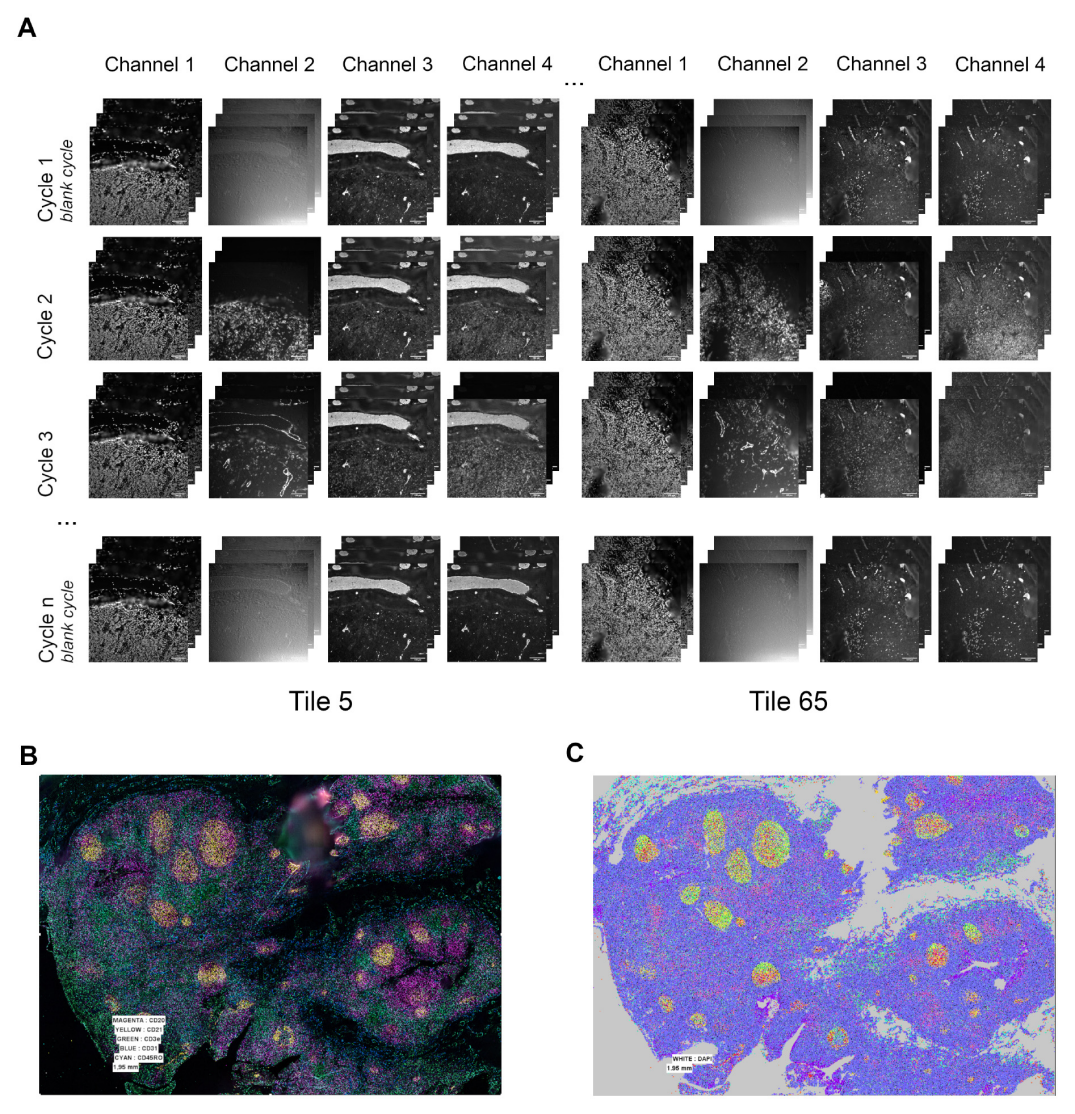
( **A**) Raw images tiles before processing step: examples of tiles 5 and 65 extracted from 117 (13x9) mosaic acquisition. For each tile, one image was obtained for each plane of Z-stack, for each fluorescent channel and for each cycle acquisition. ( **B**) Stitched image after processing: combined channels for visualisation with CD20 in magenta, CD21 in yellow, CD3e in green, CD31 in blue and CD45RO in cyan. ( **C**) Color-coded populations as identified from the output of our pipeline by x-shift clustering using the Multiviewer Analysis Viewer (MAV), the Akoya Biosciences analysis software.

Here we focus on the processing step to process raw images out from the microscope ( Figure 1 (A)) to obtain the full stitched image ( Figure 1 (B)), including segmentation based on nuclei staining. The output of our workflow can then be used in different analysis software to analyse the different populations of cells ( Figure 1 (C)). Our workflow is very similar to the original Codex Processor software provided by the vendor, or to the open source CODEX Toolkit Uploader ^6, 8^. Briefly, this software computationally concatenates and drift-compensates the images using nuclear stain as a reference, removes out-of-focus light using the Microvolution deconvolution algorithm, subtracts the background (using blank imaging cycles without fluorescent oligonucleotides), and creates hyperstacks of all fluorescence channels and imaging cycles. A Codex Segmenter was also made available by the same team to identify the cells incorporating recent advances in the segmentation methods ^9^. However, as we encountered several issues with the original software provided along with the device, we developed our own processing pipeline, to solve in particular issues regarding the stitching, the intensity normalisation and the segmentation.

The cell phenotyping and identification of population usually relies on the crowded nuclei segmentation and intensity measurements ^8, 10– 12^ and then on the quality of the processing step. In particular one needs to take into account the non-perfect flatness of the tissue, the mechanical drift between the subsequent acquisitions of the same position for different cycles, the non-uniformity of the microscope fields, the correction of the autofluorescence signal to make intensity measurement measurable, the stitching for the full mosaic to identify uniquely nuclei, and the segmentation of crowded nuclei in tissue.

## Methods

### Implementation

**Figure 2.**
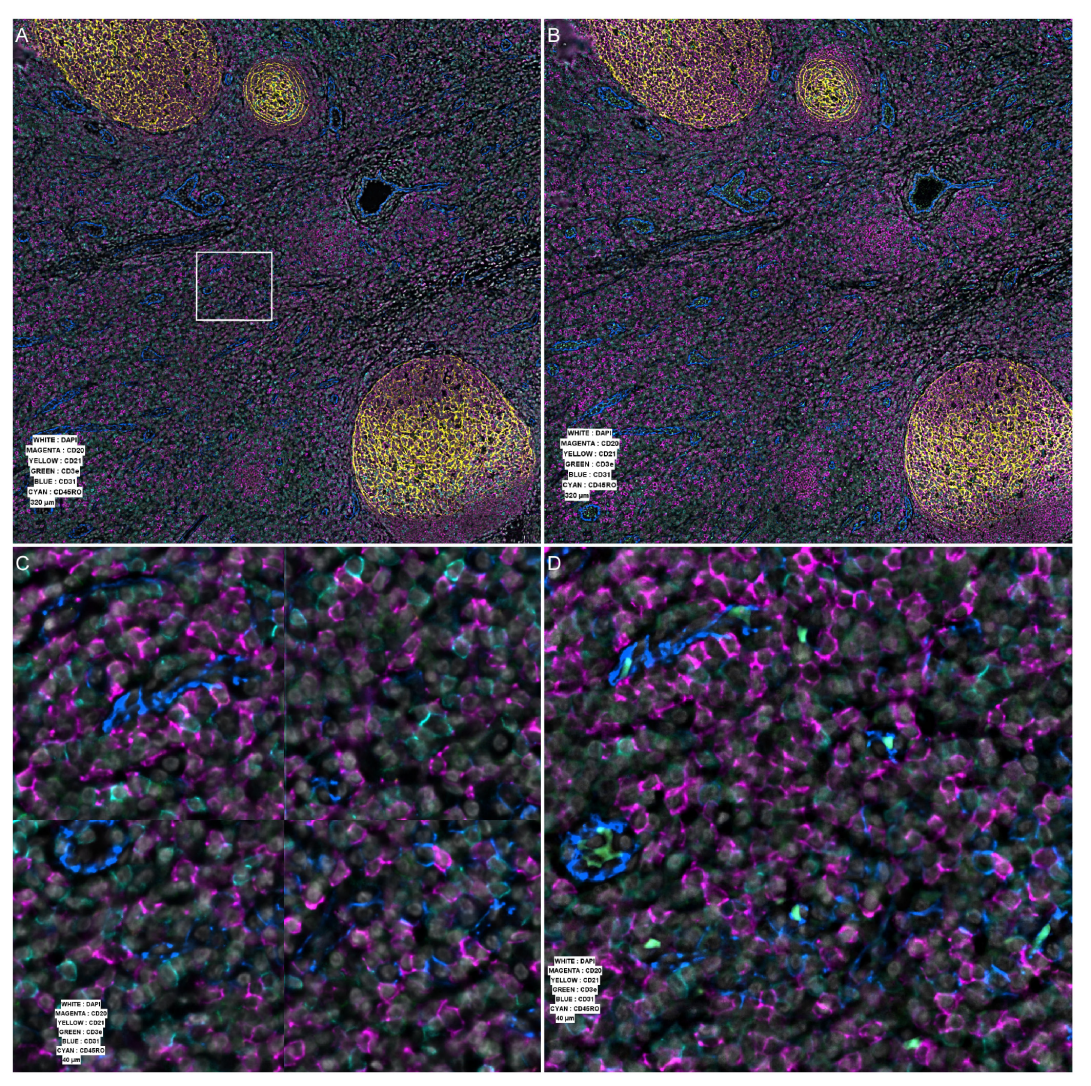
Comparison with Codex Processor from Akoya Biosciences v1.8.2. ( **A**) Processed data as generated by the original vendor software v1.8.2. White square shows the position of the insets shown in panels ( **C**) and ( **D**), at the intersection of four tiles. ( **B**) The same area processed by our software. ( **C**) Zoomed area on the intersection showing defect in stitching. ( **D**) The same area after our processing.

**Figure 3.**
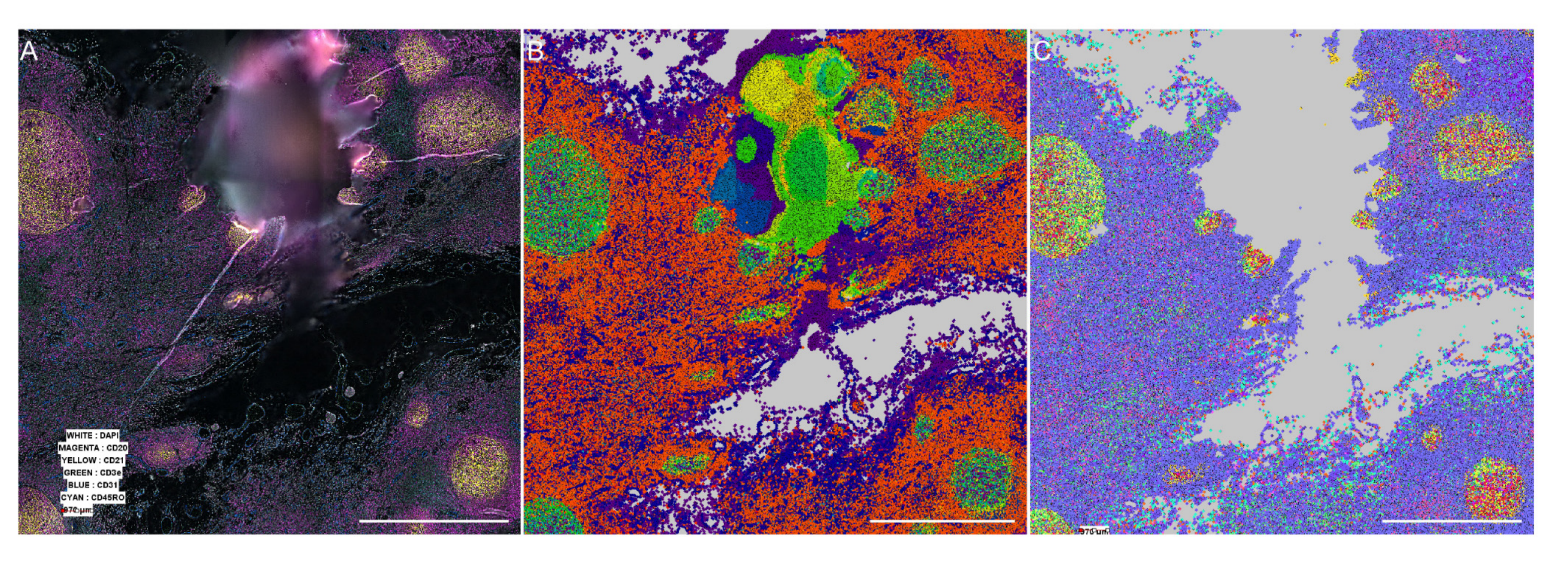
Comparison of populations generated by the vendor processor and our processor on an area with visible effect. Scale bar is 5 millimeters. ( **A**) Stitched intensity image generated by our processor. ( **B**) Color-coded x-shift generated populations generated by the MAV based on the segmentation and intensity measured by the vendor processed pipeline. ( **C**) Color-coded x-shift generated populations generated by the MAV based on the segmentation and intensity measured by our pipeline. Grey means no cells detected.

We have developed a new implementation of the processor, in particular adapted to the PhenoCycler *^TM^*, but that could be used for any other system relying on cycles of acquisition. In particular our goal was to solve some problems encountered by the original software provided with the PhenoCycler as demonstrated in Figure 2 (C) and Figure 3 (B).

Our pipeline is written using the JAVA programming language. Our code is organised around a core library providing main functionalities and consumed by a command line interface (CLI) as well as a GUI (Graphical User Interface). The CLI application exposes each processing step independently whereas the GUI provides an easy interface to start the entire pipeline, with the option to select a subpart of the workflow to be run. The different dependencies and required packages are listed in https://gitlab.in2p3.fr/micropicell/multiplexprocessor/-/blob/master/README.md. Maven is used for software dependencies. A binary version compiled for Windows is provided with instructions for reader convenience, at https://gitlab.in2p3.fr/micropicell/multiplexprocessor/-/wikis/Install-from-binary ( Figure 2).

### Operation

**Figure 4.**
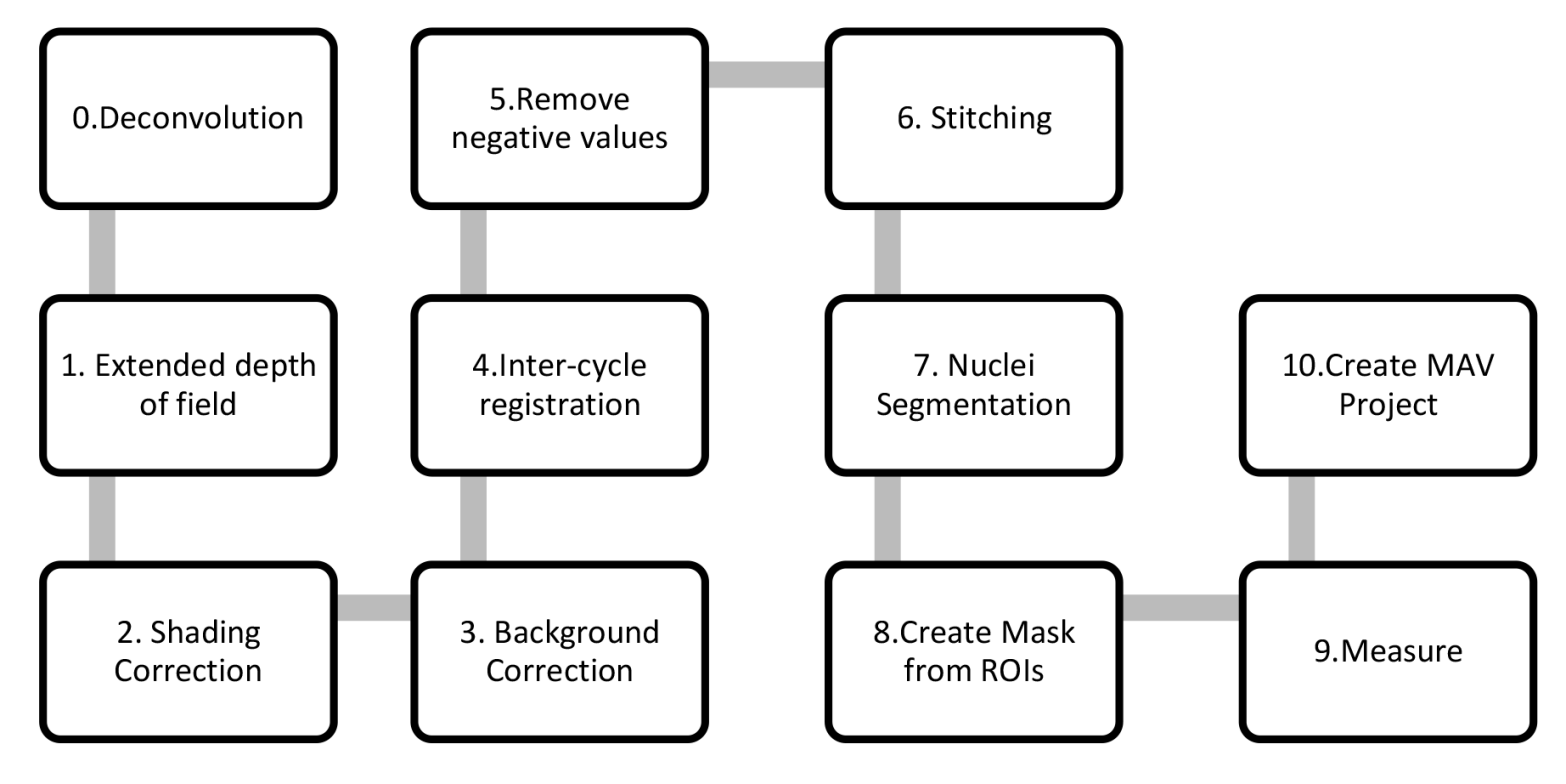
Complete workflow of the pipeline.

The workflow was run on a laptop with Intel® Core *^TM^* i7-7Y75, CPU @ 1.30 GHz, 16 Gb of RAM, with Windows 10 64-bit. Image processing performed includes deconvolution, extended depth of field, shading correction, background subtraction, cycle registration, stitching and segmentation ( Figure 4).

Deconvolution is included using DeconvolutionLab2 ^13^ and PSFGenerator ^14^ from EPFL BIG. However this step can be performed using any deconvolution software, and an example implementation with the commercial licensed software Microvolution® ^15^ is provided as a branch in the code repository. Extended depth of field is provided using EPFL BIG implementation ^16^. Shading correction is performed by dividing a profile image computed using median intensities. Background correction is performed by subtracting signal from the blank cycles. Cycle registration is performed using TurboReg ^17^ from EPFL BIG. Position-wise tiles from reference channel are registered against tiles from reference cycle. Computed translations are then applied to all the remaining channels. Finally, registered tiles are cropped by the greatest computed translation. Stitching is performed using MIST ^18^ from NIST. Stitched positions are computed using reference channel of the reference cycle and then applied to all remaining channels and cycles. Segmentation of nuclei is performed using Stardist ^19, 20^. The output directory structure is compatible with the original analysis software provided with the device CODEX® multiplex analysis viewer (MAV). Furthermore FCS files are generated using Flow Cytometry Data Standards ^21^ and can be analyzed using any other tool.

## Use case

We provide an example dataset acquired in our lab using Formalin-Fixed Paraffin-Embedded (FFPE) human tonsil and describe the step-by-step operation using the GUI to call the core functions and the provided dataset, described in Data Availability.

### Tissue material

FFPE tonsil human sample was obtained from the tissue bank of the Nantes university hospital. FFPE tonsil human was sectioned at a thickness of 5 *µ*m and directly adhered onto poly-L-lysine (Sigma Aldrich) coated coverslips (Akoya Biosciences). Tissue coverslips were stored at 4°C until staining.

### Sample staining and imaging

Staining and imaging with PhenoCycler *^TM^* were performed using the Akoya Biosciences protocol available in https://www.akoyabio.com/wp-content/uploads/2021/01/CODEX-User-Manual.pdf

The sample cover slip was placed on a 55°C hot plate for 20 minutes. Tissue section was cooled down before deparaffinization and hydration steps. Tissue was immersed in the following solvent series for 5 minutes by step: twice in Xylene, twice in 100% Ethanol, once in 90%, 70%, 50%, 30% Ethanol and twice in ddH2O. Antigen retrieval was performed in a hot water bath at 94°C in a 1X Citrate Buffer (Sigma Aldrich) for 20 minutes.

After cooling at room temperature, tissue was briefly washed twice in ddH2O for 2 minutes, twice in Hydration buffer (Akoya Biosciences), then was incubated in Staining buffer (Akoya Biosciences) at room temperature for 20 minutes. The antibody cocktail (10 antibodies and DAPI nuclear stain - Table 1) was prepared in a blocking solution according to the Akoya Biosciences instructions and tissue section was incubated with 190 µL of this solution at room temperature for 3 hours in humidity chamber. Stained tissue was washed twice in Staining buffer (Akoya Biosciences) for 2 minutes, then was post-fixed with 1.6% PFA in Storage Buffer (Akoya Biosciences) for 10 minutes. After an incubation in a cold methanol solution for 5 minutes, tissue section was successively washed three times in PBS 1X and fixed with a PhenoCycler *^TM^* fixative solution at room temperature in humidity chamber for 20 minutes. Finally, tissue cover slip was washed three times in PBS 1X and stored in Storage Buffer (Akoya Biosciences) at 4°C before PhenoCycler *^TM^* multi-cycle imaging.

**Table 1.** Marker panel for PhenoCycler *^TM^* (antibodies from Akoya Biosciences).

Target	Clone	Reporter dye	Dilution	Cycle
CD21	EP3093	ATTO550	1 */*200	2
CD3e	EP449E	Cy5	1 */*200	2
CD20	L26	AlexaFluor *^TM^* 750	1 */*200	2
CD8	C8/144B	ATTO550	1 */*200	3
CD4	EPR6855	Cy5	1 */*200	3
CD31	EP3095	AlexaFluor *^TM^* 750	1/750	3
CD45RO	UCHL1	ATTO550	1/200	4
CD11c	118/A5	Cy5	1/200	4
Pan-Cytokeratin	AE-1/AE-3	AlexaFluor *^TM^* 750	1 */*750	4
Ki67	B56	ATTO550	1 */*200	5

Sample and reagents were equilibrated at room temperature before the PhenoCycler *^TM^* run. According with Akoya Biosciences recommendations, a 96-well plate of PhenoCycler *^TM^* reporters complementary to the bar-codes used in the antibody panel with a nuclear stain was prepared.

Solutions (PhenoCycler *^TM^* 1X buffer, dimethyl sulfoxide, water) required for the PhenoCycler *^TM^* fluidic instrument automate were loaded into the PhenoCycler *^TM^* and the tissue coverslip was installed onto the stage insert. Multi-imaging was performed using a Zeiss Axio Observer inverted microscope with Colibri 7 light source, coupled with the ORCA Flash 4.0 LT camera (Hamamatsu) and associated at the Filter Set 112 HE LED from Zeiss. The multiplex imaging run was executed with the PhenoCycler *^TM^* Instrument Manager (CIM) software. Nuclear DAPI staining was used to design tiled regions of the human tonsil section of interest at the 5x magnification (N-Achroplan 5x/0.15 M27, Zeiss). The focus of tissue was performed using a Plan-Apochromat 20x/0.8 M27 objective (Zeiss), with every image being 2048x2048 pixels, and a x-y pixel size of 0.325 micrometers. Led intensity and light exposure times were for each channel respectively: 50% and 500 milliseconds for AlexaFluor *^TM^* 750 and Cy5 dyes, 50% and 400 milliseconds for ATTO550 dye, and 30% and 5 milliseconds for the DAPI dye. For each antibody, images were acquired per tile for each of 4 channels with an 11 plane Z-stack and an acquisition step in Z of 1.5 micrometers.

**Figure 5.**
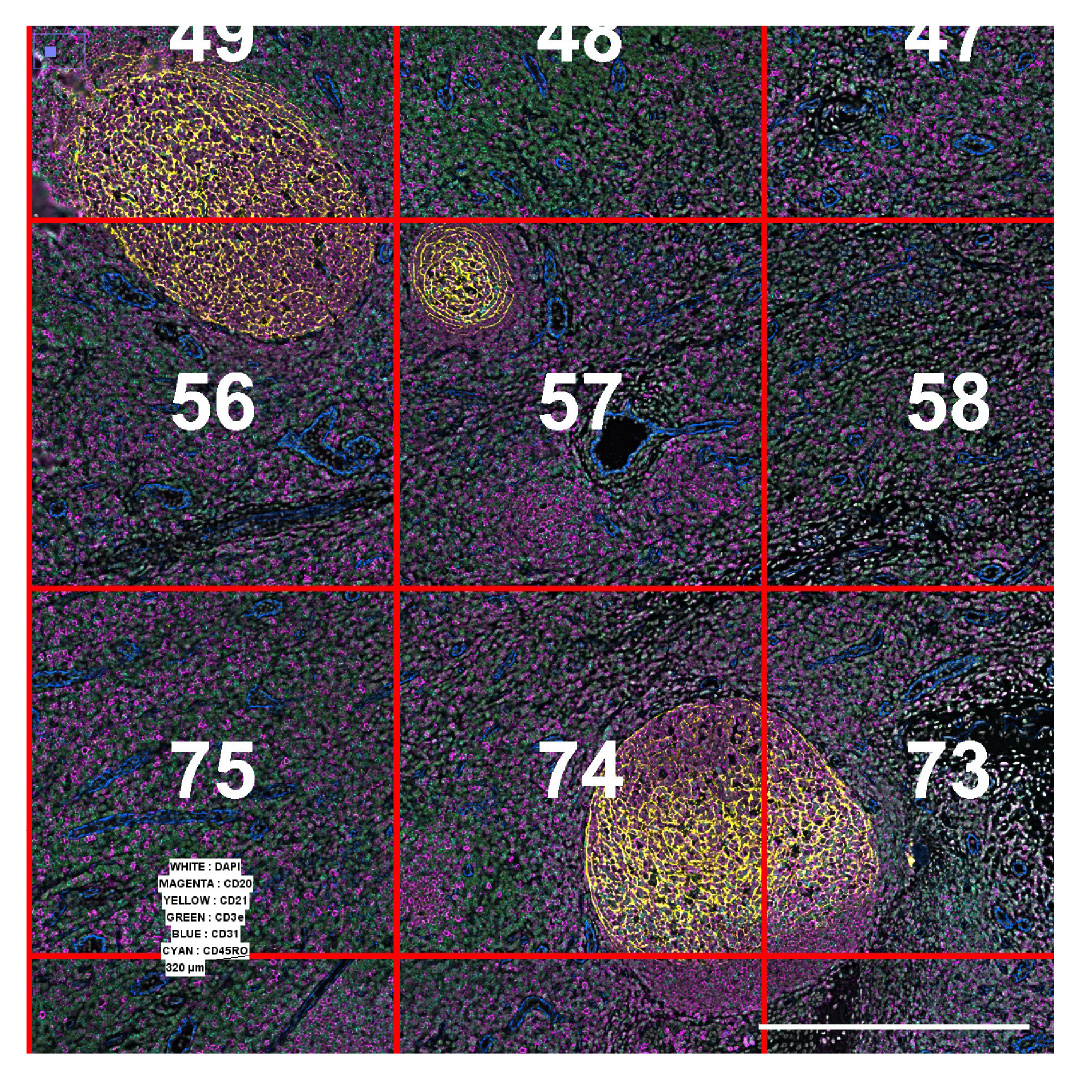
Tiles 56, 57, 75 and 74 from the original full acquisition were combined to create a small example data set. Scale bar is 2 millimeters.

A total of 117 (13x9) tiles were acquired in snake order, but the example data set is a sub-sampling of 4 tiles ( Figure 5) created by renaming image files and creating new experiment files.

### Processing

**Figure 6.**
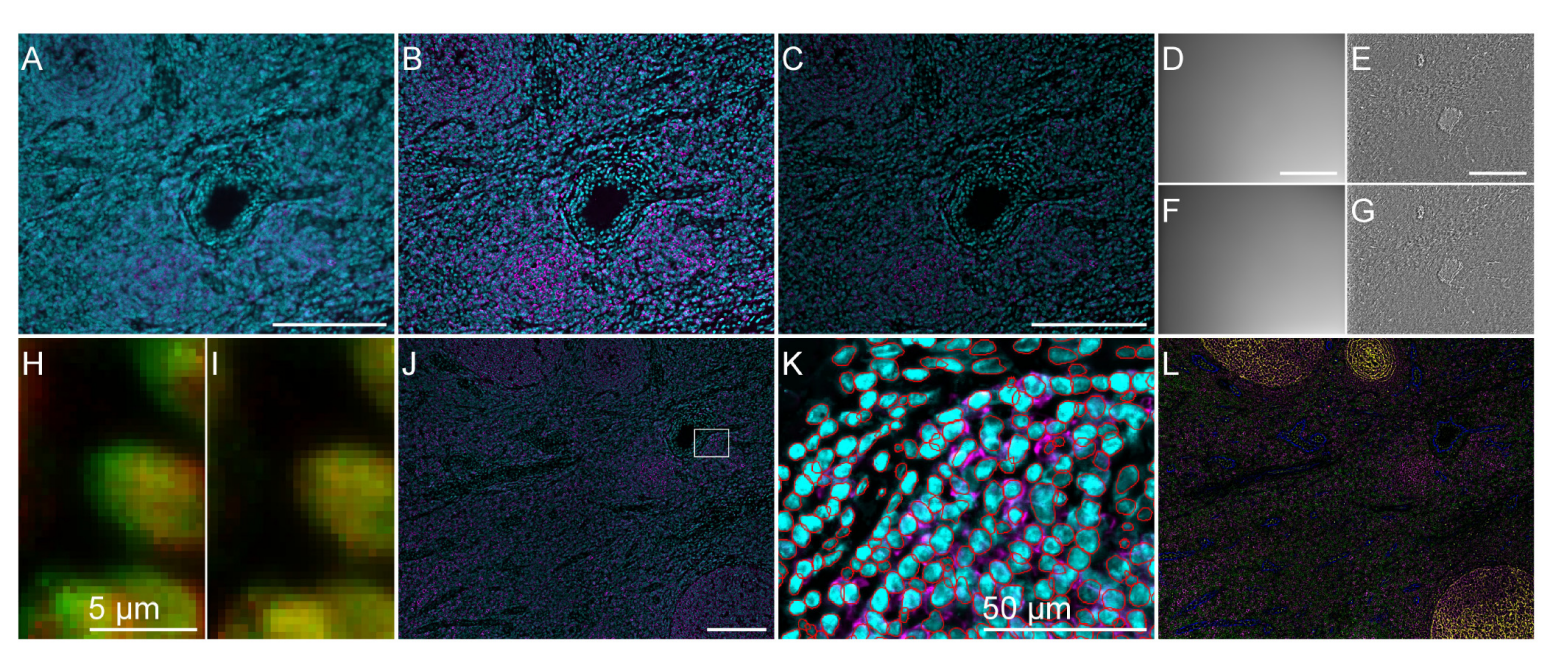
Some steps output from our workflow. All scale bars are 200 micrometers if not specified on the figure. ( **A**) One example of tile slice before deconvolution (slice 8 position 2, CD20 in magenta and DAPI in cyan). ( **B**) The same position and channels after deconvolution by Microvolution and extended field of view (from these steps all data are 2D). ( **C**) The same view after shading and background correction. ( **D**, **F**) The shading profile from cycle 1 ( **D**) and the last cycle 6 ( **F**) for the same position. ( **E**, **G**) Contrast-adjusted blank cycles 1 ( **E**) and 6 ( **G**) used to correct the autofluorescence in each wavelength of the tissue, here for channel 2, after shading correction. ( **H**, **I**) Cycle registration uses DAPI staining to correct the drift that can occur between cycles: ( **H**) shows the discrepancy between cycle 1 in red and 2 in green, ( **I**) is the same field of view after drift correction. ( **J**) Stitching result of the 4 tiles examples used with only CD20 in magenta and DAPI in cyan. The white rectangle shows the area corresponding to panel ( **K**). ( **K**) Regions of interest obtained by Stardist superimposed on DAPI (cyan) and CD20 (magenta). ( **L**) Color-merged image from stitching with CD20 in magenta, CD21 in yellow, CD3e in green, CD31 in blue and CD45RO in cyan.

Here we describe the use of the Multiplex Processor graphical user interface, exemplified on the data set provided ( Figure 6). The GUI has no parameters exposed, since metadata related to the acquisition are read from the file generated by the PhenoCycler CIM and other parameters have been optimized on different types of tissue. They can be modified if needed in a special java class for every step for parameters, but in our case are kept the same for all experimentation by the PhenoCycler *^TM^* users. Note that the workflow can be started and ended at any step, assuming that data are organised as expected in the input description in the wiki https://gitlab.in2p3.fr/micropicell/multiplexprocessor/-/wikis/MultiplexProcessor-Usage-documentation. Error and log files are generated and can be used to monitor the processing.

**Step 0: Deconvolution**

The data having been acquired with a wide-field microscope as 3D stack (an example of one slice is shown in Figure 6 (A)), the deconvolution step is a classical step to ameliorate the quality and resolution of data. Two options are offered in the Multiplex Processor as two different branches of the software. The compiled version relies on DeconvolutionLab2 ^13^. The point spread function for each wavelength is generated based on the parameters contained in the experiment json file (wavelengths, voxel size, aperture). By default when using DeconvolutionLab2 in our workflow, Richardson Lucy algorithm is used with 10 iterations. The option can be changed in our DeconvolutionProcessor class.

This step is very long using Deconvolutionlab2 (96 stacks of 11 slices each, 2048x2048, take 8 hours to be processed, at 5.10 minutes per 3D stack on a laptop). A processed deconvolved data set using Microvolution commercial software is provided as a companion data set. To use it, unzip bu_deconvolution.zip and rename the extracted directory to "out".

**Step 1: Extended depth of field (EDF)**

The purpose of this step is to create a 2D image from the 3D stack to get the in-focus information (the resulting processed slice from one stack using Microvolution followed by EDF is shown in Figure 6 (B)). We rely on the BIG EPFL plugin ^16^ for this step, with quality HIGH and topology NO_MEDIUM in the GUI implementation used here, where the full list of associated parameters are defined in the ParametersFactory class from our processor. This step should take about 1.22 minutes per stack on a laptop.

**Step 2: Shading correction**

A shading profile for the first and the last cycle of each channel is computed ( Figure 6 (D-E)) by dividing the median of all positions by its convolution with a Gaussian blur (sigma 64 pixels) kernel. For each cycle and each channel, a specific shading profile is computed by linear interpolation of the shading profile of the first cycle and the last cycle. Each tile is then corrected by dividing the EDF image by this normalised interpolated profile. This step should take about 1 second per stack.

**Step 3: Background correction**

The first and the last cycle of acquisition are acquired without marker, to create blank cycles images ( Figure 6 (F-G)). After a Gaussian blur of sigma 10 pixels, we assume these images represent the autofluorescence intensity. This autofluorescence is then linearly interpolated between the first and last cycle and subtracted for each cycle and channel to be processed. This step should take about 0.74 seconds per stack.

**Step 4: Cycle registration**

Because the acquisition is done by cycles, coming back to the same position but moving the microscope stage, a mechanical drift due to the accuracy of stage repositioning is likely to occur, as exemplified in Figure 6 (H). For these reasons, all cycles have a nuclei marker acquisition, that will then serve as a reference channel for inter-cycle registration. We used the automatic mode of Turboreg ^17^, constraining the transformation to be a translation. This step took 168 seconds in total on a non-CUDA-enabled desktop.

**Step 5: Remove negative values**

After the registration step, we have added a "Remove negative values" step. All negative intensity values in the images are replaced by 0 and the minimal intensity value of every image is subtracted to the non-zeros values.

**Step 6: Stitching**

Stitching is performed using the MIST software ^18^ based on the information contained in the experiment json file. This file contains the percentage of overlap during acquisitions between tiles and the number of rows and columns of tiles, as well as the reading order (by default in snake row order). We have made here the controversial choice to generate one mosaic per channel and cycle, instead of tiles (regions in MAV) due to the usage of the MAV software provided by Akoya Biosciences, free but not open source, afterwards ( Figure 6 (J)), which was re-correcting the regions’ positions.

**Step 7: Segmentation**

Nuclei are segmented from the nuclei stained channel of the first cycle stitched image, on which all other cycles have been registered during step 4. We used Stardist pre-trained model "2D_versatile_fluo" with image normalisation ^19, 20^ to segment the nuclei as exemplified in Figure 6 (K)). An imageJ ^22^ set of ROIs is then generated and saved as zip.

**Step 8: Create Mask from ROI**

This optional step allows the creation of an RGB image corresponding to the segmented nuclei perimeter with a correct naming for visualisation only.

**Step 9: Measure**

Every cell is analyzed as an ImageJ ROI using the ImageJ analyzer plugin to measure their spatial location and intensity values, but also other parameters that we did not use in the analysis, but which could be of interest for other analysis such as nuclei shape descriptors, orientation, intensity statistics in every channel. These measurements are then stored in both a csv file compatible with the MAV software, and in standard FCS format using the CSVtoFCS functionalities offered by the Flow Cytometry Data standard project ^21^.

**Step 10: Create MAV Project**

This last optional step allows the use of the MAV software afterwards, as in our lab routine workflow usage of the PhenoCycler *^TM^*. This simply reorganised our output to make it compatible with the expected input experiment and file organisation for MAV.

### Post-processing analysis

For the clustering analysis in this paper, we used the freely available CODEX MAV, provided by Akoya Biosciences as a Fiji plugin. All files are prepared in MAV compatible format by the last step of our workflow. The directory selected as output directory can then be used directly in the "open experiment" field from MAV. Note that any other analysis tools could be used, including histocat ^23^, qupath ^24^ or other tools, as well as any population analysis based tools usually used in cytometry thanks to the FCS output.

## Conclusions

In this paper we present an open source workflow, based on an optimised sequential arrangement of existing steps. This workflow is now used routinely in our lab, replacing the commercial solution provided by the vendor and solving the problems encountered of stitching or intensity normalisation. Recently a generalist software suite was implemented for multiplexed data processing ^25^, which also incorporated the processing step ^26^. The MCMicro software is conceived in order to be ready to host different implementation of steps because it is based on Galaxy and Nextflow. Our command line implementation would ease this implementation in the future, and would also facilitate the extension of usage to other multiplexed tissue imaging systems. However we provide here an out of the shell tool for other labs using the PhenoCycler *^TM^* that may fix problems encountered.

## Data Availability

Example data are available from: https://doi.org/10.5281/zenodo.6461611 ^27^. This project contains the following underlying data in one 6.5 Gb zipped file. The imaging was generated from a larger acquisition by sub-selecting 6 cycles and 4 fields of view by rewriting the metadata files accordingly. 6 cycles, organised in folders by cycles, each folder 1.37Gb. Each folder contains 2D tif experimentname_image -position1-4_Z01-11_CH1-4.tif (176 files in each folder). 2048x2048 image in 16 bits; each image 8Mb Experiment.json was generated by the PhenoCycler *^TM^* acquisition and modified. Note that the path under the experiment json has no importance, but the raw data should be at the same level. channelNames.txt contains the list of fluorescent reporters used. Blank means no reporters. Licence: Creative Commons Attribution 4.0 International.

## Software availability

- Software v 1.0 compiled for Windows available from: https://doi.org/10.5281/zenodo.6773173
- Source code available from: https://gitlab.in2p3.fr/micropicell/multiplexprocessor
- Archived source code at time of publication: https://gitlab.in2p3.fr/micropicell/multiplexprocessor/-/tree/7eaa31f285f255f5f1f371bad58003e8eaa94d33
- License: GNU GENERAL PUBLIC LICENSE Version 3.
