# Supplementary material for: A flexible open-source processing workflow for multiplexed fluorescence imaging based on cycles
Source: F1000Res. 2022 Sep 29;11:1121. [Version 1] doi: 10.12688/f1000research.124990.1 (PMC10799232; doi:10.12688/f1000research.124990.1)
Supplement: Peer review history

# Reviewer report: Reviewer response for version 1

**DOI:** 10.5256/f1000research.137248.r230517

**Published:** 2024-01-19

**Authors:** Asier Antoranz

**Affiliations:** 1 Katholieke Universiteit Leuven, Leuven, Flanders, Belgium

Summary

 The authors present a computational workflow for the analysis of cyclic multiplexing images. They pipeline cover from the raw data acquisition to the segmentation based on a nuclear staining. Their implementation is similar to that of the CODEX Processor software provided by the vendor with a few improvements regarding stitching, intensity normalization, and segmentation.

 This is a timely and interesting work since the analysis of multiplexing images remains challenging and commercial platforms do not often perform properly. 

 Comments

 The description of the software tool is very shallow. They provide a wiki page, but this is not extensively descriptive.

 In the introduction, the authors mention they introduce improvements with respect to the commercial software. However, all the results are presented visually (no quantifications) and are based on a single tonsil image.

 The authors mention that the workflow is parameter free. However, it seems that a large number of parameters have been hard coded in the individual steps which can reduce generalizability to other imaging platforms, tissue types.

Are the conclusions about the tool and its performance adequately supported by the findings presented in the article?

Yes

Is the rationale for developing the new software tool clearly explained?

Yes

Is the description of the software tool technically sound?

Partly

Are sufficient details of the code, methods and analysis (if applicable) provided to allow replication of the software development and its use by others?

Yes

Is sufficient information provided to allow interpretation of the expected output datasets and any results generated using the tool?

Partly

Reviewer Expertise:

Spatial -omics, bioinformatics

I confirm that I have read this submission and believe that I have an appropriate level of expertise to confirm that it is of an acceptable scientific standard, however I have significant reservations, as outlined above.

# Reviewer report: Reviewer response for version 1

**DOI:** 10.5256/f1000research.137248.r230519

**Published:** 2023-12-28

**Authors:** Mark Zaidi

**Affiliations:** 1 University of Toronto, Toronto, Ontario, Canada

This paper essentially presents a new open-source (excellent!) software workflow for processing multiplexed fluorescence images. It addresses the challenges in existing commercial software, particularly around image stitching, intensity normalization, and segmentation. The authors demonstrate the workflow using the PhenoCycler (formerly CODEX) system, and compare its performance with the commercial processor, highlighting improvements in various processing aspects.

 Some of it's strengths are that this is open-source, adaptable to other cyclic imaging systems (not just PhenoCycler), and does a head-to-head comparison with commercial software.

 Some of the weaknesses include that they're not using the latest version of the CODEX processor for comparison (paper shows v1.8.2, newest release is 1.8.3). This should not be considered a valid negative criticism, because in the period during manuscript write up, the commercial software profiled in this study has been updated. It is not feasible to expect this manuscript to be constantly revised for each software release. Instead, the authors should take note of the differences between 1.8.2 and the current release, to see if any of their criticisms have been resolved in the new release (such as improved stitching, if it has been). Also, the repository has not been sufficiently populated with documentation and code necessary to replicate this study. Under post-processing analysis, histoCAT and QuPath should have the correct case sensitivity in its spelling.

 Fig.2C - please include arrows highlighting where the defect in stitching is. While it seems quite apparent (huge cross in the middle of the FOV), Fig. 2D seems to have an additional channel displayed not present in Fig.2C which may confuse the reader as to what this defect is. Also, consider increasing the font size for this and all subsequent figures (fig 1 is good).

 Aside from these otherwise minor issues, I don't see anything that would bar this manuscript from being published. The meticulous nature of the author in describing the technical detail at each step of the workflow, is greatly appreciated and seldom seen in similar studies. Running this pipeline on a laptop highlights how robust this workflow is, in its capacity to operate with limited computational resources. The CLI interface allows this tool to run in high-performance headless environments such as a SLURM cluster, highlighting its potential for scalability. Future studies can include integrating this workflow as an extension to other java-based applications such as QuPath. Excellent work!

Are the conclusions about the tool and its performance adequately supported by the findings presented in the article?

Yes

Is the rationale for developing the new software tool clearly explained?

Yes

Is the description of the software tool technically sound?

Yes

Are sufficient details of the code, methods and analysis (if applicable) provided to allow replication of the software development and its use by others?

Partly

Is sufficient information provided to allow interpretation of the expected output datasets and any results generated using the tool?

Yes

Reviewer Expertise:

Multiplexed Immunohistochemistry, Image Analysis, Digital Pathology, Image Registration, Pipeline Development

I confirm that I have read this submission and believe that I have an appropriate level of expertise to confirm that it is of an acceptable scientific standard.

# Reviewer report: Reviewer response for version 1

**DOI:** 10.5256/f1000research.137248.r152007

**Published:** 2022-10-21

**Authors:** Anna H. Klemm

**Affiliations:** 1 Department of Information Technology and SciLifeLab BioImage Informatics Facility, Uppsala University, Uppsala, Sweden

The authors present a pipeline for PhenoCycler(CODEX)/similarly structured data sets that run preprocessing, segmentation, and parameter extraction using open-source tools. Based on a limited demonstration in Fig.2 it seems to perform better than the vendor-provided software. Also, using open-source, free tools come with the advantages of higher availability and the possibility to adjust to own needs. The description of the tool is easy to follow and it comes with an example dataset and description to run the pipeline on this dataset.

**Minor comments to the text:**

- Fig 1A: z-stacks should be indicated by either drawing an axis or visualizing bigger steps between slices.
- Stitching: How big is the typical translation needed and how much is a typical overlap?
- Fig. 6: Show B, C, J, and L with comparable adjusted brightness/contrast in order to better inspect the contrast.
- Registration/corrections: Eventually, it could help to include a table with all cycles and recordings for displaying which images were used for registration, etc.
- Post-processing/Fig 3C: I suggest including a short description of how cell classification was done.

**Tool:**

 I run the binary version from https://zenodo.org/record/6773173#.Y1KHQWdBxD8 on the example data, starting with the deconvolved data.

**Comments:**

- Running it requires Java 11. The information could be found on https://gitlab.in2p3.fr/micropicell/multiplexprocessor/-/tree/master/, but not on the instruction of the binary version or the workflow description so it should be included there.
- Workflow description https://gitlab.in2p3.fr/micropicell/multiplexprocessor/-/wikis/MultiplexProcessor-Usage-documentation ends with Step 6: Stitching.
- Running the processor on the example data generated stitched datasets, but no further output (segmentation, MAV project, FCS file).
- The output stitched results don’t show the expected quality - see image.
- There is relatively little dialog when running the processor. Some brief information about the progress of processing would help.
- https://gitlab.in2p3.fr/micropicell/multiplexprocessor/-/tree/master/ misses description of Usage and Codex Splitter.

This needs to be double-checked before indexing.

Are the conclusions about the tool and its performance adequately supported by the findings presented in the article?

Yes

Is the rationale for developing the new software tool clearly explained?

Yes

Is the description of the software tool technically sound?

Yes

Are sufficient details of the code, methods and analysis (if applicable) provided to allow replication of the software development and its use by others?

Partly

Is sufficient information provided to allow interpretation of the expected output datasets and any results generated using the tool?

Yes

Reviewer Expertise:

Bioimage analysis

I confirm that I have read this submission and believe that I have an appropriate level of expertise to confirm that it is of an acceptable scientific standard, however I have significant reservations, as outlined above.

# Response

**Published:** 2022-10-28

**Authors:** Perrine Paul-Gilloteaux

**Affiliations:** University of Nantes, France

Dear Dr. Klemm,

 Many thanks for your constructive and useful comments. Before answering your review point by point and providing an adequately revised new version, could you upload or copy the log and error files that you should find in the same directory as the binary software? Indeed, I was not able to reproduce the bug reported about the stitching having crashed, these log files would be very useful. 

 Best regards,

 Perrine Paul-Gilloteaux

# Response

**Published:** 2022-11-08

**Authors:** Anna Klemm

Yes, happy to do so. Please let me know if you need more information. 

 Error message:

 okt. 21, 2022 3:53:18 EM com.sun.javafx.application.PlatformImpl startup

 WARNING: Unsupported JavaFX configuration: classes were loaded from 'unnamed module @3c95b7a5'

 java.util.concurrent.ExecutionException: java.lang.OutOfMemoryError

     at java.base/java.util.concurrent.ForkJoinTask.get(ForkJoinTask.java:1006)

     at fr.univ_nantes.codex_processor.gui.DatasetExtendedDepthOfFieldProcessor.run(DatasetExtendedDepthOfFieldProcessor.java:69)

     at fr.univ_nantes.codex_processor.gui.MainController$1.handle(MainController.java:96)

     at fr.univ_nantes.codex_processor.gui.MainController$1.handle(MainController.java:64)

     at com.sun.javafx.event.CompositeEventHandler.dispatchBubblingEvent(CompositeEventHandler.java:86)

     at com.sun.javafx.event.EventHandlerManager.dispatchBubblingEvent(EventHandlerManager.java:234)

     at com.sun.javafx.event.EventHandlerManager.dispatchBubblingEvent(EventHandlerManager.java:191)

     at com.sun.javafx.event.CompositeEventDispatcher.dispatchBubblingEvent(CompositeEventDispatcher.java:59)

     at com.sun.javafx.event.BasicEventDispatcher.dispatchEvent(BasicEventDispatcher.java:58)

     at com.sun.javafx.event.EventDispatchChainImpl.dispatchEvent(EventDispatchChainImpl.java:114)

     at com.sun.javafx.event.BasicEventDispatcher.dispatchEvent(BasicEventDispatcher.java:56)

     at com.sun.javafx.event.EventDispatchChainImpl.dispatchEvent(EventDispatchChainImpl.java:114)

     at com.sun.javafx.event.BasicEventDispatcher.dispatchEvent(BasicEventDispatcher.java:56)

     at com.sun.javafx.event.EventDispatchChainImpl.dispatchEvent(EventDispatchChainImpl.java:114)

     at com.sun.javafx.event.EventUtil.fireEventImpl(EventUtil.java:74)

     at com.sun.javafx.event.EventUtil.fireEvent(EventUtil.java:49)

     at javafx.event.Event.fireEvent(Event.java:198)

     at javafx.scene.Node.fireEvent(Node.java:8889)

     at javafx.scene.control.Button.fire(Button.java:203)

     at com.sun.javafx.scene.control.behavior.ButtonBehavior.mouseReleased(ButtonBehavior.java:208)

     at com.sun.javafx.scene.control.inputmap.InputMap.handle(InputMap.java:274)

     at com.sun.javafx.event.CompositeEventHandler$NormalEventHandlerRecord.handleBubblingEvent(CompositeEventHandler.java:247)

     at com.sun.javafx.event.CompositeEventHandler.dispatchBubblingEvent(CompositeEventHandler.java:80)

     at com.sun.javafx.event.EventHandlerManager.dispatchBubblingEvent(EventHandlerManager.java:234)

     at com.sun.javafx.event.EventHandlerManager.dispatchBubblingEvent(EventHandlerManager.java:191)

     at com.sun.javafx.event.CompositeEventDispatcher.dispatchBubblingEvent(CompositeEventDispatcher.java:59)

     at com.sun.javafx.event.BasicEventDispatcher.dispatchEvent(BasicEventDispatcher.java:58)

     at com.sun.javafx.event.EventDispatchChainImpl.dispatchEvent(EventDispatchChainImpl.java:114)

     at com.sun.javafx.event.BasicEventDispatcher.dispatchEvent(BasicEventDispatcher.java:56)

     at com.sun.javafx.event.EventDispatchChainImpl.dispatchEvent(EventDispatchChainImpl.java:114)

     at com.sun.javafx.event.BasicEventDispatcher.dispatchEvent(BasicEventDispatcher.java:56)

     at com.sun.javafx.event.EventDispatchChainImpl.dispatchEvent(EventDispatchChainImpl.java:114)

     at com.sun.javafx.event.EventUtil.fireEventImpl(EventUtil.java:74)

     at com.sun.javafx.event.EventUtil.fireEvent(EventUtil.java:54)

     at javafx.event.Event.fireEvent(Event.java:198)

     at javafx.scene.Scene$MouseHandler.process(Scene.java:3856)

     at javafx.scene.Scene.processMouseEvent(Scene.java:1851)

     at javafx.scene.Scene$ScenePeerListener.mouseEvent(Scene.java:2584)

     at com.sun.javafx.tk.quantum.GlassViewEventHandler$MouseEventNotification.run(GlassViewEventHandler.java:409)

     at com.sun.javafx.tk.quantum.GlassViewEventHandler$MouseEventNotification.run(GlassViewEventHandler.java:299)

     at java.base/java.security.AccessController.doPrivileged(Native Method)

     at com.sun.javafx.tk.quantum.GlassViewEventHandler.lambda$handleMouseEvent$2(GlassViewEventHandler.java:447)

     at com.sun.javafx.tk.quantum.QuantumToolkit.runWithoutRenderLock(QuantumToolkit.java:412)

     at com.sun.javafx.tk.quantum.GlassViewEventHandler.handleMouseEvent(GlassViewEventHandler.java:446)

     at com.sun.glass.ui.View.handleMouseEvent(View.java:556)

     at com.sun.glass.ui.View.notifyMouse(View.java:942)

     at com.sun.glass.ui.win.WinApplication._runLoop(Native Method)

     at com.sun.glass.ui.win.WinApplication.lambda$runLoop$3(WinApplication.java:174)

     at java.base/java.lang.Thread.run(Thread.java:829)

 Caused by: java.lang.OutOfMemoryError

     at java.base/jdk.internal.reflect.NativeConstructorAccessorImpl.newInstance0(Native Method)

     at java.base/jdk.internal.reflect.NativeConstructorAccessorImpl.newInstance(NativeConstructorAccessorImpl.java:62)

     at java.base/jdk.internal.reflect.DelegatingConstructorAccessorImpl.newInstance(DelegatingConstructorAccessorImpl.java:45)

     at java.base/java.lang.reflect.Constructor.newInstance(Constructor.java:490)

     at java.base/java.util.concurrent.ForkJoinTask.getThrowableException(ForkJoinTask.java:603)

     ... 49 more

 Caused by: java.lang.OutOfMemoryError: Java heap space

 java.util.concurrent.ExecutionException: java.lang.OutOfMemoryError

     at java.base/java.util.concurrent.ForkJoinTask.get(ForkJoinTask.java:1006)

     at fr.univ_nantes.codex_processor.gui.DatasetExtendedDepthOfFieldProcessor.run(DatasetExtendedDepthOfFieldProcessor.java:69)

     at fr.univ_nantes.codex_processor.gui.MainController$1.handle(MainController.java:96)

     at fr.univ_nantes.codex_processor.gui.MainController$1.handle(MainController.java:64)

     at com.sun.javafx.event.CompositeEventHandler.dispatchBubblingEvent(CompositeEventHandler.java:86)

     at com.sun.javafx.event.EventHandlerManager.dispatchBubblingEvent(EventHandlerManager.java:234)

     at com.sun.javafx.event.EventHandlerManager.dispatchBubblingEvent(EventHandlerManager.java:191)

     at com.sun.javafx.event.CompositeEventDispatcher.dispatchBubblingEvent(CompositeEventDispatcher.java:59)

     at com.sun.javafx.event.BasicEventDispatcher.dispatchEvent(BasicEventDispatcher.java:58)

     at com.sun.javafx.event.EventDispatchChainImpl.dispatchEvent(EventDispatchChainImpl.java:114)

     at com.sun.javafx.event.BasicEventDispatcher.dispatchEvent(BasicEventDispatcher.java:56)

     at com.sun.javafx.event.EventDispatchChainImpl.dispatchEvent(EventDispatchChainImpl.java:114)

     at com.sun.javafx.event.BasicEventDispatcher.dispatchEvent(BasicEventDispatcher.java:56)

     at com.sun.javafx.event.EventDispatchChainImpl.dispatchEvent(EventDispatchChainImpl.java:114)

     at com.sun.javafx.event.EventUtil.fireEventImpl(EventUtil.java:74)

     at com.sun.javafx.event.EventUtil.fireEvent(EventUtil.java:49)

     at javafx.event.Event.fireEvent(Event.java:198)

     at javafx.scene.Node.fireEvent(Node.java:8889)

     at javafx.scene.control.Button.fire(Button.java:203)

     at com.sun.javafx.scene.control.behavior.ButtonBehavior.mouseReleased(ButtonBehavior.java:208)

     at com.sun.javafx.scene.control.inputmap.InputMap.handle(InputMap.java:274)

     at com.sun.javafx.event.CompositeEventHandler$NormalEventHandlerRecord.handleBubblingEvent(CompositeEventHandler.java:247)

     at com.sun.javafx.event.CompositeEventHandler.dispatchBubblingEvent(CompositeEventHandler.java:80)

     at com.sun.javafx.event.EventHandlerManager.dispatchBubblingEvent(EventHandlerManager.java:234)

     at com.sun.javafx.event.EventHandlerManager.dispatchBubblingEvent(EventHandlerManager.java:191)

     at com.sun.javafx.event.CompositeEventDispatcher.dispatchBubblingEvent(CompositeEventDispatcher.java:59)

     at com.sun.javafx.event.BasicEventDispatcher.dispatchEvent(BasicEventDispatcher.java:58)

     at com.sun.javafx.event.EventDispatchChainImpl.dispatchEvent(EventDispatchChainImpl.java:114)

     at com.sun.javafx.event.BasicEventDispatcher.dispatchEvent(BasicEventDispatcher.java:56)

     at com.sun.javafx.event.EventDispatchChainImpl.dispatchEvent(EventDispatchChainImpl.java:114)

     at com.sun.javafx.event.BasicEventDispatcher.dispatchEvent(BasicEventDispatcher.java:56)

     at com.sun.javafx.event.EventDispatchChainImpl.dispatchEvent(EventDispatchChainImpl.java:114)

     at com.sun.javafx.event.EventUtil.fireEventImpl(EventUtil.java:74)

     at com.sun.javafx.event.EventUtil.fireEvent(EventUtil.java:54)

     at javafx.event.Event.fireEvent(Event.java:198)

     at javafx.scene.Scene$MouseHandler.process(Scene.java:3856)

     at javafx.scene.Scene.processMouseEvent(Scene.java:1851)

     at javafx.scene.Scene$ScenePeerListener.mouseEvent(Scene.java:2584)

     at com.sun.javafx.tk.quantum.GlassViewEventHandler$MouseEventNotification.run(GlassViewEventHandler.java:409)

     at com.sun.javafx.tk.quantum.GlassViewEventHandler$MouseEventNotification.run(GlassViewEventHandler.java:299)

     at java.base/java.security.AccessController.doPrivileged(Native Method)

     at com.sun.javafx.tk.quantum.GlassViewEventHandler.lambda$handleMouseEvent$2(GlassViewEventHandler.java:447)

     at com.sun.javafx.tk.quantum.QuantumToolkit.runWithoutRenderLock(QuantumToolkit.java:412)

     at com.sun.javafx.tk.quantum.GlassViewEventHandler.handleMouseEvent(GlassViewEventHandler.java:446)

     at com.sun.glass.ui.View.handleMouseEvent(View.java:556)

     at com.sun.glass.ui.View.notifyMouse(View.java:942)

     at com.sun.glass.ui.win.WinApplication._runLoop(Native Method)

     at com.sun.glass.ui.win.WinApplication.lambda$runLoop$3(WinApplication.java:174)

     at java.base/java.lang.Thread.run(Thread.java:829)

 Caused by: java.lang.OutOfMemoryError

     at java.base/jdk.internal.reflect.NativeConstructorAccessorImpl.newInstance0(Native Method)

     at java.base/jdk.internal.reflect.NativeConstructorAccessorImpl.newInstance(NativeConstructorAccessorImpl.java:62)

     at java.base/jdk.internal.reflect.DelegatingConstructorAccessorImpl.newInstance(DelegatingConstructorAccessorImpl.java:45)

     at java.base/java.lang.reflect.Constructor.newInstance(Constructor.java:490)

     at java.base/java.util.concurrent.ForkJoinTask.getThrowableException(ForkJoinTask.java:603)

     ... 49 more

 Caused by: java.lang.OutOfMemoryError: Java heap space

 java.util.concurrent.ExecutionException: java.lang.OutOfMemoryError

     at java.base/java.util.concurrent.ForkJoinTask.get(ForkJoinTask.java:1006)

     at fr.univ_nantes.codex_processor.gui.DatasetExtendedDepthOfFieldProcessor.run(DatasetExtendedDepthOfFieldProcessor.java:69)

     at fr.univ_nantes.codex_processor.gui.MainController$1.handle(MainController.java:96)

     at fr.univ_nantes.codex_processor.gui.MainController$1.handle(MainController.java:64)

     at com.sun.javafx.event.CompositeEventHandler.dispatchBubblingEvent(CompositeEventHandler.java:86)

     at com.sun.javafx.event.EventHandlerManager.dispatchBubblingEvent(EventHandlerManager.java:234)

     at com.sun.javafx.event.EventHandlerManager.dispatchBubblingEvent(EventHandlerManager.java:191)

     at com.sun.javafx.event.CompositeEventDispatcher.dispatchBubblingEvent(CompositeEventDispatcher.java:59)

     at com.sun.javafx.event.BasicEventDispatcher.dispatchEvent(BasicEventDispatcher.java:58)

     at com.sun.javafx.event.EventDispatchChainImpl.dispatchEvent(EventDispatchChainImpl.java:114)

     at com.sun.javafx.event.BasicEventDispatcher.dispatchEvent(BasicEventDispatcher.java:56)

     at com.sun.javafx.event.EventDispatchChainImpl.dispatchEvent(EventDispatchChainImpl.java:114)

     at com.sun.javafx.event.BasicEventDispatcher.dispatchEvent(BasicEventDispatcher.java:56)

     at com.sun.javafx.event.EventDispatchChainImpl.dispatchEvent(EventDispatchChainImpl.java:114)

     at com.sun.javafx.event.EventUtil.fireEventImpl(EventUtil.java:74)

     at com.sun.javafx.event.EventUtil.fireEvent(EventUtil.java:49)

     at javafx.event.Event.fireEvent(Event.java:198)

     at javafx.scene.Node.fireEvent(Node.java:8889)

     at javafx.scene.control.Button.fire(Button.java:203)

     at com.sun.javafx.scene.control.behavior.ButtonBehavior.mouseReleased(ButtonBehavior.java:208)

     at com.sun.javafx.scene.control.inputmap.InputMap.handle(InputMap.java:274)

     at com.sun.javafx.event.CompositeEventHandler$NormalEventHandlerRecord.handleBubblingEvent(CompositeEventHandler.java:247)

     at com.sun.javafx.event.CompositeEventHandler.dispatchBubblingEvent(CompositeEventHandler.java:80)

     at com.sun.javafx.event.EventHandlerManager.dispatchBubblingEvent(EventHandlerManager.java:234)

     at com.sun.javafx.event.EventHandlerManager.dispatchBubblingEvent(EventHandlerManager.java:191)

     at com.sun.javafx.event.CompositeEventDispatcher.dispatchBubblingEvent(CompositeEventDispatcher.java:59)

     at com.sun.javafx.event.BasicEventDispatcher.dispatchEvent(BasicEventDispatcher.java:58)

     at com.sun.javafx.event.EventDispatchChainImpl.dispatchEvent(EventDispatchChainImpl.java:114)

     at com.sun.javafx.event.BasicEventDispatcher.dispatchEvent(BasicEventDispatcher.java:56)

     at com.sun.javafx.event.EventDispatchChainImpl.dispatchEvent(EventDispatchChainImpl.java:114)

     at com.sun.javafx.event.BasicEventDispatcher.dispatchEvent(BasicEventDispatcher.java:56)

     at com.sun.javafx.event.EventDispatchChainImpl.dispatchEvent(EventDispatchChainImpl.java:114)

     at com.sun.javafx.event.EventUtil.fireEventImpl(EventUtil.java:74)

     at com.sun.javafx.event.EventUtil.fireEvent(EventUtil.java:54)

     at javafx.event.Event.fireEvent(Event.java:198)

     at javafx.scene.Scene$MouseHandler.process(Scene.java:3856)

     at javafx.scene.Scene.processMouseEvent(Scene.java:1851)

     at javafx.scene.Scene$ScenePeerListener.mouseEvent(Scene.java:2584)

     at com.sun.javafx.tk.quantum.GlassViewEventHandler$MouseEventNotification.run(GlassViewEventHandler.java:409)

     at com.sun.javafx.tk.quantum.GlassViewEventHandler$MouseEventNotification.run(GlassViewEventHandler.java:299)

     at java.base/java.security.AccessController.doPrivileged(Native Method)

     at com.sun.javafx.tk.quantum.GlassViewEventHandler.lambda$handleMouseEvent$2(GlassViewEventHandler.java:447)

     at com.sun.javafx.tk.quantum.QuantumToolkit.runWithoutRenderLock(QuantumToolkit.java:412)

     at com.sun.javafx.tk.quantum.GlassViewEventHandler.handleMouseEvent(GlassViewEventHandler.java:446)

     at com.sun.glass.ui.View.handleMouseEvent(View.java:556)

     at com.sun.glass.ui.View.notifyMouse(View.java:942)

     at com.sun.glass.ui.win.WinApplication._runLoop(Native Method)

     at com.sun.glass.ui.win.WinApplication.lambda$runLoop$3(WinApplication.java:174)

     at java.base/java.lang.Thread.run(Thread.java:829)

 Caused by: java.lang.OutOfMemoryError

     at java.base/jdk.internal.reflect.NativeConstructorAccessorImpl.newInstance0(Native Method)

     at java.base/jdk.internal.reflect.NativeConstructorAccessorImpl.newInstance(NativeConstructorAccessorImpl.java:62)

     at java.base/jdk.internal.reflect.DelegatingConstructorAccessorImpl.newInstance(DelegatingConstructorAccessorImpl.java:45)

     at java.base/java.lang.reflect.Constructor.newInstance(Constructor.java:490)

     at java.base/java.util.concurrent.ForkJoinTask.getThrowableException(ForkJoinTask.java:603)

     ... 49 more

 Caused by: java.lang.OutOfMemoryError: Java heap space

 java.util.concurrent.ExecutionException: java.lang.OutOfMemoryError

     at java.base/java.util.concurrent.ForkJoinTask.get(ForkJoinTask.java:1006)

     at fr.univ_nantes.codex_processor.gui.DatasetExtendedDepthOfFieldProcessor.run(DatasetExtendedDepthOfFieldProcessor.java:69)

     at fr.univ_nantes.codex_processor.gui.MainController$1.handle(MainController.java:96)

     at fr.univ_nantes.codex_processor.gui.MainController$1.handle(MainController.java:64)

     at com.sun.javafx.event.CompositeEventHandler.dispatchBubblingEvent(CompositeEventHandler.java:86)

     at com.sun.javafx.event.EventHandlerManager.dispatchBubblingEvent(EventHandlerManager.java:234)

     at com.sun.javafx.event.EventHandlerManager.dispatchBubblingEvent(EventHandlerManager.java:191)

     at com.sun.javafx.event.CompositeEventDispatcher.dispatchBubblingEvent(CompositeEventDispatcher.java:59)

     at com.sun.javafx.event.BasicEventDispatcher.dispatchEvent(BasicEventDispatcher.java:58)

     at com.sun.javafx.event.EventDispatchChainImpl.dispatchEvent(EventDispatchChainImpl.java:114)

     at com.sun.javafx.event.BasicEventDispatcher.dispatchEvent(BasicEventDispatcher.java:56)

     at com.sun.javafx.event.EventDispatchChainImpl.dispatchEvent(EventDispatchChainImpl.java:114)

     at com.sun.javafx.event.BasicEventDispatcher.dispatchEvent(BasicEventDispatcher.java:56)

     at com.sun.javafx.event.EventDispatchChainImpl.dispatchEvent(EventDispatchChainImpl.java:114)

     at com.sun.javafx.event.EventUtil.fireEventImpl(EventUtil.java:74)

     at com.sun.javafx.event.EventUtil.fireEvent(EventUtil.java:49)

     at javafx.event.Event.fireEvent(Event.java:198)

     at javafx.scene.Node.fireEvent(Node.java:8889)

     at javafx.scene.control.Button.fire(Button.java:203)

     at com.sun.javafx.scene.control.behavior.ButtonBehavior.mouseReleased(ButtonBehavior.java:208)

     at com.sun.javafx.scene.control.inputmap.InputMap.handle(InputMap.java:274)

     at com.sun.javafx.event.CompositeEventHandler$NormalEventHandlerRecord.handleBubblingEvent(CompositeEventHandler.java:247)

     at com.sun.javafx.event.CompositeEventHandler.dispatchBubblingEvent(CompositeEventHandler.java:80)

     at com.sun.javafx.event.EventHandlerManager.dispatchBubblingEvent(EventHandlerManager.java:234)

     at com.sun.javafx.event.EventHandlerManager.dispatchBubblingEvent(EventHandlerManager.java:191)

     at com.sun.javafx.event.CompositeEventDispatcher.dispatchBubblingEvent(CompositeEventDispatcher.java:59)

     at com.sun.javafx.event.BasicEventDispatcher.dispatchEvent(BasicEventDispatcher.java:58)

     at com.sun.javafx.event.EventDispatchChainImpl.dispatchEvent(EventDispatchChainImpl.java:114)

     at com.sun.javafx.event.BasicEventDispatcher.dispatchEvent(BasicEventDispatcher.java:56)

     at com.sun.javafx.event.EventDispatchChainImpl.dispatchEvent(EventDispatchChainImpl.java:114)

     at com.sun.javafx.event.BasicEventDispatcher.dispatchEvent(BasicEventDispatcher.java:56)

     at com.sun.javafx.event.EventDispatchChainImpl.dispatchEvent(EventDispatchChainImpl.java:114)

     at com.sun.javafx.event.EventUtil.fireEventImpl(EventUtil.java:74)

     at com.sun.javafx.event.EventUtil.fireEvent(EventUtil.java:54)

     at javafx.event.Event.fireEvent(Event.java:198)

     at javafx.scene.Scene$MouseHandler.process(Scene.java:3856)

     at javafx.scene.Scene.processMouseEvent(Scene.java:1851)

     at javafx.scene.Scene$ScenePeerListener.mouseEvent(Scene.java:2584)

     at com.sun.javafx.tk.quantum.GlassViewEventHandler$MouseEventNotification.run(GlassViewEventHandler.java:409)

     at com.sun.javafx.tk.quantum.GlassViewEventHandler$MouseEventNotification.run(GlassViewEventHandler.java:299)

     at java.base/java.security.AccessController.doPrivileged(Native Method)

     at com.sun.javafx.tk.quantum.GlassViewEventHandler.lambda$handleMouseEvent$2(GlassViewEventHandler.java:447)

     at com.sun.javafx.tk.quantum.QuantumToolkit.runWithoutRenderLock(QuantumToolkit.java:412)

     at com.sun.javafx.tk.quantum.GlassViewEventHandler.handleMouseEvent(GlassViewEventHandler.java:446)

     at com.sun.glass.ui.View.handleMouseEvent(View.java:556)

     at com.sun.glass.ui.View.notifyMouse(View.java:942)

     at com.sun.glass.ui.win.WinApplication._runLoop(Native Method)

     at com.sun.glass.ui.win.WinApplication.lambda$runLoop$3(WinApplication.java:174)

     at java.base/java.lang.Thread.run(Thread.java:829)

 Caused by: java.lang.OutOfMemoryError

     at java.base/jdk.internal.reflect.NativeConstructorAccessorImpl.newInstance0(Native Method)

     at java.base/jdk.internal.reflect.NativeConstructorAccessorImpl.newInstance(NativeConstructorAccessorImpl.java:62)

     at java.base/jdk.internal.reflect.DelegatingConstructorAccessorImpl.newInstance(DelegatingConstructorAccessorImpl.java:45)

     at java.base/java.lang.reflect.Constructor.newInstance(Constructor.java:490)

     at java.base/java.util.concurrent.ForkJoinTask.getThrowableException(ForkJoinTask.java:603)

     ... 49 more

 Caused by: java.lang.OutOfMemoryError: Java heap space

     at wavelets.ImageAccess.(ImageAccess.java:165)

     at wavelets.ImageAccess.duplicate(ImageAccess.java:294)

     at wavelets.ComplexWavelet.analysis(ComplexWavelet.java:54)

     at edf.EdfComplexWavelets.process(EdfComplexWavelets.java:91)

     at fr.univ_nantes.codex_processor.core.extended_depth_of_field.ExtendedDepthOfField.process(ExtendedDepthOfField.java:73)

     at fr.univ_nantes.codex_processor.core.extended_depth_of_field.ExtendedDepthOfFieldProcessor.run(ExtendedDepthOfFieldProcessor.java:23)

     at fr.univ_nantes.codex_processor.gui.DatasetExtendedDepthOfFieldProcessor.lambda$run$0(DatasetExtendedDepthOfFieldProcessor.java:58)

     at fr.univ_nantes.codex_processor.gui.DatasetExtendedDepthOfFieldProcessor$$Lambda$408/0x0000000800389440.run(Unknown Source)

     at java.base/java.util.concurrent.ForkJoinTask$AdaptedRunnableAction.exec(ForkJoinTask.java:1407)

     at java.base/java.util.concurrent.ForkJoinTask.doExec(ForkJoinTask.java:290)

     at java.base/java.util.concurrent.ForkJoinPool$WorkQueue.topLevelExec(ForkJoinPool.java:1020)

     at java.base/java.util.concurrent.ForkJoinPool.scan(ForkJoinPool.java:1656)

     at java.base/java.util.concurrent.ForkJoinPool.runWorker(ForkJoinPool.java:1594)

     at java.base/java.util.concurrent.ForkJoinWorkerThread.run(ForkJoinWorkerThread.java:183)

 java.util.concurrent.ExecutionException: java.lang.OutOfMemoryError

     at java.base/java.util.concurrent.ForkJoinTask.get(ForkJoinTask.java:1006)

     at fr.univ_nantes.codex_processor.gui.DatasetExtendedDepthOfFieldProcessor.run(DatasetExtendedDepthOfFieldProcessor.java:69)

     at fr.univ_nantes.codex_processor.gui.MainController$1.handle(MainController.java:96)

     at fr.univ_nantes.codex_processor.gui.MainController$1.handle(MainController.java:64)

     at com.sun.javafx.event.CompositeEventHandler.dispatchBubblingEvent(CompositeEventHandler.java:86)

     at com.sun.javafx.event.EventHandlerManager.dispatchBubblingEvent(EventHandlerManager.java:234)

     at com.sun.javafx.event.EventHandlerManager.dispatchBubblingEvent(EventHandlerManager.java:191)

     at com.sun.javafx.event.CompositeEventDispatcher.dispatchBubblingEvent(CompositeEventDispatcher.java:59)

     at com.sun.javafx.event.BasicEventDispatcher.dispatchEvent(BasicEventDispatcher.java:58)

     at com.sun.javafx.event.EventDispatchChainImpl.dispatchEvent(EventDispatchChainImpl.java:114)

     at com.sun.javafx.event.BasicEventDispatcher.dispatchEvent(BasicEventDispatcher.java:56)

     at com.sun.javafx.event.EventDispatchChainImpl.dispatchEvent(EventDispatchChainImpl.java:114)

     at com.sun.javafx.event.BasicEventDispatcher.dispatchEvent(BasicEventDispatcher.java:56)

     at com.sun.javafx.event.EventDispatchChainImpl.dispatchEvent(EventDispatchChainImpl.java:114)

     at com.sun.javafx.event.EventUtil.fireEventImpl(EventUtil.java:74)

     at com.sun.javafx.event.EventUtil.fireEvent(EventUtil.java:49)

     at javafx.event.Event.fireEvent(Event.java:198)

     at javafx.scene.Node.fireEvent(Node.java:8889)

     at javafx.scene.control.Button.fire(Button.java:203)

     at com.sun.javafx.scene.control.behavior.ButtonBehavior.mouseReleased(ButtonBehavior.java:208)

     at com.sun.javafx.scene.control.inputmap.InputMap.handle(InputMap.java:274)

     at com.sun.javafx.event.CompositeEventHandler$NormalEventHandlerRecord.handleBubblingEvent(CompositeEventHandler.java:247)

     at com.sun.javafx.event.CompositeEventHandler.dispatchBubblingEvent(CompositeEventHandler.java:80)

     at com.sun.javafx.event.EventHandlerManager.dispatchBubblingEvent(EventHandlerManager.java:234)

     at com.sun.javafx.event.EventHandlerManager.dispatchBubblingEvent(EventHandlerManager.java:191)

     at com.sun.javafx.event.CompositeEventDispatcher.dispatchBubblingEvent(CompositeEventDispatcher.java:59)

     at com.sun.javafx.event.BasicEventDispatcher.dispatchEvent(BasicEventDispatcher.java:58)

     at com.sun.javafx.event.EventDispatchChainImpl.dispatchEvent(EventDispatchChainImpl.java:114)

     at com.sun.javafx.event.BasicEventDispatcher.dispatchEvent(BasicEventDispatcher.java:56)

     at com.sun.javafx.event.EventDispatchChainImpl.dispatchEvent(EventDispatchChainImpl.java:114)

     at com.sun.javafx.event.BasicEventDispatcher.dispatchEvent(BasicEventDispatcher.java:56)

     at com.sun.javafx.event.EventDispatchChainImpl.dispatchEvent(EventDispatchChainImpl.java:114)

     at com.sun.javafx.event.EventUtil.fireEventImpl(EventUtil.java:74)

     at com.sun.javafx.event.EventUtil.fireEvent(EventUtil.java:54)

     at javafx.event.Event.fireEvent(Event.java:198)

     at javafx.scene.Scene$MouseHandler.process(Scene.java:3856)

     at javafx.scene.Scene.processMouseEvent(Scene.java:1851)

     at javafx.scene.Scene$ScenePeerListener.mouseEvent(Scene.java:2584)

     at com.sun.javafx.tk.quantum.GlassViewEventHandler$MouseEventNotification.run(GlassViewEventHandler.java:409)

     at com.sun.javafx.tk.quantum.GlassViewEventHandler$MouseEventNotification.run(GlassViewEventHandler.java:299)

     at java.base/java.security.AccessController.doPrivileged(Native Method)

     at com.sun.javafx.tk.quantum.GlassViewEventHandler.lambda$handleMouseEvent$2(GlassViewEventHandler.java:447)

     at com.sun.javafx.tk.quantum.QuantumToolkit.runWithoutRenderLock(QuantumToolkit.java:412)

     at com.sun.javafx.tk.quantum.GlassViewEventHandler.handleMouseEvent(GlassViewEventHandler.java:446)

     at com.sun.glass.ui.View.handleMouseEvent(View.java:556)

     at com.sun.glass.ui.View.notifyMouse(View.java:942)

     at com.sun.glass.ui.win.WinApplication._runLoop(Native Method)

     at com.sun.glass.ui.win.WinApplication.lambda$runLoop$3(WinApplication.java:174)

     at java.base/java.lang.Thread.run(Thread.java:829)

 Caused by: java.lang.OutOfMemoryError

     at java.base/jdk.internal.reflect.NativeConstructorAccessorImpl.newInstance0(Native Method)

     at java.base/jdk.internal.reflect.NativeConstructorAccessorImpl.newInstance(NativeConstructorAccessorImpl.java:62)

     at java.base/jdk.internal.reflect.DelegatingConstructorAccessorImpl.newInstance(DelegatingConstructorAccessorImpl.java:45)

     at java.base/java.lang.reflect.Constructor.newInstance(Constructor.java:490)

     at java.base/java.util.concurrent.ForkJoinTask.getThrowableException(ForkJoinTask.java:603)

     ... 49 more

 Caused by: java.lang.OutOfMemoryError: Java heap space

 java.util.concurrent.ExecutionException: java.lang.OutOfMemoryError

     at java.base/java.util.concurrent.ForkJoinTask.get(ForkJoinTask.java:1006)

     at fr.univ_nantes.codex_processor.gui.DatasetExtendedDepthOfFieldProcessor.run(DatasetExtendedDepthOfFieldProcessor.java:69)

     at fr.univ_nantes.codex_processor.gui.MainController$1.handle(MainController.java:96)

     at fr.univ_nantes.codex_processor.gui.MainController$1.handle(MainController.java:64)

     at com.sun.javafx.event.CompositeEventHandler.dispatchBubblingEvent(CompositeEventHandler.java:86)

     at com.sun.javafx.event.EventHandlerManager.dispatchBubblingEvent(EventHandlerManager.java:234)

     at com.sun.javafx.event.EventHandlerManager.dispatchBubblingEvent(EventHandlerManager.java:191)

     at com.sun.javafx.event.CompositeEventDispatcher.dispatchBubblingEvent(CompositeEventDispatcher.java:59)

     at com.sun.javafx.event.BasicEventDispatcher.dispatchEvent(BasicEventDispatcher.java:58)

     at com.sun.javafx.event.EventDispatchChainImpl.dispatchEvent(EventDispatchChainImpl.java:114)

     at com.sun.javafx.event.BasicEventDispatcher.dispatchEvent(BasicEventDispatcher.java:56)

     at com.sun.javafx.event.EventDispatchChainImpl.dispatchEvent(EventDispatchChainImpl.java:114)

     at com.sun.javafx.event.BasicEventDispatcher.dispatchEvent(BasicEventDispatcher.java:56)

     at com.sun.javafx.event.EventDispatchChainImpl.dispatchEvent(EventDispatchChainImpl.java:114)

     at com.sun.javafx.event.EventUtil.fireEventImpl(EventUtil.java:74)

     at com.sun.javafx.event.EventUtil.fireEvent(EventUtil.java:49)

     at javafx.event.Event.fireEvent(Event.java:198)

     at javafx.scene.Node.fireEvent(Node.java:8889)

     at javafx.scene.control.Button.fire(Button.java:203)

     at com.sun.javafx.scene.control.behavior.ButtonBehavior.mouseReleased(ButtonBehavior.java:208)

     at com.sun.javafx.scene.control.inputmap.InputMap.handle(InputMap.java:274)

     at com.sun.javafx.event.CompositeEventHandler$NormalEventHandlerRecord.handleBubblingEvent(CompositeEventHandler.java:247)

     at com.sun.javafx.event.CompositeEventHandler.dispatchBubblingEvent(CompositeEventHandler.java:80)

     at com.sun.javafx.event.EventHandlerManager.dispatchBubblingEvent(EventHandlerManager.java:234)

     at com.sun.javafx.event.EventHandlerManager.dispatchBubblingEvent(EventHandlerManager.java:191)

     at com.sun.javafx.event.CompositeEventDispatcher.dispatchBubblingEvent(CompositeEventDispatcher.java:59)

     at com.sun.javafx.event.BasicEventDispatcher.dispatchEvent(BasicEventDispatcher.java:58)

     at com.sun.javafx.event.EventDispatchChainImpl.dispatchEvent(EventDispatchChainImpl.java:114)

     at com.sun.javafx.event.BasicEventDispatcher.dispatchEvent(BasicEventDispatcher.java:56)

     at com.sun.javafx.event.EventDispatchChainImpl.dispatchEvent(EventDispatchChainImpl.java:114)

     at com.sun.javafx.event.BasicEventDispatcher.dispatchEvent(BasicEventDispatcher.java:56)

     at com.sun.javafx.event.EventDispatchChainImpl.dispatchEvent(EventDispatchChainImpl.java:114)

     at com.sun.javafx.event.EventUtil.fireEventImpl(EventUtil.java:74)

     at com.sun.javafx.event.EventUtil.fireEvent(EventUtil.java:54)

     at javafx.event.Event.fireEvent(Event.java:198)

     at javafx.scene.Scene$MouseHandler.process(Scene.java:3856)

     at javafx.scene.Scene.processMouseEvent(Scene.java:1851)

     at javafx.scene.Scene$ScenePeerListener.mouseEvent(Scene.java:2584)

     at com.sun.javafx.tk.quantum.GlassViewEventHandler$MouseEventNotification.run(GlassViewEventHandler.java:409)

     at com.sun.javafx.tk.quantum.GlassViewEventHandler$MouseEventNotification.run(GlassViewEventHandler.java:299)

     at java.base/java.security.AccessController.doPrivileged(Native Method)

     at com.sun.javafx.tk.quantum.GlassViewEventHandler.lambda$handleMouseEvent$2(GlassViewEventHandler.java:447)

     at com.sun.javafx.tk.quantum.QuantumToolkit.runWithoutRenderLock(QuantumToolkit.java:412)

     at com.sun.javafx.tk.quantum.GlassViewEventHandler.handleMouseEvent(GlassViewEventHandler.java:446)

     at com.sun.glass.ui.View.handleMouseEvent(View.java:556)

     at com.sun.glass.ui.View.notifyMouse(View.java:942)

     at com.sun.glass.ui.win.WinApplication._runLoop(Native Method)

     at com.sun.glass.ui.win.WinApplication.lambda$runLoop$3(WinApplication.java:174)

     at java.base/java.lang.Thread.run(Thread.java:829)

 Caused by: java.lang.OutOfMemoryError

     at java.base/jdk.internal.reflect.NativeConstructorAccessorImpl.newInstance0(Native Method)

     at java.base/jdk.internal.reflect.NativeConstructorAccessorImpl.newInstance(NativeConstructorAccessorImpl.java:62)

     at java.base/jdk.internal.reflect.DelegatingConstructorAccessorImpl.newInstance(DelegatingConstructorAccessorImpl.java:45)

     at java.base/java.lang.reflect.Constructor.newInstance(Constructor.java:490)

     at java.base/java.util.concurrent.ForkJoinTask.getThrowableException(ForkJoinTask.java:603)

     ... 49 more

 Caused by: java.lang.OutOfMemoryError: Java heap space

     at wavelets.ImageAccess.(ImageAccess.java:165)

     at wavelets.ImageAccess.duplicate(ImageAccess.java:294)

     at wavelets.ComplexWavelet.analysis(ComplexWavelet.java:54)

     at edf.EdfComplexWavelets.process(EdfComplexWavelets.java:91)

     at fr.univ_nantes.codex_processor.core.extended_depth_of_field.ExtendedDepthOfField.process(ExtendedDepthOfField.java:73)

     at fr.univ_nantes.codex_processor.core.extended_depth_of_field.ExtendedDepthOfFieldProcessor.run(ExtendedDepthOfFieldProcessor.java:23)

     at fr.univ_nantes.codex_processor.gui.DatasetExtendedDepthOfFieldProcessor.lambda$run$0(DatasetExtendedDepthOfFieldProcessor.java:58)

     at fr.univ_nantes.codex_processor.gui.DatasetExtendedDepthOfFieldProcessor$$Lambda$408/0x0000000800389440.run(Unknown Source)

     at java.base/java.util.concurrent.ForkJoinTask$AdaptedRunnableAction.exec(ForkJoinTask.java:1407)

     at java.base/java.util.concurrent.ForkJoinTask.doExec(ForkJoinTask.java:290)

     at java.base/java.util.concurrent.ForkJoinPool$WorkQueue.topLevelExec(ForkJoinPool.java:1020)

     at java.base/java.util.concurrent.ForkJoinPool.scan(ForkJoinPool.java:1656)

     at java.base/java.util.concurrent.ForkJoinPool.runWorker(ForkJoinPool.java:1594)

     at java.base/java.util.concurrent.ForkJoinWorkerThread.run(ForkJoinWorkerThread.java:183)

 java.util.concurrent.ExecutionException: java.lang.OutOfMemoryError

     at java.base/java.util.concurrent.ForkJoinTask.get(ForkJoinTask.java:1006)

     at fr.univ_nantes.codex_processor.gui.DatasetExtendedDepthOfFieldProcessor.run(DatasetExtendedDepthOfFieldProcessor.java:69)

     at fr.univ_nantes.codex_processor.gui.MainController$1.handle(MainController.java:96)

     at fr.univ_nantes.codex_processor.gui.MainController$1.handle(MainController.java:64)

     at com.sun.javafx.event.CompositeEventHandler.dispatchBubblingEvent(CompositeEventHandler.java:86)

     at com.sun.javafx.event.EventHandlerManager.dispatchBubblingEvent(EventHandlerManager.java:234)

     at com.sun.javafx.event.EventHandlerManager.dispatchBubblingEvent(EventHandlerManager.java:191)

     at com.sun.javafx.event.CompositeEventDispatcher.dispatchBubblingEvent(CompositeEventDispatcher.java:59)

     at com.sun.javafx.event.BasicEventDispatcher.dispatchEvent(BasicEventDispatcher.java:58)

     at com.sun.javafx.event.EventDispatchChainImpl.dispatchEvent(EventDispatchChainImpl.java:114)

     at com.sun.javafx.event.BasicEventDispatcher.dispatchEvent(BasicEventDispatcher.java:56)

     at com.sun.javafx.event.EventDispatchChainImpl.dispatchEvent(EventDispatchChainImpl.java:114)

     at com.sun.javafx.event.BasicEventDispatcher.dispatchEvent(BasicEventDispatcher.java:56)

     at com.sun.javafx.event.EventDispatchChainImpl.dispatchEvent(EventDispatchChainImpl.java:114)

     at com.sun.javafx.event.EventUtil.fireEventImpl(EventUtil.java:74)

     at com.sun.javafx.event.EventUtil.fireEvent(EventUtil.java:49)

     at javafx.event.Event.fireEvent(Event.java:198)

     at javafx.scene.Node.fireEvent(Node.java:8889)

     at javafx.scene.control.Button.fire(Button.java:203)

     at com.sun.javafx.scene.control.behavior.ButtonBehavior.mouseReleased(ButtonBehavior.java:208)

     at com.sun.javafx.scene.control.inputmap.InputMap.handle(InputMap.java:274)

     at com.sun.javafx.event.CompositeEventHandler$NormalEventHandlerRecord.handleBubblingEvent(CompositeEventHandler.java:247)

     at com.sun.javafx.event.CompositeEventHandler.dispatchBubblingEvent(CompositeEventHandler.java:80)

     at com.sun.javafx.event.EventHandlerManager.dispatchBubblingEvent(EventHandlerManager.java:234)

     at com.sun.javafx.event.EventHandlerManager.dispatchBubblingEvent(EventHandlerManager.java:191)

     at com.sun.javafx.event.CompositeEventDispatcher.dispatchBubblingEvent(CompositeEventDispatcher.java:59)

     at com.sun.javafx.event.BasicEventDispatcher.dispatchEvent(BasicEventDispatcher.java:58)

     at com.sun.javafx.event.EventDispatchChainImpl.dispatchEvent(EventDispatchChainImpl.java:114)

     at com.sun.javafx.event.BasicEventDispatcher.dispatchEvent(BasicEventDispatcher.java:56)

     at com.sun.javafx.event.EventDispatchChainImpl.dispatchEvent(EventDispatchChainImpl.java:114)

     at com.sun.javafx.event.BasicEventDispatcher.dispatchEvent(BasicEventDispatcher.java:56)

     at com.sun.javafx.event.EventDispatchChainImpl.dispatchEvent(EventDispatchChainImpl.java:114)

     at com.sun.javafx.event.EventUtil.fireEventImpl(EventUtil.java:74)

     at com.sun.javafx.event.EventUtil.fireEvent(EventUtil.java:54)

     at javafx.event.Event.fireEvent(Event.java:198)

     at javafx.scene.Scene$MouseHandler.process(Scene.java:3856)

     at javafx.scene.Scene.processMouseEvent(Scene.java:1851)

     at javafx.scene.Scene$ScenePeerListener.mouseEvent(Scene.java:2584)

     at com.sun.javafx.tk.quantum.GlassViewEventHandler$MouseEventNotification.run(GlassViewEventHandler.java:409)

     at com.sun.javafx.tk.quantum.GlassViewEventHandler$MouseEventNotification.run(GlassViewEventHandler.java:299)

     at java.base/java.security.AccessController.doPrivileged(Native Method)

     at com.sun.javafx.tk.quantum.GlassViewEventHandler.lambda$handleMouseEvent$2(GlassViewEventHandler.java:447)

     at com.sun.javafx.tk.quantum.QuantumToolkit.runWithoutRenderLock(QuantumToolkit.java:412)

     at com.sun.javafx.tk.quantum.GlassViewEventHandler.handleMouseEvent(GlassViewEventHandler.java:446)

     at com.sun.glass.ui.View.handleMouseEvent(View.java:556)

     at com.sun.glass.ui.View.notifyMouse(View.java:942)

     at com.sun.glass.ui.win.WinApplication._runLoop(Native Method)

     at com.sun.glass.ui.win.WinApplication.lambda$runLoop$3(WinApplication.java:174)

     at java.base/java.lang.Thread.run(Thread.java:829)

 Caused by: java.lang.OutOfMemoryError

     at java.base/jdk.internal.reflect.NativeConstructorAccessorImpl.newInstance0(Native Method)

     at java.base/jdk.internal.reflect.NativeConstructorAccessorImpl.newInstance(NativeConstructorAccessorImpl.java:62)

     at java.base/jdk.internal.reflect.DelegatingConstructorAccessorImpl.newInstance(DelegatingConstructorAccessorImpl.java:45)

     at java.base/java.lang.reflect.Constructor.newInstance(Constructor.java:490)

     at java.base/java.util.concurrent.ForkJoinTask.getThrowableException(ForkJoinTask.java:603)

     ... 49 more

 Caused by: java.lang.OutOfMemoryError: Java heap space

 java.util.concurrent.ExecutionException: java.lang.OutOfMemoryError

     at java.base/java.util.concurrent.ForkJoinTask.get(ForkJoinTask.java:1006)

     at fr.univ_nantes.codex_processor.gui.DatasetExtendedDepthOfFieldProcessor.run(DatasetExtendedDepthOfFieldProcessor.java:69)

     at fr.univ_nantes.codex_processor.gui.MainController$1.handle(MainController.java:96)

     at fr.univ_nantes.codex_processor.gui.MainController$1.handle(MainController.java:64)

     at com.sun.javafx.event.CompositeEventHandler.dispatchBubblingEvent(CompositeEventHandler.java:86)

     at com.sun.javafx.event.EventHandlerManager.dispatchBubblingEvent(EventHandlerManager.java:234)

     at com.sun.javafx.event.EventHandlerManager.dispatchBubblingEvent(EventHandlerManager.java:191)

     at com.sun.javafx.event.CompositeEventDispatcher.dispatchBubblingEvent(CompositeEventDispatcher.java:59)

     at com.sun.javafx.event.BasicEventDispatcher.dispatchEvent(BasicEventDispatcher.java:58)

     at com.sun.javafx.event.EventDispatchChainImpl.dispatchEvent(EventDispatchChainImpl.java:114)

     at com.sun.javafx.event.BasicEventDispatcher.dispatchEvent(BasicEventDispatcher.java:56)

     at com.sun.javafx.event.EventDispatchChainImpl.dispatchEvent(EventDispatchChainImpl.java:114)

     at com.sun.javafx.event.BasicEventDispatcher.dispatchEvent(BasicEventDispatcher.java:56)

     at com.sun.javafx.event.EventDispatchChainImpl.dispatchEvent(EventDispatchChainImpl.java:114)

     at com.sun.javafx.event.EventUtil.fireEventImpl(EventUtil.java:74)

     at com.sun.javafx.event.EventUtil.fireEvent(EventUtil.java:49)

     at javafx.event.Event.fireEvent(Event.java:198)

     at javafx.scene.Node.fireEvent(Node.java:8889)

     at javafx.scene.control.Button.fire(Button.java:203)

     at com.sun.javafx.scene.control.behavior.ButtonBehavior.mouseReleased(ButtonBehavior.java:208)

     at com.sun.javafx.scene.control.inputmap.InputMap.handle(InputMap.java:274)

     at com.sun.javafx.event.CompositeEventHandler$NormalEventHandlerRecord.handleBubblingEvent(CompositeEventHandler.java:247)

     at com.sun.javafx.event.CompositeEventHandler.dispatchBubblingEvent(CompositeEventHandler.java:80)

     at com.sun.javafx.event.EventHandlerManager.dispatchBubblingEvent(EventHandlerManager.java:234)

     at com.sun.javafx.event.EventHandlerManager.dispatchBubblingEvent(EventHandlerManager.java:191)

     at com.sun.javafx.event.CompositeEventDispatcher.dispatchBubblingEvent(CompositeEventDispatcher.java:59)

     at com.sun.javafx.event.BasicEventDispatcher.dispatchEvent(BasicEventDispatcher.java:58)

     at com.sun.javafx.event.EventDispatchChainImpl.dispatchEvent(EventDispatchChainImpl.java:114)

     at com.sun.javafx.event.BasicEventDispatcher.dispatchEvent(BasicEventDispatcher.java:56)

     at com.sun.javafx.event.EventDispatchChainImpl.dispatchEvent(EventDispatchChainImpl.java:114)

     at com.sun.javafx.event.BasicEventDispatcher.dispatchEvent(BasicEventDispatcher.java:56)

     at com.sun.javafx.event.EventDispatchChainImpl.dispatchEvent(EventDispatchChainImpl.java:114)

     at com.sun.javafx.event.EventUtil.fireEventImpl(EventUtil.java:74)

     at com.sun.javafx.event.EventUtil.fireEvent(EventUtil.java:54)

     at javafx.event.Event.fireEvent(Event.java:198)

     at javafx.scene.Scene$MouseHandler.process(Scene.java:3856)

     at javafx.scene.Scene.processMouseEvent(Scene.java:1851)

     at javafx.scene.Scene$ScenePeerListener.mouseEvent(Scene.java:2584)

     at com.sun.javafx.tk.quantum.GlassViewEventHandler$MouseEventNotification.run(GlassViewEventHandler.java:409)

     at com.sun.javafx.tk.quantum.GlassViewEventHandler$MouseEventNotification.run(GlassViewEventHandler.java:299)

     at java.base/java.security.AccessController.doPrivileged(Native Method)

     at com.sun.javafx.tk.quantum.GlassViewEventHandler.lambda$handleMouseEvent$2(GlassViewEventHandler.java:447)

     at com.sun.javafx.tk.quantum.QuantumToolkit.runWithoutRenderLock(QuantumToolkit.java:412)

     at com.sun.javafx.tk.quantum.GlassViewEventHandler.handleMouseEvent(GlassViewEventHandler.java:446)

     at com.sun.glass.ui.View.handleMouseEvent(View.java:556)

     at com.sun.glass.ui.View.notifyMouse(View.java:942)

     at com.sun.glass.ui.win.WinApplication._runLoop(Native Method)

     at com.sun.glass.ui.win.WinApplication.lambda$runLoop$3(WinApplication.java:174)

     at java.base/java.lang.Thread.run(Thread.java:829)

 Caused by: java.lang.OutOfMemoryError

     at java.base/jdk.internal.reflect.NativeConstructorAccessorImpl.newInstance0(Native Method)

     at java.base/jdk.internal.reflect.NativeConstructorAccessorImpl.newInstance(NativeConstructorAccessorImpl.java:62)

     at java.base/jdk.internal.reflect.DelegatingConstructorAccessorImpl.newInstance(DelegatingConstructorAccessorImpl.java:45)

     at java.base/java.lang.reflect.Constructor.newInstance(Constructor.java:490)

     at java.base/java.util.concurrent.ForkJoinTask.getThrowableException(ForkJoinTask.java:603)

     ... 49 more

 Caused by: java.lang.OutOfMemoryError: Java heap space

 java.util.concurrent.ExecutionException: java.lang.OutOfMemoryError

     at java.base/java.util.concurrent.ForkJoinTask.get(ForkJoinTask.java:1006)

     at fr.univ_nantes.codex_processor.gui.DatasetExtendedDepthOfFieldProcessor.run(DatasetExtendedDepthOfFieldProcessor.java:69)

     at fr.univ_nantes.codex_processor.gui.MainController$1.handle(MainController.java:96)

     at fr.univ_nantes.codex_processor.gui.MainController$1.handle(MainController.java:64)

     at com.sun.javafx.event.CompositeEventHandler.dispatchBubblingEvent(CompositeEventHandler.java:86)

     at com.sun.javafx.event.EventHandlerManager.dispatchBubblingEvent(EventHandlerManager.java:234)

     at com.sun.javafx.event.EventHandlerManager.dispatchBubblingEvent(EventHandlerManager.java:191)

     at com.sun.javafx.event.CompositeEventDispatcher.dispatchBubblingEvent(CompositeEventDispatcher.java:59)

     at com.sun.javafx.event.BasicEventDispatcher.dispatchEvent(BasicEventDispatcher.java:58)

     at com.sun.javafx.event.EventDispatchChainImpl.dispatchEvent(EventDispatchChainImpl.java:114)

     at com.sun.javafx.event.BasicEventDispatcher.dispatchEvent(BasicEventDispatcher.java:56)

     at com.sun.javafx.event.EventDispatchChainImpl.dispatchEvent(EventDispatchChainImpl.java:114)

     at com.sun.javafx.event.BasicEventDispatcher.dispatchEvent(BasicEventDispatcher.java:56)

     at com.sun.javafx.event.EventDispatchChainImpl.dispatchEvent(EventDispatchChainImpl.java:114)

     at com.sun.javafx.event.EventUtil.fireEventImpl(EventUtil.java:74)

     at com.sun.javafx.event.EventUtil.fireEvent(EventUtil.java:49)

     at javafx.event.Event.fireEvent(Event.java:198)

     at javafx.scene.Node.fireEvent(Node.java:8889)

     at javafx.scene.control.Button.fire(Button.java:203)

     at com.sun.javafx.scene.control.behavior.ButtonBehavior.mouseReleased(ButtonBehavior.java:208)

     at com.sun.javafx.scene.control.inputmap.InputMap.handle(InputMap.java:274)

     at com.sun.javafx.event.CompositeEventHandler$NormalEventHandlerRecord.handleBubblingEvent(CompositeEventHandler.java:247)

     at com.sun.javafx.event.CompositeEventHandler.dispatchBubblingEvent(CompositeEventHandler.java:80)

     at com.sun.javafx.event.EventHandlerManager.dispatchBubblingEvent(EventHandlerManager.java:234)

     at com.sun.javafx.event.EventHandlerManager.dispatchBubblingEvent(EventHandlerManager.java:191)

     at com.sun.javafx.event.CompositeEventDispatcher.dispatchBubblingEvent(CompositeEventDispatcher.java:59)

     at com.sun.javafx.event.BasicEventDispatcher.dispatchEvent(BasicEventDispatcher.java:58)

     at com.sun.javafx.event.EventDispatchChainImpl.dispatchEvent(EventDispatchChainImpl.java:114)

     at com.sun.javafx.event.BasicEventDispatcher.dispatchEvent(BasicEventDispatcher.java:56)

     at com.sun.javafx.event.EventDispatchChainImpl.dispatchEvent(EventDispatchChainImpl.java:114)

     at com.sun.javafx.event.BasicEventDispatcher.dispatchEvent(BasicEventDispatcher.java:56)

     at com.sun.javafx.event.EventDispatchChainImpl.dispatchEvent(EventDispatchChainImpl.java:114)

     at com.sun.javafx.event.EventUtil.fireEventImpl(EventUtil.java:74)

     at com.sun.javafx.event.EventUtil.fireEvent(EventUtil.java:54)

     at javafx.event.Event.fireEvent(Event.java:198)

     at javafx.scene.Scene$MouseHandler.process(Scene.java:3856)

     at javafx.scene.Scene.processMouseEvent(Scene.java:1851)

     at javafx.scene.Scene$ScenePeerListener.mouseEvent(Scene.java:2584)

     at com.sun.javafx.tk.quantum.GlassViewEventHandler$MouseEventNotification.run(GlassViewEventHandler.java:409)

     at com.sun.javafx.tk.quantum.GlassViewEventHandler$MouseEventNotification.run(GlassViewEventHandler.java:299)

     at java.base/java.security.AccessController.doPrivileged(Native Method)

     at com.sun.javafx.tk.quantum.GlassViewEventHandler.lambda$handleMouseEvent$2(GlassViewEventHandler.java:447)

     at com.sun.javafx.tk.quantum.QuantumToolkit.runWithoutRenderLock(QuantumToolkit.java:412)

     at com.sun.javafx.tk.quantum.GlassViewEventHandler.handleMouseEvent(GlassViewEventHandler.java:446)

     at com.sun.glass.ui.View.handleMouseEvent(View.java:556)

     at com.sun.glass.ui.View.notifyMouse(View.java:942)

     at com.sun.glass.ui.win.WinApplication._runLoop(Native Method)

     at com.sun.glass.ui.win.WinApplication.lambda$runLoop$3(WinApplication.java:174)

     at java.base/java.lang.Thread.run(Thread.java:829)

 Caused by: java.lang.OutOfMemoryError

     at java.base/jdk.internal.reflect.NativeConstructorAccessorImpl.newInstance0(Native Method)

     at java.base/jdk.internal.reflect.NativeConstructorAccessorImpl.newInstance(NativeConstructorAccessorImpl.java:62)

     at java.base/jdk.internal.reflect.DelegatingConstructorAccessorImpl.newInstance(DelegatingConstructorAccessorImpl.java:45)

     at java.base/java.lang.reflect.Constructor.newInstance(Constructor.java:490)

     at java.base/java.util.concurrent.ForkJoinTask.getThrowableException(ForkJoinTask.java:603)

     ... 49 more

 Caused by: java.lang.OutOfMemoryError: Java heap space

 java.util.concurrent.ExecutionException: java.lang.OutOfMemoryError

     at java.base/java.util.concurrent.ForkJoinTask.get(ForkJoinTask.java:1006)

     at fr.univ_nantes.codex_processor.gui.DatasetExtendedDepthOfFieldProcessor.run(DatasetExtendedDepthOfFieldProcessor.java:69)

     at fr.univ_nantes.codex_processor.gui.MainController$1.handle(MainController.java:96)

     at fr.univ_nantes.codex_processor.gui.MainController$1.handle(MainController.java:64)

     at com.sun.javafx.event.CompositeEventHandler.dispatchBubblingEvent(CompositeEventHandler.java:86)

     at com.sun.javafx.event.EventHandlerManager.dispatchBubblingEvent(EventHandlerManager.java:234)

     at com.sun.javafx.event.EventHandlerManager.dispatchBubblingEvent(EventHandlerManager.java:191)

     at com.sun.javafx.event.CompositeEventDispatcher.dispatchBubblingEvent(CompositeEventDispatcher.java:59)

     at com.sun.javafx.event.BasicEventDispatcher.dispatchEvent(BasicEventDispatcher.java:58)

     at com.sun.javafx.event.EventDispatchChainImpl.dispatchEvent(EventDispatchChainImpl.java:114)

     at com.sun.javafx.event.BasicEventDispatcher.dispatchEvent(BasicEventDispatcher.java:56)

     at com.sun.javafx.event.EventDispatchChainImpl.dispatchEvent(EventDispatchChainImpl.java:114)

     at com.sun.javafx.event.BasicEventDispatcher.dispatchEvent(BasicEventDispatcher.java:56)

     at com.sun.javafx.event.EventDispatchChainImpl.dispatchEvent(EventDispatchChainImpl.java:114)

     at com.sun.javafx.event.EventUtil.fireEventImpl(EventUtil.java:74)

     at com.sun.javafx.event.EventUtil.fireEvent(EventUtil.java:49)

     at javafx.event.Event.fireEvent(Event.java:198)

     at javafx.scene.Node.fireEvent(Node.java:8889)

     at javafx.scene.control.Button.fire(Button.java:203)

     at com.sun.javafx.scene.control.behavior.ButtonBehavior.mouseReleased(ButtonBehavior.java:208)

     at com.sun.javafx.scene.control.inputmap.InputMap.handle(InputMap.java:274)

     at com.sun.javafx.event.CompositeEventHandler$NormalEventHandlerRecord.handleBubblingEvent(CompositeEventHandler.java:247)

     at com.sun.javafx.event.CompositeEventHandler.dispatchBubblingEvent(CompositeEventHandler.java:80)

     at com.sun.javafx.event.EventHandlerManager.dispatchBubblingEvent(EventHandlerManager.java:234)

     at com.sun.javafx.event.EventHandlerManager.dispatchBubblingEvent(EventHandlerManager.java:191)

     at com.sun.javafx.event.CompositeEventDispatcher.dispatchBubblingEvent(CompositeEventDispatcher.java:59)

     at com.sun.javafx.event.BasicEventDispatcher.dispatchEvent(BasicEventDispatcher.java:58)

     at com.sun.javafx.event.EventDispatchChainImpl.dispatchEvent(EventDispatchChainImpl.java:114)

     at com.sun.javafx.event.BasicEventDispatcher.dispatchEvent(BasicEventDispatcher.java:56)

     at com.sun.javafx.event.EventDispatchChainImpl.dispatchEvent(EventDispatchChainImpl.java:114)

     at com.sun.javafx.event.BasicEventDispatcher.dispatchEvent(BasicEventDispatcher.java:56)

     at com.sun.javafx.event.EventDispatchChainImpl.dispatchEvent(EventDispatchChainImpl.java:114)

     at com.sun.javafx.event.EventUtil.fireEventImpl(EventUtil.java:74)

     at com.sun.javafx.event.EventUtil.fireEvent(EventUtil.java:54)

     at javafx.event.Event.fireEvent(Event.java:198)

     at javafx.scene.Scene$MouseHandler.process(Scene.java:3856)

     at javafx.scene.Scene.processMouseEvent(Scene.java:1851)

     at javafx.scene.Scene$ScenePeerListener.mouseEvent(Scene.java:2584)

     at com.sun.javafx.tk.quantum.GlassViewEventHandler$MouseEventNotification.run(GlassViewEventHandler.java:409)

     at com.sun.javafx.tk.quantum.GlassViewEventHandler$MouseEventNotification.run(GlassViewEventHandler.java:299)

     at java.base/java.security.AccessController.doPrivileged(Native Method)

     at com.sun.javafx.tk.quantum.GlassViewEventHandler.lambda$handleMouseEvent$2(GlassViewEventHandler.java:447)

     at com.sun.javafx.tk.quantum.QuantumToolkit.runWithoutRenderLock(QuantumToolkit.java:412)

     at com.sun.javafx.tk.quantum.GlassViewEventHandler.handleMouseEvent(GlassViewEventHandler.java:446)

     at com.sun.glass.ui.View.handleMouseEvent(View.java:556)

     at com.sun.glass.ui.View.notifyMouse(View.java:942)

     at com.sun.glass.ui.win.WinApplication._runLoop(Native Method)

     at com.sun.glass.ui.win.WinApplication.lambda$runLoop$3(WinApplication.java:174)

     at java.base/java.lang.Thread.run(Thread.java:829)

 Caused by: java.lang.OutOfMemoryError

     at java.base/jdk.internal.reflect.NativeConstructorAccessorImpl.newInstance0(Native Method)

     at java.base/jdk.internal.reflect.NativeConstructorAccessorImpl.newInstance(NativeConstructorAccessorImpl.java:62)

     at java.base/jdk.internal.reflect.DelegatingConstructorAccessorImpl.newInstance(DelegatingConstructorAccessorImpl.java:45)

     at java.base/java.lang.reflect.Constructor.newInstance(Constructor.java:490)

     at java.base/java.util.concurrent.ForkJoinTask.getThrowableException(ForkJoinTask.java:603)

     ... 49 more

 Caused by: java.lang.OutOfMemoryError: Java heap space

 java.util.concurrent.ExecutionException: java.lang.OutOfMemoryError

     at java.base/java.util.concurrent.ForkJoinTask.get(ForkJoinTask.java:1006)

     at fr.univ_nantes.codex_processor.gui.DatasetExtendedDepthOfFieldProcessor.run(DatasetExtendedDepthOfFieldProcessor.java:69)

     at fr.univ_nantes.codex_processor.gui.MainController$1.handle(MainController.java:96)

     at fr.univ_nantes.codex_processor.gui.MainController$1.handle(MainController.java:64)

     at com.sun.javafx.event.CompositeEventHandler.dispatchBubblingEvent(CompositeEventHandler.java:86)

     at com.sun.javafx.event.EventHandlerManager.dispatchBubblingEvent(EventHandlerManager.java:234)

     at com.sun.javafx.event.EventHandlerManager.dispatchBubblingEvent(EventHandlerManager.java:191)

     at com.sun.javafx.event.CompositeEventDispatcher.dispatchBubblingEvent(CompositeEventDispatcher.java:59)

     at com.sun.javafx.event.BasicEventDispatcher.dispatchEvent(BasicEventDispatcher.java:58)

     at com.sun.javafx.event.EventDispatchChainImpl.dispatchEvent(EventDispatchChainImpl.java:114)

     at com.sun.javafx.event.BasicEventDispatcher.dispatchEvent(BasicEventDispatcher.java:56)

     at com.sun.javafx.event.EventDispatchChainImpl.dispatchEvent(EventDispatchChainImpl.java:114)

     at com.sun.javafx.event.BasicEventDispatcher.dispatchEvent(BasicEventDispatcher.java:56)

     at com.sun.javafx.event.EventDispatchChainImpl.dispatchEvent(EventDispatchChainImpl.java:114)

     at com.sun.javafx.event.EventUtil.fireEventImpl(EventUtil.java:74)

     at com.sun.javafx.event.EventUtil.fireEvent(EventUtil.java:49)

     at javafx.event.Event.fireEvent(Event.java:198)

     at javafx.scene.Node.fireEvent(Node.java:8889)

     at javafx.scene.control.Button.fire(Button.java:203)

     at com.sun.javafx.scene.control.behavior.ButtonBehavior.mouseReleased(ButtonBehavior.java:208)

     at com.sun.javafx.scene.control.inputmap.InputMap.handle(InputMap.java:274)

     at com.sun.javafx.event.CompositeEventHandler$NormalEventHandlerRecord.handleBubblingEvent(CompositeEventHandler.java:247)

     at com.sun.javafx.event.CompositeEventHandler.dispatchBubblingEvent(CompositeEventHandler.java:80)

     at com.sun.javafx.event.EventHandlerManager.dispatchBubblingEvent(EventHandlerManager.java:234)

     at com.sun.javafx.event.EventHandlerManager.dispatchBubblingEvent(EventHandlerManager.java:191)

     at com.sun.javafx.event.CompositeEventDispatcher.dispatchBubblingEvent(CompositeEventDispatcher.java:59)

     at com.sun.javafx.event.BasicEventDispatcher.dispatchEvent(BasicEventDispatcher.java:58)

     at com.sun.javafx.event.EventDispatchChainImpl.dispatchEvent(EventDispatchChainImpl.java:114)

     at com.sun.javafx.event.BasicEventDispatcher.dispatchEvent(BasicEventDispatcher.java:56)

     at com.sun.javafx.event.EventDispatchChainImpl.dispatchEvent(EventDispatchChainImpl.java:114)

     at com.sun.javafx.event.BasicEventDispatcher.dispatchEvent(BasicEventDispatcher.java:56)

     at com.sun.javafx.event.EventDispatchChainImpl.dispatchEvent(EventDispatchChainImpl.java:114)

     at com.sun.javafx.event.EventUtil.fireEventImpl(EventUtil.java:74)

     at com.sun.javafx.event.EventUtil.fireEvent(EventUtil.java:54)

     at javafx.event.Event.fireEvent(Event.java:198)

     at javafx.scene.Scene$MouseHandler.process(Scene.java:3856)

     at javafx.scene.Scene.processMouseEvent(Scene.java:1851)

     at javafx.scene.Scene$ScenePeerListener.mouseEvent(Scene.java:2584)

     at com.sun.javafx.tk.quantum.GlassViewEventHandler$MouseEventNotification.run(GlassViewEventHandler.java:409)

     at com.sun.javafx.tk.quantum.GlassViewEventHandler$MouseEventNotification.run(GlassViewEventHandler.java:299)

     at java.base/java.security.AccessController.doPrivileged(Native Method)

     at com.sun.javafx.tk.quantum.GlassViewEventHandler.lambda$handleMouseEvent$2(GlassViewEventHandler.java:447)

     at com.sun.javafx.tk.quantum.QuantumToolkit.runWithoutRenderLock(QuantumToolkit.java:412)

     at com.sun.javafx.tk.quantum.GlassViewEventHandler.handleMouseEvent(GlassViewEventHandler.java:446)

     at com.sun.glass.ui.View.handleMouseEvent(View.java:556)

     at com.sun.glass.ui.View.notifyMouse(View.java:942)

     at com.sun.glass.ui.win.WinApplication._runLoop(Native Method)

     at com.sun.glass.ui.win.WinApplication.lambda$runLoop$3(WinApplication.java:174)

     at java.base/java.lang.Thread.run(Thread.java:829)

 Caused by: java.lang.OutOfMemoryError

     at java.base/jdk.internal.reflect.NativeConstructorAccessorImpl.newInstance0(Native Method)

     at java.base/jdk.internal.reflect.NativeConstructorAccessorImpl.newInstance(NativeConstructorAccessorImpl.java:62)

     at java.base/jdk.internal.reflect.DelegatingConstructorAccessorImpl.newInstance(DelegatingConstructorAccessorImpl.java:45)

     at java.base/java.lang.reflect.Constructor.newInstance(Constructor.java:490)

     at java.base/java.util.concurrent.ForkJoinTask.getThrowableException(ForkJoinTask.java:603)

     ... 49 more

 Caused by: java.lang.OutOfMemoryError: Java heap space

 java.util.concurrent.ExecutionException: java.lang.OutOfMemoryError

     at java.base/java.util.concurrent.ForkJoinTask.get(ForkJoinTask.java:1006)

     at fr.univ_nantes.codex_processor.gui.DatasetExtendedDepthOfFieldProcessor.run(DatasetExtendedDepthOfFieldProcessor.java:69)

     at fr.univ_nantes.codex_processor.gui.MainController$1.handle(MainController.java:96)

     at fr.univ_nantes.codex_processor.gui.MainController$1.handle(MainController.java:64)

     at com.sun.javafx.event.CompositeEventHandler.dispatchBubblingEvent(CompositeEventHandler.java:86)

     at com.sun.javafx.event.EventHandlerManager.dispatchBubblingEvent(EventHandlerManager.java:234)

     at com.sun.javafx.event.EventHandlerManager.dispatchBubblingEvent(EventHandlerManager.java:191)

     at com.sun.javafx.event.CompositeEventDispatcher.dispatchBubblingEvent(CompositeEventDispatcher.java:59)

     at com.sun.javafx.event.BasicEventDispatcher.dispatchEvent(BasicEventDispatcher.java:58)

     at com.sun.javafx.event.EventDispatchChainImpl.dispatchEvent(EventDispatchChainImpl.java:114)

     at com.sun.javafx.event.BasicEventDispatcher.dispatchEvent(BasicEventDispatcher.java:56)

     at com.sun.javafx.event.EventDispatchChainImpl.dispatchEvent(EventDispatchChainImpl.java:114)

     at com.sun.javafx.event.BasicEventDispatcher.dispatchEvent(BasicEventDispatcher.java:56)

     at com.sun.javafx.event.EventDispatchChainImpl.dispatchEvent(EventDispatchChainImpl.java:114)

     at com.sun.javafx.event.EventUtil.fireEventImpl(EventUtil.java:74)

     at com.sun.javafx.event.EventUtil.fireEvent(EventUtil.java:49)

     at javafx.event.Event.fireEvent(Event.java:198)

     at javafx.scene.Node.fireEvent(Node.java:8889)

     at javafx.scene.control.Button.fire(Button.java:203)

     at com.sun.javafx.scene.control.behavior.ButtonBehavior.mouseReleased(ButtonBehavior.java:208)

     at com.sun.javafx.scene.control.inputmap.InputMap.handle(InputMap.java:274)

     at com.sun.javafx.event.CompositeEventHandler$NormalEventHandlerRecord.handleBubblingEvent(CompositeEventHandler.java:247)

     at com.sun.javafx.event.CompositeEventHandler.dispatchBubblingEvent(CompositeEventHandler.java:80)

     at com.sun.javafx.event.EventHandlerManager.dispatchBubblingEvent(EventHandlerManager.java:234)

     at com.sun.javafx.event.EventHandlerManager.dispatchBubblingEvent(EventHandlerManager.java:191)

     at com.sun.javafx.event.CompositeEventDispatcher.dispatchBubblingEvent(CompositeEventDispatcher.java:59)

     at com.sun.javafx.event.BasicEventDispatcher.dispatchEvent(BasicEventDispatcher.java:58)

     at com.sun.javafx.event.EventDispatchChainImpl.dispatchEvent(EventDispatchChainImpl.java:114)

     at com.sun.javafx.event.BasicEventDispatcher.dispatchEvent(BasicEventDispatcher.java:56)

     at com.sun.javafx.event.EventDispatchChainImpl.dispatchEvent(EventDispatchChainImpl.java:114)

     at com.sun.javafx.event.BasicEventDispatcher.dispatchEvent(BasicEventDispatcher.java:56)

     at com.sun.javafx.event.EventDispatchChainImpl.dispatchEvent(EventDispatchChainImpl.java:114)

     at com.sun.javafx.event.EventUtil.fireEventImpl(EventUtil.java:74)

     at com.sun.javafx.event.EventUtil.fireEvent(EventUtil.java:54)

     at javafx.event.Event.fireEvent(Event.java:198)

     at javafx.scene.Scene$MouseHandler.process(Scene.java:3856)

     at javafx.scene.Scene.processMouseEvent(Scene.java:1851)

     at javafx.scene.Scene$ScenePeerListener.mouseEvent(Scene.java:2584)

     at com.sun.javafx.tk.quantum.GlassViewEventHandler$MouseEventNotification.run(GlassViewEventHandler.java:409)

     at com.sun.javafx.tk.quantum.GlassViewEventHandler$MouseEventNotification.run(GlassViewEventHandler.java:299)

     at java.base/java.security.AccessController.doPrivileged(Native Method)

     at com.sun.javafx.tk.quantum.GlassViewEventHandler.lambda$handleMouseEvent$2(GlassViewEventHandler.java:447)

     at com.sun.javafx.tk.quantum.QuantumToolkit.runWithoutRenderLock(QuantumToolkit.java:412)

     at com.sun.javafx.tk.quantum.GlassViewEventHandler.handleMouseEvent(GlassViewEventHandler.java:446)

     at com.sun.glass.ui.View.handleMouseEvent(View.java:556)

     at com.sun.glass.ui.View.notifyMouse(View.java:942)

     at com.sun.glass.ui.win.WinApplication._runLoop(Native Method)

     at com.sun.glass.ui.win.WinApplication.lambda$runLoop$3(WinApplication.java:174)

     at java.base/java.lang.Thread.run(Thread.java:829)

 Caused by: java.lang.OutOfMemoryError

     at java.base/jdk.internal.reflect.NativeConstructorAccessorImpl.newInstance0(Native Method)

     at java.base/jdk.internal.reflect.NativeConstructorAccessorImpl.newInstance(NativeConstructorAccessorImpl.java:62)

     at java.base/jdk.internal.reflect.DelegatingConstructorAccessorImpl.newInstance(DelegatingConstructorAccessorImpl.java:45)

     at java.base/java.lang.reflect.Constructor.newInstance(Constructor.java:490)

     at java.base/java.util.concurrent.ForkJoinTask.getThrowableException(ForkJoinTask.java:603)

     ... 49 more

 Caused by: java.lang.OutOfMemoryError: Java heap space

 java.util.concurrent.ExecutionException: java.lang.OutOfMemoryError

     at java.base/java.util.concurrent.ForkJoinTask.get(ForkJoinTask.java:1006)

     at fr.univ_nantes.codex_processor.gui.DatasetExtendedDepthOfFieldProcessor.run(DatasetExtendedDepthOfFieldProcessor.java:69)

     at fr.univ_nantes.codex_processor.gui.MainController$1.handle(MainController.java:96)

     at fr.univ_nantes.codex_processor.gui.MainController$1.handle(MainController.java:64)

     at com.sun.javafx.event.CompositeEventHandler.dispatchBubblingEvent(CompositeEventHandler.java:86)

     at com.sun.javafx.event.EventHandlerManager.dispatchBubblingEvent(EventHandlerManager.java:234)

     at com.sun.javafx.event.EventHandlerManager.dispatchBubblingEvent(EventHandlerManager.java:191)

     at com.sun.javafx.event.CompositeEventDispatcher.dispatchBubblingEvent(CompositeEventDispatcher.java:59)

     at com.sun.javafx.event.BasicEventDispatcher.dispatchEvent(BasicEventDispatcher.java:58)

     at com.sun.javafx.event.EventDispatchChainImpl.dispatchEvent(EventDispatchChainImpl.java:114)

     at com.sun.javafx.event.BasicEventDispatcher.dispatchEvent(BasicEventDispatcher.java:56)

     at com.sun.javafx.event.EventDispatchChainImpl.dispatchEvent(EventDispatchChainImpl.java:114)

     at com.sun.javafx.event.BasicEventDispatcher.dispatchEvent(BasicEventDispatcher.java:56)

     at com.sun.javafx.event.EventDispatchChainImpl.dispatchEvent(EventDispatchChainImpl.java:114)

     at com.sun.javafx.event.EventUtil.fireEventImpl(EventUtil.java:74)

     at com.sun.javafx.event.EventUtil.fireEvent(EventUtil.java:49)

     at javafx.event.Event.fireEvent(Event.java:198)

     at javafx.scene.Node.fireEvent(Node.java:8889)

     at javafx.scene.control.Button.fire(Button.java:203)

     at com.sun.javafx.scene.control.behavior.ButtonBehavior.mouseReleased(ButtonBehavior.java:208)

     at com.sun.javafx.scene.control.inputmap.InputMap.handle(InputMap.java:274)

     at com.sun.javafx.event.CompositeEventHandler$NormalEventHandlerRecord.handleBubblingEvent(CompositeEventHandler.java:247)

     at com.sun.javafx.event.CompositeEventHandler.dispatchBubblingEvent(CompositeEventHandler.java:80)

     at com.sun.javafx.event.EventHandlerManager.dispatchBubblingEvent(EventHandlerManager.java:234)

     at com.sun.javafx.event.EventHandlerManager.dispatchBubblingEvent(EventHandlerManager.java:191)

     at com.sun.javafx.event.CompositeEventDispatcher.dispatchBubblingEvent(CompositeEventDispatcher.java:59)

     at com.sun.javafx.event.BasicEventDispatcher.dispatchEvent(BasicEventDispatcher.java:58)

     at com.sun.javafx.event.EventDispatchChainImpl.dispatchEvent(EventDispatchChainImpl.java:114)

     at com.sun.javafx.event.BasicEventDispatcher.dispatchEvent(BasicEventDispatcher.java:56)

     at com.sun.javafx.event.EventDispatchChainImpl.dispatchEvent(EventDispatchChainImpl.java:114)

     at com.sun.javafx.event.BasicEventDispatcher.dispatchEvent(BasicEventDispatcher.java:56)

     at com.sun.javafx.event.EventDispatchChainImpl.dispatchEvent(EventDispatchChainImpl.java:114)

     at com.sun.javafx.event.EventUtil.fireEventImpl(EventUtil.java:74)

     at com.sun.javafx.event.EventUtil.fireEvent(EventUtil.java:54)

     at javafx.event.Event.fireEvent(Event.java:198)

     at javafx.scene.Scene$MouseHandler.process(Scene.java:3856)

     at javafx.scene.Scene.processMouseEvent(Scene.java:1851)

     at javafx.scene.Scene$ScenePeerListener.mouseEvent(Scene.java:2584)

     at com.sun.javafx.tk.quantum.GlassViewEventHandler$MouseEventNotification.run(GlassViewEventHandler.java:409)

     at com.sun.javafx.tk.quantum.GlassViewEventHandler$MouseEventNotification.run(GlassViewEventHandler.java:299)

     at java.base/java.security.AccessController.doPrivileged(Native Method)

     at com.sun.javafx.tk.quantum.GlassViewEventHandler.lambda$handleMouseEvent$2(GlassViewEventHandler.java:447)

     at com.sun.javafx.tk.quantum.QuantumToolkit.runWithoutRenderLock(QuantumToolkit.java:412)

     at com.sun.javafx.tk.quantum.GlassViewEventHandler.handleMouseEvent(GlassViewEventHandler.java:446)

     at com.sun.glass.ui.View.handleMouseEvent(View.java:556)

     at com.sun.glass.ui.View.notifyMouse(View.java:942)

     at com.sun.glass.ui.win.WinApplication._runLoop(Native Method)

     at com.sun.glass.ui.win.WinApplication.lambda$runLoop$3(WinApplication.java:174)

     at java.base/java.lang.Thread.run(Thread.java:829)

 Caused by: java.lang.OutOfMemoryError

     at java.base/jdk.internal.reflect.NativeConstructorAccessorImpl.newInstance0(Native Method)

     at java.base/jdk.internal.reflect.NativeConstructorAccessorImpl.newInstance(NativeConstructorAccessorImpl.java:62)

     at java.base/jdk.internal.reflect.DelegatingConstructorAccessorImpl.newInstance(DelegatingConstructorAccessorImpl.java:45)

     at java.base/java.lang.reflect.Constructor.newInstance(Constructor.java:490)

     at java.base/java.util.concurrent.ForkJoinTask.getThrowableException(ForkJoinTask.java:603)

     ... 49 more

 Caused by: java.lang.OutOfMemoryError: Java heap space

 java.util.concurrent.ExecutionException: java.lang.OutOfMemoryError

     at java.base/java.util.concurrent.ForkJoinTask.get(ForkJoinTask.java:1006)

     at fr.univ_nantes.codex_processor.gui.DatasetExtendedDepthOfFieldProcessor.run(DatasetExtendedDepthOfFieldProcessor.java:69)

     at fr.univ_nantes.codex_processor.gui.MainController$1.handle(MainController.java:96)

     at fr.univ_nantes.codex_processor.gui.MainController$1.handle(MainController.java:64)

     at com.sun.javafx.event.CompositeEventHandler.dispatchBubblingEvent(CompositeEventHandler.java:86)

     at com.sun.javafx.event.EventHandlerManager.dispatchBubblingEvent(EventHandlerManager.java:234)

     at com.sun.javafx.event.EventHandlerManager.dispatchBubblingEvent(EventHandlerManager.java:191)

     at com.sun.javafx.event.CompositeEventDispatcher.dispatchBubblingEvent(CompositeEventDispatcher.java:59)

     at com.sun.javafx.event.BasicEventDispatcher.dispatchEvent(BasicEventDispatcher.java:58)

     at com.sun.javafx.event.EventDispatchChainImpl.dispatchEvent(EventDispatchChainImpl.java:114)

     at com.sun.javafx.event.BasicEventDispatcher.dispatchEvent(BasicEventDispatcher.java:56)

     at com.sun.javafx.event.EventDispatchChainImpl.dispatchEvent(EventDispatchChainImpl.java:114)

     at com.sun.javafx.event.BasicEventDispatcher.dispatchEvent(BasicEventDispatcher.java:56)

     at com.sun.javafx.event.EventDispatchChainImpl.dispatchEvent(EventDispatchChainImpl.java:114)

     at com.sun.javafx.event.EventUtil.fireEventImpl(EventUtil.java:74)

     at com.sun.javafx.event.EventUtil.fireEvent(EventUtil.java:49)

     at javafx.event.Event.fireEvent(Event.java:198)

     at javafx.scene.Node.fireEvent(Node.java:8889)

     at javafx.scene.control.Button.fire(Button.java:203)

     at com.sun.javafx.scene.control.behavior.ButtonBehavior.mouseReleased(ButtonBehavior.java:208)

     at com.sun.javafx.scene.control.inputmap.InputMap.handle(InputMap.java:274)

     at com.sun.javafx.event.CompositeEventHandler$NormalEventHandlerRecord.handleBubblingEvent(CompositeEventHandler.java:247)

     at com.sun.javafx.event.CompositeEventHandler.dispatchBubblingEvent(CompositeEventHandler.java:80)

     at com.sun.javafx.event.EventHandlerManager.dispatchBubblingEvent(EventHandlerManager.java:234)

     at com.sun.javafx.event.EventHandlerManager.dispatchBubblingEvent(EventHandlerManager.java:191)

     at com.sun.javafx.event.CompositeEventDispatcher.dispatchBubblingEvent(CompositeEventDispatcher.java:59)

     at com.sun.javafx.event.BasicEventDispatcher.dispatchEvent(BasicEventDispatcher.java:58)

     at com.sun.javafx.event.EventDispatchChainImpl.dispatchEvent(EventDispatchChainImpl.java:114)

     at com.sun.javafx.event.BasicEventDispatcher.dispatchEvent(BasicEventDispatcher.java:56)

     at com.sun.javafx.event.EventDispatchChainImpl.dispatchEvent(EventDispatchChainImpl.java:114)

     at com.sun.javafx.event.BasicEventDispatcher.dispatchEvent(BasicEventDispatcher.java:56)

     at com.sun.javafx.event.EventDispatchChainImpl.dispatchEvent(EventDispatchChainImpl.java:114)

     at com.sun.javafx.event.EventUtil.fireEventImpl(EventUtil.java:74)

     at com.sun.javafx.event.EventUtil.fireEvent(EventUtil.java:54)

     at javafx.event.Event.fireEvent(Event.java:198)

     at javafx.scene.Scene$MouseHandler.process(Scene.java:3856)

     at javafx.scene.Scene.processMouseEvent(Scene.java:1851)

     at javafx.scene.Scene$ScenePeerListener.mouseEvent(Scene.java:2584)

     at com.sun.javafx.tk.quantum.GlassViewEventHandler$MouseEventNotification.run(GlassViewEventHandler.java:409)

     at com.sun.javafx.tk.quantum.GlassViewEventHandler$MouseEventNotification.run(GlassViewEventHandler.java:299)

     at java.base/java.security.AccessController.doPrivileged(Native Method)

     at com.sun.javafx.tk.quantum.GlassViewEventHandler.lambda$handleMouseEvent$2(GlassViewEventHandler.java:447)

     at com.sun.javafx.tk.quantum.QuantumToolkit.runWithoutRenderLock(QuantumToolkit.java:412)

     at com.sun.javafx.tk.quantum.GlassViewEventHandler.handleMouseEvent(GlassViewEventHandler.java:446)

     at com.sun.glass.ui.View.handleMouseEvent(View.java:556)

     at com.sun.glass.ui.View.notifyMouse(View.java:942)

     at com.sun.glass.ui.win.WinApplication._runLoop(Native Method)

     at com.sun.glass.ui.win.WinApplication.lambda$runLoop$3(WinApplication.java:174)

     at java.base/java.lang.Thread.run(Thread.java:829)

 Caused by: java.lang.OutOfMemoryError

     at java.base/jdk.internal.reflect.NativeConstructorAccessorImpl.newInstance0(Native Method)

     at java.base/jdk.internal.reflect.NativeConstructorAccessorImpl.newInstance(NativeConstructorAccessorImpl.java:62)

     at java.base/jdk.internal.reflect.DelegatingConstructorAccessorImpl.newInstance(DelegatingConstructorAccessorImpl.java:45)

     at java.base/java.lang.reflect.Constructor.newInstance(Constructor.java:490)

     at java.base/java.util.concurrent.ForkJoinTask.getThrowableException(ForkJoinTask.java:603)

     ... 49 more

 Caused by: java.lang.OutOfMemoryError: Java heap space

 java.util.concurrent.ExecutionException: java.lang.OutOfMemoryError

     at java.base/java.util.concurrent.ForkJoinTask.get(ForkJoinTask.java:1006)

     at fr.univ_nantes.codex_processor.gui.DatasetExtendedDepthOfFieldProcessor.run(DatasetExtendedDepthOfFieldProcessor.java:69)

     at fr.univ_nantes.codex_processor.gui.MainController$1.handle(MainController.java:96)

     at fr.univ_nantes.codex_processor.gui.MainController$1.handle(MainController.java:64)

     at com.sun.javafx.event.CompositeEventHandler.dispatchBubblingEvent(CompositeEventHandler.java:86)

     at com.sun.javafx.event.EventHandlerManager.dispatchBubblingEvent(EventHandlerManager.java:234)

     at com.sun.javafx.event.EventHandlerManager.dispatchBubblingEvent(EventHandlerManager.java:191)

     at com.sun.javafx.event.CompositeEventDispatcher.dispatchBubblingEvent(CompositeEventDispatcher.java:59)

     at com.sun.javafx.event.BasicEventDispatcher.dispatchEvent(BasicEventDispatcher.java:58)

     at com.sun.javafx.event.EventDispatchChainImpl.dispatchEvent(EventDispatchChainImpl.java:114)

     at com.sun.javafx.event.BasicEventDispatcher.dispatchEvent(BasicEventDispatcher.java:56)

     at com.sun.javafx.event.EventDispatchChainImpl.dispatchEvent(EventDispatchChainImpl.java:114)

     at com.sun.javafx.event.BasicEventDispatcher.dispatchEvent(BasicEventDispatcher.java:56)

     at com.sun.javafx.event.EventDispatchChainImpl.dispatchEvent(EventDispatchChainImpl.java:114)

     at com.sun.javafx.event.EventUtil.fireEventImpl(EventUtil.java:74)

     at com.sun.javafx.event.EventUtil.fireEvent(EventUtil.java:49)

     at javafx.event.Event.fireEvent(Event.java:198)

     at javafx.scene.Node.fireEvent(Node.java:8889)

     at javafx.scene.control.Button.fire(Button.java:203)

     at com.sun.javafx.scene.control.behavior.ButtonBehavior.mouseReleased(ButtonBehavior.java:208)

     at com.sun.javafx.scene.control.inputmap.InputMap.handle(InputMap.java:274)

     at com.sun.javafx.event.CompositeEventHandler$NormalEventHandlerRecord.handleBubblingEvent(CompositeEventHandler.java:247)

     at com.sun.javafx.event.CompositeEventHandler.dispatchBubblingEvent(CompositeEventHandler.java:80)

     at com.sun.javafx.event.EventHandlerManager.dispatchBubblingEvent(EventHandlerManager.java:234)

     at com.sun.javafx.event.EventHandlerManager.dispatchBubblingEvent(EventHandlerManager.java:191)

     at com.sun.javafx.event.CompositeEventDispatcher.dispatchBubblingEvent(CompositeEventDispatcher.java:59)

     at com.sun.javafx.event.BasicEventDispatcher.dispatchEvent(BasicEventDispatcher.java:58)

     at com.sun.javafx.event.EventDispatchChainImpl.dispatchEvent(EventDispatchChainImpl.java:114)

     at com.sun.javafx.event.BasicEventDispatcher.dispatchEvent(BasicEventDispatcher.java:56)

     at com.sun.javafx.event.EventDispatchChainImpl.dispatchEvent(EventDispatchChainImpl.java:114)

     at com.sun.javafx.event.BasicEventDispatcher.dispatchEvent(BasicEventDispatcher.java:56)

     at com.sun.javafx.event.EventDispatchChainImpl.dispatchEvent(EventDispatchChainImpl.java:114)

     at com.sun.javafx.event.EventUtil.fireEventImpl(EventUtil.java:74)

     at com.sun.javafx.event.EventUtil.fireEvent(EventUtil.java:54)

     at javafx.event.Event.fireEvent(Event.java:198)

     at javafx.scene.Scene$MouseHandler.process(Scene.java:3856)

     at javafx.scene.Scene.processMouseEvent(Scene.java:1851)

     at javafx.scene.Scene$ScenePeerListener.mouseEvent(Scene.java:2584)

     at com.sun.javafx.tk.quantum.GlassViewEventHandler$MouseEventNotification.run(GlassViewEventHandler.java:409)

     at com.sun.javafx.tk.quantum.GlassViewEventHandler$MouseEventNotification.run(GlassViewEventHandler.java:299)

     at java.base/java.security.AccessController.doPrivileged(Native Method)

     at com.sun.javafx.tk.quantum.GlassViewEventHandler.lambda$handleMouseEvent$2(GlassViewEventHandler.java:447)

     at com.sun.javafx.tk.quantum.QuantumToolkit.runWithoutRenderLock(QuantumToolkit.java:412)

     at com.sun.javafx.tk.quantum.GlassViewEventHandler.handleMouseEvent(GlassViewEventHandler.java:446)

     at com.sun.glass.ui.View.handleMouseEvent(View.java:556)

     at com.sun.glass.ui.View.notifyMouse(View.java:942)

     at com.sun.glass.ui.win.WinApplication._runLoop(Native Method)

     at com.sun.glass.ui.win.WinApplication.lambda$runLoop$3(WinApplication.java:174)

     at java.base/java.lang.Thread.run(Thread.java:829)

 Caused by: java.lang.OutOfMemoryError

     at java.base/jdk.internal.reflect.NativeConstructorAccessorImpl.newInstance0(Native Method)

     at java.base/jdk.internal.reflect.NativeConstructorAccessorImpl.newInstance(NativeConstructorAccessorImpl.java:62)

     at java.base/jdk.internal.reflect.DelegatingConstructorAccessorImpl.newInstance(DelegatingConstructorAccessorImpl.java:45)

     at java.base/java.lang.reflect.Constructor.newInstance(Constructor.java:490)

     at java.base/java.util.concurrent.ForkJoinTask.getThrowableException(ForkJoinTask.java:603)

     ... 49 more

 Caused by: java.lang.OutOfMemoryError: Java heap space

 java.util.concurrent.ExecutionException: java.lang.OutOfMemoryError

     at java.base/java.util.concurrent.ForkJoinTask.get(ForkJoinTask.java:1006)

     at fr.univ_nantes.codex_processor.gui.DatasetExtendedDepthOfFieldProcessor.run(DatasetExtendedDepthOfFieldProcessor.java:69)

     at fr.univ_nantes.codex_processor.gui.MainController$1.handle(MainController.java:96)

     at fr.univ_nantes.codex_processor.gui.MainController$1.handle(MainController.java:64)

     at com.sun.javafx.event.CompositeEventHandler.dispatchBubblingEvent(CompositeEventHandler.java:86)

     at com.sun.javafx.event.EventHandlerManager.dispatchBubblingEvent(EventHandlerManager.java:234)

     at com.sun.javafx.event.EventHandlerManager.dispatchBubblingEvent(EventHandlerManager.java:191)

     at com.sun.javafx.event.CompositeEventDispatcher.dispatchBubblingEvent(CompositeEventDispatcher.java:59)

     at com.sun.javafx.event.BasicEventDispatcher.dispatchEvent(BasicEventDispatcher.java:58)

     at com.sun.javafx.event.EventDispatchChainImpl.dispatchEvent(EventDispatchChainImpl.java:114)

     at com.sun.javafx.event.BasicEventDispatcher.dispatchEvent(BasicEventDispatcher.java:56)

     at com.sun.javafx.event.EventDispatchChainImpl.dispatchEvent(EventDispatchChainImpl.java:114)

     at com.sun.javafx.event.BasicEventDispatcher.dispatchEvent(BasicEventDispatcher.java:56)

     at com.sun.javafx.event.EventDispatchChainImpl.dispatchEvent(EventDispatchChainImpl.java:114)

     at com.sun.javafx.event.EventUtil.fireEventImpl(EventUtil.java:74)

     at com.sun.javafx.event.EventUtil.fireEvent(EventUtil.java:49)

     at javafx.event.Event.fireEvent(Event.java:198)

     at javafx.scene.Node.fireEvent(Node.java:8889)

     at javafx.scene.control.Button.fire(Button.java:203)

     at com.sun.javafx.scene.control.behavior.ButtonBehavior.mouseReleased(ButtonBehavior.java:208)

     at com.sun.javafx.scene.control.inputmap.InputMap.handle(InputMap.java:274)

     at com.sun.javafx.event.CompositeEventHandler$NormalEventHandlerRecord.handleBubblingEvent(CompositeEventHandler.java:247)

     at com.sun.javafx.event.CompositeEventHandler.dispatchBubblingEvent(CompositeEventHandler.java:80)

     at com.sun.javafx.event.EventHandlerManager.dispatchBubblingEvent(EventHandlerManager.java:234)

     at com.sun.javafx.event.EventHandlerManager.dispatchBubblingEvent(EventHandlerManager.java:191)

     at com.sun.javafx.event.CompositeEventDispatcher.dispatchBubblingEvent(CompositeEventDispatcher.java:59)

     at com.sun.javafx.event.BasicEventDispatcher.dispatchEvent(BasicEventDispatcher.java:58)

     at com.sun.javafx.event.EventDispatchChainImpl.dispatchEvent(EventDispatchChainImpl.java:114)

     at com.sun.javafx.event.BasicEventDispatcher.dispatchEvent(BasicEventDispatcher.java:56)

     at com.sun.javafx.event.EventDispatchChainImpl.dispatchEvent(EventDispatchChainImpl.java:114)

     at com.sun.javafx.event.BasicEventDispatcher.dispatchEvent(BasicEventDispatcher.java:56)

     at com.sun.javafx.event.EventDispatchChainImpl.dispatchEvent(EventDispatchChainImpl.java:114)

     at com.sun.javafx.event.EventUtil.fireEventImpl(EventUtil.java:74)

     at com.sun.javafx.event.EventUtil.fireEvent(EventUtil.java:54)

     at javafx.event.Event.fireEvent(Event.java:198)

     at javafx.scene.Scene$MouseHandler.process(Scene.java:3856)

     at javafx.scene.Scene.processMouseEvent(Scene.java:1851)

     at javafx.scene.Scene$ScenePeerListener.mouseEvent(Scene.java:2584)

     at com.sun.javafx.tk.quantum.GlassViewEventHandler$MouseEventNotification.run(GlassViewEventHandler.java:409)

     at com.sun.javafx.tk.quantum.GlassViewEventHandler$MouseEventNotification.run(GlassViewEventHandler.java:299)

     at java.base/java.security.AccessController.doPrivileged(Native Method)

     at com.sun.javafx.tk.quantum.GlassViewEventHandler.lambda$handleMouseEvent$2(GlassViewEventHandler.java:447)

     at com.sun.javafx.tk.quantum.QuantumToolkit.runWithoutRenderLock(QuantumToolkit.java:412)

     at com.sun.javafx.tk.quantum.GlassViewEventHandler.handleMouseEvent(GlassViewEventHandler.java:446)

     at com.sun.glass.ui.View.handleMouseEvent(View.java:556)

     at com.sun.glass.ui.View.notifyMouse(View.java:942)

     at com.sun.glass.ui.win.WinApplication._runLoop(Native Method)

     at com.sun.glass.ui.win.WinApplication.lambda$runLoop$3(WinApplication.java:174)

     at java.base/java.lang.Thread.run(Thread.java:829)

 Caused by: java.lang.OutOfMemoryError

     at java.base/jdk.internal.reflect.NativeConstructorAccessorImpl.newInstance0(Native Method)

     at java.base/jdk.internal.reflect.NativeConstructorAccessorImpl.newInstance(NativeConstructorAccessorImpl.java:62)

     at java.base/jdk.internal.reflect.DelegatingConstructorAccessorImpl.newInstance(DelegatingConstructorAccessorImpl.java:45)

     at java.base/java.lang.reflect.Constructor.newInstance(Constructor.java:490)

     at java.base/java.util.concurrent.ForkJoinTask.getThrowableException(ForkJoinTask.java:603)

     ... 49 more

 Caused by: java.lang.OutOfMemoryError: Java heap space

 java.util.concurrent.ExecutionException: java.lang.OutOfMemoryError

     at java.base/java.util.concurrent.ForkJoinTask.get(ForkJoinTask.java:1006)

     at fr.univ_nantes.codex_processor.gui.DatasetExtendedDepthOfFieldProcessor.run(DatasetExtendedDepthOfFieldProcessor.java:69)

     at fr.univ_nantes.codex_processor.gui.MainController$1.handle(MainController.java:96)

     at fr.univ_nantes.codex_processor.gui.MainController$1.handle(MainController.java:64)

     at com.sun.javafx.event.CompositeEventHandler.dispatchBubblingEvent(CompositeEventHandler.java:86)

     at com.sun.javafx.event.EventHandlerManager.dispatchBubblingEvent(EventHandlerManager.java:234)

     at com.sun.javafx.event.EventHandlerManager.dispatchBubblingEvent(EventHandlerManager.java:191)

     at com.sun.javafx.event.CompositeEventDispatcher.dispatchBubblingEvent(CompositeEventDispatcher.java:59)

     at com.sun.javafx.event.BasicEventDispatcher.dispatchEvent(BasicEventDispatcher.java:58)

     at com.sun.javafx.event.EventDispatchChainImpl.dispatchEvent(EventDispatchChainImpl.java:114)

     at com.sun.javafx.event.BasicEventDispatcher.dispatchEvent(BasicEventDispatcher.java:56)

     at com.sun.javafx.event.EventDispatchChainImpl.dispatchEvent(EventDispatchChainImpl.java:114)

     at com.sun.javafx.event.BasicEventDispatcher.dispatchEvent(BasicEventDispatcher.java:56)

     at com.sun.javafx.event.EventDispatchChainImpl.dispatchEvent(EventDispatchChainImpl.java:114)

     at com.sun.javafx.event.EventUtil.fireEventImpl(EventUtil.java:74)

     at com.sun.javafx.event.EventUtil.fireEvent(EventUtil.java:49)

     at javafx.event.Event.fireEvent(Event.java:198)

     at javafx.scene.Node.fireEvent(Node.java:8889)

     at javafx.scene.control.Button.fire(Button.java:203)

     at com.sun.javafx.scene.control.behavior.ButtonBehavior.mouseReleased(ButtonBehavior.java:208)

     at com.sun.javafx.scene.control.inputmap.InputMap.handle(InputMap.java:274)

     at com.sun.javafx.event.CompositeEventHandler$NormalEventHandlerRecord.handleBubblingEvent(CompositeEventHandler.java:247)

     at com.sun.javafx.event.CompositeEventHandler.dispatchBubblingEvent(CompositeEventHandler.java:80)

     at com.sun.javafx.event.EventHandlerManager.dispatchBubblingEvent(EventHandlerManager.java:234)

     at com.sun.javafx.event.EventHandlerManager.dispatchBubblingEvent(EventHandlerManager.java:191)

     at com.sun.javafx.event.CompositeEventDispatcher.dispatchBubblingEvent(CompositeEventDispatcher.java:59)

     at com.sun.javafx.event.BasicEventDispatcher.dispatchEvent(BasicEventDispatcher.java:58)

     at com.sun.javafx.event.EventDispatchChainImpl.dispatchEvent(EventDispatchChainImpl.java:114)

     at com.sun.javafx.event.BasicEventDispatcher.dispatchEvent(BasicEventDispatcher.java:56)

     at com.sun.javafx.event.EventDispatchChainImpl.dispatchEvent(EventDispatchChainImpl.java:114)

     at com.sun.javafx.event.BasicEventDispatcher.dispatchEvent(BasicEventDispatcher.java:56)

     at com.sun.javafx.event.EventDispatchChainImpl.dispatchEvent(EventDispatchChainImpl.java:114)

     at com.sun.javafx.event.EventUtil.fireEventImpl(EventUtil.java:74)

     at com.sun.javafx.event.EventUtil.fireEvent(EventUtil.java:54)

     at javafx.event.Event.fireEvent(Event.java:198)

     at javafx.scene.Scene$MouseHandler.process(Scene.java:3856)

     at javafx.scene.Scene.processMouseEvent(Scene.java:1851)

     at javafx.scene.Scene$ScenePeerListener.mouseEvent(Scene.java:2584)

     at com.sun.javafx.tk.quantum.GlassViewEventHandler$MouseEventNotification.run(GlassViewEventHandler.java:409)

     at com.sun.javafx.tk.quantum.GlassViewEventHandler$MouseEventNotification.run(GlassViewEventHandler.java:299)

     at java.base/java.security.AccessController.doPrivileged(Native Method)

     at com.sun.javafx.tk.quantum.GlassViewEventHandler.lambda$handleMouseEvent$2(GlassViewEventHandler.java:447)

     at com.sun.javafx.tk.quantum.QuantumToolkit.runWithoutRenderLock(QuantumToolkit.java:412)

     at com.sun.javafx.tk.quantum.GlassViewEventHandler.handleMouseEvent(GlassViewEventHandler.java:446)

     at com.sun.glass.ui.View.handleMouseEvent(View.java:556)

     at com.sun.glass.ui.View.notifyMouse(View.java:942)

     at com.sun.glass.ui.win.WinApplication._runLoop(Native Method)

     at com.sun.glass.ui.win.WinApplication.lambda$runLoop$3(WinApplication.java:174)

     at java.base/java.lang.Thread.run(Thread.java:829)

 Caused by: java.lang.OutOfMemoryError

     at java.base/jdk.internal.reflect.NativeConstructorAccessorImpl.newInstance0(Native Method)

     at java.base/jdk.internal.reflect.NativeConstructorAccessorImpl.newInstance(NativeConstructorAccessorImpl.java:62)

     at java.base/jdk.internal.reflect.DelegatingConstructorAccessorImpl.newInstance(DelegatingConstructorAccessorImpl.java:45)

     at java.base/java.lang.reflect.Constructor.newInstance(Constructor.java:490)

     at java.base/java.util.concurrent.ForkJoinTask.getThrowableException(ForkJoinTask.java:603)

     ... 49 more

 Caused by: java.lang.OutOfMemoryError: Java heap space

 java.util.concurrent.ExecutionException: java.lang.OutOfMemoryError

     at java.base/java.util.concurrent.ForkJoinTask.get(ForkJoinTask.java:1006)

     at fr.univ_nantes.codex_processor.gui.DatasetExtendedDepthOfFieldProcessor.run(DatasetExtendedDepthOfFieldProcessor.java:69)

     at fr.univ_nantes.codex_processor.gui.MainController$1.handle(MainController.java:96)

     at fr.univ_nantes.codex_processor.gui.MainController$1.handle(MainController.java:64)

     at com.sun.javafx.event.CompositeEventHandler.dispatchBubblingEvent(CompositeEventHandler.java:86)

     at com.sun.javafx.event.EventHandlerManager.dispatchBubblingEvent(EventHandlerManager.java:234)

     at com.sun.javafx.event.EventHandlerManager.dispatchBubblingEvent(EventHandlerManager.java:191)

     at com.sun.javafx.event.CompositeEventDispatcher.dispatchBubblingEvent(CompositeEventDispatcher.java:59)

     at com.sun.javafx.event.BasicEventDispatcher.dispatchEvent(BasicEventDispatcher.java:58)

     at com.sun.javafx.event.EventDispatchChainImpl.dispatchEvent(EventDispatchChainImpl.java:114)

     at com.sun.javafx.event.BasicEventDispatcher.dispatchEvent(BasicEventDispatcher.java:56)

     at com.sun.javafx.event.EventDispatchChainImpl.dispatchEvent(EventDispatchChainImpl.java:114)

     at com.sun.javafx.event.BasicEventDispatcher.dispatchEvent(BasicEventDispatcher.java:56)

     at com.sun.javafx.event.EventDispatchChainImpl.dispatchEvent(EventDispatchChainImpl.java:114)

     at com.sun.javafx.event.EventUtil.fireEventImpl(EventUtil.java:74)

     at com.sun.javafx.event.EventUtil.fireEvent(EventUtil.java:49)

     at javafx.event.Event.fireEvent(Event.java:198)

     at javafx.scene.Node.fireEvent(Node.java:8889)

     at javafx.scene.control.Button.fire(Button.java:203)

     at com.sun.javafx.scene.control.behavior.ButtonBehavior.mouseReleased(ButtonBehavior.java:208)

     at com.sun.javafx.scene.control.inputmap.InputMap.handle(InputMap.java:274)

     at com.sun.javafx.event.CompositeEventHandler$NormalEventHandlerRecord.handleBubblingEvent(CompositeEventHandler.java:247)

     at com.sun.javafx.event.CompositeEventHandler.dispatchBubblingEvent(CompositeEventHandler.java:80)

     at com.sun.javafx.event.EventHandlerManager.dispatchBubblingEvent(EventHandlerManager.java:234)

     at com.sun.javafx.event.EventHandlerManager.dispatchBubblingEvent(EventHandlerManager.java:191)

     at com.sun.javafx.event.CompositeEventDispatcher.dispatchBubblingEvent(CompositeEventDispatcher.java:59)

     at com.sun.javafx.event.BasicEventDispatcher.dispatchEvent(BasicEventDispatcher.java:58)

     at com.sun.javafx.event.EventDispatchChainImpl.dispatchEvent(EventDispatchChainImpl.java:114)

     at com.sun.javafx.event.BasicEventDispatcher.dispatchEvent(BasicEventDispatcher.java:56)

     at com.sun.javafx.event.EventDispatchChainImpl.dispatchEvent(EventDispatchChainImpl.java:114)

     at com.sun.javafx.event.BasicEventDispatcher.dispatchEvent(BasicEventDispatcher.java:56)

     at com.sun.javafx.event.EventDispatchChainImpl.dispatchEvent(EventDispatchChainImpl.java:114)

     at com.sun.javafx.event.EventUtil.fireEventImpl(EventUtil.java:74)

     at com.sun.javafx.event.EventUtil.fireEvent(EventUtil.java:54)

     at javafx.event.Event.fireEvent(Event.java:198)

     at javafx.scene.Scene$MouseHandler.process(Scene.java:3856)

     at javafx.scene.Scene.processMouseEvent(Scene.java:1851)

     at javafx.scene.Scene$ScenePeerListener.mouseEvent(Scene.java:2584)

     at com.sun.javafx.tk.quantum.GlassViewEventHandler$MouseEventNotification.run(GlassViewEventHandler.java:409)

     at com.sun.javafx.tk.quantum.GlassViewEventHandler$MouseEventNotification.run(GlassViewEventHandler.java:299)

     at java.base/java.security.AccessController.doPrivileged(Native Method)

     at com.sun.javafx.tk.quantum.GlassViewEventHandler.lambda$handleMouseEvent$2(GlassViewEventHandler.java:447)

     at com.sun.javafx.tk.quantum.QuantumToolkit.runWithoutRenderLock(QuantumToolkit.java:412)

     at com.sun.javafx.tk.quantum.GlassViewEventHandler.handleMouseEvent(GlassViewEventHandler.java:446)

     at com.sun.glass.ui.View.handleMouseEvent(View.java:556)

     at com.sun.glass.ui.View.notifyMouse(View.java:942)

     at com.sun.glass.ui.win.WinApplication._runLoop(Native Method)

     at com.sun.glass.ui.win.WinApplication.lambda$runLoop$3(WinApplication.java:174)

     at java.base/java.lang.Thread.run(Thread.java:829)

 Caused by: java.lang.OutOfMemoryError

     at java.base/jdk.internal.reflect.GeneratedConstructorAccessor3.newInstance(Unknown Source)

     at java.base/jdk.internal.reflect.DelegatingConstructorAccessorImpl.newInstance(DelegatingConstructorAccessorImpl.java:45)

     at java.base/java.lang.reflect.Constructor.newInstance(Constructor.java:490)

     at java.base/java.util.concurrent.ForkJoinTask.getThrowableException(ForkJoinTask.java:603)

     ... 49 more

 Caused by: java.lang.OutOfMemoryError: Java heap space

 java.util.concurrent.ExecutionException: java.lang.OutOfMemoryError

     at java.base/java.util.concurrent.ForkJoinTask.get(ForkJoinTask.java:1006)

     at fr.univ_nantes.codex_processor.gui.DatasetExtendedDepthOfFieldProcessor.run(DatasetExtendedDepthOfFieldProcessor.java:69)

     at fr.univ_nantes.codex_processor.gui.MainController$1.handle(MainController.java:96)

     at fr.univ_nantes.codex_processor.gui.MainController$1.handle(MainController.java:64)

     at com.sun.javafx.event.CompositeEventHandler.dispatchBubblingEvent(CompositeEventHandler.java:86)

     at com.sun.javafx.event.EventHandlerManager.dispatchBubblingEvent(EventHandlerManager.java:234)

     at com.sun.javafx.event.EventHandlerManager.dispatchBubblingEvent(EventHandlerManager.java:191)

     at com.sun.javafx.event.CompositeEventDispatcher.dispatchBubblingEvent(CompositeEventDispatcher.java:59)

     at com.sun.javafx.event.BasicEventDispatcher.dispatchEvent(BasicEventDispatcher.java:58)

     at com.sun.javafx.event.EventDispatchChainImpl.dispatchEvent(EventDispatchChainImpl.java:114)

     at com.sun.javafx.event.BasicEventDispatcher.dispatchEvent(BasicEventDispatcher.java:56)

     at com.sun.javafx.event.EventDispatchChainImpl.dispatchEvent(EventDispatchChainImpl.java:114)

     at com.sun.javafx.event.BasicEventDispatcher.dispatchEvent(BasicEventDispatcher.java:56)

     at com.sun.javafx.event.EventDispatchChainImpl.dispatchEvent(EventDispatchChainImpl.java:114)

     at com.sun.javafx.event.EventUtil.fireEventImpl(EventUtil.java:74)

     at com.sun.javafx.event.EventUtil.fireEvent(EventUtil.java:49)

     at javafx.event.Event.fireEvent(Event.java:198)

     at javafx.scene.Node.fireEvent(Node.java:8889)

     at javafx.scene.control.Button.fire(Button.java:203)

     at com.sun.javafx.scene.control.behavior.ButtonBehavior.mouseReleased(ButtonBehavior.java:208)

     at com.sun.javafx.scene.control.inputmap.InputMap.handle(InputMap.java:274)

     at com.sun.javafx.event.CompositeEventHandler$NormalEventHandlerRecord.handleBubblingEvent(CompositeEventHandler.java:247)

     at com.sun.javafx.event.CompositeEventHandler.dispatchBubblingEvent(CompositeEventHandler.java:80)

     at com.sun.javafx.event.EventHandlerManager.dispatchBubblingEvent(EventHandlerManager.java:234)

     at com.sun.javafx.event.EventHandlerManager.dispatchBubblingEvent(EventHandlerManager.java:191)

     at com.sun.javafx.event.CompositeEventDispatcher.dispatchBubblingEvent(CompositeEventDispatcher.java:59)

     at com.sun.javafx.event.BasicEventDispatcher.dispatchEvent(BasicEventDispatcher.java:58)

     at com.sun.javafx.event.EventDispatchChainImpl.dispatchEvent(EventDispatchChainImpl.java:114)

     at com.sun.javafx.event.BasicEventDispatcher.dispatchEvent(BasicEventDispatcher.java:56)

     at com.sun.javafx.event.EventDispatchChainImpl.dispatchEvent(EventDispatchChainImpl.java:114)

     at com.sun.javafx.event.BasicEventDispatcher.dispatchEvent(BasicEventDispatcher.java:56)

     at com.sun.javafx.event.EventDispatchChainImpl.dispatchEvent(EventDispatchChainImpl.java:114)

     at com.sun.javafx.event.EventUtil.fireEventImpl(EventUtil.java:74)

     at com.sun.javafx.event.EventUtil.fireEvent(EventUtil.java:54)

     at javafx.event.Event.fireEvent(Event.java:198)

     at javafx.scene.Scene$MouseHandler.process(Scene.java:3856)

     at javafx.scene.Scene.processMouseEvent(Scene.java:1851)

     at javafx.scene.Scene$ScenePeerListener.mouseEvent(Scene.java:2584)

     at com.sun.javafx.tk.quantum.GlassViewEventHandler$MouseEventNotification.run(GlassViewEventHandler.java:409)

     at com.sun.javafx.tk.quantum.GlassViewEventHandler$MouseEventNotification.run(GlassViewEventHandler.java:299)

     at java.base/java.security.AccessController.doPrivileged(Native Method)

     at com.sun.javafx.tk.quantum.GlassViewEventHandler.lambda$handleMouseEvent$2(GlassViewEventHandler.java:447)

     at com.sun.javafx.tk.quantum.QuantumToolkit.runWithoutRenderLock(QuantumToolkit.java:412)

     at com.sun.javafx.tk.quantum.GlassViewEventHandler.handleMouseEvent(GlassViewEventHandler.java:446)

     at com.sun.glass.ui.View.handleMouseEvent(View.java:556)

     at com.sun.glass.ui.View.notifyMouse(View.java:942)

     at com.sun.glass.ui.win.WinApplication._runLoop(Native Method)

     at com.sun.glass.ui.win.WinApplication.lambda$runLoop$3(WinApplication.java:174)

     at java.base/java.lang.Thread.run(Thread.java:829)

 Caused by: java.lang.OutOfMemoryError

     at java.base/jdk.internal.reflect.GeneratedConstructorAccessor3.newInstance(Unknown Source)

     at java.base/jdk.internal.reflect.DelegatingConstructorAccessorImpl.newInstance(DelegatingConstructorAccessorImpl.java:45)

     at java.base/java.lang.reflect.Constructor.newInstance(Constructor.java:490)

     at java.base/java.util.concurrent.ForkJoinTask.getThrowableException(ForkJoinTask.java:603)

     ... 49 more

 Caused by: java.lang.OutOfMemoryError: Java heap space

 java.util.concurrent.ExecutionException: java.lang.OutOfMemoryError

     at java.base/java.util.concurrent.ForkJoinTask.get(ForkJoinTask.java:1006)

     at fr.univ_nantes.codex_processor.gui.DatasetExtendedDepthOfFieldProcessor.run(DatasetExtendedDepthOfFieldProcessor.java:69)

     at fr.univ_nantes.codex_processor.gui.MainController$1.handle(MainController.java:96)

     at fr.univ_nantes.codex_processor.gui.MainController$1.handle(MainController.java:64)

     at com.sun.javafx.event.CompositeEventHandler.dispatchBubblingEvent(CompositeEventHandler.java:86)

     at com.sun.javafx.event.EventHandlerManager.dispatchBubblingEvent(EventHandlerManager.java:234)

     at com.sun.javafx.event.EventHandlerManager.dispatchBubblingEvent(EventHandlerManager.java:191)

     at com.sun.javafx.event.CompositeEventDispatcher.dispatchBubblingEvent(CompositeEventDispatcher.java:59)

     at com.sun.javafx.event.BasicEventDispatcher.dispatchEvent(BasicEventDispatcher.java:58)

     at com.sun.javafx.event.EventDispatchChainImpl.dispatchEvent(EventDispatchChainImpl.java:114)

     at com.sun.javafx.event.BasicEventDispatcher.dispatchEvent(BasicEventDispatcher.java:56)

     at com.sun.javafx.event.EventDispatchChainImpl.dispatchEvent(EventDispatchChainImpl.java:114)

     at com.sun.javafx.event.BasicEventDispatcher.dispatchEvent(BasicEventDispatcher.java:56)

     at com.sun.javafx.event.EventDispatchChainImpl.dispatchEvent(EventDispatchChainImpl.java:114)

     at com.sun.javafx.event.EventUtil.fireEventImpl(EventUtil.java:74)

     at com.sun.javafx.event.EventUtil.fireEvent(EventUtil.java:49)

     at javafx.event.Event.fireEvent(Event.java:198)

     at javafx.scene.Node.fireEvent(Node.java:8889)

     at javafx.scene.control.Button.fire(Button.java:203)

     at com.sun.javafx.scene.control.behavior.ButtonBehavior.mouseReleased(ButtonBehavior.java:208)

     at com.sun.javafx.scene.control.inputmap.InputMap.handle(InputMap.java:274)

     at com.sun.javafx.event.CompositeEventHandler$NormalEventHandlerRecord.handleBubblingEvent(CompositeEventHandler.java:247)

     at com.sun.javafx.event.CompositeEventHandler.dispatchBubblingEvent(CompositeEventHandler.java:80)

     at com.sun.javafx.event.EventHandlerManager.dispatchBubblingEvent(EventHandlerManager.java:234)

     at com.sun.javafx.event.EventHandlerManager.dispatchBubblingEvent(EventHandlerManager.java:191)

     at com.sun.javafx.event.CompositeEventDispatcher.dispatchBubblingEvent(CompositeEventDispatcher.java:59)

     at com.sun.javafx.event.BasicEventDispatcher.dispatchEvent(BasicEventDispatcher.java:58)

     at com.sun.javafx.event.EventDispatchChainImpl.dispatchEvent(EventDispatchChainImpl.java:114)

     at com.sun.javafx.event.BasicEventDispatcher.dispatchEvent(BasicEventDispatcher.java:56)

     at com.sun.javafx.event.EventDispatchChainImpl.dispatchEvent(EventDispatchChainImpl.java:114)

     at com.sun.javafx.event.BasicEventDispatcher.dispatchEvent(BasicEventDispatcher.java:56)

     at com.sun.javafx.event.EventDispatchChainImpl.dispatchEvent(EventDispatchChainImpl.java:114)

     at com.sun.javafx.event.EventUtil.fireEventImpl(EventUtil.java:74)

     at com.sun.javafx.event.EventUtil.fireEvent(EventUtil.java:54)

     at javafx.event.Event.fireEvent(Event.java:198)

     at javafx.scene.Scene$MouseHandler.process(Scene.java:3856)

     at javafx.scene.Scene.processMouseEvent(Scene.java:1851)

     at javafx.scene.Scene$ScenePeerListener.mouseEvent(Scene.java:2584)

     at com.sun.javafx.tk.quantum.GlassViewEventHandler$MouseEventNotification.run(GlassViewEventHandler.java:409)

     at com.sun.javafx.tk.quantum.GlassViewEventHandler$MouseEventNotification.run(GlassViewEventHandler.java:299)

     at java.base/java.security.AccessController.doPrivileged(Native Method)

     at com.sun.javafx.tk.quantum.GlassViewEventHandler.lambda$handleMouseEvent$2(GlassViewEventHandler.java:447)

     at com.sun.javafx.tk.quantum.QuantumToolkit.runWithoutRenderLock(QuantumToolkit.java:412)

     at com.sun.javafx.tk.quantum.GlassViewEventHandler.handleMouseEvent(GlassViewEventHandler.java:446)

     at com.sun.glass.ui.View.handleMouseEvent(View.java:556)

     at com.sun.glass.ui.View.notifyMouse(View.java:942)

     at com.sun.glass.ui.win.WinApplication._runLoop(Native Method)

     at com.sun.glass.ui.win.WinApplication.lambda$runLoop$3(WinApplication.java:174)

     at java.base/java.lang.Thread.run(Thread.java:829)

 Caused by: java.lang.OutOfMemoryError

     at java.base/jdk.internal.reflect.GeneratedConstructorAccessor3.newInstance(Unknown Source)

     at java.base/jdk.internal.reflect.DelegatingConstructorAccessorImpl.newInstance(DelegatingConstructorAccessorImpl.java:45)

     at java.base/java.lang.reflect.Constructor.newInstance(Constructor.java:490)

     at java.base/java.util.concurrent.ForkJoinTask.getThrowableException(ForkJoinTask.java:603)

     ... 49 more

 Caused by: java.lang.OutOfMemoryError: Java heap space

 java.util.concurrent.ExecutionException: java.lang.OutOfMemoryError

     at java.base/java.util.concurrent.ForkJoinTask.get(ForkJoinTask.java:1006)

     at fr.univ_nantes.codex_processor.gui.DatasetExtendedDepthOfFieldProcessor.run(DatasetExtendedDepthOfFieldProcessor.java:69)

     at fr.univ_nantes.codex_processor.gui.MainController$1.handle(MainController.java:96)

     at fr.univ_nantes.codex_processor.gui.MainController$1.handle(MainController.java:64)

     at com.sun.javafx.event.CompositeEventHandler.dispatchBubblingEvent(CompositeEventHandler.java:86)

     at com.sun.javafx.event.EventHandlerManager.dispatchBubblingEvent(EventHandlerManager.java:234)

     at com.sun.javafx.event.EventHandlerManager.dispatchBubblingEvent(EventHandlerManager.java:191)

     at com.sun.javafx.event.CompositeEventDispatcher.dispatchBubblingEvent(CompositeEventDispatcher.java:59)

     at com.sun.javafx.event.BasicEventDispatcher.dispatchEvent(BasicEventDispatcher.java:58)

     at com.sun.javafx.event.EventDispatchChainImpl.dispatchEvent(EventDispatchChainImpl.java:114)

     at com.sun.javafx.event.BasicEventDispatcher.dispatchEvent(BasicEventDispatcher.java:56)

     at com.sun.javafx.event.EventDispatchChainImpl.dispatchEvent(EventDispatchChainImpl.java:114)

     at com.sun.javafx.event.BasicEventDispatcher.dispatchEvent(BasicEventDispatcher.java:56)

     at com.sun.javafx.event.EventDispatchChainImpl.dispatchEvent(EventDispatchChainImpl.java:114)

     at com.sun.javafx.event.EventUtil.fireEventImpl(EventUtil.java:74)

     at com.sun.javafx.event.EventUtil.fireEvent(EventUtil.java:49)

     at javafx.event.Event.fireEvent(Event.java:198)

     at javafx.scene.Node.fireEvent(Node.java:8889)

     at javafx.scene.control.Button.fire(Button.java:203)

     at com.sun.javafx.scene.control.behavior.ButtonBehavior.mouseReleased(ButtonBehavior.java:208)

     at com.sun.javafx.scene.control.inputmap.InputMap.handle(InputMap.java:274)

     at com.sun.javafx.event.CompositeEventHandler$NormalEventHandlerRecord.handleBubblingEvent(CompositeEventHandler.java:247)

     at com.sun.javafx.event.CompositeEventHandler.dispatchBubblingEvent(CompositeEventHandler.java:80)

     at com.sun.javafx.event.EventHandlerManager.dispatchBubblingEvent(EventHandlerManager.java:234)

     at com.sun.javafx.event.EventHandlerManager.dispatchBubblingEvent(EventHandlerManager.java:191)

     at com.sun.javafx.event.CompositeEventDispatcher.dispatchBubblingEvent(CompositeEventDispatcher.java:59)

     at com.sun.javafx.event.BasicEventDispatcher.dispatchEvent(BasicEventDispatcher.java:58)

     at com.sun.javafx.event.EventDispatchChainImpl.dispatchEvent(EventDispatchChainImpl.java:114)

     at com.sun.javafx.event.BasicEventDispatcher.dispatchEvent(BasicEventDispatcher.java:56)

     at com.sun.javafx.event.EventDispatchChainImpl.dispatchEvent(EventDispatchChainImpl.java:114)

     at com.sun.javafx.event.BasicEventDispatcher.dispatchEvent(BasicEventDispatcher.java:56)

     at com.sun.javafx.event.EventDispatchChainImpl.dispatchEvent(EventDispatchChainImpl.java:114)

     at com.sun.javafx.event.EventUtil.fireEventImpl(EventUtil.java:74)

     at com.sun.javafx.event.EventUtil.fireEvent(EventUtil.java:54)

     at javafx.event.Event.fireEvent(Event.java:198)

     at javafx.scene.Scene$MouseHandler.process(Scene.java:3856)

     at javafx.scene.Scene.processMouseEvent(Scene.java:1851)

     at javafx.scene.Scene$ScenePeerListener.mouseEvent(Scene.java:2584)

     at com.sun.javafx.tk.quantum.GlassViewEventHandler$MouseEventNotification.run(GlassViewEventHandler.java:409)

     at com.sun.javafx.tk.quantum.GlassViewEventHandler$MouseEventNotification.run(GlassViewEventHandler.java:299)

     at java.base/java.security.AccessController.doPrivileged(Native Method)

     at com.sun.javafx.tk.quantum.GlassViewEventHandler.lambda$handleMouseEvent$2(GlassViewEventHandler.java:447)

     at com.sun.javafx.tk.quantum.QuantumToolkit.runWithoutRenderLock(QuantumToolkit.java:412)

     at com.sun.javafx.tk.quantum.GlassViewEventHandler.handleMouseEvent(GlassViewEventHandler.java:446)

     at com.sun.glass.ui.View.handleMouseEvent(View.java:556)

     at com.sun.glass.ui.View.notifyMouse(View.java:942)

     at com.sun.glass.ui.win.WinApplication._runLoop(Native Method)

     at com.sun.glass.ui.win.WinApplication.lambda$runLoop$3(WinApplication.java:174)

     at java.base/java.lang.Thread.run(Thread.java:829)

 Caused by: java.lang.OutOfMemoryError

     at java.base/jdk.internal.reflect.GeneratedConstructorAccessor3.newInstance(Unknown Source)

     at java.base/jdk.internal.reflect.DelegatingConstructorAccessorImpl.newInstance(DelegatingConstructorAccessorImpl.java:45)

     at java.base/java.lang.reflect.Constructor.newInstance(Constructor.java:490)

     at java.base/java.util.concurrent.ForkJoinTask.getThrowableException(ForkJoinTask.java:603)

     ... 49 more

 Caused by: java.lang.OutOfMemoryError: Java heap space

 java.util.concurrent.ExecutionException: java.lang.OutOfMemoryError

     at java.base/java.util.concurrent.ForkJoinTask.get(ForkJoinTask.java:1006)

     at fr.univ_nantes.codex_processor.gui.DatasetExtendedDepthOfFieldProcessor.run(DatasetExtendedDepthOfFieldProcessor.java:69)

     at fr.univ_nantes.codex_processor.gui.MainController$1.handle(MainController.java:96)

     at fr.univ_nantes.codex_processor.gui.MainController$1.handle(MainController.java:64)

     at com.sun.javafx.event.CompositeEventHandler.dispatchBubblingEvent(CompositeEventHandler.java:86)

     at com.sun.javafx.event.EventHandlerManager.dispatchBubblingEvent(EventHandlerManager.java:234)

     at com.sun.javafx.event.EventHandlerManager.dispatchBubblingEvent(EventHandlerManager.java:191)

     at com.sun.javafx.event.CompositeEventDispatcher.dispatchBubblingEvent(CompositeEventDispatcher.java:59)

     at com.sun.javafx.event.BasicEventDispatcher.dispatchEvent(BasicEventDispatcher.java:58)

     at com.sun.javafx.event.EventDispatchChainImpl.dispatchEvent(EventDispatchChainImpl.java:114)

     at com.sun.javafx.event.BasicEventDispatcher.dispatchEvent(BasicEventDispatcher.java:56)

     at com.sun.javafx.event.EventDispatchChainImpl.dispatchEvent(EventDispatchChainImpl.java:114)

     at com.sun.javafx.event.BasicEventDispatcher.dispatchEvent(BasicEventDispatcher.java:56)

     at com.sun.javafx.event.EventDispatchChainImpl.dispatchEvent(EventDispatchChainImpl.java:114)

     at com.sun.javafx.event.EventUtil.fireEventImpl(EventUtil.java:74)

     at com.sun.javafx.event.EventUtil.fireEvent(EventUtil.java:49)

     at javafx.event.Event.fireEvent(Event.java:198)

     at javafx.scene.Node.fireEvent(Node.java:8889)

     at javafx.scene.control.Button.fire(Button.java:203)

     at com.sun.javafx.scene.control.behavior.ButtonBehavior.mouseReleased(ButtonBehavior.java:208)

     at com.sun.javafx.scene.control.inputmap.InputMap.handle(InputMap.java:274)

     at com.sun.javafx.event.CompositeEventHandler$NormalEventHandlerRecord.handleBubblingEvent(CompositeEventHandler.java:247)

     at com.sun.javafx.event.CompositeEventHandler.dispatchBubblingEvent(CompositeEventHandler.java:80)

     at com.sun.javafx.event.EventHandlerManager.dispatchBubblingEvent(EventHandlerManager.java:234)

     at com.sun.javafx.event.EventHandlerManager.dispatchBubblingEvent(EventHandlerManager.java:191)

     at com.sun.javafx.event.CompositeEventDispatcher.dispatchBubblingEvent(CompositeEventDispatcher.java:59)

     at com.sun.javafx.event.BasicEventDispatcher.dispatchEvent(BasicEventDispatcher.java:58)

     at com.sun.javafx.event.EventDispatchChainImpl.dispatchEvent(EventDispatchChainImpl.java:114)

     at com.sun.javafx.event.BasicEventDispatcher.dispatchEvent(BasicEventDispatcher.java:56)

     at com.sun.javafx.event.EventDispatchChainImpl.dispatchEvent(EventDispatchChainImpl.java:114)

     at com.sun.javafx.event.BasicEventDispatcher.dispatchEvent(BasicEventDispatcher.java:56)

     at com.sun.javafx.event.EventDispatchChainImpl.dispatchEvent(EventDispatchChainImpl.java:114)

     at com.sun.javafx.event.EventUtil.fireEventImpl(EventUtil.java:74)

     at com.sun.javafx.event.EventUtil.fireEvent(EventUtil.java:54)

     at javafx.event.Event.fireEvent(Event.java:198)

     at javafx.scene.Scene$MouseHandler.process(Scene.java:3856)

     at javafx.scene.Scene.processMouseEvent(Scene.java:1851)

     at javafx.scene.Scene$ScenePeerListener.mouseEvent(Scene.java:2584)

     at com.sun.javafx.tk.quantum.GlassViewEventHandler$MouseEventNotification.run(GlassViewEventHandler.java:409)

     at com.sun.javafx.tk.quantum.GlassViewEventHandler$MouseEventNotification.run(GlassViewEventHandler.java:299)

     at java.base/java.security.AccessController.doPrivileged(Native Method)

     at com.sun.javafx.tk.quantum.GlassViewEventHandler.lambda$handleMouseEvent$2(GlassViewEventHandler.java:447)

     at com.sun.javafx.tk.quantum.QuantumToolkit.runWithoutRenderLock(QuantumToolkit.java:412)

     at com.sun.javafx.tk.quantum.GlassViewEventHandler.handleMouseEvent(GlassViewEventHandler.java:446)

     at com.sun.glass.ui.View.handleMouseEvent(View.java:556)

     at com.sun.glass.ui.View.notifyMouse(View.java:942)

     at com.sun.glass.ui.win.WinApplication._runLoop(Native Method)

     at com.sun.glass.ui.win.WinApplication.lambda$runLoop$3(WinApplication.java:174)

     at java.base/java.lang.Thread.run(Thread.java:829)

 Caused by: java.lang.OutOfMemoryError

     at java.base/jdk.internal.reflect.GeneratedConstructorAccessor3.newInstance(Unknown Source)

     at java.base/jdk.internal.reflect.DelegatingConstructorAccessorImpl.newInstance(DelegatingConstructorAccessorImpl.java:45)

     at java.base/java.lang.reflect.Constructor.newInstance(Constructor.java:490)

     at java.base/java.util.concurrent.ForkJoinTask.getThrowableException(ForkJoinTask.java:603)

     ... 49 more

 Caused by: java.lang.OutOfMemoryError: Java heap space

 java.util.concurrent.ExecutionException: java.lang.OutOfMemoryError

     at java.base/java.util.concurrent.ForkJoinTask.get(ForkJoinTask.java:1006)

     at fr.univ_nantes.codex_processor.gui.DatasetExtendedDepthOfFieldProcessor.run(DatasetExtendedDepthOfFieldProcessor.java:69)

     at fr.univ_nantes.codex_processor.gui.MainController$1.handle(MainController.java:96)

     at fr.univ_nantes.codex_processor.gui.MainController$1.handle(MainController.java:64)

     at com.sun.javafx.event.CompositeEventHandler.dispatchBubblingEvent(CompositeEventHandler.java:86)

     at com.sun.javafx.event.EventHandlerManager.dispatchBubblingEvent(EventHandlerManager.java:234)

     at com.sun.javafx.event.EventHandlerManager.dispatchBubblingEvent(EventHandlerManager.java:191)

     at com.sun.javafx.event.CompositeEventDispatcher.dispatchBubblingEvent(CompositeEventDispatcher.java:59)

     at com.sun.javafx.event.BasicEventDispatcher.dispatchEvent(BasicEventDispatcher.java:58)

     at com.sun.javafx.event.EventDispatchChainImpl.dispatchEvent(EventDispatchChainImpl.java:114)

     at com.sun.javafx.event.BasicEventDispatcher.dispatchEvent(BasicEventDispatcher.java:56)

     at com.sun.javafx.event.EventDispatchChainImpl.dispatchEvent(EventDispatchChainImpl.java:114)

     at com.sun.javafx.event.BasicEventDispatcher.dispatchEvent(BasicEventDispatcher.java:56)

     at com.sun.javafx.event.EventDispatchChainImpl.dispatchEvent(EventDispatchChainImpl.java:114)

     at com.sun.javafx.event.EventUtil.fireEventImpl(EventUtil.java:74)

     at com.sun.javafx.event.EventUtil.fireEvent(EventUtil.java:49)

     at javafx.event.Event.fireEvent(Event.java:198)

     at javafx.scene.Node.fireEvent(Node.java:8889)

     at javafx.scene.control.Button.fire(Button.java:203)

     at com.sun.javafx.scene.control.behavior.ButtonBehavior.mouseReleased(ButtonBehavior.java:208)

     at com.sun.javafx.scene.control.inputmap.InputMap.handle(InputMap.java:274)

     at com.sun.javafx.event.CompositeEventHandler$NormalEventHandlerRecord.handleBubblingEvent(CompositeEventHandler.java:247)

     at com.sun.javafx.event.CompositeEventHandler.dispatchBubblingEvent(CompositeEventHandler.java:80)

     at com.sun.javafx.event.EventHandlerManager.dispatchBubblingEvent(EventHandlerManager.java:234)

     at com.sun.javafx.event.EventHandlerManager.dispatchBubblingEvent(EventHandlerManager.java:191)

     at com.sun.javafx.event.CompositeEventDispatcher.dispatchBubblingEvent(CompositeEventDispatcher.java:59)

     at com.sun.javafx.event.BasicEventDispatcher.dispatchEvent(BasicEventDispatcher.java:58)

     at com.sun.javafx.event.EventDispatchChainImpl.dispatchEvent(EventDispatchChainImpl.java:114)

     at com.sun.javafx.event.BasicEventDispatcher.dispatchEvent(BasicEventDispatcher.java:56)

     at com.sun.javafx.event.EventDispatchChainImpl.dispatchEvent(EventDispatchChainImpl.java:114)

     at com.sun.javafx.event.BasicEventDispatcher.dispatchEvent(BasicEventDispatcher.java:56)

     at com.sun.javafx.event.EventDispatchChainImpl.dispatchEvent(EventDispatchChainImpl.java:114)

     at com.sun.javafx.event.EventUtil.fireEventImpl(EventUtil.java:74)

     at com.sun.javafx.event.EventUtil.fireEvent(EventUtil.java:54)

     at javafx.event.Event.fireEvent(Event.java:198)

     at javafx.scene.Scene$MouseHandler.process(Scene.java:3856)

     at javafx.scene.Scene.processMouseEvent(Scene.java:1851)

     at javafx.scene.Scene$ScenePeerListener.mouseEvent(Scene.java:2584)

     at com.sun.javafx.tk.quantum.GlassViewEventHandler$MouseEventNotification.run(GlassViewEventHandler.java:409)

     at com.sun.javafx.tk.quantum.GlassViewEventHandler$MouseEventNotification.run(GlassViewEventHandler.java:299)

     at java.base/java.security.AccessController.doPrivileged(Native Method)

     at com.sun.javafx.tk.quantum.GlassViewEventHandler.lambda$handleMouseEvent$2(GlassViewEventHandler.java:447)

     at com.sun.javafx.tk.quantum.QuantumToolkit.runWithoutRenderLock(QuantumToolkit.java:412)

     at com.sun.javafx.tk.quantum.GlassViewEventHandler.handleMouseEvent(GlassViewEventHandler.java:446)

     at com.sun.glass.ui.View.handleMouseEvent(View.java:556)

     at com.sun.glass.ui.View.notifyMouse(View.java:942)

     at com.sun.glass.ui.win.WinApplication._runLoop(Native Method)

     at com.sun.glass.ui.win.WinApplication.lambda$runLoop$3(WinApplication.java:174)

     at java.base/java.lang.Thread.run(Thread.java:829)

 Caused by: java.lang.OutOfMemoryError

     at java.base/jdk.internal.reflect.GeneratedConstructorAccessor3.newInstance(Unknown Source)

     at java.base/jdk.internal.reflect.DelegatingConstructorAccessorImpl.newInstance(DelegatingConstructorAccessorImpl.java:45)

     at java.base/java.lang.reflect.Constructor.newInstance(Constructor.java:490)

     at java.base/java.util.concurrent.ForkJoinTask.getThrowableException(ForkJoinTask.java:603)

     ... 49 more

 Caused by: java.lang.OutOfMemoryError: Java heap space

 java.util.concurrent.ExecutionException: java.lang.OutOfMemoryError

     at java.base/java.util.concurrent.ForkJoinTask.get(ForkJoinTask.java:1006)

     at fr.univ_nantes.codex_processor.gui.DatasetExtendedDepthOfFieldProcessor.run(DatasetExtendedDepthOfFieldProcessor.java:69)

     at fr.univ_nantes.codex_processor.gui.MainController$1.handle(MainController.java:96)

     at fr.univ_nantes.codex_processor.gui.MainController$1.handle(MainController.java:64)

     at com.sun.javafx.event.CompositeEventHandler.dispatchBubblingEvent(CompositeEventHandler.java:86)

     at com.sun.javafx.event.EventHandlerManager.dispatchBubblingEvent(EventHandlerManager.java:234)

     at com.sun.javafx.event.EventHandlerManager.dispatchBubblingEvent(EventHandlerManager.java:191)

     at com.sun.javafx.event.CompositeEventDispatcher.dispatchBubblingEvent(CompositeEventDispatcher.java:59)

     at com.sun.javafx.event.BasicEventDispatcher.dispatchEvent(BasicEventDispatcher.java:58)

     at com.sun.javafx.event.EventDispatchChainImpl.dispatchEvent(EventDispatchChainImpl.java:114)

     at com.sun.javafx.event.BasicEventDispatcher.dispatchEvent(BasicEventDispatcher.java:56)

     at com.sun.javafx.event.EventDispatchChainImpl.dispatchEvent(EventDispatchChainImpl.java:114)

     at com.sun.javafx.event.BasicEventDispatcher.dispatchEvent(BasicEventDispatcher.java:56)

     at com.sun.javafx.event.EventDispatchChainImpl.dispatchEvent(EventDispatchChainImpl.java:114)

     at com.sun.javafx.event.EventUtil.fireEventImpl(EventUtil.java:74)

     at com.sun.javafx.event.EventUtil.fireEvent(EventUtil.java:49)

     at javafx.event.Event.fireEvent(Event.java:198)

     at javafx.scene.Node.fireEvent(Node.java:8889)

     at javafx.scene.control.Button.fire(Button.java:203)

     at com.sun.javafx.scene.control.behavior.ButtonBehavior.mouseReleased(ButtonBehavior.java:208)

     at com.sun.javafx.scene.control.inputmap.InputMap.handle(InputMap.java:274)

     at com.sun.javafx.event.CompositeEventHandler$NormalEventHandlerRecord.handleBubblingEvent(CompositeEventHandler.java:247)

     at com.sun.javafx.event.CompositeEventHandler.dispatchBubblingEvent(CompositeEventHandler.java:80)

     at com.sun.javafx.event.EventHandlerManager.dispatchBubblingEvent(EventHandlerManager.java:234)

     at com.sun.javafx.event.EventHandlerManager.dispatchBubblingEvent(EventHandlerManager.java:191)

     at com.sun.javafx.event.CompositeEventDispatcher.dispatchBubblingEvent(CompositeEventDispatcher.java:59)

     at com.sun.javafx.event.BasicEventDispatcher.dispatchEvent(BasicEventDispatcher.java:58)

     at com.sun.javafx.event.EventDispatchChainImpl.dispatchEvent(EventDispatchChainImpl.java:114)

     at com.sun.javafx.event.BasicEventDispatcher.dispatchEvent(BasicEventDispatcher.java:56)

     at com.sun.javafx.event.EventDispatchChainImpl.dispatchEvent(EventDispatchChainImpl.java:114)

     at com.sun.javafx.event.BasicEventDispatcher.dispatchEvent(BasicEventDispatcher.java:56)

     at com.sun.javafx.event.EventDispatchChainImpl.dispatchEvent(EventDispatchChainImpl.java:114)

     at com.sun.javafx.event.EventUtil.fireEventImpl(EventUtil.java:74)

     at com.sun.javafx.event.EventUtil.fireEvent(EventUtil.java:54)

     at javafx.event.Event.fireEvent(Event.java:198)

     at javafx.scene.Scene$MouseHandler.process(Scene.java:3856)

     at javafx.scene.Scene.processMouseEvent(Scene.java:1851)

     at javafx.scene.Scene$ScenePeerListener.mouseEvent(Scene.java:2584)

     at com.sun.javafx.tk.quantum.GlassViewEventHandler$MouseEventNotification.run(GlassViewEventHandler.java:409)

     at com.sun.javafx.tk.quantum.GlassViewEventHandler$MouseEventNotification.run(GlassViewEventHandler.java:299)

     at java.base/java.security.AccessController.doPrivileged(Native Method)

     at com.sun.javafx.tk.quantum.GlassViewEventHandler.lambda$handleMouseEvent$2(GlassViewEventHandler.java:447)

     at com.sun.javafx.tk.quantum.QuantumToolkit.runWithoutRenderLock(QuantumToolkit.java:412)

     at com.sun.javafx.tk.quantum.GlassViewEventHandler.handleMouseEvent(GlassViewEventHandler.java:446)

     at com.sun.glass.ui.View.handleMouseEvent(View.java:556)

     at com.sun.glass.ui.View.notifyMouse(View.java:942)

     at com.sun.glass.ui.win.WinApplication._runLoop(Native Method)

     at com.sun.glass.ui.win.WinApplication.lambda$runLoop$3(WinApplication.java:174)

     at java.base/java.lang.Thread.run(Thread.java:829)

 Caused by: java.lang.OutOfMemoryError

     at java.base/jdk.internal.reflect.GeneratedConstructorAccessor3.newInstance(Unknown Source)

     at java.base/jdk.internal.reflect.DelegatingConstructorAccessorImpl.newInstance(DelegatingConstructorAccessorImpl.java:45)

     at java.base/java.lang.reflect.Constructor.newInstance(Constructor.java:490)

     at java.base/java.util.concurrent.ForkJoinTask.getThrowableException(ForkJoinTask.java:603)

     ... 49 more

 Caused by: java.lang.OutOfMemoryError: Java heap space

 java.util.concurrent.ExecutionException: java.lang.OutOfMemoryError

     at java.base/java.util.concurrent.ForkJoinTask.get(ForkJoinTask.java:1006)

     at fr.univ_nantes.codex_processor.gui.DatasetExtendedDepthOfFieldProcessor.run(DatasetExtendedDepthOfFieldProcessor.java:69)

     at fr.univ_nantes.codex_processor.gui.MainController$1.handle(MainController.java:96)

     at fr.univ_nantes.codex_processor.gui.MainController$1.handle(MainController.java:64)

     at com.sun.javafx.event.CompositeEventHandler.dispatchBubblingEvent(CompositeEventHandler.java:86)

     at com.sun.javafx.event.EventHandlerManager.dispatchBubblingEvent(EventHandlerManager.java:234)

     at com.sun.javafx.event.EventHandlerManager.dispatchBubblingEvent(EventHandlerManager.java:191)

     at com.sun.javafx.event.CompositeEventDispatcher.dispatchBubblingEvent(CompositeEventDispatcher.java:59)

     at com.sun.javafx.event.BasicEventDispatcher.dispatchEvent(BasicEventDispatcher.java:58)

     at com.sun.javafx.event.EventDispatchChainImpl.dispatchEvent(EventDispatchChainImpl.java:114)

     at com.sun.javafx.event.BasicEventDispatcher.dispatchEvent(BasicEventDispatcher.java:56)

     at com.sun.javafx.event.EventDispatchChainImpl.dispatchEvent(EventDispatchChainImpl.java:114)

     at com.sun.javafx.event.BasicEventDispatcher.dispatchEvent(BasicEventDispatcher.java:56)

     at com.sun.javafx.event.EventDispatchChainImpl.dispatchEvent(EventDispatchChainImpl.java:114)

     at com.sun.javafx.event.EventUtil.fireEventImpl(EventUtil.java:74)

     at com.sun.javafx.event.EventUtil.fireEvent(EventUtil.java:49)

     at javafx.event.Event.fireEvent(Event.java:198)

     at javafx.scene.Node.fireEvent(Node.java:8889)

     at javafx.scene.control.Button.fire(Button.java:203)

     at com.sun.javafx.scene.control.behavior.ButtonBehavior.mouseReleased(ButtonBehavior.java:208)

     at com.sun.javafx.scene.control.inputmap.InputMap.handle(InputMap.java:274)

     at com.sun.javafx.event.CompositeEventHandler$NormalEventHandlerRecord.handleBubblingEvent(CompositeEventHandler.java:247)

     at com.sun.javafx.event.CompositeEventHandler.dispatchBubblingEvent(CompositeEventHandler.java:80)

     at com.sun.javafx.event.EventHandlerManager.dispatchBubblingEvent(EventHandlerManager.java:234)

     at com.sun.javafx.event.EventHandlerManager.dispatchBubblingEvent(EventHandlerManager.java:191)

     at com.sun.javafx.event.CompositeEventDispatcher.dispatchBubblingEvent(CompositeEventDispatcher.java:59)

     at com.sun.javafx.event.BasicEventDispatcher.dispatchEvent(BasicEventDispatcher.java:58)

     at com.sun.javafx.event.EventDispatchChainImpl.dispatchEvent(EventDispatchChainImpl.java:114)

     at com.sun.javafx.event.BasicEventDispatcher.dispatchEvent(BasicEventDispatcher.java:56)

     at com.sun.javafx.event.EventDispatchChainImpl.dispatchEvent(EventDispatchChainImpl.java:114)

     at com.sun.javafx.event.BasicEventDispatcher.dispatchEvent(BasicEventDispatcher.java:56)

     at com.sun.javafx.event.EventDispatchChainImpl.dispatchEvent(EventDispatchChainImpl.java:114)

     at com.sun.javafx.event.EventUtil.fireEventImpl(EventUtil.java:74)

     at com.sun.javafx.event.EventUtil.fireEvent(EventUtil.java:54)

     at javafx.event.Event.fireEvent(Event.java:198)

     at javafx.scene.Scene$MouseHandler.process(Scene.java:3856)

     at javafx.scene.Scene.processMouseEvent(Scene.java:1851)

     at javafx.scene.Scene$ScenePeerListener.mouseEvent(Scene.java:2584)

     at com.sun.javafx.tk.quantum.GlassViewEventHandler$MouseEventNotification.run(GlassViewEventHandler.java:409)

     at com.sun.javafx.tk.quantum.GlassViewEventHandler$MouseEventNotification.run(GlassViewEventHandler.java:299)

     at java.base/java.security.AccessController.doPrivileged(Native Method)

     at com.sun.javafx.tk.quantum.GlassViewEventHandler.lambda$handleMouseEvent$2(GlassViewEventHandler.java:447)

     at com.sun.javafx.tk.quantum.QuantumToolkit.runWithoutRenderLock(QuantumToolkit.java:412)

     at com.sun.javafx.tk.quantum.GlassViewEventHandler.handleMouseEvent(GlassViewEventHandler.java:446)

     at com.sun.glass.ui.View.handleMouseEvent(View.java:556)

     at com.sun.glass.ui.View.notifyMouse(View.java:942)

     at com.sun.glass.ui.win.WinApplication._runLoop(Native Method)

     at com.sun.glass.ui.win.WinApplication.lambda$runLoop$3(WinApplication.java:174)

     at java.base/java.lang.Thread.run(Thread.java:829)

 Caused by: java.lang.OutOfMemoryError

     at java.base/jdk.internal.reflect.GeneratedConstructorAccessor3.newInstance(Unknown Source)

     at java.base/jdk.internal.reflect.DelegatingConstructorAccessorImpl.newInstance(DelegatingConstructorAccessorImpl.java:45)

     at java.base/java.lang.reflect.Constructor.newInstance(Constructor.java:490)

     at java.base/java.util.concurrent.ForkJoinTask.getThrowableException(ForkJoinTask.java:603)

     ... 49 more

 Caused by: java.lang.OutOfMemoryError: Java heap space

 java.util.concurrent.ExecutionException: java.lang.OutOfMemoryError

     at java.base/java.util.concurrent.ForkJoinTask.get(ForkJoinTask.java:1006)

     at fr.univ_nantes.codex_processor.gui.DatasetExtendedDepthOfFieldProcessor.run(DatasetExtendedDepthOfFieldProcessor.java:69)

     at fr.univ_nantes.codex_processor.gui.MainController$1.handle(MainController.java:96)

     at fr.univ_nantes.codex_processor.gui.MainController$1.handle(MainController.java:64)

     at com.sun.javafx.event.CompositeEventHandler.dispatchBubblingEvent(CompositeEventHandler.java:86)

     at com.sun.javafx.event.EventHandlerManager.dispatchBubblingEvent(EventHandlerManager.java:234)

     at com.sun.javafx.event.EventHandlerManager.dispatchBubblingEvent(EventHandlerManager.java:191)

     at com.sun.javafx.event.CompositeEventDispatcher.dispatchBubblingEvent(CompositeEventDispatcher.java:59)

     at com.sun.javafx.event.BasicEventDispatcher.dispatchEvent(BasicEventDispatcher.java:58)

     at com.sun.javafx.event.EventDispatchChainImpl.dispatchEvent(EventDispatchChainImpl.java:114)

     at com.sun.javafx.event.BasicEventDispatcher.dispatchEvent(BasicEventDispatcher.java:56)

     at com.sun.javafx.event.EventDispatchChainImpl.dispatchEvent(EventDispatchChainImpl.java:114)

     at com.sun.javafx.event.BasicEventDispatcher.dispatchEvent(BasicEventDispatcher.java:56)

     at com.sun.javafx.event.EventDispatchChainImpl.dispatchEvent(EventDispatchChainImpl.java:114)

     at com.sun.javafx.event.EventUtil.fireEventImpl(EventUtil.java:74)

     at com.sun.javafx.event.EventUtil.fireEvent(EventUtil.java:49)

     at javafx.event.Event.fireEvent(Event.java:198)

     at javafx.scene.Node.fireEvent(Node.java:8889)

     at javafx.scene.control.Button.fire(Button.java:203)

     at com.sun.javafx.scene.control.behavior.ButtonBehavior.mouseReleased(ButtonBehavior.java:208)

     at com.sun.javafx.scene.control.inputmap.InputMap.handle(InputMap.java:274)

     at com.sun.javafx.event.CompositeEventHandler$NormalEventHandlerRecord.handleBubblingEvent(CompositeEventHandler.java:247)

     at com.sun.javafx.event.CompositeEventHandler.dispatchBubblingEvent(CompositeEventHandler.java:80)

     at com.sun.javafx.event.EventHandlerManager.dispatchBubblingEvent(EventHandlerManager.java:234)

     at com.sun.javafx.event.EventHandlerManager.dispatchBubblingEvent(EventHandlerManager.java:191)

     at com.sun.javafx.event.CompositeEventDispatcher.dispatchBubblingEvent(CompositeEventDispatcher.java:59)

     at com.sun.javafx.event.BasicEventDispatcher.dispatchEvent(BasicEventDispatcher.java:58)

     at com.sun.javafx.event.EventDispatchChainImpl.dispatchEvent(EventDispatchChainImpl.java:114)

     at com.sun.javafx.event.BasicEventDispatcher.dispatchEvent(BasicEventDispatcher.java:56)

     at com.sun.javafx.event.EventDispatchChainImpl.dispatchEvent(EventDispatchChainImpl.java:114)

     at com.sun.javafx.event.BasicEventDispatcher.dispatchEvent(BasicEventDispatcher.java:56)

     at com.sun.javafx.event.EventDispatchChainImpl.dispatchEvent(EventDispatchChainImpl.java:114)

     at com.sun.javafx.event.EventUtil.fireEventImpl(EventUtil.java:74)

     at com.sun.javafx.event.EventUtil.fireEvent(EventUtil.java:54)

     at javafx.event.Event.fireEvent(Event.java:198)

     at javafx.scene.Scene$MouseHandler.process(Scene.java:3856)

     at javafx.scene.Scene.processMouseEvent(Scene.java:1851)

     at javafx.scene.Scene$ScenePeerListener.mouseEvent(Scene.java:2584)

     at com.sun.javafx.tk.quantum.GlassViewEventHandler$MouseEventNotification.run(GlassViewEventHandler.java:409)

     at com.sun.javafx.tk.quantum.GlassViewEventHandler$MouseEventNotification.run(GlassViewEventHandler.java:299)

     at java.base/java.security.AccessController.doPrivileged(Native Method)

     at com.sun.javafx.tk.quantum.GlassViewEventHandler.lambda$handleMouseEvent$2(GlassViewEventHandler.java:447)

     at com.sun.javafx.tk.quantum.QuantumToolkit.runWithoutRenderLock(QuantumToolkit.java:412)

     at com.sun.javafx.tk.quantum.GlassViewEventHandler.handleMouseEvent(GlassViewEventHandler.java:446)

     at com.sun.glass.ui.View.handleMouseEvent(View.java:556)

     at com.sun.glass.ui.View.notifyMouse(View.java:942)

     at com.sun.glass.ui.win.WinApplication._runLoop(Native Method)

     at com.sun.glass.ui.win.WinApplication.lambda$runLoop$3(WinApplication.java:174)

     at java.base/java.lang.Thread.run(Thread.java:829)

 Caused by: java.lang.OutOfMemoryError

     at java.base/jdk.internal.reflect.GeneratedConstructorAccessor3.newInstance(Unknown Source)

     at java.base/jdk.internal.reflect.DelegatingConstructorAccessorImpl.newInstance(DelegatingConstructorAccessorImpl.java:45)

     at java.base/java.lang.reflect.Constructor.newInstance(Constructor.java:490)

     at java.base/java.util.concurrent.ForkJoinTask.getThrowableException(ForkJoinTask.java:603)

     ... 49 more

 Caused by: java.lang.OutOfMemoryError: Java heap space

 java.util.concurrent.ExecutionException: java.lang.OutOfMemoryError

     at java.base/java.util.concurrent.ForkJoinTask.get(ForkJoinTask.java:1006)

     at fr.univ_nantes.codex_processor.gui.DatasetExtendedDepthOfFieldProcessor.run(DatasetExtendedDepthOfFieldProcessor.java:69)

     at fr.univ_nantes.codex_processor.gui.MainController$1.handle(MainController.java:96)

     at fr.univ_nantes.codex_processor.gui.MainController$1.handle(MainController.java:64)

     at com.sun.javafx.event.CompositeEventHandler.dispatchBubblingEvent(CompositeEventHandler.java:86)

     at com.sun.javafx.event.EventHandlerManager.dispatchBubblingEvent(EventHandlerManager.java:234)

     at com.sun.javafx.event.EventHandlerManager.dispatchBubblingEvent(EventHandlerManager.java:191)

     at com.sun.javafx.event.CompositeEventDispatcher.dispatchBubblingEvent(CompositeEventDispatcher.java:59)

     at com.sun.javafx.event.BasicEventDispatcher.dispatchEvent(BasicEventDispatcher.java:58)

     at com.sun.javafx.event.EventDispatchChainImpl.dispatchEvent(EventDispatchChainImpl.java:114)

     at com.sun.javafx.event.BasicEventDispatcher.dispatchEvent(BasicEventDispatcher.java:56)

     at com.sun.javafx.event.EventDispatchChainImpl.dispatchEvent(EventDispatchChainImpl.java:114)

     at com.sun.javafx.event.BasicEventDispatcher.dispatchEvent(BasicEventDispatcher.java:56)

     at com.sun.javafx.event.EventDispatchChainImpl.dispatchEvent(EventDispatchChainImpl.java:114)

     at com.sun.javafx.event.EventUtil.fireEventImpl(EventUtil.java:74)

     at com.sun.javafx.event.EventUtil.fireEvent(EventUtil.java:49)

     at javafx.event.Event.fireEvent(Event.java:198)

     at javafx.scene.Node.fireEvent(Node.java:8889)

     at javafx.scene.control.Button.fire(Button.java:203)

     at com.sun.javafx.scene.control.behavior.ButtonBehavior.mouseReleased(ButtonBehavior.java:208)

     at com.sun.javafx.scene.control.inputmap.InputMap.handle(InputMap.java:274)

     at com.sun.javafx.event.CompositeEventHandler$NormalEventHandlerRecord.handleBubblingEvent(CompositeEventHandler.java:247)

     at com.sun.javafx.event.CompositeEventHandler.dispatchBubblingEvent(CompositeEventHandler.java:80)

     at com.sun.javafx.event.EventHandlerManager.dispatchBubblingEvent(EventHandlerManager.java:234)

     at com.sun.javafx.event.EventHandlerManager.dispatchBubblingEvent(EventHandlerManager.java:191)

     at com.sun.javafx.event.CompositeEventDispatcher.dispatchBubblingEvent(CompositeEventDispatcher.java:59)

     at com.sun.javafx.event.BasicEventDispatcher.dispatchEvent(BasicEventDispatcher.java:58)

     at com.sun.javafx.event.EventDispatchChainImpl.dispatchEvent(EventDispatchChainImpl.java:114)

     at com.sun.javafx.event.BasicEventDispatcher.dispatchEvent(BasicEventDispatcher.java:56)

     at com.sun.javafx.event.EventDispatchChainImpl.dispatchEvent(EventDispatchChainImpl.java:114)

     at com.sun.javafx.event.BasicEventDispatcher.dispatchEvent(BasicEventDispatcher.java:56)

     at com.sun.javafx.event.EventDispatchChainImpl.dispatchEvent(EventDispatchChainImpl.java:114)

     at com.sun.javafx.event.EventUtil.fireEventImpl(EventUtil.java:74)

     at com.sun.javafx.event.EventUtil.fireEvent(EventUtil.java:54)

     at javafx.event.Event.fireEvent(Event.java:198)

     at javafx.scene.Scene$MouseHandler.process(Scene.java:3856)

     at javafx.scene.Scene.processMouseEvent(Scene.java:1851)

     at javafx.scene.Scene$ScenePeerListener.mouseEvent(Scene.java:2584)

     at com.sun.javafx.tk.quantum.GlassViewEventHandler$MouseEventNotification.run(GlassViewEventHandler.java:409)

     at com.sun.javafx.tk.quantum.GlassViewEventHandler$MouseEventNotification.run(GlassViewEventHandler.java:299)

     at java.base/java.security.AccessController.doPrivileged(Native Method)

     at com.sun.javafx.tk.quantum.GlassViewEventHandler.lambda$handleMouseEvent$2(GlassViewEventHandler.java:447)

     at com.sun.javafx.tk.quantum.QuantumToolkit.runWithoutRenderLock(QuantumToolkit.java:412)

     at com.sun.javafx.tk.quantum.GlassViewEventHandler.handleMouseEvent(GlassViewEventHandler.java:446)

     at com.sun.glass.ui.View.handleMouseEvent(View.java:556)

     at com.sun.glass.ui.View.notifyMouse(View.java:942)

     at com.sun.glass.ui.win.WinApplication._runLoop(Native Method)

     at com.sun.glass.ui.win.WinApplication.lambda$runLoop$3(WinApplication.java:174)

     at java.base/java.lang.Thread.run(Thread.java:829)

 Caused by: java.lang.OutOfMemoryError

     at java.base/jdk.internal.reflect.GeneratedConstructorAccessor3.newInstance(Unknown Source)

     at java.base/jdk.internal.reflect.DelegatingConstructorAccessorImpl.newInstance(DelegatingConstructorAccessorImpl.java:45)

     at java.base/java.lang.reflect.Constructor.newInstance(Constructor.java:490)

     at java.base/java.util.concurrent.ForkJoinTask.getThrowableException(ForkJoinTask.java:603)

     ... 49 more

 Caused by: java.lang.OutOfMemoryError: Java heap space

 java.util.concurrent.ExecutionException: java.lang.OutOfMemoryError

     at java.base/java.util.concurrent.ForkJoinTask.get(ForkJoinTask.java:1006)

     at fr.univ_nantes.codex_processor.gui.DatasetExtendedDepthOfFieldProcessor.run(DatasetExtendedDepthOfFieldProcessor.java:69)

     at fr.univ_nantes.codex_processor.gui.MainController$1.handle(MainController.java:96)

     at fr.univ_nantes.codex_processor.gui.MainController$1.handle(MainController.java:64)

     at com.sun.javafx.event.CompositeEventHandler.dispatchBubblingEvent(CompositeEventHandler.java:86)

     at com.sun.javafx.event.EventHandlerManager.dispatchBubblingEvent(EventHandlerManager.java:234)

     at com.sun.javafx.event.EventHandlerManager.dispatchBubblingEvent(EventHandlerManager.java:191)

     at com.sun.javafx.event.CompositeEventDispatcher.dispatchBubblingEvent(CompositeEventDispatcher.java:59)

     at com.sun.javafx.event.BasicEventDispatcher.dispatchEvent(BasicEventDispatcher.java:58)

     at com.sun.javafx.event.EventDispatchChainImpl.dispatchEvent(EventDispatchChainImpl.java:114)

     at com.sun.javafx.event.BasicEventDispatcher.dispatchEvent(BasicEventDispatcher.java:56)

     at com.sun.javafx.event.EventDispatchChainImpl.dispatchEvent(EventDispatchChainImpl.java:114)

     at com.sun.javafx.event.BasicEventDispatcher.dispatchEvent(BasicEventDispatcher.java:56)

     at com.sun.javafx.event.EventDispatchChainImpl.dispatchEvent(EventDispatchChainImpl.java:114)

     at com.sun.javafx.event.EventUtil.fireEventImpl(EventUtil.java:74)

     at com.sun.javafx.event.EventUtil.fireEvent(EventUtil.java:49)

     at javafx.event.Event.fireEvent(Event.java:198)

     at javafx.scene.Node.fireEvent(Node.java:8889)

     at javafx.scene.control.Button.fire(Button.java:203)

     at com.sun.javafx.scene.control.behavior.ButtonBehavior.mouseReleased(ButtonBehavior.java:208)

     at com.sun.javafx.scene.control.inputmap.InputMap.handle(InputMap.java:274)

     at com.sun.javafx.event.CompositeEventHandler$NormalEventHandlerRecord.handleBubblingEvent(CompositeEventHandler.java:247)

     at com.sun.javafx.event.CompositeEventHandler.dispatchBubblingEvent(CompositeEventHandler.java:80)

     at com.sun.javafx.event.EventHandlerManager.dispatchBubblingEvent(EventHandlerManager.java:234)

     at com.sun.javafx.event.EventHandlerManager.dispatchBubblingEvent(EventHandlerManager.java:191)

     at com.sun.javafx.event.CompositeEventDispatcher.dispatchBubblingEvent(CompositeEventDispatcher.java:59)

     at com.sun.javafx.event.BasicEventDispatcher.dispatchEvent(BasicEventDispatcher.java:58)

     at com.sun.javafx.event.EventDispatchChainImpl.dispatchEvent(EventDispatchChainImpl.java:114)

     at com.sun.javafx.event.BasicEventDispatcher.dispatchEvent(BasicEventDispatcher.java:56)

     at com.sun.javafx.event.EventDispatchChainImpl.dispatchEvent(EventDispatchChainImpl.java:114)

     at com.sun.javafx.event.BasicEventDispatcher.dispatchEvent(BasicEventDispatcher.java:56)

     at com.sun.javafx.event.EventDispatchChainImpl.dispatchEvent(EventDispatchChainImpl.java:114)

     at com.sun.javafx.event.EventUtil.fireEventImpl(EventUtil.java:74)

     at com.sun.javafx.event.EventUtil.fireEvent(EventUtil.java:54)

     at javafx.event.Event.fireEvent(Event.java:198)

     at javafx.scene.Scene$MouseHandler.process(Scene.java:3856)

     at javafx.scene.Scene.processMouseEvent(Scene.java:1851)

     at javafx.scene.Scene$ScenePeerListener.mouseEvent(Scene.java:2584)

     at com.sun.javafx.tk.quantum.GlassViewEventHandler$MouseEventNotification.run(GlassViewEventHandler.java:409)

     at com.sun.javafx.tk.quantum.GlassViewEventHandler$MouseEventNotification.run(GlassViewEventHandler.java:299)

     at java.base/java.security.AccessController.doPrivileged(Native Method)

     at com.sun.javafx.tk.quantum.GlassViewEventHandler.lambda$handleMouseEvent$2(GlassViewEventHandler.java:447)

     at com.sun.javafx.tk.quantum.QuantumToolkit.runWithoutRenderLock(QuantumToolkit.java:412)

     at com.sun.javafx.tk.quantum.GlassViewEventHandler.handleMouseEvent(GlassViewEventHandler.java:446)

     at com.sun.glass.ui.View.handleMouseEvent(View.java:556)

     at com.sun.glass.ui.View.notifyMouse(View.java:942)

     at com.sun.glass.ui.win.WinApplication._runLoop(Native Method)

     at com.sun.glass.ui.win.WinApplication.lambda$runLoop$3(WinApplication.java:174)

     at java.base/java.lang.Thread.run(Thread.java:829)

 Caused by: java.lang.OutOfMemoryError

     at java.base/jdk.internal.reflect.GeneratedConstructorAccessor3.newInstance(Unknown Source)

     at java.base/jdk.internal.reflect.DelegatingConstructorAccessorImpl.newInstance(DelegatingConstructorAccessorImpl.java:45)

     at java.base/java.lang.reflect.Constructor.newInstance(Constructor.java:490)

     at java.base/java.util.concurrent.ForkJoinTask.getThrowableException(ForkJoinTask.java:603)

     ... 49 more

 Caused by: java.lang.OutOfMemoryError: Java heap space

 java.util.concurrent.ExecutionException: java.lang.OutOfMemoryError

     at java.base/java.util.concurrent.ForkJoinTask.get(ForkJoinTask.java:1006)

     at fr.univ_nantes.codex_processor.gui.DatasetExtendedDepthOfFieldProcessor.run(DatasetExtendedDepthOfFieldProcessor.java:69)

     at fr.univ_nantes.codex_processor.gui.MainController$1.handle(MainController.java:96)

     at fr.univ_nantes.codex_processor.gui.MainController$1.handle(MainController.java:64)

     at com.sun.javafx.event.CompositeEventHandler.dispatchBubblingEvent(CompositeEventHandler.java:86)

     at com.sun.javafx.event.EventHandlerManager.dispatchBubblingEvent(EventHandlerManager.java:234)

     at com.sun.javafx.event.EventHandlerManager.dispatchBubblingEvent(EventHandlerManager.java:191)

     at com.sun.javafx.event.CompositeEventDispatcher.dispatchBubblingEvent(CompositeEventDispatcher.java:59)

     at com.sun.javafx.event.BasicEventDispatcher.dispatchEvent(BasicEventDispatcher.java:58)

     at com.sun.javafx.event.EventDispatchChainImpl.dispatchEvent(EventDispatchChainImpl.java:114)

     at com.sun.javafx.event.BasicEventDispatcher.dispatchEvent(BasicEventDispatcher.java:56)

     at com.sun.javafx.event.EventDispatchChainImpl.dispatchEvent(EventDispatchChainImpl.java:114)

     at com.sun.javafx.event.BasicEventDispatcher.dispatchEvent(BasicEventDispatcher.java:56)

     at com.sun.javafx.event.EventDispatchChainImpl.dispatchEvent(EventDispatchChainImpl.java:114)

     at com.sun.javafx.event.EventUtil.fireEventImpl(EventUtil.java:74)

     at com.sun.javafx.event.EventUtil.fireEvent(EventUtil.java:49)

     at javafx.event.Event.fireEvent(Event.java:198)

     at javafx.scene.Node.fireEvent(Node.java:8889)

     at javafx.scene.control.Button.fire(Button.java:203)

     at com.sun.javafx.scene.control.behavior.ButtonBehavior.mouseReleased(ButtonBehavior.java:208)

     at com.sun.javafx.scene.control.inputmap.InputMap.handle(InputMap.java:274)

     at com.sun.javafx.event.CompositeEventHandler$NormalEventHandlerRecord.handleBubblingEvent(CompositeEventHandler.java:247)

     at com.sun.javafx.event.CompositeEventHandler.dispatchBubblingEvent(CompositeEventHandler.java:80)

     at com.sun.javafx.event.EventHandlerManager.dispatchBubblingEvent(EventHandlerManager.java:234)

     at com.sun.javafx.event.EventHandlerManager.dispatchBubblingEvent(EventHandlerManager.java:191)

     at com.sun.javafx.event.CompositeEventDispatcher.dispatchBubblingEvent(CompositeEventDispatcher.java:59)

     at com.sun.javafx.event.BasicEventDispatcher.dispatchEvent(BasicEventDispatcher.java:58)

     at com.sun.javafx.event.EventDispatchChainImpl.dispatchEvent(EventDispatchChainImpl.java:114)

     at com.sun.javafx.event.BasicEventDispatcher.dispatchEvent(BasicEventDispatcher.java:56)

     at com.sun.javafx.event.EventDispatchChainImpl.dispatchEvent(EventDispatchChainImpl.java:114)

     at com.sun.javafx.event.BasicEventDispatcher.dispatchEvent(BasicEventDispatcher.java:56)

     at com.sun.javafx.event.EventDispatchChainImpl.dispatchEvent(EventDispatchChainImpl.java:114)

     at com.sun.javafx.event.EventUtil.fireEventImpl(EventUtil.java:74)

     at com.sun.javafx.event.EventUtil.fireEvent(EventUtil.java:54)

     at javafx.event.Event.fireEvent(Event.java:198)

     at javafx.scene.Scene$MouseHandler.process(Scene.java:3856)

     at javafx.scene.Scene.processMouseEvent(Scene.java:1851)

     at javafx.scene.Scene$ScenePeerListener.mouseEvent(Scene.java:2584)

     at com.sun.javafx.tk.quantum.GlassViewEventHandler$MouseEventNotification.run(GlassViewEventHandler.java:409)

     at com.sun.javafx.tk.quantum.GlassViewEventHandler$MouseEventNotification.run(GlassViewEventHandler.java:299)

     at java.base/java.security.AccessController.doPrivileged(Native Method)

     at com.sun.javafx.tk.quantum.GlassViewEventHandler.lambda$handleMouseEvent$2(GlassViewEventHandler.java:447)

     at com.sun.javafx.tk.quantum.QuantumToolkit.runWithoutRenderLock(QuantumToolkit.java:412)

     at com.sun.javafx.tk.quantum.GlassViewEventHandler.handleMouseEvent(GlassViewEventHandler.java:446)

     at com.sun.glass.ui.View.handleMouseEvent(View.java:556)

     at com.sun.glass.ui.View.notifyMouse(View.java:942)

     at com.sun.glass.ui.win.WinApplication._runLoop(Native Method)

     at com.sun.glass.ui.win.WinApplication.lambda$runLoop$3(WinApplication.java:174)

     at java.base/java.lang.Thread.run(Thread.java:829)

 Caused by: java.lang.OutOfMemoryError

     at java.base/jdk.internal.reflect.GeneratedConstructorAccessor3.newInstance(Unknown Source)

     at java.base/jdk.internal.reflect.DelegatingConstructorAccessorImpl.newInstance(DelegatingConstructorAccessorImpl.java:45)

     at java.base/java.lang.reflect.Constructor.newInstance(Constructor.java:490)

     at java.base/java.util.concurrent.ForkJoinTask.getThrowableException(ForkJoinTask.java:603)

     ... 49 more

 Caused by: java.lang.OutOfMemoryError: Java heap space

 java.util.concurrent.ExecutionException: java.lang.OutOfMemoryError

     at java.base/java.util.concurrent.ForkJoinTask.get(ForkJoinTask.java:1006)

     at fr.univ_nantes.codex_processor.gui.DatasetExtendedDepthOfFieldProcessor.run(DatasetExtendedDepthOfFieldProcessor.java:69)

     at fr.univ_nantes.codex_processor.gui.MainController$1.handle(MainController.java:96)

     at fr.univ_nantes.codex_processor.gui.MainController$1.handle(MainController.java:64)

     at com.sun.javafx.event.CompositeEventHandler.dispatchBubblingEvent(CompositeEventHandler.java:86)

     at com.sun.javafx.event.EventHandlerManager.dispatchBubblingEvent(EventHandlerManager.java:234)

     at com.sun.javafx.event.EventHandlerManager.dispatchBubblingEvent(EventHandlerManager.java:191)

     at com.sun.javafx.event.CompositeEventDispatcher.dispatchBubblingEvent(CompositeEventDispatcher.java:59)

     at com.sun.javafx.event.BasicEventDispatcher.dispatchEvent(BasicEventDispatcher.java:58)

     at com.sun.javafx.event.EventDispatchChainImpl.dispatchEvent(EventDispatchChainImpl.java:114)

     at com.sun.javafx.event.BasicEventDispatcher.dispatchEvent(BasicEventDispatcher.java:56)

     at com.sun.javafx.event.EventDispatchChainImpl.dispatchEvent(EventDispatchChainImpl.java:114)

     at com.sun.javafx.event.BasicEventDispatcher.dispatchEvent(BasicEventDispatcher.java:56)

     at com.sun.javafx.event.EventDispatchChainImpl.dispatchEvent(EventDispatchChainImpl.java:114)

     at com.sun.javafx.event.EventUtil.fireEventImpl(EventUtil.java:74)

     at com.sun.javafx.event.EventUtil.fireEvent(EventUtil.java:49)

     at javafx.event.Event.fireEvent(Event.java:198)

     at javafx.scene.Node.fireEvent(Node.java:8889)

     at javafx.scene.control.Button.fire(Button.java:203)

     at com.sun.javafx.scene.control.behavior.ButtonBehavior.mouseReleased(ButtonBehavior.java:208)

     at com.sun.javafx.scene.control.inputmap.InputMap.handle(InputMap.java:274)

     at com.sun.javafx.event.CompositeEventHandler$NormalEventHandlerRecord.handleBubblingEvent(CompositeEventHandler.java:247)

     at com.sun.javafx.event.CompositeEventHandler.dispatchBubblingEvent(CompositeEventHandler.java:80)

     at com.sun.javafx.event.EventHandlerManager.dispatchBubblingEvent(EventHandlerManager.java:234)

     at com.sun.javafx.event.EventHandlerManager.dispatchBubblingEvent(EventHandlerManager.java:191)

     at com.sun.javafx.event.CompositeEventDispatcher.dispatchBubblingEvent(CompositeEventDispatcher.java:59)

     at com.sun.javafx.event.BasicEventDispatcher.dispatchEvent(BasicEventDispatcher.java:58)

     at com.sun.javafx.event.EventDispatchChainImpl.dispatchEvent(EventDispatchChainImpl.java:114)

     at com.sun.javafx.event.BasicEventDispatcher.dispatchEvent(BasicEventDispatcher.java:56)

     at com.sun.javafx.event.EventDispatchChainImpl.dispatchEvent(EventDispatchChainImpl.java:114)

     at com.sun.javafx.event.BasicEventDispatcher.dispatchEvent(BasicEventDispatcher.java:56)

     at com.sun.javafx.event.EventDispatchChainImpl.dispatchEvent(EventDispatchChainImpl.java:114)

     at com.sun.javafx.event.EventUtil.fireEventImpl(EventUtil.java:74)

     at com.sun.javafx.event.EventUtil.fireEvent(EventUtil.java:54)

     at javafx.event.Event.fireEvent(Event.java:198)

     at javafx.scene.Scene$MouseHandler.process(Scene.java:3856)

     at javafx.scene.Scene.processMouseEvent(Scene.java:1851)

     at javafx.scene.Scene$ScenePeerListener.mouseEvent(Scene.java:2584)

     at com.sun.javafx.tk.quantum.GlassViewEventHandler$MouseEventNotification.run(GlassViewEventHandler.java:409)

     at com.sun.javafx.tk.quantum.GlassViewEventHandler$MouseEventNotification.run(GlassViewEventHandler.java:299)

     at java.base/java.security.AccessController.doPrivileged(Native Method)

     at com.sun.javafx.tk.quantum.GlassViewEventHandler.lambda$handleMouseEvent$2(GlassViewEventHandler.java:447)

     at com.sun.javafx.tk.quantum.QuantumToolkit.runWithoutRenderLock(QuantumToolkit.java:412)

     at com.sun.javafx.tk.quantum.GlassViewEventHandler.handleMouseEvent(GlassViewEventHandler.java:446)

     at com.sun.glass.ui.View.handleMouseEvent(View.java:556)

     at com.sun.glass.ui.View.notifyMouse(View.java:942)

     at com.sun.glass.ui.win.WinApplication._runLoop(Native Method)

     at com.sun.glass.ui.win.WinApplication.lambda$runLoop$3(WinApplication.java:174)

     at java.base/java.lang.Thread.run(Thread.java:829)

 Caused by: java.lang.OutOfMemoryError

     at java.base/jdk.internal.reflect.GeneratedConstructorAccessor3.newInstance(Unknown Source)

     at java.base/jdk.internal.reflect.DelegatingConstructorAccessorImpl.newInstance(DelegatingConstructorAccessorImpl.java:45)

     at java.base/java.lang.reflect.Constructor.newInstance(Constructor.java:490)

     at java.base/java.util.concurrent.ForkJoinTask.getThrowableException(ForkJoinTask.java:603)

     ... 49 more

 Caused by: java.lang.OutOfMemoryError: Java heap space

 java.util.concurrent.ExecutionException: java.lang.OutOfMemoryError

     at java.base/java.util.concurrent.ForkJoinTask.get(ForkJoinTask.java:1006)

     at fr.univ_nantes.codex_processor.gui.DatasetExtendedDepthOfFieldProcessor.run(DatasetExtendedDepthOfFieldProcessor.java:69)

     at fr.univ_nantes.codex_processor.gui.MainController$1.handle(MainController.java:96)

     at fr.univ_nantes.codex_processor.gui.MainController$1.handle(MainController.java:64)

     at com.sun.javafx.event.CompositeEventHandler.dispatchBubblingEvent(CompositeEventHandler.java:86)

     at com.sun.javafx.event.EventHandlerManager.dispatchBubblingEvent(EventHandlerManager.java:234)

     at com.sun.javafx.event.EventHandlerManager.dispatchBubblingEvent(EventHandlerManager.java:191)

     at com.sun.javafx.event.CompositeEventDispatcher.dispatchBubblingEvent(CompositeEventDispatcher.java:59)

     at com.sun.javafx.event.BasicEventDispatcher.dispatchEvent(BasicEventDispatcher.java:58)

     at com.sun.javafx.event.EventDispatchChainImpl.dispatchEvent(EventDispatchChainImpl.java:114)

     at com.sun.javafx.event.BasicEventDispatcher.dispatchEvent(BasicEventDispatcher.java:56)

     at com.sun.javafx.event.EventDispatchChainImpl.dispatchEvent(EventDispatchChainImpl.java:114)

     at com.sun.javafx.event.BasicEventDispatcher.dispatchEvent(BasicEventDispatcher.java:56)

     at com.sun.javafx.event.EventDispatchChainImpl.dispatchEvent(EventDispatchChainImpl.java:114)

     at com.sun.javafx.event.EventUtil.fireEventImpl(EventUtil.java:74)

     at com.sun.javafx.event.EventUtil.fireEvent(EventUtil.java:49)

     at javafx.event.Event.fireEvent(Event.java:198)

     at javafx.scene.Node.fireEvent(Node.java:8889)

     at javafx.scene.control.Button.fire(Button.java:203)

     at com.sun.javafx.scene.control.behavior.ButtonBehavior.mouseReleased(ButtonBehavior.java:208)

     at com.sun.javafx.scene.control.inputmap.InputMap.handle(InputMap.java:274)

     at com.sun.javafx.event.CompositeEventHandler$NormalEventHandlerRecord.handleBubblingEvent(CompositeEventHandler.java:247)

     at com.sun.javafx.event.CompositeEventHandler.dispatchBubblingEvent(CompositeEventHandler.java:80)

     at com.sun.javafx.event.EventHandlerManager.dispatchBubblingEvent(EventHandlerManager.java:234)

     at com.sun.javafx.event.EventHandlerManager.dispatchBubblingEvent(EventHandlerManager.java:191)

     at com.sun.javafx.event.CompositeEventDispatcher.dispatchBubblingEvent(CompositeEventDispatcher.java:59)

     at com.sun.javafx.event.BasicEventDispatcher.dispatchEvent(BasicEventDispatcher.java:58)

     at com.sun.javafx.event.EventDispatchChainImpl.dispatchEvent(EventDispatchChainImpl.java:114)

     at com.sun.javafx.event.BasicEventDispatcher.dispatchEvent(BasicEventDispatcher.java:56)

     at com.sun.javafx.event.EventDispatchChainImpl.dispatchEvent(EventDispatchChainImpl.java:114)

     at com.sun.javafx.event.BasicEventDispatcher.dispatchEvent(BasicEventDispatcher.java:56)

     at com.sun.javafx.event.EventDispatchChainImpl.dispatchEvent(EventDispatchChainImpl.java:114)

     at com.sun.javafx.event.EventUtil.fireEventImpl(EventUtil.java:74)

     at com.sun.javafx.event.EventUtil.fireEvent(EventUtil.java:54)

     at javafx.event.Event.fireEvent(Event.java:198)

     at javafx.scene.Scene$MouseHandler.process(Scene.java:3856)

     at javafx.scene.Scene.processMouseEvent(Scene.java:1851)

     at javafx.scene.Scene$ScenePeerListener.mouseEvent(Scene.java:2584)

     at com.sun.javafx.tk.quantum.GlassViewEventHandler$MouseEventNotification.run(GlassViewEventHandler.java:409)

     at com.sun.javafx.tk.quantum.GlassViewEventHandler$MouseEventNotification.run(GlassViewEventHandler.java:299)

     at java.base/java.security.AccessController.doPrivileged(Native Method)

     at com.sun.javafx.tk.quantum.GlassViewEventHandler.lambda$handleMouseEvent$2(GlassViewEventHandler.java:447)

     at com.sun.javafx.tk.quantum.QuantumToolkit.runWithoutRenderLock(QuantumToolkit.java:412)

     at com.sun.javafx.tk.quantum.GlassViewEventHandler.handleMouseEvent(GlassViewEventHandler.java:446)

     at com.sun.glass.ui.View.handleMouseEvent(View.java:556)

     at com.sun.glass.ui.View.notifyMouse(View.java:942)

     at com.sun.glass.ui.win.WinApplication._runLoop(Native Method)

     at com.sun.glass.ui.win.WinApplication.lambda$runLoop$3(WinApplication.java:174)

     at java.base/java.lang.Thread.run(Thread.java:829)

 Caused by: java.lang.OutOfMemoryError

     at java.base/jdk.internal.reflect.GeneratedConstructorAccessor3.newInstance(Unknown Source)

     at java.base/jdk.internal.reflect.DelegatingConstructorAccessorImpl.newInstance(DelegatingConstructorAccessorImpl.java:45)

     at java.base/java.lang.reflect.Constructor.newInstance(Constructor.java:490)

     at java.base/java.util.concurrent.ForkJoinTask.getThrowableException(ForkJoinTask.java:603)

     ... 49 more

 Caused by: java.lang.OutOfMemoryError: Java heap space

 java.util.concurrent.ExecutionException: java.lang.OutOfMemoryError

     at java.base/java.util.concurrent.ForkJoinTask.get(ForkJoinTask.java:1006)

     at fr.univ_nantes.codex_processor.gui.DatasetExtendedDepthOfFieldProcessor.run(DatasetExtendedDepthOfFieldProcessor.java:69)

     at fr.univ_nantes.codex_processor.gui.MainController$1.handle(MainController.java:96)

     at fr.univ_nantes.codex_processor.gui.MainController$1.handle(MainController.java:64)

     at com.sun.javafx.event.CompositeEventHandler.dispatchBubblingEvent(CompositeEventHandler.java:86)

     at com.sun.javafx.event.EventHandlerManager.dispatchBubblingEvent(EventHandlerManager.java:234)

     at com.sun.javafx.event.EventHandlerManager.dispatchBubblingEvent(EventHandlerManager.java:191)

     at com.sun.javafx.event.CompositeEventDispatcher.dispatchBubblingEvent(CompositeEventDispatcher.java:59)

     at com.sun.javafx.event.BasicEventDispatcher.dispatchEvent(BasicEventDispatcher.java:58)

     at com.sun.javafx.event.EventDispatchChainImpl.dispatchEvent(EventDispatchChainImpl.java:114)

     at com.sun.javafx.event.BasicEventDispatcher.dispatchEvent(BasicEventDispatcher.java:56)

     at com.sun.javafx.event.EventDispatchChainImpl.dispatchEvent(EventDispatchChainImpl.java:114)

     at com.sun.javafx.event.BasicEventDispatcher.dispatchEvent(BasicEventDispatcher.java:56)

     at com.sun.javafx.event.EventDispatchChainImpl.dispatchEvent(EventDispatchChainImpl.java:114)

     at com.sun.javafx.event.EventUtil.fireEventImpl(EventUtil.java:74)

     at com.sun.javafx.event.EventUtil.fireEvent(EventUtil.java:49)

     at javafx.event.Event.fireEvent(Event.java:198)

     at javafx.scene.Node.fireEvent(Node.java:8889)

     at javafx.scene.control.Button.fire(Button.java:203)

     at com.sun.javafx.scene.control.behavior.ButtonBehavior.mouseReleased(ButtonBehavior.java:208)

     at com.sun.javafx.scene.control.inputmap.InputMap.handle(InputMap.java:274)

     at com.sun.javafx.event.CompositeEventHandler$NormalEventHandlerRecord.handleBubblingEvent(CompositeEventHandler.java:247)

     at com.sun.javafx.event.CompositeEventHandler.dispatchBubblingEvent(CompositeEventHandler.java:80)

     at com.sun.javafx.event.EventHandlerManager.dispatchBubblingEvent(EventHandlerManager.java:234)

     at com.sun.javafx.event.EventHandlerManager.dispatchBubblingEvent(EventHandlerManager.java:191)

     at com.sun.javafx.event.CompositeEventDispatcher.dispatchBubblingEvent(CompositeEventDispatcher.java:59)

     at com.sun.javafx.event.BasicEventDispatcher.dispatchEvent(BasicEventDispatcher.java:58)

     at com.sun.javafx.event.EventDispatchChainImpl.dispatchEvent(EventDispatchChainImpl.java:114)

     at com.sun.javafx.event.BasicEventDispatcher.dispatchEvent(BasicEventDispatcher.java:56)

     at com.sun.javafx.event.EventDispatchChainImpl.dispatchEvent(EventDispatchChainImpl.java:114)

     at com.sun.javafx.event.BasicEventDispatcher.dispatchEvent(BasicEventDispatcher.java:56)

     at com.sun.javafx.event.EventDispatchChainImpl.dispatchEvent(EventDispatchChainImpl.java:114)

     at com.sun.javafx.event.EventUtil.fireEventImpl(EventUtil.java:74)

     at com.sun.javafx.event.EventUtil.fireEvent(EventUtil.java:54)

     at javafx.event.Event.fireEvent(Event.java:198)

     at javafx.scene.Scene$MouseHandler.process(Scene.java:3856)

     at javafx.scene.Scene.processMouseEvent(Scene.java:1851)

     at javafx.scene.Scene$ScenePeerListener.mouseEvent(Scene.java:2584)

     at com.sun.javafx.tk.quantum.GlassViewEventHandler$MouseEventNotification.run(GlassViewEventHandler.java:409)

     at com.sun.javafx.tk.quantum.GlassViewEventHandler$MouseEventNotification.run(GlassViewEventHandler.java:299)

     at java.base/java.security.AccessController.doPrivileged(Native Method)

     at com.sun.javafx.tk.quantum.GlassViewEventHandler.lambda$handleMouseEvent$2(GlassViewEventHandler.java:447)

     at com.sun.javafx.tk.quantum.QuantumToolkit.runWithoutRenderLock(QuantumToolkit.java:412)

     at com.sun.javafx.tk.quantum.GlassViewEventHandler.handleMouseEvent(GlassViewEventHandler.java:446)

     at com.sun.glass.ui.View.handleMouseEvent(View.java:556)

     at com.sun.glass.ui.View.notifyMouse(View.java:942)

     at com.sun.glass.ui.win.WinApplication._runLoop(Native Method)

     at com.sun.glass.ui.win.WinApplication.lambda$runLoop$3(WinApplication.java:174)

     at java.base/java.lang.Thread.run(Thread.java:829)

 Caused by: java.lang.OutOfMemoryError

     at java.base/jdk.internal.reflect.GeneratedConstructorAccessor3.newInstance(Unknown Source)

     at java.base/jdk.internal.reflect.DelegatingConstructorAccessorImpl.newInstance(DelegatingConstructorAccessorImpl.java:45)

     at java.base/java.lang.reflect.Constructor.newInstance(Constructor.java:490)

     at java.base/java.util.concurrent.ForkJoinTask.getThrowableException(ForkJoinTask.java:603)

     ... 49 more

 Caused by: java.lang.OutOfMemoryError: Java heap space

 java.util.concurrent.ExecutionException: java.lang.OutOfMemoryError

     at java.base/java.util.concurrent.ForkJoinTask.get(ForkJoinTask.java:1006)

     at fr.univ_nantes.codex_processor.gui.DatasetExtendedDepthOfFieldProcessor.run(DatasetExtendedDepthOfFieldProcessor.java:69)

     at fr.univ_nantes.codex_processor.gui.MainController$1.handle(MainController.java:96)

     at fr.univ_nantes.codex_processor.gui.MainController$1.handle(MainController.java:64)

     at com.sun.javafx.event.CompositeEventHandler.dispatchBubblingEvent(CompositeEventHandler.java:86)

     at com.sun.javafx.event.EventHandlerManager.dispatchBubblingEvent(EventHandlerManager.java:234)

     at com.sun.javafx.event.EventHandlerManager.dispatchBubblingEvent(EventHandlerManager.java:191)

     at com.sun.javafx.event.CompositeEventDispatcher.dispatchBubblingEvent(CompositeEventDispatcher.java:59)

     at com.sun.javafx.event.BasicEventDispatcher.dispatchEvent(BasicEventDispatcher.java:58)

     at com.sun.javafx.event.EventDispatchChainImpl.dispatchEvent(EventDispatchChainImpl.java:114)

     at com.sun.javafx.event.BasicEventDispatcher.dispatchEvent(BasicEventDispatcher.java:56)

     at com.sun.javafx.event.EventDispatchChainImpl.dispatchEvent(EventDispatchChainImpl.java:114)

     at com.sun.javafx.event.BasicEventDispatcher.dispatchEvent(BasicEventDispatcher.java:56)

     at com.sun.javafx.event.EventDispatchChainImpl.dispatchEvent(EventDispatchChainImpl.java:114)

     at com.sun.javafx.event.EventUtil.fireEventImpl(EventUtil.java:74)

     at com.sun.javafx.event.EventUtil.fireEvent(EventUtil.java:49)

     at javafx.event.Event.fireEvent(Event.java:198)

     at javafx.scene.Node.fireEvent(Node.java:8889)

     at javafx.scene.control.Button.fire(Button.java:203)

     at com.sun.javafx.scene.control.behavior.ButtonBehavior.mouseReleased(ButtonBehavior.java:208)

     at com.sun.javafx.scene.control.inputmap.InputMap.handle(InputMap.java:274)

     at com.sun.javafx.event.CompositeEventHandler$NormalEventHandlerRecord.handleBubblingEvent(CompositeEventHandler.java:247)

     at com.sun.javafx.event.CompositeEventHandler.dispatchBubblingEvent(CompositeEventHandler.java:80)

     at com.sun.javafx.event.EventHandlerManager.dispatchBubblingEvent(EventHandlerManager.java:234)

     at com.sun.javafx.event.EventHandlerManager.dispatchBubblingEvent(EventHandlerManager.java:191)

     at com.sun.javafx.event.CompositeEventDispatcher.dispatchBubblingEvent(CompositeEventDispatcher.java:59)

     at com.sun.javafx.event.BasicEventDispatcher.dispatchEvent(BasicEventDispatcher.java:58)

     at com.sun.javafx.event.EventDispatchChainImpl.dispatchEvent(EventDispatchChainImpl.java:114)

     at com.sun.javafx.event.BasicEventDispatcher.dispatchEvent(BasicEventDispatcher.java:56)

     at com.sun.javafx.event.EventDispatchChainImpl.dispatchEvent(EventDispatchChainImpl.java:114)

     at com.sun.javafx.event.BasicEventDispatcher.dispatchEvent(BasicEventDispatcher.java:56)

     at com.sun.javafx.event.EventDispatchChainImpl.dispatchEvent(EventDispatchChainImpl.java:114)

     at com.sun.javafx.event.EventUtil.fireEventImpl(EventUtil.java:74)

     at com.sun.javafx.event.EventUtil.fireEvent(EventUtil.java:54)

     at javafx.event.Event.fireEvent(Event.java:198)

     at javafx.scene.Scene$MouseHandler.process(Scene.java:3856)

     at javafx.scene.Scene.processMouseEvent(Scene.java:1851)

     at javafx.scene.Scene$ScenePeerListener.mouseEvent(Scene.java:2584)

     at com.sun.javafx.tk.quantum.GlassViewEventHandler$MouseEventNotification.run(GlassViewEventHandler.java:409)

     at com.sun.javafx.tk.quantum.GlassViewEventHandler$MouseEventNotification.run(GlassViewEventHandler.java:299)

     at java.base/java.security.AccessController.doPrivileged(Native Method)

     at com.sun.javafx.tk.quantum.GlassViewEventHandler.lambda$handleMouseEvent$2(GlassViewEventHandler.java:447)

     at com.sun.javafx.tk.quantum.QuantumToolkit.runWithoutRenderLock(QuantumToolkit.java:412)

     at com.sun.javafx.tk.quantum.GlassViewEventHandler.handleMouseEvent(GlassViewEventHandler.java:446)

     at com.sun.glass.ui.View.handleMouseEvent(View.java:556)

     at com.sun.glass.ui.View.notifyMouse(View.java:942)

     at com.sun.glass.ui.win.WinApplication._runLoop(Native Method)

     at com.sun.glass.ui.win.WinApplication.lambda$runLoop$3(WinApplication.java:174)

     at java.base/java.lang.Thread.run(Thread.java:829)

 Caused by: java.lang.OutOfMemoryError

     at java.base/jdk.internal.reflect.GeneratedConstructorAccessor3.newInstance(Unknown Source)

     at java.base/jdk.internal.reflect.DelegatingConstructorAccessorImpl.newInstance(DelegatingConstructorAccessorImpl.java:45)

     at java.base/java.lang.reflect.Constructor.newInstance(Constructor.java:490)

     at java.base/java.util.concurrent.ForkJoinTask.getThrowableException(ForkJoinTask.java:603)

     ... 49 more

 Caused by: java.lang.OutOfMemoryError: Java heap space

 java.util.concurrent.ExecutionException: java.lang.OutOfMemoryError

     at java.base/java.util.concurrent.ForkJoinTask.get(ForkJoinTask.java:1006)

     at fr.univ_nantes.codex_processor.gui.DatasetExtendedDepthOfFieldProcessor.run(DatasetExtendedDepthOfFieldProcessor.java:69)

     at fr.univ_nantes.codex_processor.gui.MainController$1.handle(MainController.java:96)

     at fr.univ_nantes.codex_processor.gui.MainController$1.handle(MainController.java:64)

     at com.sun.javafx.event.CompositeEventHandler.dispatchBubblingEvent(CompositeEventHandler.java:86)

     at com.sun.javafx.event.EventHandlerManager.dispatchBubblingEvent(EventHandlerManager.java:234)

     at com.sun.javafx.event.EventHandlerManager.dispatchBubblingEvent(EventHandlerManager.java:191)

     at com.sun.javafx.event.CompositeEventDispatcher.dispatchBubblingEvent(CompositeEventDispatcher.java:59)

     at com.sun.javafx.event.BasicEventDispatcher.dispatchEvent(BasicEventDispatcher.java:58)

     at com.sun.javafx.event.EventDispatchChainImpl.dispatchEvent(EventDispatchChainImpl.java:114)

     at com.sun.javafx.event.BasicEventDispatcher.dispatchEvent(BasicEventDispatcher.java:56)

     at com.sun.javafx.event.EventDispatchChainImpl.dispatchEvent(EventDispatchChainImpl.java:114)

     at com.sun.javafx.event.BasicEventDispatcher.dispatchEvent(BasicEventDispatcher.java:56)

     at com.sun.javafx.event.EventDispatchChainImpl.dispatchEvent(EventDispatchChainImpl.java:114)

     at com.sun.javafx.event.EventUtil.fireEventImpl(EventUtil.java:74)

     at com.sun.javafx.event.EventUtil.fireEvent(EventUtil.java:49)

     at javafx.event.Event.fireEvent(Event.java:198)

     at javafx.scene.Node.fireEvent(Node.java:8889)

     at javafx.scene.control.Button.fire(Button.java:203)

     at com.sun.javafx.scene.control.behavior.ButtonBehavior.mouseReleased(ButtonBehavior.java:208)

     at com.sun.javafx.scene.control.inputmap.InputMap.handle(InputMap.java:274)

     at com.sun.javafx.event.CompositeEventHandler$NormalEventHandlerRecord.handleBubblingEvent(CompositeEventHandler.java:247)

     at com.sun.javafx.event.CompositeEventHandler.dispatchBubblingEvent(CompositeEventHandler.java:80)

     at com.sun.javafx.event.EventHandlerManager.dispatchBubblingEvent(EventHandlerManager.java:234)

     at com.sun.javafx.event.EventHandlerManager.dispatchBubblingEvent(EventHandlerManager.java:191)

     at com.sun.javafx.event.CompositeEventDispatcher.dispatchBubblingEvent(CompositeEventDispatcher.java:59)

     at com.sun.javafx.event.BasicEventDispatcher.dispatchEvent(BasicEventDispatcher.java:58)

     at com.sun.javafx.event.EventDispatchChainImpl.dispatchEvent(EventDispatchChainImpl.java:114)

     at com.sun.javafx.event.BasicEventDispatcher.dispatchEvent(BasicEventDispatcher.java:56)

     at com.sun.javafx.event.EventDispatchChainImpl.dispatchEvent(EventDispatchChainImpl.java:114)

     at com.sun.javafx.event.BasicEventDispatcher.dispatchEvent(BasicEventDispatcher.java:56)

     at com.sun.javafx.event.EventDispatchChainImpl.dispatchEvent(EventDispatchChainImpl.java:114)

     at com.sun.javafx.event.EventUtil.fireEventImpl(EventUtil.java:74)

     at com.sun.javafx.event.EventUtil.fireEvent(EventUtil.java:54)

     at javafx.event.Event.fireEvent(Event.java:198)

     at javafx.scene.Scene$MouseHandler.process(Scene.java:3856)

     at javafx.scene.Scene.processMouseEvent(Scene.java:1851)

     at javafx.scene.Scene$ScenePeerListener.mouseEvent(Scene.java:2584)

     at com.sun.javafx.tk.quantum.GlassViewEventHandler$MouseEventNotification.run(GlassViewEventHandler.java:409)

     at com.sun.javafx.tk.quantum.GlassViewEventHandler$MouseEventNotification.run(GlassViewEventHandler.java:299)

     at java.base/java.security.AccessController.doPrivileged(Native Method)

     at com.sun.javafx.tk.quantum.GlassViewEventHandler.lambda$handleMouseEvent$2(GlassViewEventHandler.java:447)

     at com.sun.javafx.tk.quantum.QuantumToolkit.runWithoutRenderLock(QuantumToolkit.java:412)

     at com.sun.javafx.tk.quantum.GlassViewEventHandler.handleMouseEvent(GlassViewEventHandler.java:446)

     at com.sun.glass.ui.View.handleMouseEvent(View.java:556)

     at com.sun.glass.ui.View.notifyMouse(View.java:942)

     at com.sun.glass.ui.win.WinApplication._runLoop(Native Method)

     at com.sun.glass.ui.win.WinApplication.lambda$runLoop$3(WinApplication.java:174)

     at java.base/java.lang.Thread.run(Thread.java:829)

 Caused by: java.lang.OutOfMemoryError

     at java.base/jdk.internal.reflect.GeneratedConstructorAccessor3.newInstance(Unknown Source)

     at java.base/jdk.internal.reflect.DelegatingConstructorAccessorImpl.newInstance(DelegatingConstructorAccessorImpl.java:45)

     at java.base/java.lang.reflect.Constructor.newInstance(Constructor.java:490)

     at java.base/java.util.concurrent.ForkJoinTask.getThrowableException(ForkJoinTask.java:603)

     ... 49 more

 Caused by: java.lang.OutOfMemoryError: Java heap space

 java.util.concurrent.ExecutionException: java.lang.OutOfMemoryError

     at java.base/java.util.concurrent.ForkJoinTask.get(ForkJoinTask.java:1006)

     at fr.univ_nantes.codex_processor.gui.DatasetExtendedDepthOfFieldProcessor.run(DatasetExtendedDepthOfFieldProcessor.java:69)

     at fr.univ_nantes.codex_processor.gui.MainController$1.handle(MainController.java:96)

     at fr.univ_nantes.codex_processor.gui.MainController$1.handle(MainController.java:64)

     at com.sun.javafx.event.CompositeEventHandler.dispatchBubblingEvent(CompositeEventHandler.java:86)

     at com.sun.javafx.event.EventHandlerManager.dispatchBubblingEvent(EventHandlerManager.java:234)

     at com.sun.javafx.event.EventHandlerManager.dispatchBubblingEvent(EventHandlerManager.java:191)

     at com.sun.javafx.event.CompositeEventDispatcher.dispatchBubblingEvent(CompositeEventDispatcher.java:59)

     at com.sun.javafx.event.BasicEventDispatcher.dispatchEvent(BasicEventDispatcher.java:58)

     at com.sun.javafx.event.EventDispatchChainImpl.dispatchEvent(EventDispatchChainImpl.java:114)

     at com.sun.javafx.event.BasicEventDispatcher.dispatchEvent(BasicEventDispatcher.java:56)

     at com.sun.javafx.event.EventDispatchChainImpl.dispatchEvent(EventDispatchChainImpl.java:114)

     at com.sun.javafx.event.BasicEventDispatcher.dispatchEvent(BasicEventDispatcher.java:56)

     at com.sun.javafx.event.EventDispatchChainImpl.dispatchEvent(EventDispatchChainImpl.java:114)

     at com.sun.javafx.event.EventUtil.fireEventImpl(EventUtil.java:74)

     at com.sun.javafx.event.EventUtil.fireEvent(EventUtil.java:49)

     at javafx.event.Event.fireEvent(Event.java:198)

     at javafx.scene.Node.fireEvent(Node.java:8889)

     at javafx.scene.control.Button.fire(Button.java:203)

     at com.sun.javafx.scene.control.behavior.ButtonBehavior.mouseReleased(ButtonBehavior.java:208)

     at com.sun.javafx.scene.control.inputmap.InputMap.handle(InputMap.java:274)

     at com.sun.javafx.event.CompositeEventHandler$NormalEventHandlerRecord.handleBubblingEvent(CompositeEventHandler.java:247)

     at com.sun.javafx.event.CompositeEventHandler.dispatchBubblingEvent(CompositeEventHandler.java:80)

     at com.sun.javafx.event.EventHandlerManager.dispatchBubblingEvent(EventHandlerManager.java:234)

     at com.sun.javafx.event.EventHandlerManager.dispatchBubblingEvent(EventHandlerManager.java:191)

     at com.sun.javafx.event.CompositeEventDispatcher.dispatchBubblingEvent(CompositeEventDispatcher.java:59)

     at com.sun.javafx.event.BasicEventDispatcher.dispatchEvent(BasicEventDispatcher.java:58)

     at com.sun.javafx.event.EventDispatchChainImpl.dispatchEvent(EventDispatchChainImpl.java:114)

     at com.sun.javafx.event.BasicEventDispatcher.dispatchEvent(BasicEventDispatcher.java:56)

     at com.sun.javafx.event.EventDispatchChainImpl.dispatchEvent(EventDispatchChainImpl.java:114)

     at com.sun.javafx.event.BasicEventDispatcher.dispatchEvent(BasicEventDispatcher.java:56)

     at com.sun.javafx.event.EventDispatchChainImpl.dispatchEvent(EventDispatchChainImpl.java:114)

     at com.sun.javafx.event.EventUtil.fireEventImpl(EventUtil.java:74)

     at com.sun.javafx.event.EventUtil.fireEvent(EventUtil.java:54)

     at javafx.event.Event.fireEvent(Event.java:198)

     at javafx.scene.Scene$MouseHandler.process(Scene.java:3856)

     at javafx.scene.Scene.processMouseEvent(Scene.java:1851)

     at javafx.scene.Scene$ScenePeerListener.mouseEvent(Scene.java:2584)

     at com.sun.javafx.tk.quantum.GlassViewEventHandler$MouseEventNotification.run(GlassViewEventHandler.java:409)

     at com.sun.javafx.tk.quantum.GlassViewEventHandler$MouseEventNotification.run(GlassViewEventHandler.java:299)

     at java.base/java.security.AccessController.doPrivileged(Native Method)

     at com.sun.javafx.tk.quantum.GlassViewEventHandler.lambda$handleMouseEvent$2(GlassViewEventHandler.java:447)

     at com.sun.javafx.tk.quantum.QuantumToolkit.runWithoutRenderLock(QuantumToolkit.java:412)

     at com.sun.javafx.tk.quantum.GlassViewEventHandler.handleMouseEvent(GlassViewEventHandler.java:446)

     at com.sun.glass.ui.View.handleMouseEvent(View.java:556)

     at com.sun.glass.ui.View.notifyMouse(View.java:942)

     at com.sun.glass.ui.win.WinApplication._runLoop(Native Method)

     at com.sun.glass.ui.win.WinApplication.lambda$runLoop$3(WinApplication.java:174)

     at java.base/java.lang.Thread.run(Thread.java:829)

 Caused by: java.lang.OutOfMemoryError

     at java.base/jdk.internal.reflect.GeneratedConstructorAccessor3.newInstance(Unknown Source)

     at java.base/jdk.internal.reflect.DelegatingConstructorAccessorImpl.newInstance(DelegatingConstructorAccessorImpl.java:45)

     at java.base/java.lang.reflect.Constructor.newInstance(Constructor.java:490)

     at java.base/java.util.concurrent.ForkJoinTask.getThrowableException(ForkJoinTask.java:603)

     ... 49 more

 Caused by: java.lang.OutOfMemoryError: Java heap space

 java.util.concurrent.ExecutionException: java.lang.OutOfMemoryError

     at java.base/java.util.concurrent.ForkJoinTask.get(ForkJoinTask.java:1006)

     at fr.univ_nantes.codex_processor.gui.DatasetExtendedDepthOfFieldProcessor.run(DatasetExtendedDepthOfFieldProcessor.java:69)

     at fr.univ_nantes.codex_processor.gui.MainController$1.handle(MainController.java:96)

     at fr.univ_nantes.codex_processor.gui.MainController$1.handle(MainController.java:64)

     at com.sun.javafx.event.CompositeEventHandler.dispatchBubblingEvent(CompositeEventHandler.java:86)

     at com.sun.javafx.event.EventHandlerManager.dispatchBubblingEvent(EventHandlerManager.java:234)

     at com.sun.javafx.event.EventHandlerManager.dispatchBubblingEvent(EventHandlerManager.java:191)

     at com.sun.javafx.event.CompositeEventDispatcher.dispatchBubblingEvent(CompositeEventDispatcher.java:59)

     at com.sun.javafx.event.BasicEventDispatcher.dispatchEvent(BasicEventDispatcher.java:58)

     at com.sun.javafx.event.EventDispatchChainImpl.dispatchEvent(EventDispatchChainImpl.java:114)

     at com.sun.javafx.event.BasicEventDispatcher.dispatchEvent(BasicEventDispatcher.java:56)

     at com.sun.javafx.event.EventDispatchChainImpl.dispatchEvent(EventDispatchChainImpl.java:114)

     at com.sun.javafx.event.BasicEventDispatcher.dispatchEvent(BasicEventDispatcher.java:56)

     at com.sun.javafx.event.EventDispatchChainImpl.dispatchEvent(EventDispatchChainImpl.java:114)

     at com.sun.javafx.event.EventUtil.fireEventImpl(EventUtil.java:74)

     at com.sun.javafx.event.EventUtil.fireEvent(EventUtil.java:49)

     at javafx.event.Event.fireEvent(Event.java:198)

     at javafx.scene.Node.fireEvent(Node.java:8889)

     at javafx.scene.control.Button.fire(Button.java:203)

     at com.sun.javafx.scene.control.behavior.ButtonBehavior.mouseReleased(ButtonBehavior.java:208)

     at com.sun.javafx.scene.control.inputmap.InputMap.handle(InputMap.java:274)

     at com.sun.javafx.event.CompositeEventHandler$NormalEventHandlerRecord.handleBubblingEvent(CompositeEventHandler.java:247)

     at com.sun.javafx.event.CompositeEventHandler.dispatchBubblingEvent(CompositeEventHandler.java:80)

     at com.sun.javafx.event.EventHandlerManager.dispatchBubblingEvent(EventHandlerManager.java:234)

     at com.sun.javafx.event.EventHandlerManager.dispatchBubblingEvent(EventHandlerManager.java:191)

     at com.sun.javafx.event.CompositeEventDispatcher.dispatchBubblingEvent(CompositeEventDispatcher.java:59)

     at com.sun.javafx.event.BasicEventDispatcher.dispatchEvent(BasicEventDispatcher.java:58)

     at com.sun.javafx.event.EventDispatchChainImpl.dispatchEvent(EventDispatchChainImpl.java:114)

     at com.sun.javafx.event.BasicEventDispatcher.dispatchEvent(BasicEventDispatcher.java:56)

     at com.sun.javafx.event.EventDispatchChainImpl.dispatchEvent(EventDispatchChainImpl.java:114)

     at com.sun.javafx.event.BasicEventDispatcher.dispatchEvent(BasicEventDispatcher.java:56)

     at com.sun.javafx.event.EventDispatchChainImpl.dispatchEvent(EventDispatchChainImpl.java:114)

     at com.sun.javafx.event.EventUtil.fireEventImpl(EventUtil.java:74)

     at com.sun.javafx.event.EventUtil.fireEvent(EventUtil.java:54)

     at javafx.event.Event.fireEvent(Event.java:198)

     at javafx.scene.Scene$MouseHandler.process(Scene.java:3856)

     at javafx.scene.Scene.processMouseEvent(Scene.java:1851)

     at javafx.scene.Scene$ScenePeerListener.mouseEvent(Scene.java:2584)

     at com.sun.javafx.tk.quantum.GlassViewEventHandler$MouseEventNotification.run(GlassViewEventHandler.java:409)

     at com.sun.javafx.tk.quantum.GlassViewEventHandler$MouseEventNotification.run(GlassViewEventHandler.java:299)

     at java.base/java.security.AccessController.doPrivileged(Native Method)

     at com.sun.javafx.tk.quantum.GlassViewEventHandler.lambda$handleMouseEvent$2(GlassViewEventHandler.java:447)

     at com.sun.javafx.tk.quantum.QuantumToolkit.runWithoutRenderLock(QuantumToolkit.java:412)

     at com.sun.javafx.tk.quantum.GlassViewEventHandler.handleMouseEvent(GlassViewEventHandler.java:446)

     at com.sun.glass.ui.View.handleMouseEvent(View.java:556)

     at com.sun.glass.ui.View.notifyMouse(View.java:942)

     at com.sun.glass.ui.win.WinApplication._runLoop(Native Method)

     at com.sun.glass.ui.win.WinApplication.lambda$runLoop$3(WinApplication.java:174)

     at java.base/java.lang.Thread.run(Thread.java:829)

 Caused by: java.lang.OutOfMemoryError

     at java.base/jdk.internal.reflect.GeneratedConstructorAccessor3.newInstance(Unknown Source)

     at java.base/jdk.internal.reflect.DelegatingConstructorAccessorImpl.newInstance(DelegatingConstructorAccessorImpl.java:45)

     at java.base/java.lang.reflect.Constructor.newInstance(Constructor.java:490)

     at java.base/java.util.concurrent.ForkJoinTask.getThrowableException(ForkJoinTask.java:603)

     ... 49 more

 Caused by: java.lang.OutOfMemoryError: Java heap space

 java.util.concurrent.ExecutionException: java.lang.OutOfMemoryError

     at java.base/java.util.concurrent.ForkJoinTask.get(ForkJoinTask.java:1006)

     at fr.univ_nantes.codex_processor.gui.DatasetExtendedDepthOfFieldProcessor.run(DatasetExtendedDepthOfFieldProcessor.java:69)

     at fr.univ_nantes.codex_processor.gui.MainController$1.handle(MainController.java:96)

     at fr.univ_nantes.codex_processor.gui.MainController$1.handle(MainController.java:64)

     at com.sun.javafx.event.CompositeEventHandler.dispatchBubblingEvent(CompositeEventHandler.java:86)

     at com.sun.javafx.event.EventHandlerManager.dispatchBubblingEvent(EventHandlerManager.java:234)

     at com.sun.javafx.event.EventHandlerManager.dispatchBubblingEvent(EventHandlerManager.java:191)

     at com.sun.javafx.event.CompositeEventDispatcher.dispatchBubblingEvent(CompositeEventDispatcher.java:59)

     at com.sun.javafx.event.BasicEventDispatcher.dispatchEvent(BasicEventDispatcher.java:58)

     at com.sun.javafx.event.EventDispatchChainImpl.dispatchEvent(EventDispatchChainImpl.java:114)

     at com.sun.javafx.event.BasicEventDispatcher.dispatchEvent(BasicEventDispatcher.java:56)

     at com.sun.javafx.event.EventDispatchChainImpl.dispatchEvent(EventDispatchChainImpl.java:114)

     at com.sun.javafx.event.BasicEventDispatcher.dispatchEvent(BasicEventDispatcher.java:56)

     at com.sun.javafx.event.EventDispatchChainImpl.dispatchEvent(EventDispatchChainImpl.java:114)

     at com.sun.javafx.event.EventUtil.fireEventImpl(EventUtil.java:74)

     at com.sun.javafx.event.EventUtil.fireEvent(EventUtil.java:49)

     at javafx.event.Event.fireEvent(Event.java:198)

     at javafx.scene.Node.fireEvent(Node.java:8889)

     at javafx.scene.control.Button.fire(Button.java:203)

     at com.sun.javafx.scene.control.behavior.ButtonBehavior.mouseReleased(ButtonBehavior.java:208)

     at com.sun.javafx.scene.control.inputmap.InputMap.handle(InputMap.java:274)

     at com.sun.javafx.event.CompositeEventHandler$NormalEventHandlerRecord.handleBubblingEvent(CompositeEventHandler.java:247)

     at com.sun.javafx.event.CompositeEventHandler.dispatchBubblingEvent(CompositeEventHandler.java:80)

     at com.sun.javafx.event.EventHandlerManager.dispatchBubblingEvent(EventHandlerManager.java:234)

     at com.sun.javafx.event.EventHandlerManager.dispatchBubblingEvent(EventHandlerManager.java:191)

     at com.sun.javafx.event.CompositeEventDispatcher.dispatchBubblingEvent(CompositeEventDispatcher.java:59)

     at com.sun.javafx.event.BasicEventDispatcher.dispatchEvent(BasicEventDispatcher.java:58)

     at com.sun.javafx.event.EventDispatchChainImpl.dispatchEvent(EventDispatchChainImpl.java:114)

     at com.sun.javafx.event.BasicEventDispatcher.dispatchEvent(BasicEventDispatcher.java:56)

     at com.sun.javafx.event.EventDispatchChainImpl.dispatchEvent(EventDispatchChainImpl.java:114)

     at com.sun.javafx.event.BasicEventDispatcher.dispatchEvent(BasicEventDispatcher.java:56)

     at com.sun.javafx.event.EventDispatchChainImpl.dispatchEvent(EventDispatchChainImpl.java:114)

     at com.sun.javafx.event.EventUtil.fireEventImpl(EventUtil.java:74)

     at com.sun.javafx.event.EventUtil.fireEvent(EventUtil.java:54)

     at javafx.event.Event.fireEvent(Event.java:198)

     at javafx.scene.Scene$MouseHandler.process(Scene.java:3856)

     at javafx.scene.Scene.processMouseEvent(Scene.java:1851)

     at javafx.scene.Scene$ScenePeerListener.mouseEvent(Scene.java:2584)

     at com.sun.javafx.tk.quantum.GlassViewEventHandler$MouseEventNotification.run(GlassViewEventHandler.java:409)

     at com.sun.javafx.tk.quantum.GlassViewEventHandler$MouseEventNotification.run(GlassViewEventHandler.java:299)

     at java.base/java.security.AccessController.doPrivileged(Native Method)

     at com.sun.javafx.tk.quantum.GlassViewEventHandler.lambda$handleMouseEvent$2(GlassViewEventHandler.java:447)

     at com.sun.javafx.tk.quantum.QuantumToolkit.runWithoutRenderLock(QuantumToolkit.java:412)

     at com.sun.javafx.tk.quantum.GlassViewEventHandler.handleMouseEvent(GlassViewEventHandler.java:446)

     at com.sun.glass.ui.View.handleMouseEvent(View.java:556)

     at com.sun.glass.ui.View.notifyMouse(View.java:942)

     at com.sun.glass.ui.win.WinApplication._runLoop(Native Method)

     at com.sun.glass.ui.win.WinApplication.lambda$runLoop$3(WinApplication.java:174)

     at java.base/java.lang.Thread.run(Thread.java:829)

 Caused by: java.lang.OutOfMemoryError

     at java.base/jdk.internal.reflect.GeneratedConstructorAccessor3.newInstance(Unknown Source)

     at java.base/jdk.internal.reflect.DelegatingConstructorAccessorImpl.newInstance(DelegatingConstructorAccessorImpl.java:45)

     at java.base/java.lang.reflect.Constructor.newInstance(Constructor.java:490)

     at java.base/java.util.concurrent.ForkJoinTask.getThrowableException(ForkJoinTask.java:603)

     ... 49 more

 Caused by: java.lang.OutOfMemoryError: Java heap space

 java.util.concurrent.ExecutionException: java.lang.OutOfMemoryError

     at java.base/java.util.concurrent.ForkJoinTask.get(ForkJoinTask.java:1006)

     at fr.univ_nantes.codex_processor.gui.DatasetExtendedDepthOfFieldProcessor.run(DatasetExtendedDepthOfFieldProcessor.java:69)

     at fr.univ_nantes.codex_processor.gui.MainController$1.handle(MainController.java:96)

     at fr.univ_nantes.codex_processor.gui.MainController$1.handle(MainController.java:64)

     at com.sun.javafx.event.CompositeEventHandler.dispatchBubblingEvent(CompositeEventHandler.java:86)

     at com.sun.javafx.event.EventHandlerManager.dispatchBubblingEvent(EventHandlerManager.java:234)

     at com.sun.javafx.event.EventHandlerManager.dispatchBubblingEvent(EventHandlerManager.java:191)

     at com.sun.javafx.event.CompositeEventDispatcher.dispatchBubblingEvent(CompositeEventDispatcher.java:59)

     at com.sun.javafx.event.BasicEventDispatcher.dispatchEvent(BasicEventDispatcher.java:58)

     at com.sun.javafx.event.EventDispatchChainImpl.dispatchEvent(EventDispatchChainImpl.java:114)

     at com.sun.javafx.event.BasicEventDispatcher.dispatchEvent(BasicEventDispatcher.java:56)

     at com.sun.javafx.event.EventDispatchChainImpl.dispatchEvent(EventDispatchChainImpl.java:114)

     at com.sun.javafx.event.BasicEventDispatcher.dispatchEvent(BasicEventDispatcher.java:56)

     at com.sun.javafx.event.EventDispatchChainImpl.dispatchEvent(EventDispatchChainImpl.java:114)

     at com.sun.javafx.event.EventUtil.fireEventImpl(EventUtil.java:74)

     at com.sun.javafx.event.EventUtil.fireEvent(EventUtil.java:49)

     at javafx.event.Event.fireEvent(Event.java:198)

     at javafx.scene.Node.fireEvent(Node.java:8889)

     at javafx.scene.control.Button.fire(Button.java:203)

     at com.sun.javafx.scene.control.behavior.ButtonBehavior.mouseReleased(ButtonBehavior.java:208)

     at com.sun.javafx.scene.control.inputmap.InputMap.handle(InputMap.java:274)

     at com.sun.javafx.event.CompositeEventHandler$NormalEventHandlerRecord.handleBubblingEvent(CompositeEventHandler.java:247)

     at com.sun.javafx.event.CompositeEventHandler.dispatchBubblingEvent(CompositeEventHandler.java:80)

     at com.sun.javafx.event.EventHandlerManager.dispatchBubblingEvent(EventHandlerManager.java:234)

     at com.sun.javafx.event.EventHandlerManager.dispatchBubblingEvent(EventHandlerManager.java:191)

     at com.sun.javafx.event.CompositeEventDispatcher.dispatchBubblingEvent(CompositeEventDispatcher.java:59)

     at com.sun.javafx.event.BasicEventDispatcher.dispatchEvent(BasicEventDispatcher.java:58)

     at com.sun.javafx.event.EventDispatchChainImpl.dispatchEvent(EventDispatchChainImpl.java:114)

     at com.sun.javafx.event.BasicEventDispatcher.dispatchEvent(BasicEventDispatcher.java:56)

     at com.sun.javafx.event.EventDispatchChainImpl.dispatchEvent(EventDispatchChainImpl.java:114)

     at com.sun.javafx.event.BasicEventDispatcher.dispatchEvent(BasicEventDispatcher.java:56)

     at com.sun.javafx.event.EventDispatchChainImpl.dispatchEvent(EventDispatchChainImpl.java:114)

     at com.sun.javafx.event.EventUtil.fireEventImpl(EventUtil.java:74)

     at com.sun.javafx.event.EventUtil.fireEvent(EventUtil.java:54)

     at javafx.event.Event.fireEvent(Event.java:198)

     at javafx.scene.Scene$MouseHandler.process(Scene.java:3856)

     at javafx.scene.Scene.processMouseEvent(Scene.java:1851)

     at javafx.scene.Scene$ScenePeerListener.mouseEvent(Scene.java:2584)

     at com.sun.javafx.tk.quantum.GlassViewEventHandler$MouseEventNotification.run(GlassViewEventHandler.java:409)

     at com.sun.javafx.tk.quantum.GlassViewEventHandler$MouseEventNotification.run(GlassViewEventHandler.java:299)

     at java.base/java.security.AccessController.doPrivileged(Native Method)

     at com.sun.javafx.tk.quantum.GlassViewEventHandler.lambda$handleMouseEvent$2(GlassViewEventHandler.java:447)

     at com.sun.javafx.tk.quantum.QuantumToolkit.runWithoutRenderLock(QuantumToolkit.java:412)

     at com.sun.javafx.tk.quantum.GlassViewEventHandler.handleMouseEvent(GlassViewEventHandler.java:446)

     at com.sun.glass.ui.View.handleMouseEvent(View.java:556)

     at com.sun.glass.ui.View.notifyMouse(View.java:942)

     at com.sun.glass.ui.win.WinApplication._runLoop(Native Method)

     at com.sun.glass.ui.win.WinApplication.lambda$runLoop$3(WinApplication.java:174)

     at java.base/java.lang.Thread.run(Thread.java:829)

 Caused by: java.lang.OutOfMemoryError

     at java.base/jdk.internal.reflect.GeneratedConstructorAccessor3.newInstance(Unknown Source)

     at java.base/jdk.internal.reflect.DelegatingConstructorAccessorImpl.newInstance(DelegatingConstructorAccessorImpl.java:45)

     at java.base/java.lang.reflect.Constructor.newInstance(Constructor.java:490)

     at java.base/java.util.concurrent.ForkJoinTask.getThrowableException(ForkJoinTask.java:603)

     ... 49 more

 Caused by: java.lang.OutOfMemoryError: Java heap space

 java.util.concurrent.ExecutionException: java.lang.OutOfMemoryError

     at java.base/java.util.concurrent.ForkJoinTask.get(ForkJoinTask.java:1006)

     at fr.univ_nantes.codex_processor.gui.DatasetExtendedDepthOfFieldProcessor.run(DatasetExtendedDepthOfFieldProcessor.java:69)

     at fr.univ_nantes.codex_processor.gui.MainController$1.handle(MainController.java:96)

     at fr.univ_nantes.codex_processor.gui.MainController$1.handle(MainController.java:64)

     at com.sun.javafx.event.CompositeEventHandler.dispatchBubblingEvent(CompositeEventHandler.java:86)

     at com.sun.javafx.event.EventHandlerManager.dispatchBubblingEvent(EventHandlerManager.java:234)

     at com.sun.javafx.event.EventHandlerManager.dispatchBubblingEvent(EventHandlerManager.java:191)

     at com.sun.javafx.event.CompositeEventDispatcher.dispatchBubblingEvent(CompositeEventDispatcher.java:59)

     at com.sun.javafx.event.BasicEventDispatcher.dispatchEvent(BasicEventDispatcher.java:58)

     at com.sun.javafx.event.EventDispatchChainImpl.dispatchEvent(EventDispatchChainImpl.java:114)

     at com.sun.javafx.event.BasicEventDispatcher.dispatchEvent(BasicEventDispatcher.java:56)

     at com.sun.javafx.event.EventDispatchChainImpl.dispatchEvent(EventDispatchChainImpl.java:114)

     at com.sun.javafx.event.BasicEventDispatcher.dispatchEvent(BasicEventDispatcher.java:56)

     at com.sun.javafx.event.EventDispatchChainImpl.dispatchEvent(EventDispatchChainImpl.java:114)

     at com.sun.javafx.event.EventUtil.fireEventImpl(EventUtil.java:74)

     at com.sun.javafx.event.EventUtil.fireEvent(EventUtil.java:49)

     at javafx.event.Event.fireEvent(Event.java:198)

     at javafx.scene.Node.fireEvent(Node.java:8889)

     at javafx.scene.control.Button.fire(Button.java:203)

     at com.sun.javafx.scene.control.behavior.ButtonBehavior.mouseReleased(ButtonBehavior.java:208)

     at com.sun.javafx.scene.control.inputmap.InputMap.handle(InputMap.java:274)

     at com.sun.javafx.event.CompositeEventHandler$NormalEventHandlerRecord.handleBubblingEvent(CompositeEventHandler.java:247)

     at com.sun.javafx.event.CompositeEventHandler.dispatchBubblingEvent(CompositeEventHandler.java:80)

     at com.sun.javafx.event.EventHandlerManager.dispatchBubblingEvent(EventHandlerManager.java:234)

     at com.sun.javafx.event.EventHandlerManager.dispatchBubblingEvent(EventHandlerManager.java:191)

     at com.sun.javafx.event.CompositeEventDispatcher.dispatchBubblingEvent(CompositeEventDispatcher.java:59)

     at com.sun.javafx.event.BasicEventDispatcher.dispatchEvent(BasicEventDispatcher.java:58)

     at com.sun.javafx.event.EventDispatchChainImpl.dispatchEvent(EventDispatchChainImpl.java:114)

     at com.sun.javafx.event.BasicEventDispatcher.dispatchEvent(BasicEventDispatcher.java:56)

     at com.sun.javafx.event.EventDispatchChainImpl.dispatchEvent(EventDispatchChainImpl.java:114)

     at com.sun.javafx.event.BasicEventDispatcher.dispatchEvent(BasicEventDispatcher.java:56)

     at com.sun.javafx.event.EventDispatchChainImpl.dispatchEvent(EventDispatchChainImpl.java:114)

     at com.sun.javafx.event.EventUtil.fireEventImpl(EventUtil.java:74)

     at com.sun.javafx.event.EventUtil.fireEvent(EventUtil.java:54)

     at javafx.event.Event.fireEvent(Event.java:198)

     at javafx.scene.Scene$MouseHandler.process(Scene.java:3856)

     at javafx.scene.Scene.processMouseEvent(Scene.java:1851)

     at javafx.scene.Scene$ScenePeerListener.mouseEvent(Scene.java:2584)

     at com.sun.javafx.tk.quantum.GlassViewEventHandler$MouseEventNotification.run(GlassViewEventHandler.java:409)

     at com.sun.javafx.tk.quantum.GlassViewEventHandler$MouseEventNotification.run(GlassViewEventHandler.java:299)

     at java.base/java.security.AccessController.doPrivileged(Native Method)

     at com.sun.javafx.tk.quantum.GlassViewEventHandler.lambda$handleMouseEvent$2(GlassViewEventHandler.java:447)

     at com.sun.javafx.tk.quantum.QuantumToolkit.runWithoutRenderLock(QuantumToolkit.java:412)

     at com.sun.javafx.tk.quantum.GlassViewEventHandler.handleMouseEvent(GlassViewEventHandler.java:446)

     at com.sun.glass.ui.View.handleMouseEvent(View.java:556)

     at com.sun.glass.ui.View.notifyMouse(View.java:942)

     at com.sun.glass.ui.win.WinApplication._runLoop(Native Method)

     at com.sun.glass.ui.win.WinApplication.lambda$runLoop$3(WinApplication.java:174)

     at java.base/java.lang.Thread.run(Thread.java:829)

 Caused by: java.lang.OutOfMemoryError

     at java.base/jdk.internal.reflect.GeneratedConstructorAccessor3.newInstance(Unknown Source)

     at java.base/jdk.internal.reflect.DelegatingConstructorAccessorImpl.newInstance(DelegatingConstructorAccessorImpl.java:45)

     at java.base/java.lang.reflect.Constructor.newInstance(Constructor.java:490)

     at java.base/java.util.concurrent.ForkJoinTask.getThrowableException(ForkJoinTask.java:603)

     ... 49 more

 Caused by: java.lang.OutOfMemoryError: Java heap space

 java.util.concurrent.ExecutionException: java.lang.OutOfMemoryError

     at java.base/java.util.concurrent.ForkJoinTask.get(ForkJoinTask.java:1006)

     at fr.univ_nantes.codex_processor.gui.DatasetExtendedDepthOfFieldProcessor.run(DatasetExtendedDepthOfFieldProcessor.java:69)

     at fr.univ_nantes.codex_processor.gui.MainController$1.handle(MainController.java:96)

     at fr.univ_nantes.codex_processor.gui.MainController$1.handle(MainController.java:64)

     at com.sun.javafx.event.CompositeEventHandler.dispatchBubblingEvent(CompositeEventHandler.java:86)

     at com.sun.javafx.event.EventHandlerManager.dispatchBubblingEvent(EventHandlerManager.java:234)

     at com.sun.javafx.event.EventHandlerManager.dispatchBubblingEvent(EventHandlerManager.java:191)

     at com.sun.javafx.event.CompositeEventDispatcher.dispatchBubblingEvent(CompositeEventDispatcher.java:59)

     at com.sun.javafx.event.BasicEventDispatcher.dispatchEvent(BasicEventDispatcher.java:58)

     at com.sun.javafx.event.EventDispatchChainImpl.dispatchEvent(EventDispatchChainImpl.java:114)

     at com.sun.javafx.event.BasicEventDispatcher.dispatchEvent(BasicEventDispatcher.java:56)

     at com.sun.javafx.event.EventDispatchChainImpl.dispatchEvent(EventDispatchChainImpl.java:114)

     at com.sun.javafx.event.BasicEventDispatcher.dispatchEvent(BasicEventDispatcher.java:56)

     at com.sun.javafx.event.EventDispatchChainImpl.dispatchEvent(EventDispatchChainImpl.java:114)

     at com.sun.javafx.event.EventUtil.fireEventImpl(EventUtil.java:74)

     at com.sun.javafx.event.EventUtil.fireEvent(EventUtil.java:49)

     at javafx.event.Event.fireEvent(Event.java:198)

     at javafx.scene.Node.fireEvent(Node.java:8889)

     at javafx.scene.control.Button.fire(Button.java:203)

     at com.sun.javafx.scene.control.behavior.ButtonBehavior.mouseReleased(ButtonBehavior.java:208)

     at com.sun.javafx.scene.control.inputmap.InputMap.handle(InputMap.java:274)

     at com.sun.javafx.event.CompositeEventHandler$NormalEventHandlerRecord.handleBubblingEvent(CompositeEventHandler.java:247)

     at com.sun.javafx.event.CompositeEventHandler.dispatchBubblingEvent(CompositeEventHandler.java:80)

     at com.sun.javafx.event.EventHandlerManager.dispatchBubblingEvent(EventHandlerManager.java:234)

     at com.sun.javafx.event.EventHandlerManager.dispatchBubblingEvent(EventHandlerManager.java:191)

     at com.sun.javafx.event.CompositeEventDispatcher.dispatchBubblingEvent(CompositeEventDispatcher.java:59)

     at com.sun.javafx.event.BasicEventDispatcher.dispatchEvent(BasicEventDispatcher.java:58)

     at com.sun.javafx.event.EventDispatchChainImpl.dispatchEvent(EventDispatchChainImpl.java:114)

     at com.sun.javafx.event.BasicEventDispatcher.dispatchEvent(BasicEventDispatcher.java:56)

     at com.sun.javafx.event.EventDispatchChainImpl.dispatchEvent(EventDispatchChainImpl.java:114)

     at com.sun.javafx.event.BasicEventDispatcher.dispatchEvent(BasicEventDispatcher.java:56)

     at com.sun.javafx.event.EventDispatchChainImpl.dispatchEvent(EventDispatchChainImpl.java:114)

     at com.sun.javafx.event.EventUtil.fireEventImpl(EventUtil.java:74)

     at com.sun.javafx.event.EventUtil.fireEvent(EventUtil.java:54)

     at javafx.event.Event.fireEvent(Event.java:198)

     at javafx.scene.Scene$MouseHandler.process(Scene.java:3856)

     at javafx.scene.Scene.processMouseEvent(Scene.java:1851)

     at javafx.scene.Scene$ScenePeerListener.mouseEvent(Scene.java:2584)

     at com.sun.javafx.tk.quantum.GlassViewEventHandler$MouseEventNotification.run(GlassViewEventHandler.java:409)

     at com.sun.javafx.tk.quantum.GlassViewEventHandler$MouseEventNotification.run(GlassViewEventHandler.java:299)

     at java.base/java.security.AccessController.doPrivileged(Native Method)

     at com.sun.javafx.tk.quantum.GlassViewEventHandler.lambda$handleMouseEvent$2(GlassViewEventHandler.java:447)

     at com.sun.javafx.tk.quantum.QuantumToolkit.runWithoutRenderLock(QuantumToolkit.java:412)

     at com.sun.javafx.tk.quantum.GlassViewEventHandler.handleMouseEvent(GlassViewEventHandler.java:446)

     at com.sun.glass.ui.View.handleMouseEvent(View.java:556)

     at com.sun.glass.ui.View.notifyMouse(View.java:942)

     at com.sun.glass.ui.win.WinApplication._runLoop(Native Method)

     at com.sun.glass.ui.win.WinApplication.lambda$runLoop$3(WinApplication.java:174)

     at java.base/java.lang.Thread.run(Thread.java:829)

 Caused by: java.lang.OutOfMemoryError

     at java.base/jdk.internal.reflect.GeneratedConstructorAccessor3.newInstance(Unknown Source)

     at java.base/jdk.internal.reflect.DelegatingConstructorAccessorImpl.newInstance(DelegatingConstructorAccessorImpl.java:45)

     at java.base/java.lang.reflect.Constructor.newInstance(Constructor.java:490)

     at java.base/java.util.concurrent.ForkJoinTask.getThrowableException(ForkJoinTask.java:603)

     ... 49 more

 Caused by: java.lang.OutOfMemoryError: Java heap space

 java.util.concurrent.ExecutionException: java.lang.OutOfMemoryError

     at java.base/java.util.concurrent.ForkJoinTask.get(ForkJoinTask.java:1006)

     at fr.univ_nantes.codex_processor.gui.DatasetExtendedDepthOfFieldProcessor.run(DatasetExtendedDepthOfFieldProcessor.java:69)

     at fr.univ_nantes.codex_processor.gui.MainController$1.handle(MainController.java:96)

     at fr.univ_nantes.codex_processor.gui.MainController$1.handle(MainController.java:64)

     at com.sun.javafx.event.CompositeEventHandler.dispatchBubblingEvent(CompositeEventHandler.java:86)

     at com.sun.javafx.event.EventHandlerManager.dispatchBubblingEvent(EventHandlerManager.java:234)

     at com.sun.javafx.event.EventHandlerManager.dispatchBubblingEvent(EventHandlerManager.java:191)

     at com.sun.javafx.event.CompositeEventDispatcher.dispatchBubblingEvent(CompositeEventDispatcher.java:59)

     at com.sun.javafx.event.BasicEventDispatcher.dispatchEvent(BasicEventDispatcher.java:58)

     at com.sun.javafx.event.EventDispatchChainImpl.dispatchEvent(EventDispatchChainImpl.java:114)

     at com.sun.javafx.event.BasicEventDispatcher.dispatchEvent(BasicEventDispatcher.java:56)

     at com.sun.javafx.event.EventDispatchChainImpl.dispatchEvent(EventDispatchChainImpl.java:114)

     at com.sun.javafx.event.BasicEventDispatcher.dispatchEvent(BasicEventDispatcher.java:56)

     at com.sun.javafx.event.EventDispatchChainImpl.dispatchEvent(EventDispatchChainImpl.java:114)

     at com.sun.javafx.event.EventUtil.fireEventImpl(EventUtil.java:74)

     at com.sun.javafx.event.EventUtil.fireEvent(EventUtil.java:49)

     at javafx.event.Event.fireEvent(Event.java:198)

     at javafx.scene.Node.fireEvent(Node.java:8889)

     at javafx.scene.control.Button.fire(Button.java:203)

     at com.sun.javafx.scene.control.behavior.ButtonBehavior.mouseReleased(ButtonBehavior.java:208)

     at com.sun.javafx.scene.control.inputmap.InputMap.handle(InputMap.java:274)

     at com.sun.javafx.event.CompositeEventHandler$NormalEventHandlerRecord.handleBubblingEvent(CompositeEventHandler.java:247)

     at com.sun.javafx.event.CompositeEventHandler.dispatchBubblingEvent(CompositeEventHandler.java:80)

     at com.sun.javafx.event.EventHandlerManager.dispatchBubblingEvent(EventHandlerManager.java:234)

     at com.sun.javafx.event.EventHandlerManager.dispatchBubblingEvent(EventHandlerManager.java:191)

     at com.sun.javafx.event.CompositeEventDispatcher.dispatchBubblingEvent(CompositeEventDispatcher.java:59)

     at com.sun.javafx.event.BasicEventDispatcher.dispatchEvent(BasicEventDispatcher.java:58)

     at com.sun.javafx.event.EventDispatchChainImpl.dispatchEvent(EventDispatchChainImpl.java:114)

     at com.sun.javafx.event.BasicEventDispatcher.dispatchEvent(BasicEventDispatcher.java:56)

     at com.sun.javafx.event.EventDispatchChainImpl.dispatchEvent(EventDispatchChainImpl.java:114)

     at com.sun.javafx.event.BasicEventDispatcher.dispatchEvent(BasicEventDispatcher.java:56)

     at com.sun.javafx.event.EventDispatchChainImpl.dispatchEvent(EventDispatchChainImpl.java:114)

     at com.sun.javafx.event.EventUtil.fireEventImpl(EventUtil.java:74)

     at com.sun.javafx.event.EventUtil.fireEvent(EventUtil.java:54)

     at javafx.event.Event.fireEvent(Event.java:198)

     at javafx.scene.Scene$MouseHandler.process(Scene.java:3856)

     at javafx.scene.Scene.processMouseEvent(Scene.java:1851)

     at javafx.scene.Scene$ScenePeerListener.mouseEvent(Scene.java:2584)

     at com.sun.javafx.tk.quantum.GlassViewEventHandler$MouseEventNotification.run(GlassViewEventHandler.java:409)

     at com.sun.javafx.tk.quantum.GlassViewEventHandler$MouseEventNotification.run(GlassViewEventHandler.java:299)

     at java.base/java.security.AccessController.doPrivileged(Native Method)

     at com.sun.javafx.tk.quantum.GlassViewEventHandler.lambda$handleMouseEvent$2(GlassViewEventHandler.java:447)

     at com.sun.javafx.tk.quantum.QuantumToolkit.runWithoutRenderLock(QuantumToolkit.java:412)

     at com.sun.javafx.tk.quantum.GlassViewEventHandler.handleMouseEvent(GlassViewEventHandler.java:446)

     at com.sun.glass.ui.View.handleMouseEvent(View.java:556)

     at com.sun.glass.ui.View.notifyMouse(View.java:942)

     at com.sun.glass.ui.win.WinApplication._runLoop(Native Method)

     at com.sun.glass.ui.win.WinApplication.lambda$runLoop$3(WinApplication.java:174)

     at java.base/java.lang.Thread.run(Thread.java:829)

 Caused by: java.lang.OutOfMemoryError

     at java.base/jdk.internal.reflect.GeneratedConstructorAccessor3.newInstance(Unknown Source)

     at java.base/jdk.internal.reflect.DelegatingConstructorAccessorImpl.newInstance(DelegatingConstructorAccessorImpl.java:45)

     at java.base/java.lang.reflect.Constructor.newInstance(Constructor.java:490)

     at java.base/java.util.concurrent.ForkJoinTask.getThrowableException(ForkJoinTask.java:603)

     ... 49 more

 Caused by: java.lang.OutOfMemoryError: Java heap space

 java.util.concurrent.ExecutionException: java.lang.OutOfMemoryError

     at java.base/java.util.concurrent.ForkJoinTask.get(ForkJoinTask.java:1006)

     at fr.univ_nantes.codex_processor.gui.DatasetExtendedDepthOfFieldProcessor.run(DatasetExtendedDepthOfFieldProcessor.java:69)

     at fr.univ_nantes.codex_processor.gui.MainController$1.handle(MainController.java:96)

     at fr.univ_nantes.codex_processor.gui.MainController$1.handle(MainController.java:64)

     at com.sun.javafx.event.CompositeEventHandler.dispatchBubblingEvent(CompositeEventHandler.java:86)

     at com.sun.javafx.event.EventHandlerManager.dispatchBubblingEvent(EventHandlerManager.java:234)

     at com.sun.javafx.event.EventHandlerManager.dispatchBubblingEvent(EventHandlerManager.java:191)

     at com.sun.javafx.event.CompositeEventDispatcher.dispatchBubblingEvent(CompositeEventDispatcher.java:59)

     at com.sun.javafx.event.BasicEventDispatcher.dispatchEvent(BasicEventDispatcher.java:58)

     at com.sun.javafx.event.EventDispatchChainImpl.dispatchEvent(EventDispatchChainImpl.java:114)

     at com.sun.javafx.event.BasicEventDispatcher.dispatchEvent(BasicEventDispatcher.java:56)

     at com.sun.javafx.event.EventDispatchChainImpl.dispatchEvent(EventDispatchChainImpl.java:114)

     at com.sun.javafx.event.BasicEventDispatcher.dispatchEvent(BasicEventDispatcher.java:56)

     at com.sun.javafx.event.EventDispatchChainImpl.dispatchEvent(EventDispatchChainImpl.java:114)

     at com.sun.javafx.event.EventUtil.fireEventImpl(EventUtil.java:74)

     at com.sun.javafx.event.EventUtil.fireEvent(EventUtil.java:49)

     at javafx.event.Event.fireEvent(Event.java:198)

     at javafx.scene.Node.fireEvent(Node.java:8889)

     at javafx.scene.control.Button.fire(Button.java:203)

     at com.sun.javafx.scene.control.behavior.ButtonBehavior.mouseReleased(ButtonBehavior.java:208)

     at com.sun.javafx.scene.control.inputmap.InputMap.handle(InputMap.java:274)

     at com.sun.javafx.event.CompositeEventHandler$NormalEventHandlerRecord.handleBubblingEvent(CompositeEventHandler.java:247)

     at com.sun.javafx.event.CompositeEventHandler.dispatchBubblingEvent(CompositeEventHandler.java:80)

     at com.sun.javafx.event.EventHandlerManager.dispatchBubblingEvent(EventHandlerManager.java:234)

     at com.sun.javafx.event.EventHandlerManager.dispatchBubblingEvent(EventHandlerManager.java:191)

     at com.sun.javafx.event.CompositeEventDispatcher.dispatchBubblingEvent(CompositeEventDispatcher.java:59)

     at com.sun.javafx.event.BasicEventDispatcher.dispatchEvent(BasicEventDispatcher.java:58)

     at com.sun.javafx.event.EventDispatchChainImpl.dispatchEvent(EventDispatchChainImpl.java:114)

     at com.sun.javafx.event.BasicEventDispatcher.dispatchEvent(BasicEventDispatcher.java:56)

     at com.sun.javafx.event.EventDispatchChainImpl.dispatchEvent(EventDispatchChainImpl.java:114)

     at com.sun.javafx.event.BasicEventDispatcher.dispatchEvent(BasicEventDispatcher.java:56)

     at com.sun.javafx.event.EventDispatchChainImpl.dispatchEvent(EventDispatchChainImpl.java:114)

     at com.sun.javafx.event.EventUtil.fireEventImpl(EventUtil.java:74)

     at com.sun.javafx.event.EventUtil.fireEvent(EventUtil.java:54)

     at javafx.event.Event.fireEvent(Event.java:198)

     at javafx.scene.Scene$MouseHandler.process(Scene.java:3856)

     at javafx.scene.Scene.processMouseEvent(Scene.java:1851)

     at javafx.scene.Scene$ScenePeerListener.mouseEvent(Scene.java:2584)

     at com.sun.javafx.tk.quantum.GlassViewEventHandler$MouseEventNotification.run(GlassViewEventHandler.java:409)

     at com.sun.javafx.tk.quantum.GlassViewEventHandler$MouseEventNotification.run(GlassViewEventHandler.java:299)

     at java.base/java.security.AccessController.doPrivileged(Native Method)

     at com.sun.javafx.tk.quantum.GlassViewEventHandler.lambda$handleMouseEvent$2(GlassViewEventHandler.java:447)

     at com.sun.javafx.tk.quantum.QuantumToolkit.runWithoutRenderLock(QuantumToolkit.java:412)

     at com.sun.javafx.tk.quantum.GlassViewEventHandler.handleMouseEvent(GlassViewEventHandler.java:446)

     at com.sun.glass.ui.View.handleMouseEvent(View.java:556)

     at com.sun.glass.ui.View.notifyMouse(View.java:942)

     at com.sun.glass.ui.win.WinApplication._runLoop(Native Method)

     at com.sun.glass.ui.win.WinApplication.lambda$runLoop$3(WinApplication.java:174)

     at java.base/java.lang.Thread.run(Thread.java:829)

 Caused by: java.lang.OutOfMemoryError

     at java.base/jdk.internal.reflect.GeneratedConstructorAccessor3.newInstance(Unknown Source)

     at java.base/jdk.internal.reflect.DelegatingConstructorAccessorImpl.newInstance(DelegatingConstructorAccessorImpl.java:45)

     at java.base/java.lang.reflect.Constructor.newInstance(Constructor.java:490)

     at java.base/java.util.concurrent.ForkJoinTask.getThrowableException(ForkJoinTask.java:603)

     ... 49 more

 Caused by: java.lang.OutOfMemoryError: Java heap space

 java.util.concurrent.ExecutionException: java.lang.OutOfMemoryError

     at java.base/java.util.concurrent.ForkJoinTask.get(ForkJoinTask.java:1006)

     at fr.univ_nantes.codex_processor.gui.DatasetExtendedDepthOfFieldProcessor.run(DatasetExtendedDepthOfFieldProcessor.java:69)

     at fr.univ_nantes.codex_processor.gui.MainController$1.handle(MainController.java:96)

     at fr.univ_nantes.codex_processor.gui.MainController$1.handle(MainController.java:64)

     at com.sun.javafx.event.CompositeEventHandler.dispatchBubblingEvent(CompositeEventHandler.java:86)

     at com.sun.javafx.event.EventHandlerManager.dispatchBubblingEvent(EventHandlerManager.java:234)

     at com.sun.javafx.event.EventHandlerManager.dispatchBubblingEvent(EventHandlerManager.java:191)

     at com.sun.javafx.event.CompositeEventDispatcher.dispatchBubblingEvent(CompositeEventDispatcher.java:59)

     at com.sun.javafx.event.BasicEventDispatcher.dispatchEvent(BasicEventDispatcher.java:58)

     at com.sun.javafx.event.EventDispatchChainImpl.dispatchEvent(EventDispatchChainImpl.java:114)

     at com.sun.javafx.event.BasicEventDispatcher.dispatchEvent(BasicEventDispatcher.java:56)

     at com.sun.javafx.event.EventDispatchChainImpl.dispatchEvent(EventDispatchChainImpl.java:114)

     at com.sun.javafx.event.BasicEventDispatcher.dispatchEvent(BasicEventDispatcher.java:56)

     at com.sun.javafx.event.EventDispatchChainImpl.dispatchEvent(EventDispatchChainImpl.java:114)

     at com.sun.javafx.event.EventUtil.fireEventImpl(EventUtil.java:74)

     at com.sun.javafx.event.EventUtil.fireEvent(EventUtil.java:49)

     at javafx.event.Event.fireEvent(Event.java:198)

     at javafx.scene.Node.fireEvent(Node.java:8889)

     at javafx.scene.control.Button.fire(Button.java:203)

     at com.sun.javafx.scene.control.behavior.ButtonBehavior.mouseReleased(ButtonBehavior.java:208)

     at com.sun.javafx.scene.control.inputmap.InputMap.handle(InputMap.java:274)

     at com.sun.javafx.event.CompositeEventHandler$NormalEventHandlerRecord.handleBubblingEvent(CompositeEventHandler.java:247)

     at com.sun.javafx.event.CompositeEventHandler.dispatchBubblingEvent(CompositeEventHandler.java:80)

     at com.sun.javafx.event.EventHandlerManager.dispatchBubblingEvent(EventHandlerManager.java:234)

     at com.sun.javafx.event.EventHandlerManager.dispatchBubblingEvent(EventHandlerManager.java:191)

     at com.sun.javafx.event.CompositeEventDispatcher.dispatchBubblingEvent(CompositeEventDispatcher.java:59)

     at com.sun.javafx.event.BasicEventDispatcher.dispatchEvent(BasicEventDispatcher.java:58)

     at com.sun.javafx.event.EventDispatchChainImpl.dispatchEvent(EventDispatchChainImpl.java:114)

     at com.sun.javafx.event.BasicEventDispatcher.dispatchEvent(BasicEventDispatcher.java:56)

     at com.sun.javafx.event.EventDispatchChainImpl.dispatchEvent(EventDispatchChainImpl.java:114)

     at com.sun.javafx.event.BasicEventDispatcher.dispatchEvent(BasicEventDispatcher.java:56)

     at com.sun.javafx.event.EventDispatchChainImpl.dispatchEvent(EventDispatchChainImpl.java:114)

     at com.sun.javafx.event.EventUtil.fireEventImpl(EventUtil.java:74)

     at com.sun.javafx.event.EventUtil.fireEvent(EventUtil.java:54)

     at javafx.event.Event.fireEvent(Event.java:198)

     at javafx.scene.Scene$MouseHandler.process(Scene.java:3856)

     at javafx.scene.Scene.processMouseEvent(Scene.java:1851)

     at javafx.scene.Scene$ScenePeerListener.mouseEvent(Scene.java:2584)

     at com.sun.javafx.tk.quantum.GlassViewEventHandler$MouseEventNotification.run(GlassViewEventHandler.java:409)

     at com.sun.javafx.tk.quantum.GlassViewEventHandler$MouseEventNotification.run(GlassViewEventHandler.java:299)

     at java.base/java.security.AccessController.doPrivileged(Native Method)

     at com.sun.javafx.tk.quantum.GlassViewEventHandler.lambda$handleMouseEvent$2(GlassViewEventHandler.java:447)

     at com.sun.javafx.tk.quantum.QuantumToolkit.runWithoutRenderLock(QuantumToolkit.java:412)

     at com.sun.javafx.tk.quantum.GlassViewEventHandler.handleMouseEvent(GlassViewEventHandler.java:446)

     at com.sun.glass.ui.View.handleMouseEvent(View.java:556)

     at com.sun.glass.ui.View.notifyMouse(View.java:942)

     at com.sun.glass.ui.win.WinApplication._runLoop(Native Method)

     at com.sun.glass.ui.win.WinApplication.lambda$runLoop$3(WinApplication.java:174)

     at java.base/java.lang.Thread.run(Thread.java:829)

 Caused by: java.lang.OutOfMemoryError

     at java.base/jdk.internal.reflect.GeneratedConstructorAccessor3.newInstance(Unknown Source)

     at java.base/jdk.internal.reflect.DelegatingConstructorAccessorImpl.newInstance(DelegatingConstructorAccessorImpl.java:45)

     at java.base/java.lang.reflect.Constructor.newInstance(Constructor.java:490)

     at java.base/java.util.concurrent.ForkJoinTask.getThrowableException(ForkJoinTask.java:603)

     ... 49 more

 Caused by: java.lang.OutOfMemoryError: Java heap space

 java.util.concurrent.ExecutionException: java.lang.OutOfMemoryError

     at java.base/java.util.concurrent.ForkJoinTask.get(ForkJoinTask.java:1006)

     at fr.univ_nantes.codex_processor.gui.DatasetExtendedDepthOfFieldProcessor.run(DatasetExtendedDepthOfFieldProcessor.java:69)

     at fr.univ_nantes.codex_processor.gui.MainController$1.handle(MainController.java:96)

     at fr.univ_nantes.codex_processor.gui.MainController$1.handle(MainController.java:64)

     at com.sun.javafx.event.CompositeEventHandler.dispatchBubblingEvent(CompositeEventHandler.java:86)

     at com.sun.javafx.event.EventHandlerManager.dispatchBubblingEvent(EventHandlerManager.java:234)

     at com.sun.javafx.event.EventHandlerManager.dispatchBubblingEvent(EventHandlerManager.java:191)

     at com.sun.javafx.event.CompositeEventDispatcher.dispatchBubblingEvent(CompositeEventDispatcher.java:59)

     at com.sun.javafx.event.BasicEventDispatcher.dispatchEvent(BasicEventDispatcher.java:58)

     at com.sun.javafx.event.EventDispatchChainImpl.dispatchEvent(EventDispatchChainImpl.java:114)

     at com.sun.javafx.event.BasicEventDispatcher.dispatchEvent(BasicEventDispatcher.java:56)

     at com.sun.javafx.event.EventDispatchChainImpl.dispatchEvent(EventDispatchChainImpl.java:114)

     at com.sun.javafx.event.BasicEventDispatcher.dispatchEvent(BasicEventDispatcher.java:56)

     at com.sun.javafx.event.EventDispatchChainImpl.dispatchEvent(EventDispatchChainImpl.java:114)

     at com.sun.javafx.event.EventUtil.fireEventImpl(EventUtil.java:74)

     at com.sun.javafx.event.EventUtil.fireEvent(EventUtil.java:49)

     at javafx.event.Event.fireEvent(Event.java:198)

     at javafx.scene.Node.fireEvent(Node.java:8889)

     at javafx.scene.control.Button.fire(Button.java:203)

     at com.sun.javafx.scene.control.behavior.ButtonBehavior.mouseReleased(ButtonBehavior.java:208)

     at com.sun.javafx.scene.control.inputmap.InputMap.handle(InputMap.java:274)

     at com.sun.javafx.event.CompositeEventHandler$NormalEventHandlerRecord.handleBubblingEvent(CompositeEventHandler.java:247)

     at com.sun.javafx.event.CompositeEventHandler.dispatchBubblingEvent(CompositeEventHandler.java:80)

     at com.sun.javafx.event.EventHandlerManager.dispatchBubblingEvent(EventHandlerManager.java:234)

     at com.sun.javafx.event.EventHandlerManager.dispatchBubblingEvent(EventHandlerManager.java:191)

     at com.sun.javafx.event.CompositeEventDispatcher.dispatchBubblingEvent(CompositeEventDispatcher.java:59)

     at com.sun.javafx.event.BasicEventDispatcher.dispatchEvent(BasicEventDispatcher.java:58)

     at com.sun.javafx.event.EventDispatchChainImpl.dispatchEvent(EventDispatchChainImpl.java:114)

     at com.sun.javafx.event.BasicEventDispatcher.dispatchEvent(BasicEventDispatcher.java:56)

     at com.sun.javafx.event.EventDispatchChainImpl.dispatchEvent(EventDispatchChainImpl.java:114)

     at com.sun.javafx.event.BasicEventDispatcher.dispatchEvent(BasicEventDispatcher.java:56)

     at com.sun.javafx.event.EventDispatchChainImpl.dispatchEvent(EventDispatchChainImpl.java:114)

     at com.sun.javafx.event.EventUtil.fireEventImpl(EventUtil.java:74)

     at com.sun.javafx.event.EventUtil.fireEvent(EventUtil.java:54)

     at javafx.event.Event.fireEvent(Event.java:198)

     at javafx.scene.Scene$MouseHandler.process(Scene.java:3856)

     at javafx.scene.Scene.processMouseEvent(Scene.java:1851)

     at javafx.scene.Scene$ScenePeerListener.mouseEvent(Scene.java:2584)

     at com.sun.javafx.tk.quantum.GlassViewEventHandler$MouseEventNotification.run(GlassViewEventHandler.java:409)

     at com.sun.javafx.tk.quantum.GlassViewEventHandler$MouseEventNotification.run(GlassViewEventHandler.java:299)

     at java.base/java.security.AccessController.doPrivileged(Native Method)

     at com.sun.javafx.tk.quantum.GlassViewEventHandler.lambda$handleMouseEvent$2(GlassViewEventHandler.java:447)

     at com.sun.javafx.tk.quantum.QuantumToolkit.runWithoutRenderLock(QuantumToolkit.java:412)

     at com.sun.javafx.tk.quantum.GlassViewEventHandler.handleMouseEvent(GlassViewEventHandler.java:446)

     at com.sun.glass.ui.View.handleMouseEvent(View.java:556)

     at com.sun.glass.ui.View.notifyMouse(View.java:942)

     at com.sun.glass.ui.win.WinApplication._runLoop(Native Method)

     at com.sun.glass.ui.win.WinApplication.lambda$runLoop$3(WinApplication.java:174)

     at java.base/java.lang.Thread.run(Thread.java:829)

 Caused by: java.lang.OutOfMemoryError

     at java.base/jdk.internal.reflect.GeneratedConstructorAccessor3.newInstance(Unknown Source)

     at java.base/jdk.internal.reflect.DelegatingConstructorAccessorImpl.newInstance(DelegatingConstructorAccessorImpl.java:45)

     at java.base/java.lang.reflect.Constructor.newInstance(Constructor.java:490)

     at java.base/java.util.concurrent.ForkJoinTask.getThrowableException(ForkJoinTask.java:603)

     ... 49 more

 Caused by: java.lang.OutOfMemoryError: Java heap space

 java.util.concurrent.ExecutionException: java.lang.OutOfMemoryError

     at java.base/java.util.concurrent.ForkJoinTask.get(ForkJoinTask.java:1006)

     at fr.univ_nantes.codex_processor.gui.DatasetExtendedDepthOfFieldProcessor.run(DatasetExtendedDepthOfFieldProcessor.java:69)

     at fr.univ_nantes.codex_processor.gui.MainController$1.handle(MainController.java:96)

     at fr.univ_nantes.codex_processor.gui.MainController$1.handle(MainController.java:64)

     at com.sun.javafx.event.CompositeEventHandler.dispatchBubblingEvent(CompositeEventHandler.java:86)

     at com.sun.javafx.event.EventHandlerManager.dispatchBubblingEvent(EventHandlerManager.java:234)

     at com.sun.javafx.event.EventHandlerManager.dispatchBubblingEvent(EventHandlerManager.java:191)

     at com.sun.javafx.event.CompositeEventDispatcher.dispatchBubblingEvent(CompositeEventDispatcher.java:59)

     at com.sun.javafx.event.BasicEventDispatcher.dispatchEvent(BasicEventDispatcher.java:58)

     at com.sun.javafx.event.EventDispatchChainImpl.dispatchEvent(EventDispatchChainImpl.java:114)

     at com.sun.javafx.event.BasicEventDispatcher.dispatchEvent(BasicEventDispatcher.java:56)

     at com.sun.javafx.event.EventDispatchChainImpl.dispatchEvent(EventDispatchChainImpl.java:114)

     at com.sun.javafx.event.BasicEventDispatcher.dispatchEvent(BasicEventDispatcher.java:56)

     at com.sun.javafx.event.EventDispatchChainImpl.dispatchEvent(EventDispatchChainImpl.java:114)

     at com.sun.javafx.event.EventUtil.fireEventImpl(EventUtil.java:74)

     at com.sun.javafx.event.EventUtil.fireEvent(EventUtil.java:49)

     at javafx.event.Event.fireEvent(Event.java:198)

     at javafx.scene.Node.fireEvent(Node.java:8889)

     at javafx.scene.control.Button.fire(Button.java:203)

     at com.sun.javafx.scene.control.behavior.ButtonBehavior.mouseReleased(ButtonBehavior.java:208)

     at com.sun.javafx.scene.control.inputmap.InputMap.handle(InputMap.java:274)

     at com.sun.javafx.event.CompositeEventHandler$NormalEventHandlerRecord.handleBubblingEvent(CompositeEventHandler.java:247)

     at com.sun.javafx.event.CompositeEventHandler.dispatchBubblingEvent(CompositeEventHandler.java:80)

     at com.sun.javafx.event.EventHandlerManager.dispatchBubblingEvent(EventHandlerManager.java:234)

     at com.sun.javafx.event.EventHandlerManager.dispatchBubblingEvent(EventHandlerManager.java:191)

     at com.sun.javafx.event.CompositeEventDispatcher.dispatchBubblingEvent(CompositeEventDispatcher.java:59)

     at com.sun.javafx.event.BasicEventDispatcher.dispatchEvent(BasicEventDispatcher.java:58)

     at com.sun.javafx.event.EventDispatchChainImpl.dispatchEvent(EventDispatchChainImpl.java:114)

     at com.sun.javafx.event.BasicEventDispatcher.dispatchEvent(BasicEventDispatcher.java:56)

     at com.sun.javafx.event.EventDispatchChainImpl.dispatchEvent(EventDispatchChainImpl.java:114)

     at com.sun.javafx.event.BasicEventDispatcher.dispatchEvent(BasicEventDispatcher.java:56)

     at com.sun.javafx.event.EventDispatchChainImpl.dispatchEvent(EventDispatchChainImpl.java:114)

     at com.sun.javafx.event.EventUtil.fireEventImpl(EventUtil.java:74)

     at com.sun.javafx.event.EventUtil.fireEvent(EventUtil.java:54)

     at javafx.event.Event.fireEvent(Event.java:198)

     at javafx.scene.Scene$MouseHandler.process(Scene.java:3856)

     at javafx.scene.Scene.processMouseEvent(Scene.java:1851)

     at javafx.scene.Scene$ScenePeerListener.mouseEvent(Scene.java:2584)

     at com.sun.javafx.tk.quantum.GlassViewEventHandler$MouseEventNotification.run(GlassViewEventHandler.java:409)

     at com.sun.javafx.tk.quantum.GlassViewEventHandler$MouseEventNotification.run(GlassViewEventHandler.java:299)

     at java.base/java.security.AccessController.doPrivileged(Native Method)

     at com.sun.javafx.tk.quantum.GlassViewEventHandler.lambda$handleMouseEvent$2(GlassViewEventHandler.java:447)

     at com.sun.javafx.tk.quantum.QuantumToolkit.runWithoutRenderLock(QuantumToolkit.java:412)

     at com.sun.javafx.tk.quantum.GlassViewEventHandler.handleMouseEvent(GlassViewEventHandler.java:446)

     at com.sun.glass.ui.View.handleMouseEvent(View.java:556)

     at com.sun.glass.ui.View.notifyMouse(View.java:942)

     at com.sun.glass.ui.win.WinApplication._runLoop(Native Method)

     at com.sun.glass.ui.win.WinApplication.lambda$runLoop$3(WinApplication.java:174)

     at java.base/java.lang.Thread.run(Thread.java:829)

 Caused by: java.lang.OutOfMemoryError

     at java.base/jdk.internal.reflect.GeneratedConstructorAccessor3.newInstance(Unknown Source)

     at java.base/jdk.internal.reflect.DelegatingConstructorAccessorImpl.newInstance(DelegatingConstructorAccessorImpl.java:45)

     at java.base/java.lang.reflect.Constructor.newInstance(Constructor.java:490)

     at java.base/java.util.concurrent.ForkJoinTask.getThrowableException(ForkJoinTask.java:603)

     ... 49 more

 Caused by: java.lang.OutOfMemoryError: Java heap space

 java.util.concurrent.ExecutionException: java.lang.OutOfMemoryError

     at java.base/java.util.concurrent.ForkJoinTask.get(ForkJoinTask.java:1006)

     at fr.univ_nantes.codex_processor.gui.DatasetExtendedDepthOfFieldProcessor.run(DatasetExtendedDepthOfFieldProcessor.java:69)

     at fr.univ_nantes.codex_processor.gui.MainController$1.handle(MainController.java:96)

     at fr.univ_nantes.codex_processor.gui.MainController$1.handle(MainController.java:64)

     at com.sun.javafx.event.CompositeEventHandler.dispatchBubblingEvent(CompositeEventHandler.java:86)

     at com.sun.javafx.event.EventHandlerManager.dispatchBubblingEvent(EventHandlerManager.java:234)

     at com.sun.javafx.event.EventHandlerManager.dispatchBubblingEvent(EventHandlerManager.java:191)

     at com.sun.javafx.event.CompositeEventDispatcher.dispatchBubblingEvent(CompositeEventDispatcher.java:59)

     at com.sun.javafx.event.BasicEventDispatcher.dispatchEvent(BasicEventDispatcher.java:58)

     at com.sun.javafx.event.EventDispatchChainImpl.dispatchEvent(EventDispatchChainImpl.java:114)

     at com.sun.javafx.event.BasicEventDispatcher.dispatchEvent(BasicEventDispatcher.java:56)

     at com.sun.javafx.event.EventDispatchChainImpl.dispatchEvent(EventDispatchChainImpl.java:114)

     at com.sun.javafx.event.BasicEventDispatcher.dispatchEvent(BasicEventDispatcher.java:56)

     at com.sun.javafx.event.EventDispatchChainImpl.dispatchEvent(EventDispatchChainImpl.java:114)

     at com.sun.javafx.event.EventUtil.fireEventImpl(EventUtil.java:74)

     at com.sun.javafx.event.EventUtil.fireEvent(EventUtil.java:49)

     at javafx.event.Event.fireEvent(Event.java:198)

     at javafx.scene.Node.fireEvent(Node.java:8889)

     at javafx.scene.control.Button.fire(Button.java:203)

     at com.sun.javafx.scene.control.behavior.ButtonBehavior.mouseReleased(ButtonBehavior.java:208)

     at com.sun.javafx.scene.control.inputmap.InputMap.handle(InputMap.java:274)

     at com.sun.javafx.event.CompositeEventHandler$NormalEventHandlerRecord.handleBubblingEvent(CompositeEventHandler.java:247)

     at com.sun.javafx.event.CompositeEventHandler.dispatchBubblingEvent(CompositeEventHandler.java:80)

     at com.sun.javafx.event.EventHandlerManager.dispatchBubblingEvent(EventHandlerManager.java:234)

     at com.sun.javafx.event.EventHandlerManager.dispatchBubblingEvent(EventHandlerManager.java:191)

     at com.sun.javafx.event.CompositeEventDispatcher.dispatchBubblingEvent(CompositeEventDispatcher.java:59)

     at com.sun.javafx.event.BasicEventDispatcher.dispatchEvent(BasicEventDispatcher.java:58)

     at com.sun.javafx.event.EventDispatchChainImpl.dispatchEvent(EventDispatchChainImpl.java:114)

     at com.sun.javafx.event.BasicEventDispatcher.dispatchEvent(BasicEventDispatcher.java:56)

     at com.sun.javafx.event.EventDispatchChainImpl.dispatchEvent(EventDispatchChainImpl.java:114)

     at com.sun.javafx.event.BasicEventDispatcher.dispatchEvent(BasicEventDispatcher.java:56)

     at com.sun.javafx.event.EventDispatchChainImpl.dispatchEvent(EventDispatchChainImpl.java:114)

     at com.sun.javafx.event.EventUtil.fireEventImpl(EventUtil.java:74)

     at com.sun.javafx.event.EventUtil.fireEvent(EventUtil.java:54)

     at javafx.event.Event.fireEvent(Event.java:198)

     at javafx.scene.Scene$MouseHandler.process(Scene.java:3856)

     at javafx.scene.Scene.processMouseEvent(Scene.java:1851)

     at javafx.scene.Scene$ScenePeerListener.mouseEvent(Scene.java:2584)

     at com.sun.javafx.tk.quantum.GlassViewEventHandler$MouseEventNotification.run(GlassViewEventHandler.java:409)

     at com.sun.javafx.tk.quantum.GlassViewEventHandler$MouseEventNotification.run(GlassViewEventHandler.java:299)

     at java.base/java.security.AccessController.doPrivileged(Native Method)

     at com.sun.javafx.tk.quantum.GlassViewEventHandler.lambda$handleMouseEvent$2(GlassViewEventHandler.java:447)

     at com.sun.javafx.tk.quantum.QuantumToolkit.runWithoutRenderLock(QuantumToolkit.java:412)

     at com.sun.javafx.tk.quantum.GlassViewEventHandler.handleMouseEvent(GlassViewEventHandler.java:446)

     at com.sun.glass.ui.View.handleMouseEvent(View.java:556)

     at com.sun.glass.ui.View.notifyMouse(View.java:942)

     at com.sun.glass.ui.win.WinApplication._runLoop(Native Method)

     at com.sun.glass.ui.win.WinApplication.lambda$runLoop$3(WinApplication.java:174)

     at java.base/java.lang.Thread.run(Thread.java:829)

 Caused by: java.lang.OutOfMemoryError

     at java.base/jdk.internal.reflect.GeneratedConstructorAccessor3.newInstance(Unknown Source)

     at java.base/jdk.internal.reflect.DelegatingConstructorAccessorImpl.newInstance(DelegatingConstructorAccessorImpl.java:45)

     at java.base/java.lang.reflect.Constructor.newInstance(Constructor.java:490)

     at java.base/java.util.concurrent.ForkJoinTask.getThrowableException(ForkJoinTask.java:603)

     ... 49 more

 Caused by: java.lang.OutOfMemoryError: Java heap space

 java.util.concurrent.ExecutionException: java.lang.OutOfMemoryError

     at java.base/java.util.concurrent.ForkJoinTask.get(ForkJoinTask.java:1006)

     at fr.univ_nantes.codex_processor.gui.DatasetExtendedDepthOfFieldProcessor.run(DatasetExtendedDepthOfFieldProcessor.java:69)

     at fr.univ_nantes.codex_processor.gui.MainController$1.handle(MainController.java:96)

     at fr.univ_nantes.codex_processor.gui.MainController$1.handle(MainController.java:64)

     at com.sun.javafx.event.CompositeEventHandler.dispatchBubblingEvent(CompositeEventHandler.java:86)

     at com.sun.javafx.event.EventHandlerManager.dispatchBubblingEvent(EventHandlerManager.java:234)

     at com.sun.javafx.event.EventHandlerManager.dispatchBubblingEvent(EventHandlerManager.java:191)

     at com.sun.javafx.event.CompositeEventDispatcher.dispatchBubblingEvent(CompositeEventDispatcher.java:59)

     at com.sun.javafx.event.BasicEventDispatcher.dispatchEvent(BasicEventDispatcher.java:58)

     at com.sun.javafx.event.EventDispatchChainImpl.dispatchEvent(EventDispatchChainImpl.java:114)

     at com.sun.javafx.event.BasicEventDispatcher.dispatchEvent(BasicEventDispatcher.java:56)

     at com.sun.javafx.event.EventDispatchChainImpl.dispatchEvent(EventDispatchChainImpl.java:114)

     at com.sun.javafx.event.BasicEventDispatcher.dispatchEvent(BasicEventDispatcher.java:56)

     at com.sun.javafx.event.EventDispatchChainImpl.dispatchEvent(EventDispatchChainImpl.java:114)

     at com.sun.javafx.event.EventUtil.fireEventImpl(EventUtil.java:74)

     at com.sun.javafx.event.EventUtil.fireEvent(EventUtil.java:49)

     at javafx.event.Event.fireEvent(Event.java:198)

     at javafx.scene.Node.fireEvent(Node.java:8889)

     at javafx.scene.control.Button.fire(Button.java:203)

     at com.sun.javafx.scene.control.behavior.ButtonBehavior.mouseReleased(ButtonBehavior.java:208)

     at com.sun.javafx.scene.control.inputmap.InputMap.handle(InputMap.java:274)

     at com.sun.javafx.event.CompositeEventHandler$NormalEventHandlerRecord.handleBubblingEvent(CompositeEventHandler.java:247)

     at com.sun.javafx.event.CompositeEventHandler.dispatchBubblingEvent(CompositeEventHandler.java:80)

     at com.sun.javafx.event.EventHandlerManager.dispatchBubblingEvent(EventHandlerManager.java:234)

     at com.sun.javafx.event.EventHandlerManager.dispatchBubblingEvent(EventHandlerManager.java:191)

     at com.sun.javafx.event.CompositeEventDispatcher.dispatchBubblingEvent(CompositeEventDispatcher.java:59)

     at com.sun.javafx.event.BasicEventDispatcher.dispatchEvent(BasicEventDispatcher.java:58)

     at com.sun.javafx.event.EventDispatchChainImpl.dispatchEvent(EventDispatchChainImpl.java:114)

     at com.sun.javafx.event.BasicEventDispatcher.dispatchEvent(BasicEventDispatcher.java:56)

     at com.sun.javafx.event.EventDispatchChainImpl.dispatchEvent(EventDispatchChainImpl.java:114)

     at com.sun.javafx.event.BasicEventDispatcher.dispatchEvent(BasicEventDispatcher.java:56)

     at com.sun.javafx.event.EventDispatchChainImpl.dispatchEvent(EventDispatchChainImpl.java:114)

     at com.sun.javafx.event.EventUtil.fireEventImpl(EventUtil.java:74)

     at com.sun.javafx.event.EventUtil.fireEvent(EventUtil.java:54)

     at javafx.event.Event.fireEvent(Event.java:198)

     at javafx.scene.Scene$MouseHandler.process(Scene.java:3856)

     at javafx.scene.Scene.processMouseEvent(Scene.java:1851)

     at javafx.scene.Scene$ScenePeerListener.mouseEvent(Scene.java:2584)

     at com.sun.javafx.tk.quantum.GlassViewEventHandler$MouseEventNotification.run(GlassViewEventHandler.java:409)

     at com.sun.javafx.tk.quantum.GlassViewEventHandler$MouseEventNotification.run(GlassViewEventHandler.java:299)

     at java.base/java.security.AccessController.doPrivileged(Native Method)

     at com.sun.javafx.tk.quantum.GlassViewEventHandler.lambda$handleMouseEvent$2(GlassViewEventHandler.java:447)

     at com.sun.javafx.tk.quantum.QuantumToolkit.runWithoutRenderLock(QuantumToolkit.java:412)

     at com.sun.javafx.tk.quantum.GlassViewEventHandler.handleMouseEvent(GlassViewEventHandler.java:446)

     at com.sun.glass.ui.View.handleMouseEvent(View.java:556)

     at com.sun.glass.ui.View.notifyMouse(View.java:942)

     at com.sun.glass.ui.win.WinApplication._runLoop(Native Method)

     at com.sun.glass.ui.win.WinApplication.lambda$runLoop$3(WinApplication.java:174)

     at java.base/java.lang.Thread.run(Thread.java:829)

 Caused by: java.lang.OutOfMemoryError

     at java.base/jdk.internal.reflect.GeneratedConstructorAccessor3.newInstance(Unknown Source)

     at java.base/jdk.internal.reflect.DelegatingConstructorAccessorImpl.newInstance(DelegatingConstructorAccessorImpl.java:45)

     at java.base/java.lang.reflect.Constructor.newInstance(Constructor.java:490)

     at java.base/java.util.concurrent.ForkJoinTask.getThrowableException(ForkJoinTask.java:603)

     ... 49 more

 Caused by: java.lang.OutOfMemoryError: Java heap space

 java.util.concurrent.ExecutionException: java.lang.OutOfMemoryError

     at java.base/java.util.concurrent.ForkJoinTask.get(ForkJoinTask.java:1006)

     at fr.univ_nantes.codex_processor.gui.DatasetExtendedDepthOfFieldProcessor.run(DatasetExtendedDepthOfFieldProcessor.java:69)

     at fr.univ_nantes.codex_processor.gui.MainController$1.handle(MainController.java:96)

     at fr.univ_nantes.codex_processor.gui.MainController$1.handle(MainController.java:64)

     at com.sun.javafx.event.CompositeEventHandler.dispatchBubblingEvent(CompositeEventHandler.java:86)

     at com.sun.javafx.event.EventHandlerManager.dispatchBubblingEvent(EventHandlerManager.java:234)

     at com.sun.javafx.event.EventHandlerManager.dispatchBubblingEvent(EventHandlerManager.java:191)

     at com.sun.javafx.event.CompositeEventDispatcher.dispatchBubblingEvent(CompositeEventDispatcher.java:59)

     at com.sun.javafx.event.BasicEventDispatcher.dispatchEvent(BasicEventDispatcher.java:58)

     at com.sun.javafx.event.EventDispatchChainImpl.dispatchEvent(EventDispatchChainImpl.java:114)

     at com.sun.javafx.event.BasicEventDispatcher.dispatchEvent(BasicEventDispatcher.java:56)

     at com.sun.javafx.event.EventDispatchChainImpl.dispatchEvent(EventDispatchChainImpl.java:114)

     at com.sun.javafx.event.BasicEventDispatcher.dispatchEvent(BasicEventDispatcher.java:56)

     at com.sun.javafx.event.EventDispatchChainImpl.dispatchEvent(EventDispatchChainImpl.java:114)

     at com.sun.javafx.event.EventUtil.fireEventImpl(EventUtil.java:74)

     at com.sun.javafx.event.EventUtil.fireEvent(EventUtil.java:49)

     at javafx.event.Event.fireEvent(Event.java:198)

     at javafx.scene.Node.fireEvent(Node.java:8889)

     at javafx.scene.control.Button.fire(Button.java:203)

     at com.sun.javafx.scene.control.behavior.ButtonBehavior.mouseReleased(ButtonBehavior.java:208)

     at com.sun.javafx.scene.control.inputmap.InputMap.handle(InputMap.java:274)

     at com.sun.javafx.event.CompositeEventHandler$NormalEventHandlerRecord.handleBubblingEvent(CompositeEventHandler.java:247)

     at com.sun.javafx.event.CompositeEventHandler.dispatchBubblingEvent(CompositeEventHandler.java:80)

     at com.sun.javafx.event.EventHandlerManager.dispatchBubblingEvent(EventHandlerManager.java:234)

     at com.sun.javafx.event.EventHandlerManager.dispatchBubblingEvent(EventHandlerManager.java:191)

     at com.sun.javafx.event.CompositeEventDispatcher.dispatchBubblingEvent(CompositeEventDispatcher.java:59)

     at com.sun.javafx.event.BasicEventDispatcher.dispatchEvent(BasicEventDispatcher.java:58)

     at com.sun.javafx.event.EventDispatchChainImpl.dispatchEvent(EventDispatchChainImpl.java:114)

     at com.sun.javafx.event.BasicEventDispatcher.dispatchEvent(BasicEventDispatcher.java:56)

     at com.sun.javafx.event.EventDispatchChainImpl.dispatchEvent(EventDispatchChainImpl.java:114)

     at com.sun.javafx.event.BasicEventDispatcher.dispatchEvent(BasicEventDispatcher.java:56)

     at com.sun.javafx.event.EventDispatchChainImpl.dispatchEvent(EventDispatchChainImpl.java:114)

     at com.sun.javafx.event.EventUtil.fireEventImpl(EventUtil.java:74)

     at com.sun.javafx.event.EventUtil.fireEvent(EventUtil.java:54)

     at javafx.event.Event.fireEvent(Event.java:198)

     at javafx.scene.Scene$MouseHandler.process(Scene.java:3856)

     at javafx.scene.Scene.processMouseEvent(Scene.java:1851)

     at javafx.scene.Scene$ScenePeerListener.mouseEvent(Scene.java:2584)

     at com.sun.javafx.tk.quantum.GlassViewEventHandler$MouseEventNotification.run(GlassViewEventHandler.java:409)

     at com.sun.javafx.tk.quantum.GlassViewEventHandler$MouseEventNotification.run(GlassViewEventHandler.java:299)

     at java.base/java.security.AccessController.doPrivileged(Native Method)

     at com.sun.javafx.tk.quantum.GlassViewEventHandler.lambda$handleMouseEvent$2(GlassViewEventHandler.java:447)

     at com.sun.javafx.tk.quantum.QuantumToolkit.runWithoutRenderLock(QuantumToolkit.java:412)

     at com.sun.javafx.tk.quantum.GlassViewEventHandler.handleMouseEvent(GlassViewEventHandler.java:446)

     at com.sun.glass.ui.View.handleMouseEvent(View.java:556)

     at com.sun.glass.ui.View.notifyMouse(View.java:942)

     at com.sun.glass.ui.win.WinApplication._runLoop(Native Method)

     at com.sun.glass.ui.win.WinApplication.lambda$runLoop$3(WinApplication.java:174)

     at java.base/java.lang.Thread.run(Thread.java:829)

 Caused by: java.lang.OutOfMemoryError

     at java.base/jdk.internal.reflect.GeneratedConstructorAccessor3.newInstance(Unknown Source)

     at java.base/jdk.internal.reflect.DelegatingConstructorAccessorImpl.newInstance(DelegatingConstructorAccessorImpl.java:45)

     at java.base/java.lang.reflect.Constructor.newInstance(Constructor.java:490)

     at java.base/java.util.concurrent.ForkJoinTask.getThrowableException(ForkJoinTask.java:603)

     ... 49 more

 Caused by: java.lang.OutOfMemoryError: Java heap space

 java.util.concurrent.ExecutionException: java.lang.OutOfMemoryError

     at java.base/java.util.concurrent.ForkJoinTask.get(ForkJoinTask.java:1006)

     at fr.univ_nantes.codex_processor.gui.DatasetExtendedDepthOfFieldProcessor.run(DatasetExtendedDepthOfFieldProcessor.java:69)

     at fr.univ_nantes.codex_processor.gui.MainController$1.handle(MainController.java:96)

     at fr.univ_nantes.codex_processor.gui.MainController$1.handle(MainController.java:64)

     at com.sun.javafx.event.CompositeEventHandler.dispatchBubblingEvent(CompositeEventHandler.java:86)

     at com.sun.javafx.event.EventHandlerManager.dispatchBubblingEvent(EventHandlerManager.java:234)

     at com.sun.javafx.event.EventHandlerManager.dispatchBubblingEvent(EventHandlerManager.java:191)

     at com.sun.javafx.event.CompositeEventDispatcher.dispatchBubblingEvent(CompositeEventDispatcher.java:59)

     at com.sun.javafx.event.BasicEventDispatcher.dispatchEvent(BasicEventDispatcher.java:58)

     at com.sun.javafx.event.EventDispatchChainImpl.dispatchEvent(EventDispatchChainImpl.java:114)

     at com.sun.javafx.event.BasicEventDispatcher.dispatchEvent(BasicEventDispatcher.java:56)

     at com.sun.javafx.event.EventDispatchChainImpl.dispatchEvent(EventDispatchChainImpl.java:114)

     at com.sun.javafx.event.BasicEventDispatcher.dispatchEvent(BasicEventDispatcher.java:56)

     at com.sun.javafx.event.EventDispatchChainImpl.dispatchEvent(EventDispatchChainImpl.java:114)

     at com.sun.javafx.event.EventUtil.fireEventImpl(EventUtil.java:74)

     at com.sun.javafx.event.EventUtil.fireEvent(EventUtil.java:49)

     at javafx.event.Event.fireEvent(Event.java:198)

     at javafx.scene.Node.fireEvent(Node.java:8889)

     at javafx.scene.control.Button.fire(Button.java:203)

     at com.sun.javafx.scene.control.behavior.ButtonBehavior.mouseReleased(ButtonBehavior.java:208)

     at com.sun.javafx.scene.control.inputmap.InputMap.handle(InputMap.java:274)

     at com.sun.javafx.event.CompositeEventHandler$NormalEventHandlerRecord.handleBubblingEvent(CompositeEventHandler.java:247)

     at com.sun.javafx.event.CompositeEventHandler.dispatchBubblingEvent(CompositeEventHandler.java:80)

     at com.sun.javafx.event.EventHandlerManager.dispatchBubblingEvent(EventHandlerManager.java:234)

     at com.sun.javafx.event.EventHandlerManager.dispatchBubblingEvent(EventHandlerManager.java:191)

     at com.sun.javafx.event.CompositeEventDispatcher.dispatchBubblingEvent(CompositeEventDispatcher.java:59)

     at com.sun.javafx.event.BasicEventDispatcher.dispatchEvent(BasicEventDispatcher.java:58)

     at com.sun.javafx.event.EventDispatchChainImpl.dispatchEvent(EventDispatchChainImpl.java:114)

     at com.sun.javafx.event.BasicEventDispatcher.dispatchEvent(BasicEventDispatcher.java:56)

     at com.sun.javafx.event.EventDispatchChainImpl.dispatchEvent(EventDispatchChainImpl.java:114)

     at com.sun.javafx.event.BasicEventDispatcher.dispatchEvent(BasicEventDispatcher.java:56)

     at com.sun.javafx.event.EventDispatchChainImpl.dispatchEvent(EventDispatchChainImpl.java:114)

     at com.sun.javafx.event.EventUtil.fireEventImpl(EventUtil.java:74)

     at com.sun.javafx.event.EventUtil.fireEvent(EventUtil.java:54)

     at javafx.event.Event.fireEvent(Event.java:198)

     at javafx.scene.Scene$MouseHandler.process(Scene.java:3856)

     at javafx.scene.Scene.processMouseEvent(Scene.java:1851)

     at javafx.scene.Scene$ScenePeerListener.mouseEvent(Scene.java:2584)

     at com.sun.javafx.tk.quantum.GlassViewEventHandler$MouseEventNotification.run(GlassViewEventHandler.java:409)

     at com.sun.javafx.tk.quantum.GlassViewEventHandler$MouseEventNotification.run(GlassViewEventHandler.java:299)

     at java.base/java.security.AccessController.doPrivileged(Native Method)

     at com.sun.javafx.tk.quantum.GlassViewEventHandler.lambda$handleMouseEvent$2(GlassViewEventHandler.java:447)

     at com.sun.javafx.tk.quantum.QuantumToolkit.runWithoutRenderLock(QuantumToolkit.java:412)

     at com.sun.javafx.tk.quantum.GlassViewEventHandler.handleMouseEvent(GlassViewEventHandler.java:446)

     at com.sun.glass.ui.View.handleMouseEvent(View.java:556)

     at com.sun.glass.ui.View.notifyMouse(View.java:942)

     at com.sun.glass.ui.win.WinApplication._runLoop(Native Method)

     at com.sun.glass.ui.win.WinApplication.lambda$runLoop$3(WinApplication.java:174)

     at java.base/java.lang.Thread.run(Thread.java:829)

 Caused by: java.lang.OutOfMemoryError

     at java.base/jdk.internal.reflect.GeneratedConstructorAccessor3.newInstance(Unknown Source)

     at java.base/jdk.internal.reflect.DelegatingConstructorAccessorImpl.newInstance(DelegatingConstructorAccessorImpl.java:45)

     at java.base/java.lang.reflect.Constructor.newInstance(Constructor.java:490)

     at java.base/java.util.concurrent.ForkJoinTask.getThrowableException(ForkJoinTask.java:603)

     ... 49 more

 Caused by: java.lang.OutOfMemoryError: Java heap space

 java.util.concurrent.ExecutionException: java.lang.OutOfMemoryError

     at java.base/java.util.concurrent.ForkJoinTask.get(ForkJoinTask.java:1006)

     at fr.univ_nantes.codex_processor.gui.DatasetExtendedDepthOfFieldProcessor.run(DatasetExtendedDepthOfFieldProcessor.java:69)

     at fr.univ_nantes.codex_processor.gui.MainController$1.handle(MainController.java:96)

     at fr.univ_nantes.codex_processor.gui.MainController$1.handle(MainController.java:64)

     at com.sun.javafx.event.CompositeEventHandler.dispatchBubblingEvent(CompositeEventHandler.java:86)

     at com.sun.javafx.event.EventHandlerManager.dispatchBubblingEvent(EventHandlerManager.java:234)

     at com.sun.javafx.event.EventHandlerManager.dispatchBubblingEvent(EventHandlerManager.java:191)

     at com.sun.javafx.event.CompositeEventDispatcher.dispatchBubblingEvent(CompositeEventDispatcher.java:59)

     at com.sun.javafx.event.BasicEventDispatcher.dispatchEvent(BasicEventDispatcher.java:58)

     at com.sun.javafx.event.EventDispatchChainImpl.dispatchEvent(EventDispatchChainImpl.java:114)

     at com.sun.javafx.event.BasicEventDispatcher.dispatchEvent(BasicEventDispatcher.java:56)

     at com.sun.javafx.event.EventDispatchChainImpl.dispatchEvent(EventDispatchChainImpl.java:114)

     at com.sun.javafx.event.BasicEventDispatcher.dispatchEvent(BasicEventDispatcher.java:56)

     at com.sun.javafx.event.EventDispatchChainImpl.dispatchEvent(EventDispatchChainImpl.java:114)

     at com.sun.javafx.event.EventUtil.fireEventImpl(EventUtil.java:74)

     at com.sun.javafx.event.EventUtil.fireEvent(EventUtil.java:49)

     at javafx.event.Event.fireEvent(Event.java:198)

     at javafx.scene.Node.fireEvent(Node.java:8889)

     at javafx.scene.control.Button.fire(Button.java:203)

     at com.sun.javafx.scene.control.behavior.ButtonBehavior.mouseReleased(ButtonBehavior.java:208)

     at com.sun.javafx.scene.control.inputmap.InputMap.handle(InputMap.java:274)

     at com.sun.javafx.event.CompositeEventHandler$NormalEventHandlerRecord.handleBubblingEvent(CompositeEventHandler.java:247)

     at com.sun.javafx.event.CompositeEventHandler.dispatchBubblingEvent(CompositeEventHandler.java:80)

     at com.sun.javafx.event.EventHandlerManager.dispatchBubblingEvent(EventHandlerManager.java:234)

     at com.sun.javafx.event.EventHandlerManager.dispatchBubblingEvent(EventHandlerManager.java:191)

     at com.sun.javafx.event.CompositeEventDispatcher.dispatchBubblingEvent(CompositeEventDispatcher.java:59)

     at com.sun.javafx.event.BasicEventDispatcher.dispatchEvent(BasicEventDispatcher.java:58)

     at com.sun.javafx.event.EventDispatchChainImpl.dispatchEvent(EventDispatchChainImpl.java:114)

     at com.sun.javafx.event.BasicEventDispatcher.dispatchEvent(BasicEventDispatcher.java:56)

     at com.sun.javafx.event.EventDispatchChainImpl.dispatchEvent(EventDispatchChainImpl.java:114)

     at com.sun.javafx.event.BasicEventDispatcher.dispatchEvent(BasicEventDispatcher.java:56)

     at com.sun.javafx.event.EventDispatchChainImpl.dispatchEvent(EventDispatchChainImpl.java:114)

     at com.sun.javafx.event.EventUtil.fireEventImpl(EventUtil.java:74)

     at com.sun.javafx.event.EventUtil.fireEvent(EventUtil.java:54)

     at javafx.event.Event.fireEvent(Event.java:198)

     at javafx.scene.Scene$MouseHandler.process(Scene.java:3856)

     at javafx.scene.Scene.processMouseEvent(Scene.java:1851)

     at javafx.scene.Scene$ScenePeerListener.mouseEvent(Scene.java:2584)

     at com.sun.javafx.tk.quantum.GlassViewEventHandler$MouseEventNotification.run(GlassViewEventHandler.java:409)

     at com.sun.javafx.tk.quantum.GlassViewEventHandler$MouseEventNotification.run(GlassViewEventHandler.java:299)

     at java.base/java.security.AccessController.doPrivileged(Native Method)

     at com.sun.javafx.tk.quantum.GlassViewEventHandler.lambda$handleMouseEvent$2(GlassViewEventHandler.java:447)

     at com.sun.javafx.tk.quantum.QuantumToolkit.runWithoutRenderLock(QuantumToolkit.java:412)

     at com.sun.javafx.tk.quantum.GlassViewEventHandler.handleMouseEvent(GlassViewEventHandler.java:446)

     at com.sun.glass.ui.View.handleMouseEvent(View.java:556)

     at com.sun.glass.ui.View.notifyMouse(View.java:942)

     at com.sun.glass.ui.win.WinApplication._runLoop(Native Method)

     at com.sun.glass.ui.win.WinApplication.lambda$runLoop$3(WinApplication.java:174)

     at java.base/java.lang.Thread.run(Thread.java:829)

 Caused by: java.lang.OutOfMemoryError

     at java.base/jdk.internal.reflect.GeneratedConstructorAccessor3.newInstance(Unknown Source)

     at java.base/jdk.internal.reflect.DelegatingConstructorAccessorImpl.newInstance(DelegatingConstructorAccessorImpl.java:45)

     at java.base/java.lang.reflect.Constructor.newInstance(Constructor.java:490)

     at java.base/java.util.concurrent.ForkJoinTask.getThrowableException(ForkJoinTask.java:603)

     ... 49 more

 Caused by: java.lang.OutOfMemoryError: Java heap space

 java.util.concurrent.ExecutionException: java.lang.OutOfMemoryError

     at java.base/java.util.concurrent.ForkJoinTask.get(ForkJoinTask.java:1006)

     at fr.univ_nantes.codex_processor.gui.DatasetExtendedDepthOfFieldProcessor.run(DatasetExtendedDepthOfFieldProcessor.java:69)

     at fr.univ_nantes.codex_processor.gui.MainController$1.handle(MainController.java:96)

     at fr.univ_nantes.codex_processor.gui.MainController$1.handle(MainController.java:64)

     at com.sun.javafx.event.CompositeEventHandler.dispatchBubblingEvent(CompositeEventHandler.java:86)

     at com.sun.javafx.event.EventHandlerManager.dispatchBubblingEvent(EventHandlerManager.java:234)

     at com.sun.javafx.event.EventHandlerManager.dispatchBubblingEvent(EventHandlerManager.java:191)

     at com.sun.javafx.event.CompositeEventDispatcher.dispatchBubblingEvent(CompositeEventDispatcher.java:59)

     at com.sun.javafx.event.BasicEventDispatcher.dispatchEvent(BasicEventDispatcher.java:58)

     at com.sun.javafx.event.EventDispatchChainImpl.dispatchEvent(EventDispatchChainImpl.java:114)

     at com.sun.javafx.event.BasicEventDispatcher.dispatchEvent(BasicEventDispatcher.java:56)

     at com.sun.javafx.event.EventDispatchChainImpl.dispatchEvent(EventDispatchChainImpl.java:114)

     at com.sun.javafx.event.BasicEventDispatcher.dispatchEvent(BasicEventDispatcher.java:56)

     at com.sun.javafx.event.EventDispatchChainImpl.dispatchEvent(EventDispatchChainImpl.java:114)

     at com.sun.javafx.event.EventUtil.fireEventImpl(EventUtil.java:74)

     at com.sun.javafx.event.EventUtil.fireEvent(EventUtil.java:49)

     at javafx.event.Event.fireEvent(Event.java:198)

     at javafx.scene.Node.fireEvent(Node.java:8889)

     at javafx.scene.control.Button.fire(Button.java:203)

     at com.sun.javafx.scene.control.behavior.ButtonBehavior.mouseReleased(ButtonBehavior.java:208)

     at com.sun.javafx.scene.control.inputmap.InputMap.handle(InputMap.java:274)

     at com.sun.javafx.event.CompositeEventHandler$NormalEventHandlerRecord.handleBubblingEvent(CompositeEventHandler.java:247)

     at com.sun.javafx.event.CompositeEventHandler.dispatchBubblingEvent(CompositeEventHandler.java:80)

     at com.sun.javafx.event.EventHandlerManager.dispatchBubblingEvent(EventHandlerManager.java:234)

     at com.sun.javafx.event.EventHandlerManager.dispatchBubblingEvent(EventHandlerManager.java:191)

     at com.sun.javafx.event.CompositeEventDispatcher.dispatchBubblingEvent(CompositeEventDispatcher.java:59)

     at com.sun.javafx.event.BasicEventDispatcher.dispatchEvent(BasicEventDispatcher.java:58)

     at com.sun.javafx.event.EventDispatchChainImpl.dispatchEvent(EventDispatchChainImpl.java:114)

     at com.sun.javafx.event.BasicEventDispatcher.dispatchEvent(BasicEventDispatcher.java:56)

     at com.sun.javafx.event.EventDispatchChainImpl.dispatchEvent(EventDispatchChainImpl.java:114)

     at com.sun.javafx.event.BasicEventDispatcher.dispatchEvent(BasicEventDispatcher.java:56)

     at com.sun.javafx.event.EventDispatchChainImpl.dispatchEvent(EventDispatchChainImpl.java:114)

     at com.sun.javafx.event.EventUtil.fireEventImpl(EventUtil.java:74)

     at com.sun.javafx.event.EventUtil.fireEvent(EventUtil.java:54)

     at javafx.event.Event.fireEvent(Event.java:198)

     at javafx.scene.Scene$MouseHandler.process(Scene.java:3856)

     at javafx.scene.Scene.processMouseEvent(Scene.java:1851)

     at javafx.scene.Scene$ScenePeerListener.mouseEvent(Scene.java:2584)

     at com.sun.javafx.tk.quantum.GlassViewEventHandler$MouseEventNotification.run(GlassViewEventHandler.java:409)

     at com.sun.javafx.tk.quantum.GlassViewEventHandler$MouseEventNotification.run(GlassViewEventHandler.java:299)

     at java.base/java.security.AccessController.doPrivileged(Native Method)

     at com.sun.javafx.tk.quantum.GlassViewEventHandler.lambda$handleMouseEvent$2(GlassViewEventHandler.java:447)

     at com.sun.javafx.tk.quantum.QuantumToolkit.runWithoutRenderLock(QuantumToolkit.java:412)

     at com.sun.javafx.tk.quantum.GlassViewEventHandler.handleMouseEvent(GlassViewEventHandler.java:446)

     at com.sun.glass.ui.View.handleMouseEvent(View.java:556)

     at com.sun.glass.ui.View.notifyMouse(View.java:942)

     at com.sun.glass.ui.win.WinApplication._runLoop(Native Method)

     at com.sun.glass.ui.win.WinApplication.lambda$runLoop$3(WinApplication.java:174)

     at java.base/java.lang.Thread.run(Thread.java:829)

 Caused by: java.lang.OutOfMemoryError

     at java.base/jdk.internal.reflect.GeneratedConstructorAccessor3.newInstance(Unknown Source)

     at java.base/jdk.internal.reflect.DelegatingConstructorAccessorImpl.newInstance(DelegatingConstructorAccessorImpl.java:45)

     at java.base/java.lang.reflect.Constructor.newInstance(Constructor.java:490)

     at java.base/java.util.concurrent.ForkJoinTask.getThrowableException(ForkJoinTask.java:603)

     ... 49 more

 Caused by: java.lang.OutOfMemoryError: Java heap space

 java.util.concurrent.ExecutionException: java.lang.OutOfMemoryError

     at java.base/java.util.concurrent.ForkJoinTask.get(ForkJoinTask.java:1006)

     at fr.univ_nantes.codex_processor.gui.DatasetExtendedDepthOfFieldProcessor.run(DatasetExtendedDepthOfFieldProcessor.java:69)

     at fr.univ_nantes.codex_processor.gui.MainController$1.handle(MainController.java:96)

     at fr.univ_nantes.codex_processor.gui.MainController$1.handle(MainController.java:64)

     at com.sun.javafx.event.CompositeEventHandler.dispatchBubblingEvent(CompositeEventHandler.java:86)

     at com.sun.javafx.event.EventHandlerManager.dispatchBubblingEvent(EventHandlerManager.java:234)

     at com.sun.javafx.event.EventHandlerManager.dispatchBubblingEvent(EventHandlerManager.java:191)

     at com.sun.javafx.event.CompositeEventDispatcher.dispatchBubblingEvent(CompositeEventDispatcher.java:59)

     at com.sun.javafx.event.BasicEventDispatcher.dispatchEvent(BasicEventDispatcher.java:58)

     at com.sun.javafx.event.EventDispatchChainImpl.dispatchEvent(EventDispatchChainImpl.java:114)

     at com.sun.javafx.event.BasicEventDispatcher.dispatchEvent(BasicEventDispatcher.java:56)

     at com.sun.javafx.event.EventDispatchChainImpl.dispatchEvent(EventDispatchChainImpl.java:114)

     at com.sun.javafx.event.BasicEventDispatcher.dispatchEvent(BasicEventDispatcher.java:56)

     at com.sun.javafx.event.EventDispatchChainImpl.dispatchEvent(EventDispatchChainImpl.java:114)

     at com.sun.javafx.event.EventUtil.fireEventImpl(EventUtil.java:74)

     at com.sun.javafx.event.EventUtil.fireEvent(EventUtil.java:49)

     at javafx.event.Event.fireEvent(Event.java:198)

     at javafx.scene.Node.fireEvent(Node.java:8889)

     at javafx.scene.control.Button.fire(Button.java:203)

     at com.sun.javafx.scene.control.behavior.ButtonBehavior.mouseReleased(ButtonBehavior.java:208)

     at com.sun.javafx.scene.control.inputmap.InputMap.handle(InputMap.java:274)

     at com.sun.javafx.event.CompositeEventHandler$NormalEventHandlerRecord.handleBubblingEvent(CompositeEventHandler.java:247)

     at com.sun.javafx.event.CompositeEventHandler.dispatchBubblingEvent(CompositeEventHandler.java:80)

     at com.sun.javafx.event.EventHandlerManager.dispatchBubblingEvent(EventHandlerManager.java:234)

     at com.sun.javafx.event.EventHandlerManager.dispatchBubblingEvent(EventHandlerManager.java:191)

     at com.sun.javafx.event.CompositeEventDispatcher.dispatchBubblingEvent(CompositeEventDispatcher.java:59)

     at com.sun.javafx.event.BasicEventDispatcher.dispatchEvent(BasicEventDispatcher.java:58)

     at com.sun.javafx.event.EventDispatchChainImpl.dispatchEvent(EventDispatchChainImpl.java:114)

     at com.sun.javafx.event.BasicEventDispatcher.dispatchEvent(BasicEventDispatcher.java:56)

     at com.sun.javafx.event.EventDispatchChainImpl.dispatchEvent(EventDispatchChainImpl.java:114)

     at com.sun.javafx.event.BasicEventDispatcher.dispatchEvent(BasicEventDispatcher.java:56)

     at com.sun.javafx.event.EventDispatchChainImpl.dispatchEvent(EventDispatchChainImpl.java:114)

     at com.sun.javafx.event.EventUtil.fireEventImpl(EventUtil.java:74)

     at com.sun.javafx.event.EventUtil.fireEvent(EventUtil.java:54)

     at javafx.event.Event.fireEvent(Event.java:198)

     at javafx.scene.Scene$MouseHandler.process(Scene.java:3856)

     at javafx.scene.Scene.processMouseEvent(Scene.java:1851)

     at javafx.scene.Scene$ScenePeerListener.mouseEvent(Scene.java:2584)

     at com.sun.javafx.tk.quantum.GlassViewEventHandler$MouseEventNotification.run(GlassViewEventHandler.java:409)

     at com.sun.javafx.tk.quantum.GlassViewEventHandler$MouseEventNotification.run(GlassViewEventHandler.java:299)

     at java.base/java.security.AccessController.doPrivileged(Native Method)

     at com.sun.javafx.tk.quantum.GlassViewEventHandler.lambda$handleMouseEvent$2(GlassViewEventHandler.java:447)

     at com.sun.javafx.tk.quantum.QuantumToolkit.runWithoutRenderLock(QuantumToolkit.java:412)

     at com.sun.javafx.tk.quantum.GlassViewEventHandler.handleMouseEvent(GlassViewEventHandler.java:446)

     at com.sun.glass.ui.View.handleMouseEvent(View.java:556)

     at com.sun.glass.ui.View.notifyMouse(View.java:942)

     at com.sun.glass.ui.win.WinApplication._runLoop(Native Method)

     at com.sun.glass.ui.win.WinApplication.lambda$runLoop$3(WinApplication.java:174)

     at java.base/java.lang.Thread.run(Thread.java:829)

 Caused by: java.lang.OutOfMemoryError

     at java.base/jdk.internal.reflect.GeneratedConstructorAccessor3.newInstance(Unknown Source)

     at java.base/jdk.internal.reflect.DelegatingConstructorAccessorImpl.newInstance(DelegatingConstructorAccessorImpl.java:45)

     at java.base/java.lang.reflect.Constructor.newInstance(Constructor.java:490)

     at java.base/java.util.concurrent.ForkJoinTask.getThrowableException(ForkJoinTask.java:603)

     ... 49 more

 Caused by: java.lang.OutOfMemoryError: Java heap space

 java.util.concurrent.ExecutionException: java.lang.OutOfMemoryError

     at java.base/java.util.concurrent.ForkJoinTask.get(ForkJoinTask.java:1006)

     at fr.univ_nantes.codex_processor.gui.DatasetExtendedDepthOfFieldProcessor.run(DatasetExtendedDepthOfFieldProcessor.java:69)

     at fr.univ_nantes.codex_processor.gui.MainController$1.handle(MainController.java:96)

     at fr.univ_nantes.codex_processor.gui.MainController$1.handle(MainController.java:64)

     at com.sun.javafx.event.CompositeEventHandler.dispatchBubblingEvent(CompositeEventHandler.java:86)

     at com.sun.javafx.event.EventHandlerManager.dispatchBubblingEvent(EventHandlerManager.java:234)

     at com.sun.javafx.event.EventHandlerManager.dispatchBubblingEvent(EventHandlerManager.java:191)

     at com.sun.javafx.event.CompositeEventDispatcher.dispatchBubblingEvent(CompositeEventDispatcher.java:59)

     at com.sun.javafx.event.BasicEventDispatcher.dispatchEvent(BasicEventDispatcher.java:58)

     at com.sun.javafx.event.EventDispatchChainImpl.dispatchEvent(EventDispatchChainImpl.java:114)

     at com.sun.javafx.event.BasicEventDispatcher.dispatchEvent(BasicEventDispatcher.java:56)

     at com.sun.javafx.event.EventDispatchChainImpl.dispatchEvent(EventDispatchChainImpl.java:114)

     at com.sun.javafx.event.BasicEventDispatcher.dispatchEvent(BasicEventDispatcher.java:56)

     at com.sun.javafx.event.EventDispatchChainImpl.dispatchEvent(EventDispatchChainImpl.java:114)

     at com.sun.javafx.event.EventUtil.fireEventImpl(EventUtil.java:74)

     at com.sun.javafx.event.EventUtil.fireEvent(EventUtil.java:49)

     at javafx.event.Event.fireEvent(Event.java:198)

     at javafx.scene.Node.fireEvent(Node.java:8889)

     at javafx.scene.control.Button.fire(Button.java:203)

     at com.sun.javafx.scene.control.behavior.ButtonBehavior.mouseReleased(ButtonBehavior.java:208)

     at com.sun.javafx.scene.control.inputmap.InputMap.handle(InputMap.java:274)

     at com.sun.javafx.event.CompositeEventHandler$NormalEventHandlerRecord.handleBubblingEvent(CompositeEventHandler.java:247)

     at com.sun.javafx.event.CompositeEventHandler.dispatchBubblingEvent(CompositeEventHandler.java:80)

     at com.sun.javafx.event.EventHandlerManager.dispatchBubblingEvent(EventHandlerManager.java:234)

     at com.sun.javafx.event.EventHandlerManager.dispatchBubblingEvent(EventHandlerManager.java:191)

     at com.sun.javafx.event.CompositeEventDispatcher.dispatchBubblingEvent(CompositeEventDispatcher.java:59)

     at com.sun.javafx.event.BasicEventDispatcher.dispatchEvent(BasicEventDispatcher.java:58)

     at com.sun.javafx.event.EventDispatchChainImpl.dispatchEvent(EventDispatchChainImpl.java:114)

     at com.sun.javafx.event.BasicEventDispatcher.dispatchEvent(BasicEventDispatcher.java:56)

     at com.sun.javafx.event.EventDispatchChainImpl.dispatchEvent(EventDispatchChainImpl.java:114)

     at com.sun.javafx.event.BasicEventDispatcher.dispatchEvent(BasicEventDispatcher.java:56)

     at com.sun.javafx.event.EventDispatchChainImpl.dispatchEvent(EventDispatchChainImpl.java:114)

     at com.sun.javafx.event.EventUtil.fireEventImpl(EventUtil.java:74)

     at com.sun.javafx.event.EventUtil.fireEvent(EventUtil.java:54)

     at javafx.event.Event.fireEvent(Event.java:198)

     at javafx.scene.Scene$MouseHandler.process(Scene.java:3856)

     at javafx.scene.Scene.processMouseEvent(Scene.java:1851)

     at javafx.scene.Scene$ScenePeerListener.mouseEvent(Scene.java:2584)

     at com.sun.javafx.tk.quantum.GlassViewEventHandler$MouseEventNotification.run(GlassViewEventHandler.java:409)

     at com.sun.javafx.tk.quantum.GlassViewEventHandler$MouseEventNotification.run(GlassViewEventHandler.java:299)

     at java.base/java.security.AccessController.doPrivileged(Native Method)

     at com.sun.javafx.tk.quantum.GlassViewEventHandler.lambda$handleMouseEvent$2(GlassViewEventHandler.java:447)

     at com.sun.javafx.tk.quantum.QuantumToolkit.runWithoutRenderLock(QuantumToolkit.java:412)

     at com.sun.javafx.tk.quantum.GlassViewEventHandler.handleMouseEvent(GlassViewEventHandler.java:446)

     at com.sun.glass.ui.View.handleMouseEvent(View.java:556)

     at com.sun.glass.ui.View.notifyMouse(View.java:942)

     at com.sun.glass.ui.win.WinApplication._runLoop(Native Method)

     at com.sun.glass.ui.win.WinApplication.lambda$runLoop$3(WinApplication.java:174)

     at java.base/java.lang.Thread.run(Thread.java:829)

 Caused by: java.lang.OutOfMemoryError

     at java.base/jdk.internal.reflect.GeneratedConstructorAccessor3.newInstance(Unknown Source)

     at java.base/jdk.internal.reflect.DelegatingConstructorAccessorImpl.newInstance(DelegatingConstructorAccessorImpl.java:45)

     at java.base/java.lang.reflect.Constructor.newInstance(Constructor.java:490)

     at java.base/java.util.concurrent.ForkJoinTask.getThrowableException(ForkJoinTask.java:603)

     ... 49 more

 Caused by: java.lang.OutOfMemoryError: Java heap space

 java.util.concurrent.ExecutionException: java.lang.OutOfMemoryError

     at java.base/java.util.concurrent.ForkJoinTask.get(ForkJoinTask.java:1006)

     at fr.univ_nantes.codex_processor.gui.DatasetExtendedDepthOfFieldProcessor.run(DatasetExtendedDepthOfFieldProcessor.java:69)

     at fr.univ_nantes.codex_processor.gui.MainController$1.handle(MainController.java:96)

     at fr.univ_nantes.codex_processor.gui.MainController$1.handle(MainController.java:64)

     at com.sun.javafx.event.CompositeEventHandler.dispatchBubblingEvent(CompositeEventHandler.java:86)

     at com.sun.javafx.event.EventHandlerManager.dispatchBubblingEvent(EventHandlerManager.java:234)

     at com.sun.javafx.event.EventHandlerManager.dispatchBubblingEvent(EventHandlerManager.java:191)

     at com.sun.javafx.event.CompositeEventDispatcher.dispatchBubblingEvent(CompositeEventDispatcher.java:59)

     at com.sun.javafx.event.BasicEventDispatcher.dispatchEvent(BasicEventDispatcher.java:58)

     at com.sun.javafx.event.EventDispatchChainImpl.dispatchEvent(EventDispatchChainImpl.java:114)

     at com.sun.javafx.event.BasicEventDispatcher.dispatchEvent(BasicEventDispatcher.java:56)

     at com.sun.javafx.event.EventDispatchChainImpl.dispatchEvent(EventDispatchChainImpl.java:114)

     at com.sun.javafx.event.BasicEventDispatcher.dispatchEvent(BasicEventDispatcher.java:56)

     at com.sun.javafx.event.EventDispatchChainImpl.dispatchEvent(EventDispatchChainImpl.java:114)

     at com.sun.javafx.event.EventUtil.fireEventImpl(EventUtil.java:74)

     at com.sun.javafx.event.EventUtil.fireEvent(EventUtil.java:49)

     at javafx.event.Event.fireEvent(Event.java:198)

     at javafx.scene.Node.fireEvent(Node.java:8889)

     at javafx.scene.control.Button.fire(Button.java:203)

     at com.sun.javafx.scene.control.behavior.ButtonBehavior.mouseReleased(ButtonBehavior.java:208)

     at com.sun.javafx.scene.control.inputmap.InputMap.handle(InputMap.java:274)

     at com.sun.javafx.event.CompositeEventHandler$NormalEventHandlerRecord.handleBubblingEvent(CompositeEventHandler.java:247)

     at com.sun.javafx.event.CompositeEventHandler.dispatchBubblingEvent(CompositeEventHandler.java:80)

     at com.sun.javafx.event.EventHandlerManager.dispatchBubblingEvent(EventHandlerManager.java:234)

     at com.sun.javafx.event.EventHandlerManager.dispatchBubblingEvent(EventHandlerManager.java:191)

     at com.sun.javafx.event.CompositeEventDispatcher.dispatchBubblingEvent(CompositeEventDispatcher.java:59)

     at com.sun.javafx.event.BasicEventDispatcher.dispatchEvent(BasicEventDispatcher.java:58)

     at com.sun.javafx.event.EventDispatchChainImpl.dispatchEvent(EventDispatchChainImpl.java:114)

     at com.sun.javafx.event.BasicEventDispatcher.dispatchEvent(BasicEventDispatcher.java:56)

     at com.sun.javafx.event.EventDispatchChainImpl.dispatchEvent(EventDispatchChainImpl.java:114)

     at com.sun.javafx.event.BasicEventDispatcher.dispatchEvent(BasicEventDispatcher.java:56)

     at com.sun.javafx.event.EventDispatchChainImpl.dispatchEvent(EventDispatchChainImpl.java:114)

     at com.sun.javafx.event.EventUtil.fireEventImpl(EventUtil.java:74)

     at com.sun.javafx.event.EventUtil.fireEvent(EventUtil.java:54)

     at javafx.event.Event.fireEvent(Event.java:198)

     at javafx.scene.Scene$MouseHandler.process(Scene.java:3856)

     at javafx.scene.Scene.processMouseEvent(Scene.java:1851)

     at javafx.scene.Scene$ScenePeerListener.mouseEvent(Scene.java:2584)

     at com.sun.javafx.tk.quantum.GlassViewEventHandler$MouseEventNotification.run(GlassViewEventHandler.java:409)

     at com.sun.javafx.tk.quantum.GlassViewEventHandler$MouseEventNotification.run(GlassViewEventHandler.java:299)

     at java.base/java.security.AccessController.doPrivileged(Native Method)

     at com.sun.javafx.tk.quantum.GlassViewEventHandler.lambda$handleMouseEvent$2(GlassViewEventHandler.java:447)

     at com.sun.javafx.tk.quantum.QuantumToolkit.runWithoutRenderLock(QuantumToolkit.java:412)

     at com.sun.javafx.tk.quantum.GlassViewEventHandler.handleMouseEvent(GlassViewEventHandler.java:446)

     at com.sun.glass.ui.View.handleMouseEvent(View.java:556)

     at com.sun.glass.ui.View.notifyMouse(View.java:942)

     at com.sun.glass.ui.win.WinApplication._runLoop(Native Method)

     at com.sun.glass.ui.win.WinApplication.lambda$runLoop$3(WinApplication.java:174)

     at java.base/java.lang.Thread.run(Thread.java:829)

 Caused by: java.lang.OutOfMemoryError

     at java.base/jdk.internal.reflect.GeneratedConstructorAccessor3.newInstance(Unknown Source)

     at java.base/jdk.internal.reflect.DelegatingConstructorAccessorImpl.newInstance(DelegatingConstructorAccessorImpl.java:45)

     at java.base/java.lang.reflect.Constructor.newInstance(Constructor.java:490)

     at java.base/java.util.concurrent.ForkJoinTask.getThrowableException(ForkJoinTask.java:603)

     ... 49 more

 Caused by: java.lang.OutOfMemoryError: Java heap space

 java.util.concurrent.ExecutionException: java.lang.OutOfMemoryError

     at java.base/java.util.concurrent.ForkJoinTask.get(ForkJoinTask.java:1006)

     at fr.univ_nantes.codex_processor.gui.DatasetExtendedDepthOfFieldProcessor.run(DatasetExtendedDepthOfFieldProcessor.java:69)

     at fr.univ_nantes.codex_processor.gui.MainController$1.handle(MainController.java:96)

     at fr.univ_nantes.codex_processor.gui.MainController$1.handle(MainController.java:64)

     at com.sun.javafx.event.CompositeEventHandler.dispatchBubblingEvent(CompositeEventHandler.java:86)

     at com.sun.javafx.event.EventHandlerManager.dispatchBubblingEvent(EventHandlerManager.java:234)

     at com.sun.javafx.event.EventHandlerManager.dispatchBubblingEvent(EventHandlerManager.java:191)

     at com.sun.javafx.event.CompositeEventDispatcher.dispatchBubblingEvent(CompositeEventDispatcher.java:59)

     at com.sun.javafx.event.BasicEventDispatcher.dispatchEvent(BasicEventDispatcher.java:58)

     at com.sun.javafx.event.EventDispatchChainImpl.dispatchEvent(EventDispatchChainImpl.java:114)

     at com.sun.javafx.event.BasicEventDispatcher.dispatchEvent(BasicEventDispatcher.java:56)

     at com.sun.javafx.event.EventDispatchChainImpl.dispatchEvent(EventDispatchChainImpl.java:114)

     at com.sun.javafx.event.BasicEventDispatcher.dispatchEvent(BasicEventDispatcher.java:56)

     at com.sun.javafx.event.EventDispatchChainImpl.dispatchEvent(EventDispatchChainImpl.java:114)

     at com.sun.javafx.event.EventUtil.fireEventImpl(EventUtil.java:74)

     at com.sun.javafx.event.EventUtil.fireEvent(EventUtil.java:49)

     at javafx.event.Event.fireEvent(Event.java:198)

     at javafx.scene.Node.fireEvent(Node.java:8889)

     at javafx.scene.control.Button.fire(Button.java:203)

     at com.sun.javafx.scene.control.behavior.ButtonBehavior.mouseReleased(ButtonBehavior.java:208)

     at com.sun.javafx.scene.control.inputmap.InputMap.handle(InputMap.java:274)

     at com.sun.javafx.event.CompositeEventHandler$NormalEventHandlerRecord.handleBubblingEvent(CompositeEventHandler.java:247)

     at com.sun.javafx.event.CompositeEventHandler.dispatchBubblingEvent(CompositeEventHandler.java:80)

     at com.sun.javafx.event.EventHandlerManager.dispatchBubblingEvent(EventHandlerManager.java:234)

     at com.sun.javafx.event.EventHandlerManager.dispatchBubblingEvent(EventHandlerManager.java:191)

     at com.sun.javafx.event.CompositeEventDispatcher.dispatchBubblingEvent(CompositeEventDispatcher.java:59)

     at com.sun.javafx.event.BasicEventDispatcher.dispatchEvent(BasicEventDispatcher.java:58)

     at com.sun.javafx.event.EventDispatchChainImpl.dispatchEvent(EventDispatchChainImpl.java:114)

     at com.sun.javafx.event.BasicEventDispatcher.dispatchEvent(BasicEventDispatcher.java:56)

     at com.sun.javafx.event.EventDispatchChainImpl.dispatchEvent(EventDispatchChainImpl.java:114)

     at com.sun.javafx.event.BasicEventDispatcher.dispatchEvent(BasicEventDispatcher.java:56)

     at com.sun.javafx.event.EventDispatchChainImpl.dispatchEvent(EventDispatchChainImpl.java:114)

     at com.sun.javafx.event.EventUtil.fireEventImpl(EventUtil.java:74)

     at com.sun.javafx.event.EventUtil.fireEvent(EventUtil.java:54)

     at javafx.event.Event.fireEvent(Event.java:198)

     at javafx.scene.Scene$MouseHandler.process(Scene.java:3856)

     at javafx.scene.Scene.processMouseEvent(Scene.java:1851)

     at javafx.scene.Scene$ScenePeerListener.mouseEvent(Scene.java:2584)

     at com.sun.javafx.tk.quantum.GlassViewEventHandler$MouseEventNotification.run(GlassViewEventHandler.java:409)

     at com.sun.javafx.tk.quantum.GlassViewEventHandler$MouseEventNotification.run(GlassViewEventHandler.java:299)

     at java.base/java.security.AccessController.doPrivileged(Native Method)

     at com.sun.javafx.tk.quantum.GlassViewEventHandler.lambda$handleMouseEvent$2(GlassViewEventHandler.java:447)

     at com.sun.javafx.tk.quantum.QuantumToolkit.runWithoutRenderLock(QuantumToolkit.java:412)

     at com.sun.javafx.tk.quantum.GlassViewEventHandler.handleMouseEvent(GlassViewEventHandler.java:446)

     at com.sun.glass.ui.View.handleMouseEvent(View.java:556)

     at com.sun.glass.ui.View.notifyMouse(View.java:942)

     at com.sun.glass.ui.win.WinApplication._runLoop(Native Method)

     at com.sun.glass.ui.win.WinApplication.lambda$runLoop$3(WinApplication.java:174)

     at java.base/java.lang.Thread.run(Thread.java:829)

 Caused by: java.lang.OutOfMemoryError

     at java.base/jdk.internal.reflect.GeneratedConstructorAccessor3.newInstance(Unknown Source)

     at java.base/jdk.internal.reflect.DelegatingConstructorAccessorImpl.newInstance(DelegatingConstructorAccessorImpl.java:45)

     at java.base/java.lang.reflect.Constructor.newInstance(Constructor.java:490)

     at java.base/java.util.concurrent.ForkJoinTask.getThrowableException(ForkJoinTask.java:603)

     ... 49 more

 Caused by: java.lang.OutOfMemoryError: Java heap space

 java.util.concurrent.ExecutionException: java.lang.OutOfMemoryError

     at java.base/java.util.concurrent.ForkJoinTask.get(ForkJoinTask.java:1006)

     at fr.univ_nantes.codex_processor.gui.DatasetExtendedDepthOfFieldProcessor.run(DatasetExtendedDepthOfFieldProcessor.java:69)

     at fr.univ_nantes.codex_processor.gui.MainController$1.handle(MainController.java:96)

     at fr.univ_nantes.codex_processor.gui.MainController$1.handle(MainController.java:64)

     at com.sun.javafx.event.CompositeEventHandler.dispatchBubblingEvent(CompositeEventHandler.java:86)

     at com.sun.javafx.event.EventHandlerManager.dispatchBubblingEvent(EventHandlerManager.java:234)

     at com.sun.javafx.event.EventHandlerManager.dispatchBubblingEvent(EventHandlerManager.java:191)

     at com.sun.javafx.event.CompositeEventDispatcher.dispatchBubblingEvent(CompositeEventDispatcher.java:59)

     at com.sun.javafx.event.BasicEventDispatcher.dispatchEvent(BasicEventDispatcher.java:58)

     at com.sun.javafx.event.EventDispatchChainImpl.dispatchEvent(EventDispatchChainImpl.java:114)

     at com.sun.javafx.event.BasicEventDispatcher.dispatchEvent(BasicEventDispatcher.java:56)

     at com.sun.javafx.event.EventDispatchChainImpl.dispatchEvent(EventDispatchChainImpl.java:114)

     at com.sun.javafx.event.BasicEventDispatcher.dispatchEvent(BasicEventDispatcher.java:56)

     at com.sun.javafx.event.EventDispatchChainImpl.dispatchEvent(EventDispatchChainImpl.java:114)

     at com.sun.javafx.event.EventUtil.fireEventImpl(EventUtil.java:74)

     at com.sun.javafx.event.EventUtil.fireEvent(EventUtil.java:49)

     at javafx.event.Event.fireEvent(Event.java:198)

     at javafx.scene.Node.fireEvent(Node.java:8889)

     at javafx.scene.control.Button.fire(Button.java:203)

     at com.sun.javafx.scene.control.behavior.ButtonBehavior.mouseReleased(ButtonBehavior.java:208)

     at com.sun.javafx.scene.control.inputmap.InputMap.handle(InputMap.java:274)

     at com.sun.javafx.event.CompositeEventHandler$NormalEventHandlerRecord.handleBubblingEvent(CompositeEventHandler.java:247)

     at com.sun.javafx.event.CompositeEventHandler.dispatchBubblingEvent(CompositeEventHandler.java:80)

     at com.sun.javafx.event.EventHandlerManager.dispatchBubblingEvent(EventHandlerManager.java:234)

     at com.sun.javafx.event.EventHandlerManager.dispatchBubblingEvent(EventHandlerManager.java:191)

     at com.sun.javafx.event.CompositeEventDispatcher.dispatchBubblingEvent(CompositeEventDispatcher.java:59)

     at com.sun.javafx.event.BasicEventDispatcher.dispatchEvent(BasicEventDispatcher.java:58)

     at com.sun.javafx.event.EventDispatchChainImpl.dispatchEvent(EventDispatchChainImpl.java:114)

     at com.sun.javafx.event.BasicEventDispatcher.dispatchEvent(BasicEventDispatcher.java:56)

     at com.sun.javafx.event.EventDispatchChainImpl.dispatchEvent(EventDispatchChainImpl.java:114)

     at com.sun.javafx.event.BasicEventDispatcher.dispatchEvent(BasicEventDispatcher.java:56)

     at com.sun.javafx.event.EventDispatchChainImpl.dispatchEvent(EventDispatchChainImpl.java:114)

     at com.sun.javafx.event.EventUtil.fireEventImpl(EventUtil.java:74)

     at com.sun.javafx.event.EventUtil.fireEvent(EventUtil.java:54)

     at javafx.event.Event.fireEvent(Event.java:198)

     at javafx.scene.Scene$MouseHandler.process(Scene.java:3856)

     at javafx.scene.Scene.processMouseEvent(Scene.java:1851)

     at javafx.scene.Scene$ScenePeerListener.mouseEvent(Scene.java:2584)

     at com.sun.javafx.tk.quantum.GlassViewEventHandler$MouseEventNotification.run(GlassViewEventHandler.java:409)

     at com.sun.javafx.tk.quantum.GlassViewEventHandler$MouseEventNotification.run(GlassViewEventHandler.java:299)

     at java.base/java.security.AccessController.doPrivileged(Native Method)

     at com.sun.javafx.tk.quantum.GlassViewEventHandler.lambda$handleMouseEvent$2(GlassViewEventHandler.java:447)

     at com.sun.javafx.tk.quantum.QuantumToolkit.runWithoutRenderLock(QuantumToolkit.java:412)

     at com.sun.javafx.tk.quantum.GlassViewEventHandler.handleMouseEvent(GlassViewEventHandler.java:446)

     at com.sun.glass.ui.View.handleMouseEvent(View.java:556)

     at com.sun.glass.ui.View.notifyMouse(View.java:942)

     at com.sun.glass.ui.win.WinApplication._runLoop(Native Method)

     at com.sun.glass.ui.win.WinApplication.lambda$runLoop$3(WinApplication.java:174)

     at java.base/java.lang.Thread.run(Thread.java:829)

 Caused by: java.lang.OutOfMemoryError

     at java.base/jdk.internal.reflect.GeneratedConstructorAccessor3.newInstance(Unknown Source)

     at java.base/jdk.internal.reflect.DelegatingConstructorAccessorImpl.newInstance(DelegatingConstructorAccessorImpl.java:45)

     at java.base/java.lang.reflect.Constructor.newInstance(Constructor.java:490)

     at java.base/java.util.concurrent.ForkJoinTask.getThrowableException(ForkJoinTask.java:603)

     ... 49 more

 Caused by: java.lang.OutOfMemoryError: Java heap space

 java.util.concurrent.ExecutionException: java.lang.OutOfMemoryError

     at java.base/java.util.concurrent.ForkJoinTask.get(ForkJoinTask.java:1006)

     at fr.univ_nantes.codex_processor.gui.DatasetExtendedDepthOfFieldProcessor.run(DatasetExtendedDepthOfFieldProcessor.java:69)

     at fr.univ_nantes.codex_processor.gui.MainController$1.handle(MainController.java:96)

     at fr.univ_nantes.codex_processor.gui.MainController$1.handle(MainController.java:64)

     at com.sun.javafx.event.CompositeEventHandler.dispatchBubblingEvent(CompositeEventHandler.java:86)

     at com.sun.javafx.event.EventHandlerManager.dispatchBubblingEvent(EventHandlerManager.java:234)

     at com.sun.javafx.event.EventHandlerManager.dispatchBubblingEvent(EventHandlerManager.java:191)

     at com.sun.javafx.event.CompositeEventDispatcher.dispatchBubblingEvent(CompositeEventDispatcher.java:59)

     at com.sun.javafx.event.BasicEventDispatcher.dispatchEvent(BasicEventDispatcher.java:58)

     at com.sun.javafx.event.EventDispatchChainImpl.dispatchEvent(EventDispatchChainImpl.java:114)

     at com.sun.javafx.event.BasicEventDispatcher.dispatchEvent(BasicEventDispatcher.java:56)

     at com.sun.javafx.event.EventDispatchChainImpl.dispatchEvent(EventDispatchChainImpl.java:114)

     at com.sun.javafx.event.BasicEventDispatcher.dispatchEvent(BasicEventDispatcher.java:56)

     at com.sun.javafx.event.EventDispatchChainImpl.dispatchEvent(EventDispatchChainImpl.java:114)

     at com.sun.javafx.event.EventUtil.fireEventImpl(EventUtil.java:74)

     at com.sun.javafx.event.EventUtil.fireEvent(EventUtil.java:49)

     at javafx.event.Event.fireEvent(Event.java:198)

     at javafx.scene.Node.fireEvent(Node.java:8889)

     at javafx.scene.control.Button.fire(Button.java:203)

     at com.sun.javafx.scene.control.behavior.ButtonBehavior.mouseReleased(ButtonBehavior.java:208)

     at com.sun.javafx.scene.control.inputmap.InputMap.handle(InputMap.java:274)

     at com.sun.javafx.event.CompositeEventHandler$NormalEventHandlerRecord.handleBubblingEvent(CompositeEventHandler.java:247)

     at com.sun.javafx.event.CompositeEventHandler.dispatchBubblingEvent(CompositeEventHandler.java:80)

     at com.sun.javafx.event.EventHandlerManager.dispatchBubblingEvent(EventHandlerManager.java:234)

     at com.sun.javafx.event.EventHandlerManager.dispatchBubblingEvent(EventHandlerManager.java:191)

     at com.sun.javafx.event.CompositeEventDispatcher.dispatchBubblingEvent(CompositeEventDispatcher.java:59)

     at com.sun.javafx.event.BasicEventDispatcher.dispatchEvent(BasicEventDispatcher.java:58)

     at com.sun.javafx.event.EventDispatchChainImpl.dispatchEvent(EventDispatchChainImpl.java:114)

     at com.sun.javafx.event.BasicEventDispatcher.dispatchEvent(BasicEventDispatcher.java:56)

     at com.sun.javafx.event.EventDispatchChainImpl.dispatchEvent(EventDispatchChainImpl.java:114)

     at com.sun.javafx.event.BasicEventDispatcher.dispatchEvent(BasicEventDispatcher.java:56)

     at com.sun.javafx.event.EventDispatchChainImpl.dispatchEvent(EventDispatchChainImpl.java:114)

     at com.sun.javafx.event.EventUtil.fireEventImpl(EventUtil.java:74)

     at com.sun.javafx.event.EventUtil.fireEvent(EventUtil.java:54)

     at javafx.event.Event.fireEvent(Event.java:198)

     at javafx.scene.Scene$MouseHandler.process(Scene.java:3856)

     at javafx.scene.Scene.processMouseEvent(Scene.java:1851)

     at javafx.scene.Scene$ScenePeerListener.mouseEvent(Scene.java:2584)

     at com.sun.javafx.tk.quantum.GlassViewEventHandler$MouseEventNotification.run(GlassViewEventHandler.java:409)

     at com.sun.javafx.tk.quantum.GlassViewEventHandler$MouseEventNotification.run(GlassViewEventHandler.java:299)

     at java.base/java.security.AccessController.doPrivileged(Native Method)

     at com.sun.javafx.tk.quantum.GlassViewEventHandler.lambda$handleMouseEvent$2(GlassViewEventHandler.java:447)

     at com.sun.javafx.tk.quantum.QuantumToolkit.runWithoutRenderLock(QuantumToolkit.java:412)

     at com.sun.javafx.tk.quantum.GlassViewEventHandler.handleMouseEvent(GlassViewEventHandler.java:446)

     at com.sun.glass.ui.View.handleMouseEvent(View.java:556)

     at com.sun.glass.ui.View.notifyMouse(View.java:942)

     at com.sun.glass.ui.win.WinApplication._runLoop(Native Method)

     at com.sun.glass.ui.win.WinApplication.lambda$runLoop$3(WinApplication.java:174)

     at java.base/java.lang.Thread.run(Thread.java:829)

 Caused by: java.lang.OutOfMemoryError

     at java.base/jdk.internal.reflect.GeneratedConstructorAccessor3.newInstance(Unknown Source)

     at java.base/jdk.internal.reflect.DelegatingConstructorAccessorImpl.newInstance(DelegatingConstructorAccessorImpl.java:45)

     at java.base/java.lang.reflect.Constructor.newInstance(Constructor.java:490)

     at java.base/java.util.concurrent.ForkJoinTask.getThrowableException(ForkJoinTask.java:603)

     ... 49 more

 Caused by: java.lang.OutOfMemoryError: Java heap space

 java.util.concurrent.ExecutionException: java.lang.OutOfMemoryError

     at java.base/java.util.concurrent.ForkJoinTask.get(ForkJoinTask.java:1006)

     at fr.univ_nantes.codex_processor.gui.DatasetExtendedDepthOfFieldProcessor.run(DatasetExtendedDepthOfFieldProcessor.java:69)

     at fr.univ_nantes.codex_processor.gui.MainController$1.handle(MainController.java:96)

     at fr.univ_nantes.codex_processor.gui.MainController$1.handle(MainController.java:64)

     at com.sun.javafx.event.CompositeEventHandler.dispatchBubblingEvent(CompositeEventHandler.java:86)

     at com.sun.javafx.event.EventHandlerManager.dispatchBubblingEvent(EventHandlerManager.java:234)

     at com.sun.javafx.event.EventHandlerManager.dispatchBubblingEvent(EventHandlerManager.java:191)

     at com.sun.javafx.event.CompositeEventDispatcher.dispatchBubblingEvent(CompositeEventDispatcher.java:59)

     at com.sun.javafx.event.BasicEventDispatcher.dispatchEvent(BasicEventDispatcher.java:58)

     at com.sun.javafx.event.EventDispatchChainImpl.dispatchEvent(EventDispatchChainImpl.java:114)

     at com.sun.javafx.event.BasicEventDispatcher.dispatchEvent(BasicEventDispatcher.java:56)

     at com.sun.javafx.event.EventDispatchChainImpl.dispatchEvent(EventDispatchChainImpl.java:114)

     at com.sun.javafx.event.BasicEventDispatcher.dispatchEvent(BasicEventDispatcher.java:56)

     at com.sun.javafx.event.EventDispatchChainImpl.dispatchEvent(EventDispatchChainImpl.java:114)

     at com.sun.javafx.event.EventUtil.fireEventImpl(EventUtil.java:74)

     at com.sun.javafx.event.EventUtil.fireEvent(EventUtil.java:49)

     at javafx.event.Event.fireEvent(Event.java:198)

     at javafx.scene.Node.fireEvent(Node.java:8889)

     at javafx.scene.control.Button.fire(Button.java:203)

     at com.sun.javafx.scene.control.behavior.ButtonBehavior.mouseReleased(ButtonBehavior.java:208)

     at com.sun.javafx.scene.control.inputmap.InputMap.handle(InputMap.java:274)

     at com.sun.javafx.event.CompositeEventHandler$NormalEventHandlerRecord.handleBubblingEvent(CompositeEventHandler.java:247)

     at com.sun.javafx.event.CompositeEventHandler.dispatchBubblingEvent(CompositeEventHandler.java:80)

     at com.sun.javafx.event.EventHandlerManager.dispatchBubblingEvent(EventHandlerManager.java:234)

     at com.sun.javafx.event.EventHandlerManager.dispatchBubblingEvent(EventHandlerManager.java:191)

     at com.sun.javafx.event.CompositeEventDispatcher.dispatchBubblingEvent(CompositeEventDispatcher.java:59)

     at com.sun.javafx.event.BasicEventDispatcher.dispatchEvent(BasicEventDispatcher.java:58)

     at com.sun.javafx.event.EventDispatchChainImpl.dispatchEvent(EventDispatchChainImpl.java:114)

     at com.sun.javafx.event.BasicEventDispatcher.dispatchEvent(BasicEventDispatcher.java:56)

     at com.sun.javafx.event.EventDispatchChainImpl.dispatchEvent(EventDispatchChainImpl.java:114)

     at com.sun.javafx.event.BasicEventDispatcher.dispatchEvent(BasicEventDispatcher.java:56)

     at com.sun.javafx.event.EventDispatchChainImpl.dispatchEvent(EventDispatchChainImpl.java:114)

     at com.sun.javafx.event.EventUtil.fireEventImpl(EventUtil.java:74)

     at com.sun.javafx.event.EventUtil.fireEvent(EventUtil.java:54)

     at javafx.event.Event.fireEvent(Event.java:198)

     at javafx.scene.Scene$MouseHandler.process(Scene.java:3856)

     at javafx.scene.Scene.processMouseEvent(Scene.java:1851)

     at javafx.scene.Scene$ScenePeerListener.mouseEvent(Scene.java:2584)

     at com.sun.javafx.tk.quantum.GlassViewEventHandler$MouseEventNotification.run(GlassViewEventHandler.java:409)

     at com.sun.javafx.tk.quantum.GlassViewEventHandler$MouseEventNotification.run(GlassViewEventHandler.java:299)

     at java.base/java.security.AccessController.doPrivileged(Native Method)

     at com.sun.javafx.tk.quantum.GlassViewEventHandler.lambda$handleMouseEvent$2(GlassViewEventHandler.java:447)

     at com.sun.javafx.tk.quantum.QuantumToolkit.runWithoutRenderLock(QuantumToolkit.java:412)

     at com.sun.javafx.tk.quantum.GlassViewEventHandler.handleMouseEvent(GlassViewEventHandler.java:446)

     at com.sun.glass.ui.View.handleMouseEvent(View.java:556)

     at com.sun.glass.ui.View.notifyMouse(View.java:942)

     at com.sun.glass.ui.win.WinApplication._runLoop(Native Method)

     at com.sun.glass.ui.win.WinApplication.lambda$runLoop$3(WinApplication.java:174)

     at java.base/java.lang.Thread.run(Thread.java:829)

 Caused by: java.lang.OutOfMemoryError

     at java.base/jdk.internal.reflect.GeneratedConstructorAccessor3.newInstance(Unknown Source)

     at java.base/jdk.internal.reflect.DelegatingConstructorAccessorImpl.newInstance(DelegatingConstructorAccessorImpl.java:45)

     at java.base/java.lang.reflect.Constructor.newInstance(Constructor.java:490)

     at java.base/java.util.concurrent.ForkJoinTask.getThrowableException(ForkJoinTask.java:603)

     ... 49 more

 Caused by: java.lang.OutOfMemoryError: Java heap space

 java.util.concurrent.ExecutionException: java.lang.OutOfMemoryError

     at java.base/java.util.concurrent.ForkJoinTask.get(ForkJoinTask.java:1006)

     at fr.univ_nantes.codex_processor.gui.DatasetExtendedDepthOfFieldProcessor.run(DatasetExtendedDepthOfFieldProcessor.java:69)

     at fr.univ_nantes.codex_processor.gui.MainController$1.handle(MainController.java:96)

     at fr.univ_nantes.codex_processor.gui.MainController$1.handle(MainController.java:64)

     at com.sun.javafx.event.CompositeEventHandler.dispatchBubblingEvent(CompositeEventHandler.java:86)

     at com.sun.javafx.event.EventHandlerManager.dispatchBubblingEvent(EventHandlerManager.java:234)

     at com.sun.javafx.event.EventHandlerManager.dispatchBubblingEvent(EventHandlerManager.java:191)

     at com.sun.javafx.event.CompositeEventDispatcher.dispatchBubblingEvent(CompositeEventDispatcher.java:59)

     at com.sun.javafx.event.BasicEventDispatcher.dispatchEvent(BasicEventDispatcher.java:58)

     at com.sun.javafx.event.EventDispatchChainImpl.dispatchEvent(EventDispatchChainImpl.java:114)

     at com.sun.javafx.event.BasicEventDispatcher.dispatchEvent(BasicEventDispatcher.java:56)

     at com.sun.javafx.event.EventDispatchChainImpl.dispatchEvent(EventDispatchChainImpl.java:114)

     at com.sun.javafx.event.BasicEventDispatcher.dispatchEvent(BasicEventDispatcher.java:56)

     at com.sun.javafx.event.EventDispatchChainImpl.dispatchEvent(EventDispatchChainImpl.java:114)

     at com.sun.javafx.event.EventUtil.fireEventImpl(EventUtil.java:74)

     at com.sun.javafx.event.EventUtil.fireEvent(EventUtil.java:49)

     at javafx.event.Event.fireEvent(Event.java:198)

     at javafx.scene.Node.fireEvent(Node.java:8889)

     at javafx.scene.control.Button.fire(Button.java:203)

     at com.sun.javafx.scene.control.behavior.ButtonBehavior.mouseReleased(ButtonBehavior.java:208)

     at com.sun.javafx.scene.control.inputmap.InputMap.handle(InputMap.java:274)

     at com.sun.javafx.event.CompositeEventHandler$NormalEventHandlerRecord.handleBubblingEvent(CompositeEventHandler.java:247)

     at com.sun.javafx.event.CompositeEventHandler.dispatchBubblingEvent(CompositeEventHandler.java:80)

     at com.sun.javafx.event.EventHandlerManager.dispatchBubblingEvent(EventHandlerManager.java:234)

     at com.sun.javafx.event.EventHandlerManager.dispatchBubblingEvent(EventHandlerManager.java:191)

     at com.sun.javafx.event.CompositeEventDispatcher.dispatchBubblingEvent(CompositeEventDispatcher.java:59)

     at com.sun.javafx.event.BasicEventDispatcher.dispatchEvent(BasicEventDispatcher.java:58)

     at com.sun.javafx.event.EventDispatchChainImpl.dispatchEvent(EventDispatchChainImpl.java:114)

     at com.sun.javafx.event.BasicEventDispatcher.dispatchEvent(BasicEventDispatcher.java:56)

     at com.sun.javafx.event.EventDispatchChainImpl.dispatchEvent(EventDispatchChainImpl.java:114)

     at com.sun.javafx.event.BasicEventDispatcher.dispatchEvent(BasicEventDispatcher.java:56)

     at com.sun.javafx.event.EventDispatchChainImpl.dispatchEvent(EventDispatchChainImpl.java:114)

     at com.sun.javafx.event.EventUtil.fireEventImpl(EventUtil.java:74)

     at com.sun.javafx.event.EventUtil.fireEvent(EventUtil.java:54)

     at javafx.event.Event.fireEvent(Event.java:198)

     at javafx.scene.Scene$MouseHandler.process(Scene.java:3856)

     at javafx.scene.Scene.processMouseEvent(Scene.java:1851)

     at javafx.scene.Scene$ScenePeerListener.mouseEvent(Scene.java:2584)

     at com.sun.javafx.tk.quantum.GlassViewEventHandler$MouseEventNotification.run(GlassViewEventHandler.java:409)

     at com.sun.javafx.tk.quantum.GlassViewEventHandler$MouseEventNotification.run(GlassViewEventHandler.java:299)

     at java.base/java.security.AccessController.doPrivileged(Native Method)

     at com.sun.javafx.tk.quantum.GlassViewEventHandler.lambda$handleMouseEvent$2(GlassViewEventHandler.java:447)

     at com.sun.javafx.tk.quantum.QuantumToolkit.runWithoutRenderLock(QuantumToolkit.java:412)

     at com.sun.javafx.tk.quantum.GlassViewEventHandler.handleMouseEvent(GlassViewEventHandler.java:446)

     at com.sun.glass.ui.View.handleMouseEvent(View.java:556)

     at com.sun.glass.ui.View.notifyMouse(View.java:942)

     at com.sun.glass.ui.win.WinApplication._runLoop(Native Method)

     at com.sun.glass.ui.win.WinApplication.lambda$runLoop$3(WinApplication.java:174)

     at java.base/java.lang.Thread.run(Thread.java:829)

 Caused by: java.lang.OutOfMemoryError

     at java.base/jdk.internal.reflect.GeneratedConstructorAccessor3.newInstance(Unknown Source)

     at java.base/jdk.internal.reflect.DelegatingConstructorAccessorImpl.newInstance(DelegatingConstructorAccessorImpl.java:45)

     at java.base/java.lang.reflect.Constructor.newInstance(Constructor.java:490)

     at java.base/java.util.concurrent.ForkJoinTask.getThrowableException(ForkJoinTask.java:603)

     ... 49 more

 Caused by: java.lang.OutOfMemoryError: Java heap space

 java.util.concurrent.ExecutionException: java.lang.OutOfMemoryError

     at java.base/java.util.concurrent.ForkJoinTask.get(ForkJoinTask.java:1006)

     at fr.univ_nantes.codex_processor.gui.DatasetExtendedDepthOfFieldProcessor.run(DatasetExtendedDepthOfFieldProcessor.java:69)

     at fr.univ_nantes.codex_processor.gui.MainController$1.handle(MainController.java:96)

     at fr.univ_nantes.codex_processor.gui.MainController$1.handle(MainController.java:64)

     at com.sun.javafx.event.CompositeEventHandler.dispatchBubblingEvent(CompositeEventHandler.java:86)

     at com.sun.javafx.event.EventHandlerManager.dispatchBubblingEvent(EventHandlerManager.java:234)

     at com.sun.javafx.event.EventHandlerManager.dispatchBubblingEvent(EventHandlerManager.java:191)

     at com.sun.javafx.event.CompositeEventDispatcher.dispatchBubblingEvent(CompositeEventDispatcher.java:59)

     at com.sun.javafx.event.BasicEventDispatcher.dispatchEvent(BasicEventDispatcher.java:58)

     at com.sun.javafx.event.EventDispatchChainImpl.dispatchEvent(EventDispatchChainImpl.java:114)

     at com.sun.javafx.event.BasicEventDispatcher.dispatchEvent(BasicEventDispatcher.java:56)

     at com.sun.javafx.event.EventDispatchChainImpl.dispatchEvent(EventDispatchChainImpl.java:114)

     at com.sun.javafx.event.BasicEventDispatcher.dispatchEvent(BasicEventDispatcher.java:56)

     at com.sun.javafx.event.EventDispatchChainImpl.dispatchEvent(EventDispatchChainImpl.java:114)

     at com.sun.javafx.event.EventUtil.fireEventImpl(EventUtil.java:74)

     at com.sun.javafx.event.EventUtil.fireEvent(EventUtil.java:49)

     at javafx.event.Event.fireEvent(Event.java:198)

     at javafx.scene.Node.fireEvent(Node.java:8889)

     at javafx.scene.control.Button.fire(Button.java:203)

     at com.sun.javafx.scene.control.behavior.ButtonBehavior.mouseReleased(ButtonBehavior.java:208)

     at com.sun.javafx.scene.control.inputmap.InputMap.handle(InputMap.java:274)

     at com.sun.javafx.event.CompositeEventHandler$NormalEventHandlerRecord.handleBubblingEvent(CompositeEventHandler.java:247)

     at com.sun.javafx.event.CompositeEventHandler.dispatchBubblingEvent(CompositeEventHandler.java:80)

     at com.sun.javafx.event.EventHandlerManager.dispatchBubblingEvent(EventHandlerManager.java:234)

     at com.sun.javafx.event.EventHandlerManager.dispatchBubblingEvent(EventHandlerManager.java:191)

     at com.sun.javafx.event.CompositeEventDispatcher.dispatchBubblingEvent(CompositeEventDispatcher.java:59)

     at com.sun.javafx.event.BasicEventDispatcher.dispatchEvent(BasicEventDispatcher.java:58)

     at com.sun.javafx.event.EventDispatchChainImpl.dispatchEvent(EventDispatchChainImpl.java:114)

     at com.sun.javafx.event.BasicEventDispatcher.dispatchEvent(BasicEventDispatcher.java:56)

     at com.sun.javafx.event.EventDispatchChainImpl.dispatchEvent(EventDispatchChainImpl.java:114)

     at com.sun.javafx.event.BasicEventDispatcher.dispatchEvent(BasicEventDispatcher.java:56)

     at com.sun.javafx.event.EventDispatchChainImpl.dispatchEvent(EventDispatchChainImpl.java:114)

     at com.sun.javafx.event.EventUtil.fireEventImpl(EventUtil.java:74)

     at com.sun.javafx.event.EventUtil.fireEvent(EventUtil.java:54)

     at javafx.event.Event.fireEvent(Event.java:198)

     at javafx.scene.Scene$MouseHandler.process(Scene.java:3856)

     at javafx.scene.Scene.processMouseEvent(Scene.java:1851)

     at javafx.scene.Scene$ScenePeerListener.mouseEvent(Scene.java:2584)

     at com.sun.javafx.tk.quantum.GlassViewEventHandler$MouseEventNotification.run(GlassViewEventHandler.java:409)

     at com.sun.javafx.tk.quantum.GlassViewEventHandler$MouseEventNotification.run(GlassViewEventHandler.java:299)

     at java.base/java.security.AccessController.doPrivileged(Native Method)

     at com.sun.javafx.tk.quantum.GlassViewEventHandler.lambda$handleMouseEvent$2(GlassViewEventHandler.java:447)

     at com.sun.javafx.tk.quantum.QuantumToolkit.runWithoutRenderLock(QuantumToolkit.java:412)

     at com.sun.javafx.tk.quantum.GlassViewEventHandler.handleMouseEvent(GlassViewEventHandler.java:446)

     at com.sun.glass.ui.View.handleMouseEvent(View.java:556)

     at com.sun.glass.ui.View.notifyMouse(View.java:942)

     at com.sun.glass.ui.win.WinApplication._runLoop(Native Method)

     at com.sun.glass.ui.win.WinApplication.lambda$runLoop$3(WinApplication.java:174)

     at java.base/java.lang.Thread.run(Thread.java:829)

 Caused by: java.lang.OutOfMemoryError

     at java.base/jdk.internal.reflect.GeneratedConstructorAccessor3.newInstance(Unknown Source)

     at java.base/jdk.internal.reflect.DelegatingConstructorAccessorImpl.newInstance(DelegatingConstructorAccessorImpl.java:45)

     at java.base/java.lang.reflect.Constructor.newInstance(Constructor.java:490)

     at java.base/java.util.concurrent.ForkJoinTask.getThrowableException(ForkJoinTask.java:603)

     ... 49 more

 Caused by: java.lang.OutOfMemoryError: Java heap space

 java.util.concurrent.ExecutionException: java.lang.OutOfMemoryError

     at java.base/java.util.concurrent.ForkJoinTask.get(ForkJoinTask.java:1006)

     at fr.univ_nantes.codex_processor.gui.DatasetExtendedDepthOfFieldProcessor.run(DatasetExtendedDepthOfFieldProcessor.java:69)

     at fr.univ_nantes.codex_processor.gui.MainController$1.handle(MainController.java:96)

     at fr.univ_nantes.codex_processor.gui.MainController$1.handle(MainController.java:64)

     at com.sun.javafx.event.CompositeEventHandler.dispatchBubblingEvent(CompositeEventHandler.java:86)

     at com.sun.javafx.event.EventHandlerManager.dispatchBubblingEvent(EventHandlerManager.java:234)

     at com.sun.javafx.event.EventHandlerManager.dispatchBubblingEvent(EventHandlerManager.java:191)

     at com.sun.javafx.event.CompositeEventDispatcher.dispatchBubblingEvent(CompositeEventDispatcher.java:59)

     at com.sun.javafx.event.BasicEventDispatcher.dispatchEvent(BasicEventDispatcher.java:58)

     at com.sun.javafx.event.EventDispatchChainImpl.dispatchEvent(EventDispatchChainImpl.java:114)

     at com.sun.javafx.event.BasicEventDispatcher.dispatchEvent(BasicEventDispatcher.java:56)

     at com.sun.javafx.event.EventDispatchChainImpl.dispatchEvent(EventDispatchChainImpl.java:114)

     at com.sun.javafx.event.BasicEventDispatcher.dispatchEvent(BasicEventDispatcher.java:56)

     at com.sun.javafx.event.EventDispatchChainImpl.dispatchEvent(EventDispatchChainImpl.java:114)

     at com.sun.javafx.event.EventUtil.fireEventImpl(EventUtil.java:74)

     at com.sun.javafx.event.EventUtil.fireEvent(EventUtil.java:49)

     at javafx.event.Event.fireEvent(Event.java:198)

     at javafx.scene.Node.fireEvent(Node.java:8889)

     at javafx.scene.control.Button.fire(Button.java:203)

     at com.sun.javafx.scene.control.behavior.ButtonBehavior.mouseReleased(ButtonBehavior.java:208)

     at com.sun.javafx.scene.control.inputmap.InputMap.handle(InputMap.java:274)

     at com.sun.javafx.event.CompositeEventHandler$NormalEventHandlerRecord.handleBubblingEvent(CompositeEventHandler.java:247)

     at com.sun.javafx.event.CompositeEventHandler.dispatchBubblingEvent(CompositeEventHandler.java:80)

     at com.sun.javafx.event.EventHandlerManager.dispatchBubblingEvent(EventHandlerManager.java:234)

     at com.sun.javafx.event.EventHandlerManager.dispatchBubblingEvent(EventHandlerManager.java:191)

     at com.sun.javafx.event.CompositeEventDispatcher.dispatchBubblingEvent(CompositeEventDispatcher.java:59)

     at com.sun.javafx.event.BasicEventDispatcher.dispatchEvent(BasicEventDispatcher.java:58)

     at com.sun.javafx.event.EventDispatchChainImpl.dispatchEvent(EventDispatchChainImpl.java:114)

     at com.sun.javafx.event.BasicEventDispatcher.dispatchEvent(BasicEventDispatcher.java:56)

     at com.sun.javafx.event.EventDispatchChainImpl.dispatchEvent(EventDispatchChainImpl.java:114)

     at com.sun.javafx.event.BasicEventDispatcher.dispatchEvent(BasicEventDispatcher.java:56)

     at com.sun.javafx.event.EventDispatchChainImpl.dispatchEvent(EventDispatchChainImpl.java:114)

     at com.sun.javafx.event.EventUtil.fireEventImpl(EventUtil.java:74)

     at com.sun.javafx.event.EventUtil.fireEvent(EventUtil.java:54)

     at javafx.event.Event.fireEvent(Event.java:198)

     at javafx.scene.Scene$MouseHandler.process(Scene.java:3856)

     at javafx.scene.Scene.processMouseEvent(Scene.java:1851)

     at javafx.scene.Scene$ScenePeerListener.mouseEvent(Scene.java:2584)

     at com.sun.javafx.tk.quantum.GlassViewEventHandler$MouseEventNotification.run(GlassViewEventHandler.java:409)

     at com.sun.javafx.tk.quantum.GlassViewEventHandler$MouseEventNotification.run(GlassViewEventHandler.java:299)

     at java.base/java.security.AccessController.doPrivileged(Native Method)

     at com.sun.javafx.tk.quantum.GlassViewEventHandler.lambda$handleMouseEvent$2(GlassViewEventHandler.java:447)

     at com.sun.javafx.tk.quantum.QuantumToolkit.runWithoutRenderLock(QuantumToolkit.java:412)

     at com.sun.javafx.tk.quantum.GlassViewEventHandler.handleMouseEvent(GlassViewEventHandler.java:446)

     at com.sun.glass.ui.View.handleMouseEvent(View.java:556)

     at com.sun.glass.ui.View.notifyMouse(View.java:942)

     at com.sun.glass.ui.win.WinApplication._runLoop(Native Method)

     at com.sun.glass.ui.win.WinApplication.lambda$runLoop$3(WinApplication.java:174)

     at java.base/java.lang.Thread.run(Thread.java:829)

 Caused by: java.lang.OutOfMemoryError

     at java.base/jdk.internal.reflect.GeneratedConstructorAccessor3.newInstance(Unknown Source)

     at java.base/jdk.internal.reflect.DelegatingConstructorAccessorImpl.newInstance(DelegatingConstructorAccessorImpl.java:45)

     at java.base/java.lang.reflect.Constructor.newInstance(Constructor.java:490)

     at java.base/java.util.concurrent.ForkJoinTask.getThrowableException(ForkJoinTask.java:603)

     ... 49 more

 Caused by: java.lang.OutOfMemoryError: Java heap space

 java.util.concurrent.ExecutionException: java.lang.OutOfMemoryError

     at java.base/java.util.concurrent.ForkJoinTask.get(ForkJoinTask.java:1006)

     at fr.univ_nantes.codex_processor.gui.DatasetExtendedDepthOfFieldProcessor.run(DatasetExtendedDepthOfFieldProcessor.java:69)

     at fr.univ_nantes.codex_processor.gui.MainController$1.handle(MainController.java:96)

     at fr.univ_nantes.codex_processor.gui.MainController$1.handle(MainController.java:64)

     at com.sun.javafx.event.CompositeEventHandler.dispatchBubblingEvent(CompositeEventHandler.java:86)

     at com.sun.javafx.event.EventHandlerManager.dispatchBubblingEvent(EventHandlerManager.java:234)

     at com.sun.javafx.event.EventHandlerManager.dispatchBubblingEvent(EventHandlerManager.java:191)

     at com.sun.javafx.event.CompositeEventDispatcher.dispatchBubblingEvent(CompositeEventDispatcher.java:59)

     at com.sun.javafx.event.BasicEventDispatcher.dispatchEvent(BasicEventDispatcher.java:58)

     at com.sun.javafx.event.EventDispatchChainImpl.dispatchEvent(EventDispatchChainImpl.java:114)

     at com.sun.javafx.event.BasicEventDispatcher.dispatchEvent(BasicEventDispatcher.java:56)

     at com.sun.javafx.event.EventDispatchChainImpl.dispatchEvent(EventDispatchChainImpl.java:114)

     at com.sun.javafx.event.BasicEventDispatcher.dispatchEvent(BasicEventDispatcher.java:56)

     at com.sun.javafx.event.EventDispatchChainImpl.dispatchEvent(EventDispatchChainImpl.java:114)

     at com.sun.javafx.event.EventUtil.fireEventImpl(EventUtil.java:74)

     at com.sun.javafx.event.EventUtil.fireEvent(EventUtil.java:49)

     at javafx.event.Event.fireEvent(Event.java:198)

     at javafx.scene.Node.fireEvent(Node.java:8889)

     at javafx.scene.control.Button.fire(Button.java:203)

     at com.sun.javafx.scene.control.behavior.ButtonBehavior.mouseReleased(ButtonBehavior.java:208)

     at com.sun.javafx.scene.control.inputmap.InputMap.handle(InputMap.java:274)

     at com.sun.javafx.event.CompositeEventHandler$NormalEventHandlerRecord.handleBubblingEvent(CompositeEventHandler.java:247)

     at com.sun.javafx.event.CompositeEventHandler.dispatchBubblingEvent(CompositeEventHandler.java:80)

     at com.sun.javafx.event.EventHandlerManager.dispatchBubblingEvent(EventHandlerManager.java:234)

     at com.sun.javafx.event.EventHandlerManager.dispatchBubblingEvent(EventHandlerManager.java:191)

     at com.sun.javafx.event.CompositeEventDispatcher.dispatchBubblingEvent(CompositeEventDispatcher.java:59)

     at com.sun.javafx.event.BasicEventDispatcher.dispatchEvent(BasicEventDispatcher.java:58)

     at com.sun.javafx.event.EventDispatchChainImpl.dispatchEvent(EventDispatchChainImpl.java:114)

     at com.sun.javafx.event.BasicEventDispatcher.dispatchEvent(BasicEventDispatcher.java:56)

     at com.sun.javafx.event.EventDispatchChainImpl.dispatchEvent(EventDispatchChainImpl.java:114)

     at com.sun.javafx.event.BasicEventDispatcher.dispatchEvent(BasicEventDispatcher.java:56)

     at com.sun.javafx.event.EventDispatchChainImpl.dispatchEvent(EventDispatchChainImpl.java:114)

     at com.sun.javafx.event.EventUtil.fireEventImpl(EventUtil.java:74)

     at com.sun.javafx.event.EventUtil.fireEvent(EventUtil.java:54)

     at javafx.event.Event.fireEvent(Event.java:198)

     at javafx.scene.Scene$MouseHandler.process(Scene.java:3856)

     at javafx.scene.Scene.processMouseEvent(Scene.java:1851)

     at javafx.scene.Scene$ScenePeerListener.mouseEvent(Scene.java:2584)

     at com.sun.javafx.tk.quantum.GlassViewEventHandler$MouseEventNotification.run(GlassViewEventHandler.java:409)

     at com.sun.javafx.tk.quantum.GlassViewEventHandler$MouseEventNotification.run(GlassViewEventHandler.java:299)

     at java.base/java.security.AccessController.doPrivileged(Native Method)

     at com.sun.javafx.tk.quantum.GlassViewEventHandler.lambda$handleMouseEvent$2(GlassViewEventHandler.java:447)

     at com.sun.javafx.tk.quantum.QuantumToolkit.runWithoutRenderLock(QuantumToolkit.java:412)

     at com.sun.javafx.tk.quantum.GlassViewEventHandler.handleMouseEvent(GlassViewEventHandler.java:446)

     at com.sun.glass.ui.View.handleMouseEvent(View.java:556)

     at com.sun.glass.ui.View.notifyMouse(View.java:942)

     at com.sun.glass.ui.win.WinApplication._runLoop(Native Method)

     at com.sun.glass.ui.win.WinApplication.lambda$runLoop$3(WinApplication.java:174)

     at java.base/java.lang.Thread.run(Thread.java:829)

 Caused by: java.lang.OutOfMemoryError

     at java.base/jdk.internal.reflect.GeneratedConstructorAccessor3.newInstance(Unknown Source)

     at java.base/jdk.internal.reflect.DelegatingConstructorAccessorImpl.newInstance(DelegatingConstructorAccessorImpl.java:45)

     at java.base/java.lang.reflect.Constructor.newInstance(Constructor.java:490)

     at java.base/java.util.concurrent.ForkJoinTask.getThrowableException(ForkJoinTask.java:603)

     ... 49 more

 Caused by: java.lang.OutOfMemoryError: Java heap space

 java.util.concurrent.ExecutionException: java.lang.OutOfMemoryError

     at java.base/java.util.concurrent.ForkJoinTask.get(ForkJoinTask.java:1006)

     at fr.univ_nantes.codex_processor.gui.DatasetExtendedDepthOfFieldProcessor.run(DatasetExtendedDepthOfFieldProcessor.java:69)

     at fr.univ_nantes.codex_processor.gui.MainController$1.handle(MainController.java:96)

     at fr.univ_nantes.codex_processor.gui.MainController$1.handle(MainController.java:64)

     at com.sun.javafx.event.CompositeEventHandler.dispatchBubblingEvent(CompositeEventHandler.java:86)

     at com.sun.javafx.event.EventHandlerManager.dispatchBubblingEvent(EventHandlerManager.java:234)

     at com.sun.javafx.event.EventHandlerManager.dispatchBubblingEvent(EventHandlerManager.java:191)

     at com.sun.javafx.event.CompositeEventDispatcher.dispatchBubblingEvent(CompositeEventDispatcher.java:59)

     at com.sun.javafx.event.BasicEventDispatcher.dispatchEvent(BasicEventDispatcher.java:58)

     at com.sun.javafx.event.EventDispatchChainImpl.dispatchEvent(EventDispatchChainImpl.java:114)

     at com.sun.javafx.event.BasicEventDispatcher.dispatchEvent(BasicEventDispatcher.java:56)

     at com.sun.javafx.event.EventDispatchChainImpl.dispatchEvent(EventDispatchChainImpl.java:114)

     at com.sun.javafx.event.BasicEventDispatcher.dispatchEvent(BasicEventDispatcher.java:56)

     at com.sun.javafx.event.EventDispatchChainImpl.dispatchEvent(EventDispatchChainImpl.java:114)

     at com.sun.javafx.event.EventUtil.fireEventImpl(EventUtil.java:74)

     at com.sun.javafx.event.EventUtil.fireEvent(EventUtil.java:49)

     at javafx.event.Event.fireEvent(Event.java:198)

     at javafx.scene.Node.fireEvent(Node.java:8889)

     at javafx.scene.control.Button.fire(Button.java:203)

     at com.sun.javafx.scene.control.behavior.ButtonBehavior.mouseReleased(ButtonBehavior.java:208)

     at com.sun.javafx.scene.control.inputmap.InputMap.handle(InputMap.java:274)

     at com.sun.javafx.event.CompositeEventHandler$NormalEventHandlerRecord.handleBubblingEvent(CompositeEventHandler.java:247)

     at com.sun.javafx.event.CompositeEventHandler.dispatchBubblingEvent(CompositeEventHandler.java:80)

     at com.sun.javafx.event.EventHandlerManager.dispatchBubblingEvent(EventHandlerManager.java:234)

     at com.sun.javafx.event.EventHandlerManager.dispatchBubblingEvent(EventHandlerManager.java:191)

     at com.sun.javafx.event.CompositeEventDispatcher.dispatchBubblingEvent(CompositeEventDispatcher.java:59)

     at com.sun.javafx.event.BasicEventDispatcher.dispatchEvent(BasicEventDispatcher.java:58)

     at com.sun.javafx.event.EventDispatchChainImpl.dispatchEvent(EventDispatchChainImpl.java:114)

     at com.sun.javafx.event.BasicEventDispatcher.dispatchEvent(BasicEventDispatcher.java:56)

     at com.sun.javafx.event.EventDispatchChainImpl.dispatchEvent(EventDispatchChainImpl.java:114)

     at com.sun.javafx.event.BasicEventDispatcher.dispatchEvent(BasicEventDispatcher.java:56)

     at com.sun.javafx.event.EventDispatchChainImpl.dispatchEvent(EventDispatchChainImpl.java:114)

     at com.sun.javafx.event.EventUtil.fireEventImpl(EventUtil.java:74)

     at com.sun.javafx.event.EventUtil.fireEvent(EventUtil.java:54)

     at javafx.event.Event.fireEvent(Event.java:198)

     at javafx.scene.Scene$MouseHandler.process(Scene.java:3856)

     at javafx.scene.Scene.processMouseEvent(Scene.java:1851)

     at javafx.scene.Scene$ScenePeerListener.mouseEvent(Scene.java:2584)

     at com.sun.javafx.tk.quantum.GlassViewEventHandler$MouseEventNotification.run(GlassViewEventHandler.java:409)

     at com.sun.javafx.tk.quantum.GlassViewEventHandler$MouseEventNotification.run(GlassViewEventHandler.java:299)

     at java.base/java.security.AccessController.doPrivileged(Native Method)

     at com.sun.javafx.tk.quantum.GlassViewEventHandler.lambda$handleMouseEvent$2(GlassViewEventHandler.java:447)

     at com.sun.javafx.tk.quantum.QuantumToolkit.runWithoutRenderLock(QuantumToolkit.java:412)

     at com.sun.javafx.tk.quantum.GlassViewEventHandler.handleMouseEvent(GlassViewEventHandler.java:446)

     at com.sun.glass.ui.View.handleMouseEvent(View.java:556)

     at com.sun.glass.ui.View.notifyMouse(View.java:942)

     at com.sun.glass.ui.win.WinApplication._runLoop(Native Method)

     at com.sun.glass.ui.win.WinApplication.lambda$runLoop$3(WinApplication.java:174)

     at java.base/java.lang.Thread.run(Thread.java:829)

 Caused by: java.lang.OutOfMemoryError

     at java.base/jdk.internal.reflect.GeneratedConstructorAccessor3.newInstance(Unknown Source)

     at java.base/jdk.internal.reflect.DelegatingConstructorAccessorImpl.newInstance(DelegatingConstructorAccessorImpl.java:45)

     at java.base/java.lang.reflect.Constructor.newInstance(Constructor.java:490)

     at java.base/java.util.concurrent.ForkJoinTask.getThrowableException(ForkJoinTask.java:603)

     ... 49 more

 Caused by: java.lang.OutOfMemoryError: Java heap space

 java.util.concurrent.ExecutionException: java.lang.OutOfMemoryError

     at java.base/java.util.concurrent.ForkJoinTask.get(ForkJoinTask.java:1006)

     at fr.univ_nantes.codex_processor.gui.DatasetExtendedDepthOfFieldProcessor.run(DatasetExtendedDepthOfFieldProcessor.java:69)

     at fr.univ_nantes.codex_processor.gui.MainController$1.handle(MainController.java:96)

     at fr.univ_nantes.codex_processor.gui.MainController$1.handle(MainController.java:64)

     at com.sun.javafx.event.CompositeEventHandler.dispatchBubblingEvent(CompositeEventHandler.java:86)

     at com.sun.javafx.event.EventHandlerManager.dispatchBubblingEvent(EventHandlerManager.java:234)

     at com.sun.javafx.event.EventHandlerManager.dispatchBubblingEvent(EventHandlerManager.java:191)

     at com.sun.javafx.event.CompositeEventDispatcher.dispatchBubblingEvent(CompositeEventDispatcher.java:59)

     at com.sun.javafx.event.BasicEventDispatcher.dispatchEvent(BasicEventDispatcher.java:58)

     at com.sun.javafx.event.EventDispatchChainImpl.dispatchEvent(EventDispatchChainImpl.java:114)

     at com.sun.javafx.event.BasicEventDispatcher.dispatchEvent(BasicEventDispatcher.java:56)

     at com.sun.javafx.event.EventDispatchChainImpl.dispatchEvent(EventDispatchChainImpl.java:114)

     at com.sun.javafx.event.BasicEventDispatcher.dispatchEvent(BasicEventDispatcher.java:56)

     at com.sun.javafx.event.EventDispatchChainImpl.dispatchEvent(EventDispatchChainImpl.java:114)

     at com.sun.javafx.event.EventUtil.fireEventImpl(EventUtil.java:74)

     at com.sun.javafx.event.EventUtil.fireEvent(EventUtil.java:49)

     at javafx.event.Event.fireEvent(Event.java:198)

     at javafx.scene.Node.fireEvent(Node.java:8889)

     at javafx.scene.control.Button.fire(Button.java:203)

     at com.sun.javafx.scene.control.behavior.ButtonBehavior.mouseReleased(ButtonBehavior.java:208)

     at com.sun.javafx.scene.control.inputmap.InputMap.handle(InputMap.java:274)

     at com.sun.javafx.event.CompositeEventHandler$NormalEventHandlerRecord.handleBubblingEvent(CompositeEventHandler.java:247)

     at com.sun.javafx.event.CompositeEventHandler.dispatchBubblingEvent(CompositeEventHandler.java:80)

     at com.sun.javafx.event.EventHandlerManager.dispatchBubblingEvent(EventHandlerManager.java:234)

     at com.sun.javafx.event.EventHandlerManager.dispatchBubblingEvent(EventHandlerManager.java:191)

     at com.sun.javafx.event.CompositeEventDispatcher.dispatchBubblingEvent(CompositeEventDispatcher.java:59)

     at com.sun.javafx.event.BasicEventDispatcher.dispatchEvent(BasicEventDispatcher.java:58)

     at com.sun.javafx.event.EventDispatchChainImpl.dispatchEvent(EventDispatchChainImpl.java:114)

     at com.sun.javafx.event.BasicEventDispatcher.dispatchEvent(BasicEventDispatcher.java:56)

     at com.sun.javafx.event.EventDispatchChainImpl.dispatchEvent(EventDispatchChainImpl.java:114)

     at com.sun.javafx.event.BasicEventDispatcher.dispatchEvent(BasicEventDispatcher.java:56)

     at com.sun.javafx.event.EventDispatchChainImpl.dispatchEvent(EventDispatchChainImpl.java:114)

     at com.sun.javafx.event.EventUtil.fireEventImpl(EventUtil.java:74)

     at com.sun.javafx.event.EventUtil.fireEvent(EventUtil.java:54)

     at javafx.event.Event.fireEvent(Event.java:198)

     at javafx.scene.Scene$MouseHandler.process(Scene.java:3856)

     at javafx.scene.Scene.processMouseEvent(Scene.java:1851)

     at javafx.scene.Scene$ScenePeerListener.mouseEvent(Scene.java:2584)

     at com.sun.javafx.tk.quantum.GlassViewEventHandler$MouseEventNotification.run(GlassViewEventHandler.java:409)

     at com.sun.javafx.tk.quantum.GlassViewEventHandler$MouseEventNotification.run(GlassViewEventHandler.java:299)

     at java.base/java.security.AccessController.doPrivileged(Native Method)

     at com.sun.javafx.tk.quantum.GlassViewEventHandler.lambda$handleMouseEvent$2(GlassViewEventHandler.java:447)

     at com.sun.javafx.tk.quantum.QuantumToolkit.runWithoutRenderLock(QuantumToolkit.java:412)

     at com.sun.javafx.tk.quantum.GlassViewEventHandler.handleMouseEvent(GlassViewEventHandler.java:446)

     at com.sun.glass.ui.View.handleMouseEvent(View.java:556)

     at com.sun.glass.ui.View.notifyMouse(View.java:942)

     at com.sun.glass.ui.win.WinApplication._runLoop(Native Method)

     at com.sun.glass.ui.win.WinApplication.lambda$runLoop$3(WinApplication.java:174)

     at java.base/java.lang.Thread.run(Thread.java:829)

 Caused by: java.lang.OutOfMemoryError

     at java.base/jdk.internal.reflect.GeneratedConstructorAccessor3.newInstance(Unknown Source)

     at java.base/jdk.internal.reflect.DelegatingConstructorAccessorImpl.newInstance(DelegatingConstructorAccessorImpl.java:45)

     at java.base/java.lang.reflect.Constructor.newInstance(Constructor.java:490)

     at java.base/java.util.concurrent.ForkJoinTask.getThrowableException(ForkJoinTask.java:603)

     ... 49 more

 Caused by: java.lang.OutOfMemoryError: Java heap space

 java.util.concurrent.ExecutionException: java.lang.OutOfMemoryError

     at java.base/java.util.concurrent.ForkJoinTask.get(ForkJoinTask.java:1006)

     at fr.univ_nantes.codex_processor.gui.DatasetExtendedDepthOfFieldProcessor.run(DatasetExtendedDepthOfFieldProcessor.java:69)

     at fr.univ_nantes.codex_processor.gui.MainController$1.handle(MainController.java:96)

     at fr.univ_nantes.codex_processor.gui.MainController$1.handle(MainController.java:64)

     at com.sun.javafx.event.CompositeEventHandler.dispatchBubblingEvent(CompositeEventHandler.java:86)

     at com.sun.javafx.event.EventHandlerManager.dispatchBubblingEvent(EventHandlerManager.java:234)

     at com.sun.javafx.event.EventHandlerManager.dispatchBubblingEvent(EventHandlerManager.java:191)

     at com.sun.javafx.event.CompositeEventDispatcher.dispatchBubblingEvent(CompositeEventDispatcher.java:59)

     at com.sun.javafx.event.BasicEventDispatcher.dispatchEvent(BasicEventDispatcher.java:58)

     at com.sun.javafx.event.EventDispatchChainImpl.dispatchEvent(EventDispatchChainImpl.java:114)

     at com.sun.javafx.event.BasicEventDispatcher.dispatchEvent(BasicEventDispatcher.java:56)

     at com.sun.javafx.event.EventDispatchChainImpl.dispatchEvent(EventDispatchChainImpl.java:114)

     at com.sun.javafx.event.BasicEventDispatcher.dispatchEvent(BasicEventDispatcher.java:56)

     at com.sun.javafx.event.EventDispatchChainImpl.dispatchEvent(EventDispatchChainImpl.java:114)

     at com.sun.javafx.event.EventUtil.fireEventImpl(EventUtil.java:74)

     at com.sun.javafx.event.EventUtil.fireEvent(EventUtil.java:49)

     at javafx.event.Event.fireEvent(Event.java:198)

     at javafx.scene.Node.fireEvent(Node.java:8889)

     at javafx.scene.control.Button.fire(Button.java:203)

     at com.sun.javafx.scene.control.behavior.ButtonBehavior.mouseReleased(ButtonBehavior.java:208)

     at com.sun.javafx.scene.control.inputmap.InputMap.handle(InputMap.java:274)

     at com.sun.javafx.event.CompositeEventHandler$NormalEventHandlerRecord.handleBubblingEvent(CompositeEventHandler.java:247)

     at com.sun.javafx.event.CompositeEventHandler.dispatchBubblingEvent(CompositeEventHandler.java:80)

     at com.sun.javafx.event.EventHandlerManager.dispatchBubblingEvent(EventHandlerManager.java:234)

     at com.sun.javafx.event.EventHandlerManager.dispatchBubblingEvent(EventHandlerManager.java:191)

     at com.sun.javafx.event.CompositeEventDispatcher.dispatchBubblingEvent(CompositeEventDispatcher.java:59)

     at com.sun.javafx.event.BasicEventDispatcher.dispatchEvent(BasicEventDispatcher.java:58)

     at com.sun.javafx.event.EventDispatchChainImpl.dispatchEvent(EventDispatchChainImpl.java:114)

     at com.sun.javafx.event.BasicEventDispatcher.dispatchEvent(BasicEventDispatcher.java:56)

     at com.sun.javafx.event.EventDispatchChainImpl.dispatchEvent(EventDispatchChainImpl.java:114)

     at com.sun.javafx.event.BasicEventDispatcher.dispatchEvent(BasicEventDispatcher.java:56)

     at com.sun.javafx.event.EventDispatchChainImpl.dispatchEvent(EventDispatchChainImpl.java:114)

     at com.sun.javafx.event.EventUtil.fireEventImpl(EventUtil.java:74)

     at com.sun.javafx.event.EventUtil.fireEvent(EventUtil.java:54)

     at javafx.event.Event.fireEvent(Event.java:198)

     at javafx.scene.Scene$MouseHandler.process(Scene.java:3856)

     at javafx.scene.Scene.processMouseEvent(Scene.java:1851)

     at javafx.scene.Scene$ScenePeerListener.mouseEvent(Scene.java:2584)

     at com.sun.javafx.tk.quantum.GlassViewEventHandler$MouseEventNotification.run(GlassViewEventHandler.java:409)

     at com.sun.javafx.tk.quantum.GlassViewEventHandler$MouseEventNotification.run(GlassViewEventHandler.java:299)

     at java.base/java.security.AccessController.doPrivileged(Native Method)

     at com.sun.javafx.tk.quantum.GlassViewEventHandler.lambda$handleMouseEvent$2(GlassViewEventHandler.java:447)

     at com.sun.javafx.tk.quantum.QuantumToolkit.runWithoutRenderLock(QuantumToolkit.java:412)

     at com.sun.javafx.tk.quantum.GlassViewEventHandler.handleMouseEvent(GlassViewEventHandler.java:446)

     at com.sun.glass.ui.View.handleMouseEvent(View.java:556)

     at com.sun.glass.ui.View.notifyMouse(View.java:942)

     at com.sun.glass.ui.win.WinApplication._runLoop(Native Method)

     at com.sun.glass.ui.win.WinApplication.lambda$runLoop$3(WinApplication.java:174)

     at java.base/java.lang.Thread.run(Thread.java:829)

 Caused by: java.lang.OutOfMemoryError

     at java.base/jdk.internal.reflect.GeneratedConstructorAccessor3.newInstance(Unknown Source)

     at java.base/jdk.internal.reflect.DelegatingConstructorAccessorImpl.newInstance(DelegatingConstructorAccessorImpl.java:45)

     at java.base/java.lang.reflect.Constructor.newInstance(Constructor.java:490)

     at java.base/java.util.concurrent.ForkJoinTask.getThrowableException(ForkJoinTask.java:603)

     ... 49 more

 Caused by: java.lang.OutOfMemoryError: Java heap space

 java.util.concurrent.ExecutionException: java.lang.OutOfMemoryError

     at java.base/java.util.concurrent.ForkJoinTask.get(ForkJoinTask.java:1006)

     at fr.univ_nantes.codex_processor.gui.DatasetExtendedDepthOfFieldProcessor.run(DatasetExtendedDepthOfFieldProcessor.java:69)

     at fr.univ_nantes.codex_processor.gui.MainController$1.handle(MainController.java:96)

     at fr.univ_nantes.codex_processor.gui.MainController$1.handle(MainController.java:64)

     at com.sun.javafx.event.CompositeEventHandler.dispatchBubblingEvent(CompositeEventHandler.java:86)

     at com.sun.javafx.event.EventHandlerManager.dispatchBubblingEvent(EventHandlerManager.java:234)

     at com.sun.javafx.event.EventHandlerManager.dispatchBubblingEvent(EventHandlerManager.java:191)

     at com.sun.javafx.event.CompositeEventDispatcher.dispatchBubblingEvent(CompositeEventDispatcher.java:59)

     at com.sun.javafx.event.BasicEventDispatcher.dispatchEvent(BasicEventDispatcher.java:58)

     at com.sun.javafx.event.EventDispatchChainImpl.dispatchEvent(EventDispatchChainImpl.java:114)

     at com.sun.javafx.event.BasicEventDispatcher.dispatchEvent(BasicEventDispatcher.java:56)

     at com.sun.javafx.event.EventDispatchChainImpl.dispatchEvent(EventDispatchChainImpl.java:114)

     at com.sun.javafx.event.BasicEventDispatcher.dispatchEvent(BasicEventDispatcher.java:56)

     at com.sun.javafx.event.EventDispatchChainImpl.dispatchEvent(EventDispatchChainImpl.java:114)

     at com.sun.javafx.event.EventUtil.fireEventImpl(EventUtil.java:74)

     at com.sun.javafx.event.EventUtil.fireEvent(EventUtil.java:49)

     at javafx.event.Event.fireEvent(Event.java:198)

     at javafx.scene.Node.fireEvent(Node.java:8889)

     at javafx.scene.control.Button.fire(Button.java:203)

     at com.sun.javafx.scene.control.behavior.ButtonBehavior.mouseReleased(ButtonBehavior.java:208)

     at com.sun.javafx.scene.control.inputmap.InputMap.handle(InputMap.java:274)

     at com.sun.javafx.event.CompositeEventHandler$NormalEventHandlerRecord.handleBubblingEvent(CompositeEventHandler.java:247)

     at com.sun.javafx.event.CompositeEventHandler.dispatchBubblingEvent(CompositeEventHandler.java:80)

     at com.sun.javafx.event.EventHandlerManager.dispatchBubblingEvent(EventHandlerManager.java:234)

     at com.sun.javafx.event.EventHandlerManager.dispatchBubblingEvent(EventHandlerManager.java:191)

     at com.sun.javafx.event.CompositeEventDispatcher.dispatchBubblingEvent(CompositeEventDispatcher.java:59)

     at com.sun.javafx.event.BasicEventDispatcher.dispatchEvent(BasicEventDispatcher.java:58)

     at com.sun.javafx.event.EventDispatchChainImpl.dispatchEvent(EventDispatchChainImpl.java:114)

     at com.sun.javafx.event.BasicEventDispatcher.dispatchEvent(BasicEventDispatcher.java:56)

     at com.sun.javafx.event.EventDispatchChainImpl.dispatchEvent(EventDispatchChainImpl.java:114)

     at com.sun.javafx.event.BasicEventDispatcher.dispatchEvent(BasicEventDispatcher.java:56)

     at com.sun.javafx.event.EventDispatchChainImpl.dispatchEvent(EventDispatchChainImpl.java:114)

     at com.sun.javafx.event.EventUtil.fireEventImpl(EventUtil.java:74)

     at com.sun.javafx.event.EventUtil.fireEvent(EventUtil.java:54)

     at javafx.event.Event.fireEvent(Event.java:198)

     at javafx.scene.Scene$MouseHandler.process(Scene.java:3856)

     at javafx.scene.Scene.processMouseEvent(Scene.java:1851)

     at javafx.scene.Scene$ScenePeerListener.mouseEvent(Scene.java:2584)

     at com.sun.javafx.tk.quantum.GlassViewEventHandler$MouseEventNotification.run(GlassViewEventHandler.java:409)

     at com.sun.javafx.tk.quantum.GlassViewEventHandler$MouseEventNotification.run(GlassViewEventHandler.java:299)

     at java.base/java.security.AccessController.doPrivileged(Native Method)

     at com.sun.javafx.tk.quantum.GlassViewEventHandler.lambda$handleMouseEvent$2(GlassViewEventHandler.java:447)

     at com.sun.javafx.tk.quantum.QuantumToolkit.runWithoutRenderLock(QuantumToolkit.java:412)

     at com.sun.javafx.tk.quantum.GlassViewEventHandler.handleMouseEvent(GlassViewEventHandler.java:446)

     at com.sun.glass.ui.View.handleMouseEvent(View.java:556)

     at com.sun.glass.ui.View.notifyMouse(View.java:942)

     at com.sun.glass.ui.win.WinApplication._runLoop(Native Method)

     at com.sun.glass.ui.win.WinApplication.lambda$runLoop$3(WinApplication.java:174)

     at java.base/java.lang.Thread.run(Thread.java:829)

 Caused by: java.lang.OutOfMemoryError

     at java.base/jdk.internal.reflect.GeneratedConstructorAccessor3.newInstance(Unknown Source)

     at java.base/jdk.internal.reflect.DelegatingConstructorAccessorImpl.newInstance(DelegatingConstructorAccessorImpl.java:45)

     at java.base/java.lang.reflect.Constructor.newInstance(Constructor.java:490)

     at java.base/java.util.concurrent.ForkJoinTask.getThrowableException(ForkJoinTask.java:603)

     ... 49 more

 Caused by: java.lang.OutOfMemoryError: Java heap space

 java.util.concurrent.ExecutionException: java.lang.OutOfMemoryError

     at java.base/java.util.concurrent.ForkJoinTask.get(ForkJoinTask.java:1006)

     at fr.univ_nantes.codex_processor.gui.DatasetExtendedDepthOfFieldProcessor.run(DatasetExtendedDepthOfFieldProcessor.java:69)

     at fr.univ_nantes.codex_processor.gui.MainController$1.handle(MainController.java:96)

     at fr.univ_nantes.codex_processor.gui.MainController$1.handle(MainController.java:64)

     at com.sun.javafx.event.CompositeEventHandler.dispatchBubblingEvent(CompositeEventHandler.java:86)

     at com.sun.javafx.event.EventHandlerManager.dispatchBubblingEvent(EventHandlerManager.java:234)

     at com.sun.javafx.event.EventHandlerManager.dispatchBubblingEvent(EventHandlerManager.java:191)

     at com.sun.javafx.event.CompositeEventDispatcher.dispatchBubblingEvent(CompositeEventDispatcher.java:59)

     at com.sun.javafx.event.BasicEventDispatcher.dispatchEvent(BasicEventDispatcher.java:58)

     at com.sun.javafx.event.EventDispatchChainImpl.dispatchEvent(EventDispatchChainImpl.java:114)

     at com.sun.javafx.event.BasicEventDispatcher.dispatchEvent(BasicEventDispatcher.java:56)

     at com.sun.javafx.event.EventDispatchChainImpl.dispatchEvent(EventDispatchChainImpl.java:114)

     at com.sun.javafx.event.BasicEventDispatcher.dispatchEvent(BasicEventDispatcher.java:56)

     at com.sun.javafx.event.EventDispatchChainImpl.dispatchEvent(EventDispatchChainImpl.java:114)

     at com.sun.javafx.event.EventUtil.fireEventImpl(EventUtil.java:74)

     at com.sun.javafx.event.EventUtil.fireEvent(EventUtil.java:49)

     at javafx.event.Event.fireEvent(Event.java:198)

     at javafx.scene.Node.fireEvent(Node.java:8889)

     at javafx.scene.control.Button.fire(Button.java:203)

     at com.sun.javafx.scene.control.behavior.ButtonBehavior.mouseReleased(ButtonBehavior.java:208)

     at com.sun.javafx.scene.control.inputmap.InputMap.handle(InputMap.java:274)

     at com.sun.javafx.event.CompositeEventHandler$NormalEventHandlerRecord.handleBubblingEvent(CompositeEventHandler.java:247)

     at com.sun.javafx.event.CompositeEventHandler.dispatchBubblingEvent(CompositeEventHandler.java:80)

     at com.sun.javafx.event.EventHandlerManager.dispatchBubblingEvent(EventHandlerManager.java:234)

     at com.sun.javafx.event.EventHandlerManager.dispatchBubblingEvent(EventHandlerManager.java:191)

     at com.sun.javafx.event.CompositeEventDispatcher.dispatchBubblingEvent(CompositeEventDispatcher.java:59)

     at com.sun.javafx.event.BasicEventDispatcher.dispatchEvent(BasicEventDispatcher.java:58)

     at com.sun.javafx.event.EventDispatchChainImpl.dispatchEvent(EventDispatchChainImpl.java:114)

     at com.sun.javafx.event.BasicEventDispatcher.dispatchEvent(BasicEventDispatcher.java:56)

     at com.sun.javafx.event.EventDispatchChainImpl.dispatchEvent(EventDispatchChainImpl.java:114)

     at com.sun.javafx.event.BasicEventDispatcher.dispatchEvent(BasicEventDispatcher.java:56)

     at com.sun.javafx.event.EventDispatchChainImpl.dispatchEvent(EventDispatchChainImpl.java:114)

     at com.sun.javafx.event.EventUtil.fireEventImpl(EventUtil.java:74)

     at com.sun.javafx.event.EventUtil.fireEvent(EventUtil.java:54)

     at javafx.event.Event.fireEvent(Event.java:198)

     at javafx.scene.Scene$MouseHandler.process(Scene.java:3856)

     at javafx.scene.Scene.processMouseEvent(Scene.java:1851)

     at javafx.scene.Scene$ScenePeerListener.mouseEvent(Scene.java:2584)

     at com.sun.javafx.tk.quantum.GlassViewEventHandler$MouseEventNotification.run(GlassViewEventHandler.java:409)

     at com.sun.javafx.tk.quantum.GlassViewEventHandler$MouseEventNotification.run(GlassViewEventHandler.java:299)

     at java.base/java.security.AccessController.doPrivileged(Native Method)

     at com.sun.javafx.tk.quantum.GlassViewEventHandler.lambda$handleMouseEvent$2(GlassViewEventHandler.java:447)

     at com.sun.javafx.tk.quantum.QuantumToolkit.runWithoutRenderLock(QuantumToolkit.java:412)

     at com.sun.javafx.tk.quantum.GlassViewEventHandler.handleMouseEvent(GlassViewEventHandler.java:446)

     at com.sun.glass.ui.View.handleMouseEvent(View.java:556)

     at com.sun.glass.ui.View.notifyMouse(View.java:942)

     at com.sun.glass.ui.win.WinApplication._runLoop(Native Method)

     at com.sun.glass.ui.win.WinApplication.lambda$runLoop$3(WinApplication.java:174)

     at java.base/java.lang.Thread.run(Thread.java:829)

 Caused by: java.lang.OutOfMemoryError

     at java.base/jdk.internal.reflect.GeneratedConstructorAccessor3.newInstance(Unknown Source)

     at java.base/jdk.internal.reflect.DelegatingConstructorAccessorImpl.newInstance(DelegatingConstructorAccessorImpl.java:45)

     at java.base/java.lang.reflect.Constructor.newInstance(Constructor.java:490)

     at java.base/java.util.concurrent.ForkJoinTask.getThrowableException(ForkJoinTask.java:603)

     ... 49 more

 Caused by: java.lang.OutOfMemoryError: Java heap space

 java.util.concurrent.ExecutionException: java.lang.OutOfMemoryError

     at java.base/java.util.concurrent.ForkJoinTask.get(ForkJoinTask.java:1006)

     at fr.univ_nantes.codex_processor.gui.DatasetExtendedDepthOfFieldProcessor.run(DatasetExtendedDepthOfFieldProcessor.java:69)

     at fr.univ_nantes.codex_processor.gui.MainController$1.handle(MainController.java:96)

     at fr.univ_nantes.codex_processor.gui.MainController$1.handle(MainController.java:64)

     at com.sun.javafx.event.CompositeEventHandler.dispatchBubblingEvent(CompositeEventHandler.java:86)

     at com.sun.javafx.event.EventHandlerManager.dispatchBubblingEvent(EventHandlerManager.java:234)

     at com.sun.javafx.event.EventHandlerManager.dispatchBubblingEvent(EventHandlerManager.java:191)

     at com.sun.javafx.event.CompositeEventDispatcher.dispatchBubblingEvent(CompositeEventDispatcher.java:59)

     at com.sun.javafx.event.BasicEventDispatcher.dispatchEvent(BasicEventDispatcher.java:58)

     at com.sun.javafx.event.EventDispatchChainImpl.dispatchEvent(EventDispatchChainImpl.java:114)

     at com.sun.javafx.event.BasicEventDispatcher.dispatchEvent(BasicEventDispatcher.java:56)

     at com.sun.javafx.event.EventDispatchChainImpl.dispatchEvent(EventDispatchChainImpl.java:114)

     at com.sun.javafx.event.BasicEventDispatcher.dispatchEvent(BasicEventDispatcher.java:56)

     at com.sun.javafx.event.EventDispatchChainImpl.dispatchEvent(EventDispatchChainImpl.java:114)

     at com.sun.javafx.event.EventUtil.fireEventImpl(EventUtil.java:74)

     at com.sun.javafx.event.EventUtil.fireEvent(EventUtil.java:49)

     at javafx.event.Event.fireEvent(Event.java:198)

     at javafx.scene.Node.fireEvent(Node.java:8889)

     at javafx.scene.control.Button.fire(Button.java:203)

     at com.sun.javafx.scene.control.behavior.ButtonBehavior.mouseReleased(ButtonBehavior.java:208)

     at com.sun.javafx.scene.control.inputmap.InputMap.handle(InputMap.java:274)

     at com.sun.javafx.event.CompositeEventHandler$NormalEventHandlerRecord.handleBubblingEvent(CompositeEventHandler.java:247)

     at com.sun.javafx.event.CompositeEventHandler.dispatchBubblingEvent(CompositeEventHandler.java:80)

     at com.sun.javafx.event.EventHandlerManager.dispatchBubblingEvent(EventHandlerManager.java:234)

     at com.sun.javafx.event.EventHandlerManager.dispatchBubblingEvent(EventHandlerManager.java:191)

     at com.sun.javafx.event.CompositeEventDispatcher.dispatchBubblingEvent(CompositeEventDispatcher.java:59)

     at com.sun.javafx.event.BasicEventDispatcher.dispatchEvent(BasicEventDispatcher.java:58)

     at com.sun.javafx.event.EventDispatchChainImpl.dispatchEvent(EventDispatchChainImpl.java:114)

     at com.sun.javafx.event.BasicEventDispatcher.dispatchEvent(BasicEventDispatcher.java:56)

     at com.sun.javafx.event.EventDispatchChainImpl.dispatchEvent(EventDispatchChainImpl.java:114)

     at com.sun.javafx.event.BasicEventDispatcher.dispatchEvent(BasicEventDispatcher.java:56)

     at com.sun.javafx.event.EventDispatchChainImpl.dispatchEvent(EventDispatchChainImpl.java:114)

     at com.sun.javafx.event.EventUtil.fireEventImpl(EventUtil.java:74)

     at com.sun.javafx.event.EventUtil.fireEvent(EventUtil.java:54)

     at javafx.event.Event.fireEvent(Event.java:198)

     at javafx.scene.Scene$MouseHandler.process(Scene.java:3856)

     at javafx.scene.Scene.processMouseEvent(Scene.java:1851)

     at javafx.scene.Scene$ScenePeerListener.mouseEvent(Scene.java:2584)

     at com.sun.javafx.tk.quantum.GlassViewEventHandler$MouseEventNotification.run(GlassViewEventHandler.java:409)

     at com.sun.javafx.tk.quantum.GlassViewEventHandler$MouseEventNotification.run(GlassViewEventHandler.java:299)

     at java.base/java.security.AccessController.doPrivileged(Native Method)

     at com.sun.javafx.tk.quantum.GlassViewEventHandler.lambda$handleMouseEvent$2(GlassViewEventHandler.java:447)

     at com.sun.javafx.tk.quantum.QuantumToolkit.runWithoutRenderLock(QuantumToolkit.java:412)

     at com.sun.javafx.tk.quantum.GlassViewEventHandler.handleMouseEvent(GlassViewEventHandler.java:446)

     at com.sun.glass.ui.View.handleMouseEvent(View.java:556)

     at com.sun.glass.ui.View.notifyMouse(View.java:942)

     at com.sun.glass.ui.win.WinApplication._runLoop(Native Method)

     at com.sun.glass.ui.win.WinApplication.lambda$runLoop$3(WinApplication.java:174)

     at java.base/java.lang.Thread.run(Thread.java:829)

 Caused by: java.lang.OutOfMemoryError

     at java.base/jdk.internal.reflect.GeneratedConstructorAccessor3.newInstance(Unknown Source)

     at java.base/jdk.internal.reflect.DelegatingConstructorAccessorImpl.newInstance(DelegatingConstructorAccessorImpl.java:45)

     at java.base/java.lang.reflect.Constructor.newInstance(Constructor.java:490)

     at java.base/java.util.concurrent.ForkJoinTask.getThrowableException(ForkJoinTask.java:603)

     ... 49 more

 Caused by: java.lang.OutOfMemoryError: Java heap space

 java.util.concurrent.ExecutionException: java.lang.OutOfMemoryError

     at java.base/java.util.concurrent.ForkJoinTask.get(ForkJoinTask.java:1006)

     at fr.univ_nantes.codex_processor.gui.DatasetExtendedDepthOfFieldProcessor.run(DatasetExtendedDepthOfFieldProcessor.java:69)

     at fr.univ_nantes.codex_processor.gui.MainController$1.handle(MainController.java:96)

     at fr.univ_nantes.codex_processor.gui.MainController$1.handle(MainController.java:64)

     at com.sun.javafx.event.CompositeEventHandler.dispatchBubblingEvent(CompositeEventHandler.java:86)

     at com.sun.javafx.event.EventHandlerManager.dispatchBubblingEvent(EventHandlerManager.java:234)

     at com.sun.javafx.event.EventHandlerManager.dispatchBubblingEvent(EventHandlerManager.java:191)

     at com.sun.javafx.event.CompositeEventDispatcher.dispatchBubblingEvent(CompositeEventDispatcher.java:59)

     at com.sun.javafx.event.BasicEventDispatcher.dispatchEvent(BasicEventDispatcher.java:58)

     at com.sun.javafx.event.EventDispatchChainImpl.dispatchEvent(EventDispatchChainImpl.java:114)

     at com.sun.javafx.event.BasicEventDispatcher.dispatchEvent(BasicEventDispatcher.java:56)

     at com.sun.javafx.event.EventDispatchChainImpl.dispatchEvent(EventDispatchChainImpl.java:114)

     at com.sun.javafx.event.BasicEventDispatcher.dispatchEvent(BasicEventDispatcher.java:56)

     at com.sun.javafx.event.EventDispatchChainImpl.dispatchEvent(EventDispatchChainImpl.java:114)

     at com.sun.javafx.event.EventUtil.fireEventImpl(EventUtil.java:74)

     at com.sun.javafx.event.EventUtil.fireEvent(EventUtil.java:49)

     at javafx.event.Event.fireEvent(Event.java:198)

     at javafx.scene.Node.fireEvent(Node.java:8889)

     at javafx.scene.control.Button.fire(Button.java:203)

     at com.sun.javafx.scene.control.behavior.ButtonBehavior.mouseReleased(ButtonBehavior.java:208)

     at com.sun.javafx.scene.control.inputmap.InputMap.handle(InputMap.java:274)

     at com.sun.javafx.event.CompositeEventHandler$NormalEventHandlerRecord.handleBubblingEvent(CompositeEventHandler.java:247)

     at com.sun.javafx.event.CompositeEventHandler.dispatchBubblingEvent(CompositeEventHandler.java:80)

     at com.sun.javafx.event.EventHandlerManager.dispatchBubblingEvent(EventHandlerManager.java:234)

     at com.sun.javafx.event.EventHandlerManager.dispatchBubblingEvent(EventHandlerManager.java:191)

     at com.sun.javafx.event.CompositeEventDispatcher.dispatchBubblingEvent(CompositeEventDispatcher.java:59)

     at com.sun.javafx.event.BasicEventDispatcher.dispatchEvent(BasicEventDispatcher.java:58)

     at com.sun.javafx.event.EventDispatchChainImpl.dispatchEvent(EventDispatchChainImpl.java:114)

     at com.sun.javafx.event.BasicEventDispatcher.dispatchEvent(BasicEventDispatcher.java:56)

     at com.sun.javafx.event.EventDispatchChainImpl.dispatchEvent(EventDispatchChainImpl.java:114)

     at com.sun.javafx.event.BasicEventDispatcher.dispatchEvent(BasicEventDispatcher.java:56)

     at com.sun.javafx.event.EventDispatchChainImpl.dispatchEvent(EventDispatchChainImpl.java:114)

     at com.sun.javafx.event.EventUtil.fireEventImpl(EventUtil.java:74)

     at com.sun.javafx.event.EventUtil.fireEvent(EventUtil.java:54)

     at javafx.event.Event.fireEvent(Event.java:198)

     at javafx.scene.Scene$MouseHandler.process(Scene.java:3856)

     at javafx.scene.Scene.processMouseEvent(Scene.java:1851)

     at javafx.scene.Scene$ScenePeerListener.mouseEvent(Scene.java:2584)

     at com.sun.javafx.tk.quantum.GlassViewEventHandler$MouseEventNotification.run(GlassViewEventHandler.java:409)

     at com.sun.javafx.tk.quantum.GlassViewEventHandler$MouseEventNotification.run(GlassViewEventHandler.java:299)

     at java.base/java.security.AccessController.doPrivileged(Native Method)

     at com.sun.javafx.tk.quantum.GlassViewEventHandler.lambda$handleMouseEvent$2(GlassViewEventHandler.java:447)

     at com.sun.javafx.tk.quantum.QuantumToolkit.runWithoutRenderLock(QuantumToolkit.java:412)

     at com.sun.javafx.tk.quantum.GlassViewEventHandler.handleMouseEvent(GlassViewEventHandler.java:446)

     at com.sun.glass.ui.View.handleMouseEvent(View.java:556)

     at com.sun.glass.ui.View.notifyMouse(View.java:942)

     at com.sun.glass.ui.win.WinApplication._runLoop(Native Method)

     at com.sun.glass.ui.win.WinApplication.lambda$runLoop$3(WinApplication.java:174)

     at java.base/java.lang.Thread.run(Thread.java:829)

 Caused by: java.lang.OutOfMemoryError

     at java.base/jdk.internal.reflect.GeneratedConstructorAccessor3.newInstance(Unknown Source)

     at java.base/jdk.internal.reflect.DelegatingConstructorAccessorImpl.newInstance(DelegatingConstructorAccessorImpl.java:45)

     at java.base/java.lang.reflect.Constructor.newInstance(Constructor.java:490)

     at java.base/java.util.concurrent.ForkJoinTask.getThrowableException(ForkJoinTask.java:603)

     ... 49 more

 Caused by: java.lang.OutOfMemoryError: Java heap space

 java.util.concurrent.ExecutionException: java.lang.OutOfMemoryError

     at java.base/java.util.concurrent.ForkJoinTask.get(ForkJoinTask.java:1006)

     at fr.univ_nantes.codex_processor.gui.DatasetExtendedDepthOfFieldProcessor.run(DatasetExtendedDepthOfFieldProcessor.java:69)

     at fr.univ_nantes.codex_processor.gui.MainController$1.handle(MainController.java:96)

     at fr.univ_nantes.codex_processor.gui.MainController$1.handle(MainController.java:64)

     at com.sun.javafx.event.CompositeEventHandler.dispatchBubblingEvent(CompositeEventHandler.java:86)

     at com.sun.javafx.event.EventHandlerManager.dispatchBubblingEvent(EventHandlerManager.java:234)

     at com.sun.javafx.event.EventHandlerManager.dispatchBubblingEvent(EventHandlerManager.java:191)

     at com.sun.javafx.event.CompositeEventDispatcher.dispatchBubblingEvent(CompositeEventDispatcher.java:59)

     at com.sun.javafx.event.BasicEventDispatcher.dispatchEvent(BasicEventDispatcher.java:58)

     at com.sun.javafx.event.EventDispatchChainImpl.dispatchEvent(EventDispatchChainImpl.java:114)

     at com.sun.javafx.event.BasicEventDispatcher.dispatchEvent(BasicEventDispatcher.java:56)

     at com.sun.javafx.event.EventDispatchChainImpl.dispatchEvent(EventDispatchChainImpl.java:114)

     at com.sun.javafx.event.BasicEventDispatcher.dispatchEvent(BasicEventDispatcher.java:56)

     at com.sun.javafx.event.EventDispatchChainImpl.dispatchEvent(EventDispatchChainImpl.java:114)

     at com.sun.javafx.event.EventUtil.fireEventImpl(EventUtil.java:74)

     at com.sun.javafx.event.EventUtil.fireEvent(EventUtil.java:49)

     at javafx.event.Event.fireEvent(Event.java:198)

     at javafx.scene.Node.fireEvent(Node.java:8889)

     at javafx.scene.control.Button.fire(Button.java:203)

     at com.sun.javafx.scene.control.behavior.ButtonBehavior.mouseReleased(ButtonBehavior.java:208)

     at com.sun.javafx.scene.control.inputmap.InputMap.handle(InputMap.java:274)

     at com.sun.javafx.event.CompositeEventHandler$NormalEventHandlerRecord.handleBubblingEvent(CompositeEventHandler.java:247)

     at com.sun.javafx.event.CompositeEventHandler.dispatchBubblingEvent(CompositeEventHandler.java:80)

     at com.sun.javafx.event.EventHandlerManager.dispatchBubblingEvent(EventHandlerManager.java:234)

     at com.sun.javafx.event.EventHandlerManager.dispatchBubblingEvent(EventHandlerManager.java:191)

     at com.sun.javafx.event.CompositeEventDispatcher.dispatchBubblingEvent(CompositeEventDispatcher.java:59)

     at com.sun.javafx.event.BasicEventDispatcher.dispatchEvent(BasicEventDispatcher.java:58)

     at com.sun.javafx.event.EventDispatchChainImpl.dispatchEvent(EventDispatchChainImpl.java:114)

     at com.sun.javafx.event.BasicEventDispatcher.dispatchEvent(BasicEventDispatcher.java:56)

     at com.sun.javafx.event.EventDispatchChainImpl.dispatchEvent(EventDispatchChainImpl.java:114)

     at com.sun.javafx.event.BasicEventDispatcher.dispatchEvent(BasicEventDispatcher.java:56)

     at com.sun.javafx.event.EventDispatchChainImpl.dispatchEvent(EventDispatchChainImpl.java:114)

     at com.sun.javafx.event.EventUtil.fireEventImpl(EventUtil.java:74)

     at com.sun.javafx.event.EventUtil.fireEvent(EventUtil.java:54)

     at javafx.event.Event.fireEvent(Event.java:198)

     at javafx.scene.Scene$MouseHandler.process(Scene.java:3856)

     at javafx.scene.Scene.processMouseEvent(Scene.java:1851)

     at javafx.scene.Scene$ScenePeerListener.mouseEvent(Scene.java:2584)

     at com.sun.javafx.tk.quantum.GlassViewEventHandler$MouseEventNotification.run(GlassViewEventHandler.java:409)

     at com.sun.javafx.tk.quantum.GlassViewEventHandler$MouseEventNotification.run(GlassViewEventHandler.java:299)

     at java.base/java.security.AccessController.doPrivileged(Native Method)

     at com.sun.javafx.tk.quantum.GlassViewEventHandler.lambda$handleMouseEvent$2(GlassViewEventHandler.java:447)

     at com.sun.javafx.tk.quantum.QuantumToolkit.runWithoutRenderLock(QuantumToolkit.java:412)

     at com.sun.javafx.tk.quantum.GlassViewEventHandler.handleMouseEvent(GlassViewEventHandler.java:446)

     at com.sun.glass.ui.View.handleMouseEvent(View.java:556)

     at com.sun.glass.ui.View.notifyMouse(View.java:942)

     at com.sun.glass.ui.win.WinApplication._runLoop(Native Method)

     at com.sun.glass.ui.win.WinApplication.lambda$runLoop$3(WinApplication.java:174)

     at java.base/java.lang.Thread.run(Thread.java:829)

 Caused by: java.lang.OutOfMemoryError

     at java.base/jdk.internal.reflect.GeneratedConstructorAccessor3.newInstance(Unknown Source)

     at java.base/jdk.internal.reflect.DelegatingConstructorAccessorImpl.newInstance(DelegatingConstructorAccessorImpl.java:45)

     at java.base/java.lang.reflect.Constructor.newInstance(Constructor.java:490)

     at java.base/java.util.concurrent.ForkJoinTask.getThrowableException(ForkJoinTask.java:603)

     ... 49 more

 Caused by: java.lang.OutOfMemoryError: Java heap space

 java.util.concurrent.ExecutionException: java.lang.OutOfMemoryError

     at java.base/java.util.concurrent.ForkJoinTask.get(ForkJoinTask.java:1006)

     at fr.univ_nantes.codex_processor.gui.DatasetExtendedDepthOfFieldProcessor.run(DatasetExtendedDepthOfFieldProcessor.java:69)

     at fr.univ_nantes.codex_processor.gui.MainController$1.handle(MainController.java:96)

     at fr.univ_nantes.codex_processor.gui.MainController$1.handle(MainController.java:64)

     at com.sun.javafx.event.CompositeEventHandler.dispatchBubblingEvent(CompositeEventHandler.java:86)

     at com.sun.javafx.event.EventHandlerManager.dispatchBubblingEvent(EventHandlerManager.java:234)

     at com.sun.javafx.event.EventHandlerManager.dispatchBubblingEvent(EventHandlerManager.java:191)

     at com.sun.javafx.event.CompositeEventDispatcher.dispatchBubblingEvent(CompositeEventDispatcher.java:59)

     at com.sun.javafx.event.BasicEventDispatcher.dispatchEvent(BasicEventDispatcher.java:58)

     at com.sun.javafx.event.EventDispatchChainImpl.dispatchEvent(EventDispatchChainImpl.java:114)

     at com.sun.javafx.event.BasicEventDispatcher.dispatchEvent(BasicEventDispatcher.java:56)

     at com.sun.javafx.event.EventDispatchChainImpl.dispatchEvent(EventDispatchChainImpl.java:114)

     at com.sun.javafx.event.BasicEventDispatcher.dispatchEvent(BasicEventDispatcher.java:56)

     at com.sun.javafx.event.EventDispatchChainImpl.dispatchEvent(EventDispatchChainImpl.java:114)

     at com.sun.javafx.event.EventUtil.fireEventImpl(EventUtil.java:74)

     at com.sun.javafx.event.EventUtil.fireEvent(EventUtil.java:49)

     at javafx.event.Event.fireEvent(Event.java:198)

     at javafx.scene.Node.fireEvent(Node.java:8889)

     at javafx.scene.control.Button.fire(Button.java:203)

     at com.sun.javafx.scene.control.behavior.ButtonBehavior.mouseReleased(ButtonBehavior.java:208)

     at com.sun.javafx.scene.control.inputmap.InputMap.handle(InputMap.java:274)

     at com.sun.javafx.event.CompositeEventHandler$NormalEventHandlerRecord.handleBubblingEvent(CompositeEventHandler.java:247)

     at com.sun.javafx.event.CompositeEventHandler.dispatchBubblingEvent(CompositeEventHandler.java:80)

     at com.sun.javafx.event.EventHandlerManager.dispatchBubblingEvent(EventHandlerManager.java:234)

     at com.sun.javafx.event.EventHandlerManager.dispatchBubblingEvent(EventHandlerManager.java:191)

     at com.sun.javafx.event.CompositeEventDispatcher.dispatchBubblingEvent(CompositeEventDispatcher.java:59)

     at com.sun.javafx.event.BasicEventDispatcher.dispatchEvent(BasicEventDispatcher.java:58)

     at com.sun.javafx.event.EventDispatchChainImpl.dispatchEvent(EventDispatchChainImpl.java:114)

     at com.sun.javafx.event.BasicEventDispatcher.dispatchEvent(BasicEventDispatcher.java:56)

     at com.sun.javafx.event.EventDispatchChainImpl.dispatchEvent(EventDispatchChainImpl.java:114)

     at com.sun.javafx.event.BasicEventDispatcher.dispatchEvent(BasicEventDispatcher.java:56)

     at com.sun.javafx.event.EventDispatchChainImpl.dispatchEvent(EventDispatchChainImpl.java:114)

     at com.sun.javafx.event.EventUtil.fireEventImpl(EventUtil.java:74)

     at com.sun.javafx.event.EventUtil.fireEvent(EventUtil.java:54)

     at javafx.event.Event.fireEvent(Event.java:198)

     at javafx.scene.Scene$MouseHandler.process(Scene.java:3856)

     at javafx.scene.Scene.processMouseEvent(Scene.java:1851)

     at javafx.scene.Scene$ScenePeerListener.mouseEvent(Scene.java:2584)

     at com.sun.javafx.tk.quantum.GlassViewEventHandler$MouseEventNotification.run(GlassViewEventHandler.java:409)

     at com.sun.javafx.tk.quantum.GlassViewEventHandler$MouseEventNotification.run(GlassViewEventHandler.java:299)

     at java.base/java.security.AccessController.doPrivileged(Native Method)

     at com.sun.javafx.tk.quantum.GlassViewEventHandler.lambda$handleMouseEvent$2(GlassViewEventHandler.java:447)

     at com.sun.javafx.tk.quantum.QuantumToolkit.runWithoutRenderLock(QuantumToolkit.java:412)

     at com.sun.javafx.tk.quantum.GlassViewEventHandler.handleMouseEvent(GlassViewEventHandler.java:446)

     at com.sun.glass.ui.View.handleMouseEvent(View.java:556)

     at com.sun.glass.ui.View.notifyMouse(View.java:942)

     at com.sun.glass.ui.win.WinApplication._runLoop(Native Method)

     at com.sun.glass.ui.win.WinApplication.lambda$runLoop$3(WinApplication.java:174)

     at java.base/java.lang.Thread.run(Thread.java:829)

 Caused by: java.lang.OutOfMemoryError

     at java.base/jdk.internal.reflect.GeneratedConstructorAccessor3.newInstance(Unknown Source)

     at java.base/jdk.internal.reflect.DelegatingConstructorAccessorImpl.newInstance(DelegatingConstructorAccessorImpl.java:45)

     at java.base/java.lang.reflect.Constructor.newInstance(Constructor.java:490)

     at java.base/java.util.concurrent.ForkJoinTask.getThrowableException(ForkJoinTask.java:603)

     ... 49 more

 Caused by: java.lang.OutOfMemoryError: Java heap space

 java.util.concurrent.ExecutionException: java.lang.OutOfMemoryError

     at java.base/java.util.concurrent.ForkJoinTask.get(ForkJoinTask.java:1006)

     at fr.univ_nantes.codex_processor.gui.DatasetExtendedDepthOfFieldProcessor.run(DatasetExtendedDepthOfFieldProcessor.java:69)

     at fr.univ_nantes.codex_processor.gui.MainController$1.handle(MainController.java:96)

     at fr.univ_nantes.codex_processor.gui.MainController$1.handle(MainController.java:64)

     at com.sun.javafx.event.CompositeEventHandler.dispatchBubblingEvent(CompositeEventHandler.java:86)

     at com.sun.javafx.event.EventHandlerManager.dispatchBubblingEvent(EventHandlerManager.java:234)

     at com.sun.javafx.event.EventHandlerManager.dispatchBubblingEvent(EventHandlerManager.java:191)

     at com.sun.javafx.event.CompositeEventDispatcher.dispatchBubblingEvent(CompositeEventDispatcher.java:59)

     at com.sun.javafx.event.BasicEventDispatcher.dispatchEvent(BasicEventDispatcher.java:58)

     at com.sun.javafx.event.EventDispatchChainImpl.dispatchEvent(EventDispatchChainImpl.java:114)

     at com.sun.javafx.event.BasicEventDispatcher.dispatchEvent(BasicEventDispatcher.java:56)

     at com.sun.javafx.event.EventDispatchChainImpl.dispatchEvent(EventDispatchChainImpl.java:114)

     at com.sun.javafx.event.BasicEventDispatcher.dispatchEvent(BasicEventDispatcher.java:56)

     at com.sun.javafx.event.EventDispatchChainImpl.dispatchEvent(EventDispatchChainImpl.java:114)

     at com.sun.javafx.event.EventUtil.fireEventImpl(EventUtil.java:74)

     at com.sun.javafx.event.EventUtil.fireEvent(EventUtil.java:49)

     at javafx.event.Event.fireEvent(Event.java:198)

     at javafx.scene.Node.fireEvent(Node.java:8889)

     at javafx.scene.control.Button.fire(Button.java:203)

     at com.sun.javafx.scene.control.behavior.ButtonBehavior.mouseReleased(ButtonBehavior.java:208)

     at com.sun.javafx.scene.control.inputmap.InputMap.handle(InputMap.java:274)

     at com.sun.javafx.event.CompositeEventHandler$NormalEventHandlerRecord.handleBubblingEvent(CompositeEventHandler.java:247)

     at com.sun.javafx.event.CompositeEventHandler.dispatchBubblingEvent(CompositeEventHandler.java:80)

     at com.sun.javafx.event.EventHandlerManager.dispatchBubblingEvent(EventHandlerManager.java:234)

     at com.sun.javafx.event.EventHandlerManager.dispatchBubblingEvent(EventHandlerManager.java:191)

     at com.sun.javafx.event.CompositeEventDispatcher.dispatchBubblingEvent(CompositeEventDispatcher.java:59)

     at com.sun.javafx.event.BasicEventDispatcher.dispatchEvent(BasicEventDispatcher.java:58)

     at com.sun.javafx.event.EventDispatchChainImpl.dispatchEvent(EventDispatchChainImpl.java:114)

     at com.sun.javafx.event.BasicEventDispatcher.dispatchEvent(BasicEventDispatcher.java:56)

     at com.sun.javafx.event.EventDispatchChainImpl.dispatchEvent(EventDispatchChainImpl.java:114)

     at com.sun.javafx.event.BasicEventDispatcher.dispatchEvent(BasicEventDispatcher.java:56)

     at com.sun.javafx.event.EventDispatchChainImpl.dispatchEvent(EventDispatchChainImpl.java:114)

     at com.sun.javafx.event.EventUtil.fireEventImpl(EventUtil.java:74)

     at com.sun.javafx.event.EventUtil.fireEvent(EventUtil.java:54)

     at javafx.event.Event.fireEvent(Event.java:198)

     at javafx.scene.Scene$MouseHandler.process(Scene.java:3856)

     at javafx.scene.Scene.processMouseEvent(Scene.java:1851)

     at javafx.scene.Scene$ScenePeerListener.mouseEvent(Scene.java:2584)

     at com.sun.javafx.tk.quantum.GlassViewEventHandler$MouseEventNotification.run(GlassViewEventHandler.java:409)

     at com.sun.javafx.tk.quantum.GlassViewEventHandler$MouseEventNotification.run(GlassViewEventHandler.java:299)

     at java.base/java.security.AccessController.doPrivileged(Native Method)

     at com.sun.javafx.tk.quantum.GlassViewEventHandler.lambda$handleMouseEvent$2(GlassViewEventHandler.java:447)

     at com.sun.javafx.tk.quantum.QuantumToolkit.runWithoutRenderLock(QuantumToolkit.java:412)

     at com.sun.javafx.tk.quantum.GlassViewEventHandler.handleMouseEvent(GlassViewEventHandler.java:446)

     at com.sun.glass.ui.View.handleMouseEvent(View.java:556)

     at com.sun.glass.ui.View.notifyMouse(View.java:942)

     at com.sun.glass.ui.win.WinApplication._runLoop(Native Method)

     at com.sun.glass.ui.win.WinApplication.lambda$runLoop$3(WinApplication.java:174)

     at java.base/java.lang.Thread.run(Thread.java:829)

 Caused by: java.lang.OutOfMemoryError

     at java.base/jdk.internal.reflect.GeneratedConstructorAccessor3.newInstance(Unknown Source)

     at java.base/jdk.internal.reflect.DelegatingConstructorAccessorImpl.newInstance(DelegatingConstructorAccessorImpl.java:45)

     at java.base/java.lang.reflect.Constructor.newInstance(Constructor.java:490)

     at java.base/java.util.concurrent.ForkJoinTask.getThrowableException(ForkJoinTask.java:603)

     ... 49 more

 Caused by: java.lang.OutOfMemoryError: Java heap space

 java.util.concurrent.ExecutionException: java.lang.OutOfMemoryError

     at java.base/java.util.concurrent.ForkJoinTask.get(ForkJoinTask.java:1006)

     at fr.univ_nantes.codex_processor.gui.DatasetExtendedDepthOfFieldProcessor.run(DatasetExtendedDepthOfFieldProcessor.java:69)

     at fr.univ_nantes.codex_processor.gui.MainController$1.handle(MainController.java:96)

     at fr.univ_nantes.codex_processor.gui.MainController$1.handle(MainController.java:64)

     at com.sun.javafx.event.CompositeEventHandler.dispatchBubblingEvent(CompositeEventHandler.java:86)

     at com.sun.javafx.event.EventHandlerManager.dispatchBubblingEvent(EventHandlerManager.java:234)

     at com.sun.javafx.event.EventHandlerManager.dispatchBubblingEvent(EventHandlerManager.java:191)

     at com.sun.javafx.event.CompositeEventDispatcher.dispatchBubblingEvent(CompositeEventDispatcher.java:59)

     at com.sun.javafx.event.BasicEventDispatcher.dispatchEvent(BasicEventDispatcher.java:58)

     at com.sun.javafx.event.EventDispatchChainImpl.dispatchEvent(EventDispatchChainImpl.java:114)

     at com.sun.javafx.event.BasicEventDispatcher.dispatchEvent(BasicEventDispatcher.java:56)

     at com.sun.javafx.event.EventDispatchChainImpl.dispatchEvent(EventDispatchChainImpl.java:114)

     at com.sun.javafx.event.BasicEventDispatcher.dispatchEvent(BasicEventDispatcher.java:56)

     at com.sun.javafx.event.EventDispatchChainImpl.dispatchEvent(EventDispatchChainImpl.java:114)

     at com.sun.javafx.event.EventUtil.fireEventImpl(EventUtil.java:74)

     at com.sun.javafx.event.EventUtil.fireEvent(EventUtil.java:49)

     at javafx.event.Event.fireEvent(Event.java:198)

     at javafx.scene.Node.fireEvent(Node.java:8889)

     at javafx.scene.control.Button.fire(Button.java:203)

     at com.sun.javafx.scene.control.behavior.ButtonBehavior.mouseReleased(ButtonBehavior.java:208)

     at com.sun.javafx.scene.control.inputmap.InputMap.handle(InputMap.java:274)

     at com.sun.javafx.event.CompositeEventHandler$NormalEventHandlerRecord.handleBubblingEvent(CompositeEventHandler.java:247)

     at com.sun.javafx.event.CompositeEventHandler.dispatchBubblingEvent(CompositeEventHandler.java:80)

     at com.sun.javafx.event.EventHandlerManager.dispatchBubblingEvent(EventHandlerManager.java:234)

     at com.sun.javafx.event.EventHandlerManager.dispatchBubblingEvent(EventHandlerManager.java:191)

     at com.sun.javafx.event.CompositeEventDispatcher.dispatchBubblingEvent(CompositeEventDispatcher.java:59)

     at com.sun.javafx.event.BasicEventDispatcher.dispatchEvent(BasicEventDispatcher.java:58)

     at com.sun.javafx.event.EventDispatchChainImpl.dispatchEvent(EventDispatchChainImpl.java:114)

     at com.sun.javafx.event.BasicEventDispatcher.dispatchEvent(BasicEventDispatcher.java:56)

     at com.sun.javafx.event.EventDispatchChainImpl.dispatchEvent(EventDispatchChainImpl.java:114)

     at com.sun.javafx.event.BasicEventDispatcher.dispatchEvent(BasicEventDispatcher.java:56)

     at com.sun.javafx.event.EventDispatchChainImpl.dispatchEvent(EventDispatchChainImpl.java:114)

     at com.sun.javafx.event.EventUtil.fireEventImpl(EventUtil.java:74)

     at com.sun.javafx.event.EventUtil.fireEvent(EventUtil.java:54)

     at javafx.event.Event.fireEvent(Event.java:198)

     at javafx.scene.Scene$MouseHandler.process(Scene.java:3856)

     at javafx.scene.Scene.processMouseEvent(Scene.java:1851)

     at javafx.scene.Scene$ScenePeerListener.mouseEvent(Scene.java:2584)

     at com.sun.javafx.tk.quantum.GlassViewEventHandler$MouseEventNotification.run(GlassViewEventHandler.java:409)

     at com.sun.javafx.tk.quantum.GlassViewEventHandler$MouseEventNotification.run(GlassViewEventHandler.java:299)

     at java.base/java.security.AccessController.doPrivileged(Native Method)

     at com.sun.javafx.tk.quantum.GlassViewEventHandler.lambda$handleMouseEvent$2(GlassViewEventHandler.java:447)

     at com.sun.javafx.tk.quantum.QuantumToolkit.runWithoutRenderLock(QuantumToolkit.java:412)

     at com.sun.javafx.tk.quantum.GlassViewEventHandler.handleMouseEvent(GlassViewEventHandler.java:446)

     at com.sun.glass.ui.View.handleMouseEvent(View.java:556)

     at com.sun.glass.ui.View.notifyMouse(View.java:942)

     at com.sun.glass.ui.win.WinApplication._runLoop(Native Method)

     at com.sun.glass.ui.win.WinApplication.lambda$runLoop$3(WinApplication.java:174)

     at java.base/java.lang.Thread.run(Thread.java:829)

 Caused by: java.lang.OutOfMemoryError

     at java.base/jdk.internal.reflect.GeneratedConstructorAccessor3.newInstance(Unknown Source)

     at java.base/jdk.internal.reflect.DelegatingConstructorAccessorImpl.newInstance(DelegatingConstructorAccessorImpl.java:45)

     at java.base/java.lang.reflect.Constructor.newInstance(Constructor.java:490)

     at java.base/java.util.concurrent.ForkJoinTask.getThrowableException(ForkJoinTask.java:603)

     ... 49 more

 Caused by: java.lang.OutOfMemoryError: Java heap space

 java.util.concurrent.ExecutionException: java.lang.OutOfMemoryError

     at java.base/java.util.concurrent.ForkJoinTask.get(ForkJoinTask.java:1006)

     at fr.univ_nantes.codex_processor.gui.DatasetExtendedDepthOfFieldProcessor.run(DatasetExtendedDepthOfFieldProcessor.java:69)

     at fr.univ_nantes.codex_processor.gui.MainController$1.handle(MainController.java:96)

     at fr.univ_nantes.codex_processor.gui.MainController$1.handle(MainController.java:64)

     at com.sun.javafx.event.CompositeEventHandler.dispatchBubblingEvent(CompositeEventHandler.java:86)

     at com.sun.javafx.event.EventHandlerManager.dispatchBubblingEvent(EventHandlerManager.java:234)

     at com.sun.javafx.event.EventHandlerManager.dispatchBubblingEvent(EventHandlerManager.java:191)

     at com.sun.javafx.event.CompositeEventDispatcher.dispatchBubblingEvent(CompositeEventDispatcher.java:59)

     at com.sun.javafx.event.BasicEventDispatcher.dispatchEvent(BasicEventDispatcher.java:58)

     at com.sun.javafx.event.EventDispatchChainImpl.dispatchEvent(EventDispatchChainImpl.java:114)

     at com.sun.javafx.event.BasicEventDispatcher.dispatchEvent(BasicEventDispatcher.java:56)

     at com.sun.javafx.event.EventDispatchChainImpl.dispatchEvent(EventDispatchChainImpl.java:114)

     at com.sun.javafx.event.BasicEventDispatcher.dispatchEvent(BasicEventDispatcher.java:56)

     at com.sun.javafx.event.EventDispatchChainImpl.dispatchEvent(EventDispatchChainImpl.java:114)

     at com.sun.javafx.event.EventUtil.fireEventImpl(EventUtil.java:74)

     at com.sun.javafx.event.EventUtil.fireEvent(EventUtil.java:49)

     at javafx.event.Event.fireEvent(Event.java:198)

     at javafx.scene.Node.fireEvent(Node.java:8889)

     at javafx.scene.control.Button.fire(Button.java:203)

     at com.sun.javafx.scene.control.behavior.ButtonBehavior.mouseReleased(ButtonBehavior.java:208)

     at com.sun.javafx.scene.control.inputmap.InputMap.handle(InputMap.java:274)

     at com.sun.javafx.event.CompositeEventHandler$NormalEventHandlerRecord.handleBubblingEvent(CompositeEventHandler.java:247)

     at com.sun.javafx.event.CompositeEventHandler.dispatchBubblingEvent(CompositeEventHandler.java:80)

     at com.sun.javafx.event.EventHandlerManager.dispatchBubblingEvent(EventHandlerManager.java:234)

     at com.sun.javafx.event.EventHandlerManager.dispatchBubblingEvent(EventHandlerManager.java:191)

     at com.sun.javafx.event.CompositeEventDispatcher.dispatchBubblingEvent(CompositeEventDispatcher.java:59)

     at com.sun.javafx.event.BasicEventDispatcher.dispatchEvent(BasicEventDispatcher.java:58)

     at com.sun.javafx.event.EventDispatchChainImpl.dispatchEvent(EventDispatchChainImpl.java:114)

     at com.sun.javafx.event.BasicEventDispatcher.dispatchEvent(BasicEventDispatcher.java:56)

     at com.sun.javafx.event.EventDispatchChainImpl.dispatchEvent(EventDispatchChainImpl.java:114)

     at com.sun.javafx.event.BasicEventDispatcher.dispatchEvent(BasicEventDispatcher.java:56)

     at com.sun.javafx.event.EventDispatchChainImpl.dispatchEvent(EventDispatchChainImpl.java:114)

     at com.sun.javafx.event.EventUtil.fireEventImpl(EventUtil.java:74)

     at com.sun.javafx.event.EventUtil.fireEvent(EventUtil.java:54)

     at javafx.event.Event.fireEvent(Event.java:198)

     at javafx.scene.Scene$MouseHandler.process(Scene.java:3856)

     at javafx.scene.Scene.processMouseEvent(Scene.java:1851)

     at javafx.scene.Scene$ScenePeerListener.mouseEvent(Scene.java:2584)

     at com.sun.javafx.tk.quantum.GlassViewEventHandler$MouseEventNotification.run(GlassViewEventHandler.java:409)

     at com.sun.javafx.tk.quantum.GlassViewEventHandler$MouseEventNotification.run(GlassViewEventHandler.java:299)

     at java.base/java.security.AccessController.doPrivileged(Native Method)

     at com.sun.javafx.tk.quantum.GlassViewEventHandler.lambda$handleMouseEvent$2(GlassViewEventHandler.java:447)

     at com.sun.javafx.tk.quantum.QuantumToolkit.runWithoutRenderLock(QuantumToolkit.java:412)

     at com.sun.javafx.tk.quantum.GlassViewEventHandler.handleMouseEvent(GlassViewEventHandler.java:446)

     at com.sun.glass.ui.View.handleMouseEvent(View.java:556)

     at com.sun.glass.ui.View.notifyMouse(View.java:942)

     at com.sun.glass.ui.win.WinApplication._runLoop(Native Method)

     at com.sun.glass.ui.win.WinApplication.lambda$runLoop$3(WinApplication.java:174)

     at java.base/java.lang.Thread.run(Thread.java:829)

 Caused by: java.lang.OutOfMemoryError

     at java.base/jdk.internal.reflect.GeneratedConstructorAccessor3.newInstance(Unknown Source)

     at java.base/jdk.internal.reflect.DelegatingConstructorAccessorImpl.newInstance(DelegatingConstructorAccessorImpl.java:45)

     at java.base/java.lang.reflect.Constructor.newInstance(Constructor.java:490)

     at java.base/java.util.concurrent.ForkJoinTask.getThrowableException(ForkJoinTask.java:603)

     ... 49 more

 Caused by: java.lang.OutOfMemoryError: Java heap space

 java.util.concurrent.ExecutionException: java.lang.OutOfMemoryError

     at java.base/java.util.concurrent.ForkJoinTask.get(ForkJoinTask.java:1006)

     at fr.univ_nantes.codex_processor.gui.DatasetExtendedDepthOfFieldProcessor.run(DatasetExtendedDepthOfFieldProcessor.java:69)

     at fr.univ_nantes.codex_processor.gui.MainController$1.handle(MainController.java:96)

     at fr.univ_nantes.codex_processor.gui.MainController$1.handle(MainController.java:64)

     at com.sun.javafx.event.CompositeEventHandler.dispatchBubblingEvent(CompositeEventHandler.java:86)

     at com.sun.javafx.event.EventHandlerManager.dispatchBubblingEvent(EventHandlerManager.java:234)

     at com.sun.javafx.event.EventHandlerManager.dispatchBubblingEvent(EventHandlerManager.java:191)

     at com.sun.javafx.event.CompositeEventDispatcher.dispatchBubblingEvent(CompositeEventDispatcher.java:59)

     at com.sun.javafx.event.BasicEventDispatcher.dispatchEvent(BasicEventDispatcher.java:58)

     at com.sun.javafx.event.EventDispatchChainImpl.dispatchEvent(EventDispatchChainImpl.java:114)

     at com.sun.javafx.event.BasicEventDispatcher.dispatchEvent(BasicEventDispatcher.java:56)

     at com.sun.javafx.event.EventDispatchChainImpl.dispatchEvent(EventDispatchChainImpl.java:114)

     at com.sun.javafx.event.BasicEventDispatcher.dispatchEvent(BasicEventDispatcher.java:56)

     at com.sun.javafx.event.EventDispatchChainImpl.dispatchEvent(EventDispatchChainImpl.java:114)

     at com.sun.javafx.event.EventUtil.fireEventImpl(EventUtil.java:74)

     at com.sun.javafx.event.EventUtil.fireEvent(EventUtil.java:49)

     at javafx.event.Event.fireEvent(Event.java:198)

     at javafx.scene.Node.fireEvent(Node.java:8889)

     at javafx.scene.control.Button.fire(Button.java:203)

     at com.sun.javafx.scene.control.behavior.ButtonBehavior.mouseReleased(ButtonBehavior.java:208)

     at com.sun.javafx.scene.control.inputmap.InputMap.handle(InputMap.java:274)

     at com.sun.javafx.event.CompositeEventHandler$NormalEventHandlerRecord.handleBubblingEvent(CompositeEventHandler.java:247)

     at com.sun.javafx.event.CompositeEventHandler.dispatchBubblingEvent(CompositeEventHandler.java:80)

     at com.sun.javafx.event.EventHandlerManager.dispatchBubblingEvent(EventHandlerManager.java:234)

     at com.sun.javafx.event.EventHandlerManager.dispatchBubblingEvent(EventHandlerManager.java:191)

     at com.sun.javafx.event.CompositeEventDispatcher.dispatchBubblingEvent(CompositeEventDispatcher.java:59)

     at com.sun.javafx.event.BasicEventDispatcher.dispatchEvent(BasicEventDispatcher.java:58)

     at com.sun.javafx.event.EventDispatchChainImpl.dispatchEvent(EventDispatchChainImpl.java:114)

     at com.sun.javafx.event.BasicEventDispatcher.dispatchEvent(BasicEventDispatcher.java:56)

     at com.sun.javafx.event.EventDispatchChainImpl.dispatchEvent(EventDispatchChainImpl.java:114)

     at com.sun.javafx.event.BasicEventDispatcher.dispatchEvent(BasicEventDispatcher.java:56)

     at com.sun.javafx.event.EventDispatchChainImpl.dispatchEvent(EventDispatchChainImpl.java:114)

     at com.sun.javafx.event.EventUtil.fireEventImpl(EventUtil.java:74)

     at com.sun.javafx.event.EventUtil.fireEvent(EventUtil.java:54)

     at javafx.event.Event.fireEvent(Event.java:198)

     at javafx.scene.Scene$MouseHandler.process(Scene.java:3856)

     at javafx.scene.Scene.processMouseEvent(Scene.java:1851)

     at javafx.scene.Scene$ScenePeerListener.mouseEvent(Scene.java:2584)

     at com.sun.javafx.tk.quantum.GlassViewEventHandler$MouseEventNotification.run(GlassViewEventHandler.java:409)

     at com.sun.javafx.tk.quantum.GlassViewEventHandler$MouseEventNotification.run(GlassViewEventHandler.java:299)

     at java.base/java.security.AccessController.doPrivileged(Native Method)

     at com.sun.javafx.tk.quantum.GlassViewEventHandler.lambda$handleMouseEvent$2(GlassViewEventHandler.java:447)

     at com.sun.javafx.tk.quantum.QuantumToolkit.runWithoutRenderLock(QuantumToolkit.java:412)

     at com.sun.javafx.tk.quantum.GlassViewEventHandler.handleMouseEvent(GlassViewEventHandler.java:446)

     at com.sun.glass.ui.View.handleMouseEvent(View.java:556)

     at com.sun.glass.ui.View.notifyMouse(View.java:942)

     at com.sun.glass.ui.win.WinApplication._runLoop(Native Method)

     at com.sun.glass.ui.win.WinApplication.lambda$runLoop$3(WinApplication.java:174)

     at java.base/java.lang.Thread.run(Thread.java:829)

 Caused by: java.lang.OutOfMemoryError

     at java.base/jdk.internal.reflect.GeneratedConstructorAccessor3.newInstance(Unknown Source)

     at java.base/jdk.internal.reflect.DelegatingConstructorAccessorImpl.newInstance(DelegatingConstructorAccessorImpl.java:45)

     at java.base/java.lang.reflect.Constructor.newInstance(Constructor.java:490)

     at java.base/java.util.concurrent.ForkJoinTask.getThrowableException(ForkJoinTask.java:603)

     ... 49 more

 Caused by: java.lang.OutOfMemoryError: Java heap space

 java.util.concurrent.ExecutionException: java.lang.OutOfMemoryError

     at java.base/java.util.concurrent.ForkJoinTask.get(ForkJoinTask.java:1006)

     at fr.univ_nantes.codex_processor.gui.DatasetExtendedDepthOfFieldProcessor.run(DatasetExtendedDepthOfFieldProcessor.java:69)

     at fr.univ_nantes.codex_processor.gui.MainController$1.handle(MainController.java:96)

     at fr.univ_nantes.codex_processor.gui.MainController$1.handle(MainController.java:64)

     at com.sun.javafx.event.CompositeEventHandler.dispatchBubblingEvent(CompositeEventHandler.java:86)

     at com.sun.javafx.event.EventHandlerManager.dispatchBubblingEvent(EventHandlerManager.java:234)

     at com.sun.javafx.event.EventHandlerManager.dispatchBubblingEvent(EventHandlerManager.java:191)

     at com.sun.javafx.event.CompositeEventDispatcher.dispatchBubblingEvent(CompositeEventDispatcher.java:59)

     at com.sun.javafx.event.BasicEventDispatcher.dispatchEvent(BasicEventDispatcher.java:58)

     at com.sun.javafx.event.EventDispatchChainImpl.dispatchEvent(EventDispatchChainImpl.java:114)

     at com.sun.javafx.event.BasicEventDispatcher.dispatchEvent(BasicEventDispatcher.java:56)

     at com.sun.javafx.event.EventDispatchChainImpl.dispatchEvent(EventDispatchChainImpl.java:114)

     at com.sun.javafx.event.BasicEventDispatcher.dispatchEvent(BasicEventDispatcher.java:56)

     at com.sun.javafx.event.EventDispatchChainImpl.dispatchEvent(EventDispatchChainImpl.java:114)

     at com.sun.javafx.event.EventUtil.fireEventImpl(EventUtil.java:74)

     at com.sun.javafx.event.EventUtil.fireEvent(EventUtil.java:49)

     at javafx.event.Event.fireEvent(Event.java:198)

     at javafx.scene.Node.fireEvent(Node.java:8889)

     at javafx.scene.control.Button.fire(Button.java:203)

     at com.sun.javafx.scene.control.behavior.ButtonBehavior.mouseReleased(ButtonBehavior.java:208)

     at com.sun.javafx.scene.control.inputmap.InputMap.handle(InputMap.java:274)

     at com.sun.javafx.event.CompositeEventHandler$NormalEventHandlerRecord.handleBubblingEvent(CompositeEventHandler.java:247)

     at com.sun.javafx.event.CompositeEventHandler.dispatchBubblingEvent(CompositeEventHandler.java:80)

     at com.sun.javafx.event.EventHandlerManager.dispatchBubblingEvent(EventHandlerManager.java:234)

     at com.sun.javafx.event.EventHandlerManager.dispatchBubblingEvent(EventHandlerManager.java:191)

     at com.sun.javafx.event.CompositeEventDispatcher.dispatchBubblingEvent(CompositeEventDispatcher.java:59)

     at com.sun.javafx.event.BasicEventDispatcher.dispatchEvent(BasicEventDispatcher.java:58)

     at com.sun.javafx.event.EventDispatchChainImpl.dispatchEvent(EventDispatchChainImpl.java:114)

     at com.sun.javafx.event.BasicEventDispatcher.dispatchEvent(BasicEventDispatcher.java:56)

     at com.sun.javafx.event.EventDispatchChainImpl.dispatchEvent(EventDispatchChainImpl.java:114)

     at com.sun.javafx.event.BasicEventDispatcher.dispatchEvent(BasicEventDispatcher.java:56)

     at com.sun.javafx.event.EventDispatchChainImpl.dispatchEvent(EventDispatchChainImpl.java:114)

     at com.sun.javafx.event.EventUtil.fireEventImpl(EventUtil.java:74)

     at com.sun.javafx.event.EventUtil.fireEvent(EventUtil.java:54)

     at javafx.event.Event.fireEvent(Event.java:198)

     at javafx.scene.Scene$MouseHandler.process(Scene.java:3856)

     at javafx.scene.Scene.processMouseEvent(Scene.java:1851)

     at javafx.scene.Scene$ScenePeerListener.mouseEvent(Scene.java:2584)

     at com.sun.javafx.tk.quantum.GlassViewEventHandler$MouseEventNotification.run(GlassViewEventHandler.java:409)

     at com.sun.javafx.tk.quantum.GlassViewEventHandler$MouseEventNotification.run(GlassViewEventHandler.java:299)

     at java.base/java.security.AccessController.doPrivileged(Native Method)

     at com.sun.javafx.tk.quantum.GlassViewEventHandler.lambda$handleMouseEvent$2(GlassViewEventHandler.java:447)

     at com.sun.javafx.tk.quantum.QuantumToolkit.runWithoutRenderLock(QuantumToolkit.java:412)

     at com.sun.javafx.tk.quantum.GlassViewEventHandler.handleMouseEvent(GlassViewEventHandler.java:446)

     at com.sun.glass.ui.View.handleMouseEvent(View.java:556)

     at com.sun.glass.ui.View.notifyMouse(View.java:942)

     at com.sun.glass.ui.win.WinApplication._runLoop(Native Method)

     at com.sun.glass.ui.win.WinApplication.lambda$runLoop$3(WinApplication.java:174)

     at java.base/java.lang.Thread.run(Thread.java:829)

 Caused by: java.lang.OutOfMemoryError

     at java.base/jdk.internal.reflect.GeneratedConstructorAccessor3.newInstance(Unknown Source)

     at java.base/jdk.internal.reflect.DelegatingConstructorAccessorImpl.newInstance(DelegatingConstructorAccessorImpl.java:45)

     at java.base/java.lang.reflect.Constructor.newInstance(Constructor.java:490)

     at java.base/java.util.concurrent.ForkJoinTask.getThrowableException(ForkJoinTask.java:603)

     ... 49 more

 Caused by: java.lang.OutOfMemoryError: Java heap space

 java.util.concurrent.ExecutionException: java.lang.OutOfMemoryError

     at java.base/java.util.concurrent.ForkJoinTask.get(ForkJoinTask.java:1006)

     at fr.univ_nantes.codex_processor.gui.DatasetExtendedDepthOfFieldProcessor.run(DatasetExtendedDepthOfFieldProcessor.java:69)

     at fr.univ_nantes.codex_processor.gui.MainController$1.handle(MainController.java:96)

     at fr.univ_nantes.codex_processor.gui.MainController$1.handle(MainController.java:64)

     at com.sun.javafx.event.CompositeEventHandler.dispatchBubblingEvent(CompositeEventHandler.java:86)

     at com.sun.javafx.event.EventHandlerManager.dispatchBubblingEvent(EventHandlerManager.java:234)

     at com.sun.javafx.event.EventHandlerManager.dispatchBubblingEvent(EventHandlerManager.java:191)

     at com.sun.javafx.event.CompositeEventDispatcher.dispatchBubblingEvent(CompositeEventDispatcher.java:59)

     at com.sun.javafx.event.BasicEventDispatcher.dispatchEvent(BasicEventDispatcher.java:58)

     at com.sun.javafx.event.EventDispatchChainImpl.dispatchEvent(EventDispatchChainImpl.java:114)

     at com.sun.javafx.event.BasicEventDispatcher.dispatchEvent(BasicEventDispatcher.java:56)

     at com.sun.javafx.event.EventDispatchChainImpl.dispatchEvent(EventDispatchChainImpl.java:114)

     at com.sun.javafx.event.BasicEventDispatcher.dispatchEvent(BasicEventDispatcher.java:56)

     at com.sun.javafx.event.EventDispatchChainImpl.dispatchEvent(EventDispatchChainImpl.java:114)

     at com.sun.javafx.event.EventUtil.fireEventImpl(EventUtil.java:74)

     at com.sun.javafx.event.EventUtil.fireEvent(EventUtil.java:49)

     at javafx.event.Event.fireEvent(Event.java:198)

     at javafx.scene.Node.fireEvent(Node.java:8889)

     at javafx.scene.control.Button.fire(Button.java:203)

     at com.sun.javafx.scene.control.behavior.ButtonBehavior.mouseReleased(ButtonBehavior.java:208)

     at com.sun.javafx.scene.control.inputmap.InputMap.handle(InputMap.java:274)

     at com.sun.javafx.event.CompositeEventHandler$NormalEventHandlerRecord.handleBubblingEvent(CompositeEventHandler.java:247)

     at com.sun.javafx.event.CompositeEventHandler.dispatchBubblingEvent(CompositeEventHandler.java:80)

     at com.sun.javafx.event.EventHandlerManager.dispatchBubblingEvent(EventHandlerManager.java:234)

     at com.sun.javafx.event.EventHandlerManager.dispatchBubblingEvent(EventHandlerManager.java:191)

     at com.sun.javafx.event.CompositeEventDispatcher.dispatchBubblingEvent(CompositeEventDispatcher.java:59)

     at com.sun.javafx.event.BasicEventDispatcher.dispatchEvent(BasicEventDispatcher.java:58)

     at com.sun.javafx.event.EventDispatchChainImpl.dispatchEvent(EventDispatchChainImpl.java:114)

     at com.sun.javafx.event.BasicEventDispatcher.dispatchEvent(BasicEventDispatcher.java:56)

     at com.sun.javafx.event.EventDispatchChainImpl.dispatchEvent(EventDispatchChainImpl.java:114)

     at com.sun.javafx.event.BasicEventDispatcher.dispatchEvent(BasicEventDispatcher.java:56)

     at com.sun.javafx.event.EventDispatchChainImpl.dispatchEvent(EventDispatchChainImpl.java:114)

     at com.sun.javafx.event.EventUtil.fireEventImpl(EventUtil.java:74)

     at com.sun.javafx.event.EventUtil.fireEvent(EventUtil.java:54)

     at javafx.event.Event.fireEvent(Event.java:198)

     at javafx.scene.Scene$MouseHandler.process(Scene.java:3856)

     at javafx.scene.Scene.processMouseEvent(Scene.java:1851)

     at javafx.scene.Scene$ScenePeerListener.mouseEvent(Scene.java:2584)

     at com.sun.javafx.tk.quantum.GlassViewEventHandler$MouseEventNotification.run(GlassViewEventHandler.java:409)

     at com.sun.javafx.tk.quantum.GlassViewEventHandler$MouseEventNotification.run(GlassViewEventHandler.java:299)

     at java.base/java.security.AccessController.doPrivileged(Native Method)

     at com.sun.javafx.tk.quantum.GlassViewEventHandler.lambda$handleMouseEvent$2(GlassViewEventHandler.java:447)

     at com.sun.javafx.tk.quantum.QuantumToolkit.runWithoutRenderLock(QuantumToolkit.java:412)

     at com.sun.javafx.tk.quantum.GlassViewEventHandler.handleMouseEvent(GlassViewEventHandler.java:446)

     at com.sun.glass.ui.View.handleMouseEvent(View.java:556)

     at com.sun.glass.ui.View.notifyMouse(View.java:942)

     at com.sun.glass.ui.win.WinApplication._runLoop(Native Method)

     at com.sun.glass.ui.win.WinApplication.lambda$runLoop$3(WinApplication.java:174)

     at java.base/java.lang.Thread.run(Thread.java:829)

 Caused by: java.lang.OutOfMemoryError

     at java.base/jdk.internal.reflect.GeneratedConstructorAccessor3.newInstance(Unknown Source)

     at java.base/jdk.internal.reflect.DelegatingConstructorAccessorImpl.newInstance(DelegatingConstructorAccessorImpl.java:45)

     at java.base/java.lang.reflect.Constructor.newInstance(Constructor.java:490)

     at java.base/java.util.concurrent.ForkJoinTask.getThrowableException(ForkJoinTask.java:603)

     ... 49 more

 Caused by: java.lang.OutOfMemoryError: Java heap space

 java.util.concurrent.ExecutionException: java.lang.OutOfMemoryError

     at java.base/java.util.concurrent.ForkJoinTask.get(ForkJoinTask.java:1006)

     at fr.univ_nantes.codex_processor.gui.DatasetExtendedDepthOfFieldProcessor.run(DatasetExtendedDepthOfFieldProcessor.java:69)

     at fr.univ_nantes.codex_processor.gui.MainController$1.handle(MainController.java:96)

     at fr.univ_nantes.codex_processor.gui.MainController$1.handle(MainController.java:64)

     at com.sun.javafx.event.CompositeEventHandler.dispatchBubblingEvent(CompositeEventHandler.java:86)

     at com.sun.javafx.event.EventHandlerManager.dispatchBubblingEvent(EventHandlerManager.java:234)

     at com.sun.javafx.event.EventHandlerManager.dispatchBubblingEvent(EventHandlerManager.java:191)

     at com.sun.javafx.event.CompositeEventDispatcher.dispatchBubblingEvent(CompositeEventDispatcher.java:59)

     at com.sun.javafx.event.BasicEventDispatcher.dispatchEvent(BasicEventDispatcher.java:58)

     at com.sun.javafx.event.EventDispatchChainImpl.dispatchEvent(EventDispatchChainImpl.java:114)

     at com.sun.javafx.event.BasicEventDispatcher.dispatchEvent(BasicEventDispatcher.java:56)

     at com.sun.javafx.event.EventDispatchChainImpl.dispatchEvent(EventDispatchChainImpl.java:114)

     at com.sun.javafx.event.BasicEventDispatcher.dispatchEvent(BasicEventDispatcher.java:56)

     at com.sun.javafx.event.EventDispatchChainImpl.dispatchEvent(EventDispatchChainImpl.java:114)

     at com.sun.javafx.event.EventUtil.fireEventImpl(EventUtil.java:74)

     at com.sun.javafx.event.EventUtil.fireEvent(EventUtil.java:49)

     at javafx.event.Event.fireEvent(Event.java:198)

     at javafx.scene.Node.fireEvent(Node.java:8889)

     at javafx.scene.control.Button.fire(Button.java:203)

     at com.sun.javafx.scene.control.behavior.ButtonBehavior.mouseReleased(ButtonBehavior.java:208)

     at com.sun.javafx.scene.control.inputmap.InputMap.handle(InputMap.java:274)

     at com.sun.javafx.event.CompositeEventHandler$NormalEventHandlerRecord.handleBubblingEvent(CompositeEventHandler.java:247)

     at com.sun.javafx.event.CompositeEventHandler.dispatchBubblingEvent(CompositeEventHandler.java:80)

     at com.sun.javafx.event.EventHandlerManager.dispatchBubblingEvent(EventHandlerManager.java:234)

     at com.sun.javafx.event.EventHandlerManager.dispatchBubblingEvent(EventHandlerManager.java:191)

     at com.sun.javafx.event.CompositeEventDispatcher.dispatchBubblingEvent(CompositeEventDispatcher.java:59)

     at com.sun.javafx.event.BasicEventDispatcher.dispatchEvent(BasicEventDispatcher.java:58)

     at com.sun.javafx.event.EventDispatchChainImpl.dispatchEvent(EventDispatchChainImpl.java:114)

     at com.sun.javafx.event.BasicEventDispatcher.dispatchEvent(BasicEventDispatcher.java:56)

     at com.sun.javafx.event.EventDispatchChainImpl.dispatchEvent(EventDispatchChainImpl.java:114)

     at com.sun.javafx.event.BasicEventDispatcher.dispatchEvent(BasicEventDispatcher.java:56)

     at com.sun.javafx.event.EventDispatchChainImpl.dispatchEvent(EventDispatchChainImpl.java:114)

     at com.sun.javafx.event.EventUtil.fireEventImpl(EventUtil.java:74)

     at com.sun.javafx.event.EventUtil.fireEvent(EventUtil.java:54)

     at javafx.event.Event.fireEvent(Event.java:198)

     at javafx.scene.Scene$MouseHandler.process(Scene.java:3856)

     at javafx.scene.Scene.processMouseEvent(Scene.java:1851)

     at javafx.scene.Scene$ScenePeerListener.mouseEvent(Scene.java:2584)

     at com.sun.javafx.tk.quantum.GlassViewEventHandler$MouseEventNotification.run(GlassViewEventHandler.java:409)

     at com.sun.javafx.tk.quantum.GlassViewEventHandler$MouseEventNotification.run(GlassViewEventHandler.java:299)

     at java.base/java.security.AccessController.doPrivileged(Native Method)

     at com.sun.javafx.tk.quantum.GlassViewEventHandler.lambda$handleMouseEvent$2(GlassViewEventHandler.java:447)

     at com.sun.javafx.tk.quantum.QuantumToolkit.runWithoutRenderLock(QuantumToolkit.java:412)

     at com.sun.javafx.tk.quantum.GlassViewEventHandler.handleMouseEvent(GlassViewEventHandler.java:446)

     at com.sun.glass.ui.View.handleMouseEvent(View.java:556)

     at com.sun.glass.ui.View.notifyMouse(View.java:942)

     at com.sun.glass.ui.win.WinApplication._runLoop(Native Method)

     at com.sun.glass.ui.win.WinApplication.lambda$runLoop$3(WinApplication.java:174)

     at java.base/java.lang.Thread.run(Thread.java:829)

 Caused by: java.lang.OutOfMemoryError

     at java.base/jdk.internal.reflect.GeneratedConstructorAccessor3.newInstance(Unknown Source)

     at java.base/jdk.internal.reflect.DelegatingConstructorAccessorImpl.newInstance(DelegatingConstructorAccessorImpl.java:45)

     at java.base/java.lang.reflect.Constructor.newInstance(Constructor.java:490)

     at java.base/java.util.concurrent.ForkJoinTask.getThrowableException(ForkJoinTask.java:603)

     ... 49 more

 Caused by: java.lang.OutOfMemoryError: Java heap space

 java.util.concurrent.ExecutionException: java.lang.OutOfMemoryError

     at java.base/java.util.concurrent.ForkJoinTask.get(ForkJoinTask.java:1006)

     at fr.univ_nantes.codex_processor.gui.DatasetExtendedDepthOfFieldProcessor.run(DatasetExtendedDepthOfFieldProcessor.java:69)

     at fr.univ_nantes.codex_processor.gui.MainController$1.handle(MainController.java:96)

     at fr.univ_nantes.codex_processor.gui.MainController$1.handle(MainController.java:64)

     at com.sun.javafx.event.CompositeEventHandler.dispatchBubblingEvent(CompositeEventHandler.java:86)

     at com.sun.javafx.event.EventHandlerManager.dispatchBubblingEvent(EventHandlerManager.java:234)

     at com.sun.javafx.event.EventHandlerManager.dispatchBubblingEvent(EventHandlerManager.java:191)

     at com.sun.javafx.event.CompositeEventDispatcher.dispatchBubblingEvent(CompositeEventDispatcher.java:59)

     at com.sun.javafx.event.BasicEventDispatcher.dispatchEvent(BasicEventDispatcher.java:58)

     at com.sun.javafx.event.EventDispatchChainImpl.dispatchEvent(EventDispatchChainImpl.java:114)

     at com.sun.javafx.event.BasicEventDispatcher.dispatchEvent(BasicEventDispatcher.java:56)

     at com.sun.javafx.event.EventDispatchChainImpl.dispatchEvent(EventDispatchChainImpl.java:114)

     at com.sun.javafx.event.BasicEventDispatcher.dispatchEvent(BasicEventDispatcher.java:56)

     at com.sun.javafx.event.EventDispatchChainImpl.dispatchEvent(EventDispatchChainImpl.java:114)

     at com.sun.javafx.event.EventUtil.fireEventImpl(EventUtil.java:74)

     at com.sun.javafx.event.EventUtil.fireEvent(EventUtil.java:49)

     at javafx.event.Event.fireEvent(Event.java:198)

     at javafx.scene.Node.fireEvent(Node.java:8889)

     at javafx.scene.control.Button.fire(Button.java:203)

     at com.sun.javafx.scene.control.behavior.ButtonBehavior.mouseReleased(ButtonBehavior.java:208)

     at com.sun.javafx.scene.control.inputmap.InputMap.handle(InputMap.java:274)

     at com.sun.javafx.event.CompositeEventHandler$NormalEventHandlerRecord.handleBubblingEvent(CompositeEventHandler.java:247)

     at com.sun.javafx.event.CompositeEventHandler.dispatchBubblingEvent(CompositeEventHandler.java:80)

     at com.sun.javafx.event.EventHandlerManager.dispatchBubblingEvent(EventHandlerManager.java:234)

     at com.sun.javafx.event.EventHandlerManager.dispatchBubblingEvent(EventHandlerManager.java:191)

     at com.sun.javafx.event.CompositeEventDispatcher.dispatchBubblingEvent(CompositeEventDispatcher.java:59)

     at com.sun.javafx.event.BasicEventDispatcher.dispatchEvent(BasicEventDispatcher.java:58)

     at com.sun.javafx.event.EventDispatchChainImpl.dispatchEvent(EventDispatchChainImpl.java:114)

     at com.sun.javafx.event.BasicEventDispatcher.dispatchEvent(BasicEventDispatcher.java:56)

     at com.sun.javafx.event.EventDispatchChainImpl.dispatchEvent(EventDispatchChainImpl.java:114)

     at com.sun.javafx.event.BasicEventDispatcher.dispatchEvent(BasicEventDispatcher.java:56)

     at com.sun.javafx.event.EventDispatchChainImpl.dispatchEvent(EventDispatchChainImpl.java:114)

     at com.sun.javafx.event.EventUtil.fireEventImpl(EventUtil.java:74)

     at com.sun.javafx.event.EventUtil.fireEvent(EventUtil.java:54)

     at javafx.event.Event.fireEvent(Event.java:198)

     at javafx.scene.Scene$MouseHandler.process(Scene.java:3856)

     at javafx.scene.Scene.processMouseEvent(Scene.java:1851)

     at javafx.scene.Scene$ScenePeerListener.mouseEvent(Scene.java:2584)

     at com.sun.javafx.tk.quantum.GlassViewEventHandler$MouseEventNotification.run(GlassViewEventHandler.java:409)

     at com.sun.javafx.tk.quantum.GlassViewEventHandler$MouseEventNotification.run(GlassViewEventHandler.java:299)

     at java.base/java.security.AccessController.doPrivileged(Native Method)

     at com.sun.javafx.tk.quantum.GlassViewEventHandler.lambda$handleMouseEvent$2(GlassViewEventHandler.java:447)

     at com.sun.javafx.tk.quantum.QuantumToolkit.runWithoutRenderLock(QuantumToolkit.java:412)

     at com.sun.javafx.tk.quantum.GlassViewEventHandler.handleMouseEvent(GlassViewEventHandler.java:446)

     at com.sun.glass.ui.View.handleMouseEvent(View.java:556)

     at com.sun.glass.ui.View.notifyMouse(View.java:942)

     at com.sun.glass.ui.win.WinApplication._runLoop(Native Method)

     at com.sun.glass.ui.win.WinApplication.lambda$runLoop$3(WinApplication.java:174)

     at java.base/java.lang.Thread.run(Thread.java:829)

 Caused by: java.lang.OutOfMemoryError

     at java.base/jdk.internal.reflect.GeneratedConstructorAccessor3.newInstance(Unknown Source)

     at java.base/jdk.internal.reflect.DelegatingConstructorAccessorImpl.newInstance(DelegatingConstructorAccessorImpl.java:45)

     at java.base/java.lang.reflect.Constructor.newInstance(Constructor.java:490)

     at java.base/java.util.concurrent.ForkJoinTask.getThrowableException(ForkJoinTask.java:603)

     ... 49 more

 Caused by: java.lang.OutOfMemoryError: Java heap space

 java.util.concurrent.ExecutionException: java.lang.OutOfMemoryError

     at java.base/java.util.concurrent.ForkJoinTask.get(ForkJoinTask.java:1006)

     at fr.univ_nantes.codex_processor.gui.DatasetExtendedDepthOfFieldProcessor.run(DatasetExtendedDepthOfFieldProcessor.java:69)

     at fr.univ_nantes.codex_processor.gui.MainController$1.handle(MainController.java:96)

     at fr.univ_nantes.codex_processor.gui.MainController$1.handle(MainController.java:64)

     at com.sun.javafx.event.CompositeEventHandler.dispatchBubblingEvent(CompositeEventHandler.java:86)

     at com.sun.javafx.event.EventHandlerManager.dispatchBubblingEvent(EventHandlerManager.java:234)

     at com.sun.javafx.event.EventHandlerManager.dispatchBubblingEvent(EventHandlerManager.java:191)

     at com.sun.javafx.event.CompositeEventDispatcher.dispatchBubblingEvent(CompositeEventDispatcher.java:59)

     at com.sun.javafx.event.BasicEventDispatcher.dispatchEvent(BasicEventDispatcher.java:58)

     at com.sun.javafx.event.EventDispatchChainImpl.dispatchEvent(EventDispatchChainImpl.java:114)

     at com.sun.javafx.event.BasicEventDispatcher.dispatchEvent(BasicEventDispatcher.java:56)

     at com.sun.javafx.event.EventDispatchChainImpl.dispatchEvent(EventDispatchChainImpl.java:114)

     at com.sun.javafx.event.BasicEventDispatcher.dispatchEvent(BasicEventDispatcher.java:56)

     at com.sun.javafx.event.EventDispatchChainImpl.dispatchEvent(EventDispatchChainImpl.java:114)

     at com.sun.javafx.event.EventUtil.fireEventImpl(EventUtil.java:74)

     at com.sun.javafx.event.EventUtil.fireEvent(EventUtil.java:49)

     at javafx.event.Event.fireEvent(Event.java:198)

     at javafx.scene.Node.fireEvent(Node.java:8889)

     at javafx.scene.control.Button.fire(Button.java:203)

     at com.sun.javafx.scene.control.behavior.ButtonBehavior.mouseReleased(ButtonBehavior.java:208)

     at com.sun.javafx.scene.control.inputmap.InputMap.handle(InputMap.java:274)

     at com.sun.javafx.event.CompositeEventHandler$NormalEventHandlerRecord.handleBubblingEvent(CompositeEventHandler.java:247)

     at com.sun.javafx.event.CompositeEventHandler.dispatchBubblingEvent(CompositeEventHandler.java:80)

     at com.sun.javafx.event.EventHandlerManager.dispatchBubblingEvent(EventHandlerManager.java:234)

     at com.sun.javafx.event.EventHandlerManager.dispatchBubblingEvent(EventHandlerManager.java:191)

     at com.sun.javafx.event.CompositeEventDispatcher.dispatchBubblingEvent(CompositeEventDispatcher.java:59)

     at com.sun.javafx.event.BasicEventDispatcher.dispatchEvent(BasicEventDispatcher.java:58)

     at com.sun.javafx.event.EventDispatchChainImpl.dispatchEvent(EventDispatchChainImpl.java:114)

     at com.sun.javafx.event.BasicEventDispatcher.dispatchEvent(BasicEventDispatcher.java:56)

     at com.sun.javafx.event.EventDispatchChainImpl.dispatchEvent(EventDispatchChainImpl.java:114)

     at com.sun.javafx.event.BasicEventDispatcher.dispatchEvent(BasicEventDispatcher.java:56)

     at com.sun.javafx.event.EventDispatchChainImpl.dispatchEvent(EventDispatchChainImpl.java:114)

     at com.sun.javafx.event.EventUtil.fireEventImpl(EventUtil.java:74)

     at com.sun.javafx.event.EventUtil.fireEvent(EventUtil.java:54)

     at javafx.event.Event.fireEvent(Event.java:198)

     at javafx.scene.Scene$MouseHandler.process(Scene.java:3856)

     at javafx.scene.Scene.processMouseEvent(Scene.java:1851)

     at javafx.scene.Scene$ScenePeerListener.mouseEvent(Scene.java:2584)

     at com.sun.javafx.tk.quantum.GlassViewEventHandler$MouseEventNotification.run(GlassViewEventHandler.java:409)

     at com.sun.javafx.tk.quantum.GlassViewEventHandler$MouseEventNotification.run(GlassViewEventHandler.java:299)

     at java.base/java.security.AccessController.doPrivileged(Native Method)

     at com.sun.javafx.tk.quantum.GlassViewEventHandler.lambda$handleMouseEvent$2(GlassViewEventHandler.java:447)

     at com.sun.javafx.tk.quantum.QuantumToolkit.runWithoutRenderLock(QuantumToolkit.java:412)

     at com.sun.javafx.tk.quantum.GlassViewEventHandler.handleMouseEvent(GlassViewEventHandler.java:446)

     at com.sun.glass.ui.View.handleMouseEvent(View.java:556)

     at com.sun.glass.ui.View.notifyMouse(View.java:942)

     at com.sun.glass.ui.win.WinApplication._runLoop(Native Method)

     at com.sun.glass.ui.win.WinApplication.lambda$runLoop$3(WinApplication.java:174)

     at java.base/java.lang.Thread.run(Thread.java:829)

 Caused by: java.lang.OutOfMemoryError

     at java.base/jdk.internal.reflect.GeneratedConstructorAccessor3.newInstance(Unknown Source)

     at java.base/jdk.internal.reflect.DelegatingConstructorAccessorImpl.newInstance(DelegatingConstructorAccessorImpl.java:45)

     at java.base/java.lang.reflect.Constructor.newInstance(Constructor.java:490)

     at java.base/java.util.concurrent.ForkJoinTask.getThrowableException(ForkJoinTask.java:603)

     ... 49 more

 Caused by: java.lang.OutOfMemoryError: Java heap space

 java.util.concurrent.ExecutionException: java.lang.OutOfMemoryError

     at java.base/java.util.concurrent.ForkJoinTask.get(ForkJoinTask.java:1006)

     at fr.univ_nantes.codex_processor.gui.DatasetExtendedDepthOfFieldProcessor.run(DatasetExtendedDepthOfFieldProcessor.java:69)

     at fr.univ_nantes.codex_processor.gui.MainController$1.handle(MainController.java:96)

     at fr.univ_nantes.codex_processor.gui.MainController$1.handle(MainController.java:64)

     at com.sun.javafx.event.CompositeEventHandler.dispatchBubblingEvent(CompositeEventHandler.java:86)

     at com.sun.javafx.event.EventHandlerManager.dispatchBubblingEvent(EventHandlerManager.java:234)

     at com.sun.javafx.event.EventHandlerManager.dispatchBubblingEvent(EventHandlerManager.java:191)

     at com.sun.javafx.event.CompositeEventDispatcher.dispatchBubblingEvent(CompositeEventDispatcher.java:59)

     at com.sun.javafx.event.BasicEventDispatcher.dispatchEvent(BasicEventDispatcher.java:58)

     at com.sun.javafx.event.EventDispatchChainImpl.dispatchEvent(EventDispatchChainImpl.java:114)

     at com.sun.javafx.event.BasicEventDispatcher.dispatchEvent(BasicEventDispatcher.java:56)

     at com.sun.javafx.event.EventDispatchChainImpl.dispatchEvent(EventDispatchChainImpl.java:114)

     at com.sun.javafx.event.BasicEventDispatcher.dispatchEvent(BasicEventDispatcher.java:56)

     at com.sun.javafx.event.EventDispatchChainImpl.dispatchEvent(EventDispatchChainImpl.java:114)

     at com.sun.javafx.event.EventUtil.fireEventImpl(EventUtil.java:74)

     at com.sun.javafx.event.EventUtil.fireEvent(EventUtil.java:49)

     at javafx.event.Event.fireEvent(Event.java:198)

     at javafx.scene.Node.fireEvent(Node.java:8889)

     at javafx.scene.control.Button.fire(Button.java:203)

     at com.sun.javafx.scene.control.behavior.ButtonBehavior.mouseReleased(ButtonBehavior.java:208)

     at com.sun.javafx.scene.control.inputmap.InputMap.handle(InputMap.java:274)

     at com.sun.javafx.event.CompositeEventHandler$NormalEventHandlerRecord.handleBubblingEvent(CompositeEventHandler.java:247)

     at com.sun.javafx.event.CompositeEventHandler.dispatchBubblingEvent(CompositeEventHandler.java:80)

     at com.sun.javafx.event.EventHandlerManager.dispatchBubblingEvent(EventHandlerManager.java:234)

     at com.sun.javafx.event.EventHandlerManager.dispatchBubblingEvent(EventHandlerManager.java:191)

     at com.sun.javafx.event.CompositeEventDispatcher.dispatchBubblingEvent(CompositeEventDispatcher.java:59)

     at com.sun.javafx.event.BasicEventDispatcher.dispatchEvent(BasicEventDispatcher.java:58)

     at com.sun.javafx.event.EventDispatchChainImpl.dispatchEvent(EventDispatchChainImpl.java:114)

     at com.sun.javafx.event.BasicEventDispatcher.dispatchEvent(BasicEventDispatcher.java:56)

     at com.sun.javafx.event.EventDispatchChainImpl.dispatchEvent(EventDispatchChainImpl.java:114)

     at com.sun.javafx.event.BasicEventDispatcher.dispatchEvent(BasicEventDispatcher.java:56)

     at com.sun.javafx.event.EventDispatchChainImpl.dispatchEvent(EventDispatchChainImpl.java:114)

     at com.sun.javafx.event.EventUtil.fireEventImpl(EventUtil.java:74)

     at com.sun.javafx.event.EventUtil.fireEvent(EventUtil.java:54)

     at javafx.event.Event.fireEvent(Event.java:198)

     at javafx.scene.Scene$MouseHandler.process(Scene.java:3856)

     at javafx.scene.Scene.processMouseEvent(Scene.java:1851)

     at javafx.scene.Scene$ScenePeerListener.mouseEvent(Scene.java:2584)

     at com.sun.javafx.tk.quantum.GlassViewEventHandler$MouseEventNotification.run(GlassViewEventHandler.java:409)

     at com.sun.javafx.tk.quantum.GlassViewEventHandler$MouseEventNotification.run(GlassViewEventHandler.java:299)

     at java.base/java.security.AccessController.doPrivileged(Native Method)

     at com.sun.javafx.tk.quantum.GlassViewEventHandler.lambda$handleMouseEvent$2(GlassViewEventHandler.java:447)

     at com.sun.javafx.tk.quantum.QuantumToolkit.runWithoutRenderLock(QuantumToolkit.java:412)

     at com.sun.javafx.tk.quantum.GlassViewEventHandler.handleMouseEvent(GlassViewEventHandler.java:446)

     at com.sun.glass.ui.View.handleMouseEvent(View.java:556)

     at com.sun.glass.ui.View.notifyMouse(View.java:942)

     at com.sun.glass.ui.win.WinApplication._runLoop(Native Method)

     at com.sun.glass.ui.win.WinApplication.lambda$runLoop$3(WinApplication.java:174)

     at java.base/java.lang.Thread.run(Thread.java:829)

 Caused by: java.lang.OutOfMemoryError

     at java.base/jdk.internal.reflect.GeneratedConstructorAccessor3.newInstance(Unknown Source)

     at java.base/jdk.internal.reflect.DelegatingConstructorAccessorImpl.newInstance(DelegatingConstructorAccessorImpl.java:45)

     at java.base/java.lang.reflect.Constructor.newInstance(Constructor.java:490)

     at java.base/java.util.concurrent.ForkJoinTask.getThrowableException(ForkJoinTask.java:603)

     ... 49 more

 Caused by: java.lang.OutOfMemoryError: Java heap space

 java.util.concurrent.ExecutionException: java.lang.OutOfMemoryError

     at java.base/java.util.concurrent.ForkJoinTask.get(ForkJoinTask.java:1006)

     at fr.univ_nantes.codex_processor.gui.DatasetExtendedDepthOfFieldProcessor.run(DatasetExtendedDepthOfFieldProcessor.java:69)

     at fr.univ_nantes.codex_processor.gui.MainController$1.handle(MainController.java:96)

     at fr.univ_nantes.codex_processor.gui.MainController$1.handle(MainController.java:64)

     at com.sun.javafx.event.CompositeEventHandler.dispatchBubblingEvent(CompositeEventHandler.java:86)

     at com.sun.javafx.event.EventHandlerManager.dispatchBubblingEvent(EventHandlerManager.java:234)

     at com.sun.javafx.event.EventHandlerManager.dispatchBubblingEvent(EventHandlerManager.java:191)

     at com.sun.javafx.event.CompositeEventDispatcher.dispatchBubblingEvent(CompositeEventDispatcher.java:59)

     at com.sun.javafx.event.BasicEventDispatcher.dispatchEvent(BasicEventDispatcher.java:58)

     at com.sun.javafx.event.EventDispatchChainImpl.dispatchEvent(EventDispatchChainImpl.java:114)

     at com.sun.javafx.event.BasicEventDispatcher.dispatchEvent(BasicEventDispatcher.java:56)

     at com.sun.javafx.event.EventDispatchChainImpl.dispatchEvent(EventDispatchChainImpl.java:114)

     at com.sun.javafx.event.BasicEventDispatcher.dispatchEvent(BasicEventDispatcher.java:56)

     at com.sun.javafx.event.EventDispatchChainImpl.dispatchEvent(EventDispatchChainImpl.java:114)

     at com.sun.javafx.event.EventUtil.fireEventImpl(EventUtil.java:74)

     at com.sun.javafx.event.EventUtil.fireEvent(EventUtil.java:49)

     at javafx.event.Event.fireEvent(Event.java:198)

     at javafx.scene.Node.fireEvent(Node.java:8889)

     at javafx.scene.control.Button.fire(Button.java:203)

     at com.sun.javafx.scene.control.behavior.ButtonBehavior.mouseReleased(ButtonBehavior.java:208)

     at com.sun.javafx.scene.control.inputmap.InputMap.handle(InputMap.java:274)

     at com.sun.javafx.event.CompositeEventHandler$NormalEventHandlerRecord.handleBubblingEvent(CompositeEventHandler.java:247)

     at com.sun.javafx.event.CompositeEventHandler.dispatchBubblingEvent(CompositeEventHandler.java:80)

     at com.sun.javafx.event.EventHandlerManager.dispatchBubblingEvent(EventHandlerManager.java:234)

     at com.sun.javafx.event.EventHandlerManager.dispatchBubblingEvent(EventHandlerManager.java:191)

     at com.sun.javafx.event.CompositeEventDispatcher.dispatchBubblingEvent(CompositeEventDispatcher.java:59)

     at com.sun.javafx.event.BasicEventDispatcher.dispatchEvent(BasicEventDispatcher.java:58)

     at com.sun.javafx.event.EventDispatchChainImpl.dispatchEvent(EventDispatchChainImpl.java:114)

     at com.sun.javafx.event.BasicEventDispatcher.dispatchEvent(BasicEventDispatcher.java:56)

     at com.sun.javafx.event.EventDispatchChainImpl.dispatchEvent(EventDispatchChainImpl.java:114)

     at com.sun.javafx.event.BasicEventDispatcher.dispatchEvent(BasicEventDispatcher.java:56)

     at com.sun.javafx.event.EventDispatchChainImpl.dispatchEvent(EventDispatchChainImpl.java:114)

     at com.sun.javafx.event.EventUtil.fireEventImpl(EventUtil.java:74)

     at com.sun.javafx.event.EventUtil.fireEvent(EventUtil.java:54)

     at javafx.event.Event.fireEvent(Event.java:198)

     at javafx.scene.Scene$MouseHandler.process(Scene.java:3856)

     at javafx.scene.Scene.processMouseEvent(Scene.java:1851)

     at javafx.scene.Scene$ScenePeerListener.mouseEvent(Scene.java:2584)

     at com.sun.javafx.tk.quantum.GlassViewEventHandler$MouseEventNotification.run(GlassViewEventHandler.java:409)

     at com.sun.javafx.tk.quantum.GlassViewEventHandler$MouseEventNotification.run(GlassViewEventHandler.java:299)

     at java.base/java.security.AccessController.doPrivileged(Native Method)

     at com.sun.javafx.tk.quantum.GlassViewEventHandler.lambda$handleMouseEvent$2(GlassViewEventHandler.java:447)

     at com.sun.javafx.tk.quantum.QuantumToolkit.runWithoutRenderLock(QuantumToolkit.java:412)

     at com.sun.javafx.tk.quantum.GlassViewEventHandler.handleMouseEvent(GlassViewEventHandler.java:446)

     at com.sun.glass.ui.View.handleMouseEvent(View.java:556)

     at com.sun.glass.ui.View.notifyMouse(View.java:942)

     at com.sun.glass.ui.win.WinApplication._runLoop(Native Method)

     at com.sun.glass.ui.win.WinApplication.lambda$runLoop$3(WinApplication.java:174)

     at java.base/java.lang.Thread.run(Thread.java:829)

 Caused by: java.lang.OutOfMemoryError

     at java.base/jdk.internal.reflect.GeneratedConstructorAccessor3.newInstance(Unknown Source)

     at java.base/jdk.internal.reflect.DelegatingConstructorAccessorImpl.newInstance(DelegatingConstructorAccessorImpl.java:45)

     at java.base/java.lang.reflect.Constructor.newInstance(Constructor.java:490)

     at java.base/java.util.concurrent.ForkJoinTask.getThrowableException(ForkJoinTask.java:603)

     ... 49 more

 Caused by: java.lang.OutOfMemoryError: Java heap space

 java.util.concurrent.ExecutionException: java.lang.OutOfMemoryError

     at java.base/java.util.concurrent.ForkJoinTask.get(ForkJoinTask.java:1006)

     at fr.univ_nantes.codex_processor.gui.DatasetExtendedDepthOfFieldProcessor.run(DatasetExtendedDepthOfFieldProcessor.java:69)

     at fr.univ_nantes.codex_processor.gui.MainController$1.handle(MainController.java:96)

     at fr.univ_nantes.codex_processor.gui.MainController$1.handle(MainController.java:64)

     at com.sun.javafx.event.CompositeEventHandler.dispatchBubblingEvent(CompositeEventHandler.java:86)

     at com.sun.javafx.event.EventHandlerManager.dispatchBubblingEvent(EventHandlerManager.java:234)

     at com.sun.javafx.event.EventHandlerManager.dispatchBubblingEvent(EventHandlerManager.java:191)

     at com.sun.javafx.event.CompositeEventDispatcher.dispatchBubblingEvent(CompositeEventDispatcher.java:59)

     at com.sun.javafx.event.BasicEventDispatcher.dispatchEvent(BasicEventDispatcher.java:58)

     at com.sun.javafx.event.EventDispatchChainImpl.dispatchEvent(EventDispatchChainImpl.java:114)

     at com.sun.javafx.event.BasicEventDispatcher.dispatchEvent(BasicEventDispatcher.java:56)

     at com.sun.javafx.event.EventDispatchChainImpl.dispatchEvent(EventDispatchChainImpl.java:114)

     at com.sun.javafx.event.BasicEventDispatcher.dispatchEvent(BasicEventDispatcher.java:56)

     at com.sun.javafx.event.EventDispatchChainImpl.dispatchEvent(EventDispatchChainImpl.java:114)

     at com.sun.javafx.event.EventUtil.fireEventImpl(EventUtil.java:74)

     at com.sun.javafx.event.EventUtil.fireEvent(EventUtil.java:49)

     at javafx.event.Event.fireEvent(Event.java:198)

     at javafx.scene.Node.fireEvent(Node.java:8889)

     at javafx.scene.control.Button.fire(Button.java:203)

     at com.sun.javafx.scene.control.behavior.ButtonBehavior.mouseReleased(ButtonBehavior.java:208)

     at com.sun.javafx.scene.control.inputmap.InputMap.handle(InputMap.java:274)

     at com.sun.javafx.event.CompositeEventHandler$NormalEventHandlerRecord.handleBubblingEvent(CompositeEventHandler.java:247)

     at com.sun.javafx.event.CompositeEventHandler.dispatchBubblingEvent(CompositeEventHandler.java:80)

     at com.sun.javafx.event.EventHandlerManager.dispatchBubblingEvent(EventHandlerManager.java:234)

     at com.sun.javafx.event.EventHandlerManager.dispatchBubblingEvent(EventHandlerManager.java:191)

     at com.sun.javafx.event.CompositeEventDispatcher.dispatchBubblingEvent(CompositeEventDispatcher.java:59)

     at com.sun.javafx.event.BasicEventDispatcher.dispatchEvent(BasicEventDispatcher.java:58)

     at com.sun.javafx.event.EventDispatchChainImpl.dispatchEvent(EventDispatchChainImpl.java:114)

     at com.sun.javafx.event.BasicEventDispatcher.dispatchEvent(BasicEventDispatcher.java:56)

     at com.sun.javafx.event.EventDispatchChainImpl.dispatchEvent(EventDispatchChainImpl.java:114)

     at com.sun.javafx.event.BasicEventDispatcher.dispatchEvent(BasicEventDispatcher.java:56)

     at com.sun.javafx.event.EventDispatchChainImpl.dispatchEvent(EventDispatchChainImpl.java:114)

     at com.sun.javafx.event.EventUtil.fireEventImpl(EventUtil.java:74)

     at com.sun.javafx.event.EventUtil.fireEvent(EventUtil.java:54)

     at javafx.event.Event.fireEvent(Event.java:198)

     at javafx.scene.Scene$MouseHandler.process(Scene.java:3856)

     at javafx.scene.Scene.processMouseEvent(Scene.java:1851)

     at javafx.scene.Scene$ScenePeerListener.mouseEvent(Scene.java:2584)

     at com.sun.javafx.tk.quantum.GlassViewEventHandler$MouseEventNotification.run(GlassViewEventHandler.java:409)

     at com.sun.javafx.tk.quantum.GlassViewEventHandler$MouseEventNotification.run(GlassViewEventHandler.java:299)

     at java.base/java.security.AccessController.doPrivileged(Native Method)

     at com.sun.javafx.tk.quantum.GlassViewEventHandler.lambda$handleMouseEvent$2(GlassViewEventHandler.java:447)

     at com.sun.javafx.tk.quantum.QuantumToolkit.runWithoutRenderLock(QuantumToolkit.java:412)

     at com.sun.javafx.tk.quantum.GlassViewEventHandler.handleMouseEvent(GlassViewEventHandler.java:446)

     at com.sun.glass.ui.View.handleMouseEvent(View.java:556)

     at com.sun.glass.ui.View.notifyMouse(View.java:942)

     at com.sun.glass.ui.win.WinApplication._runLoop(Native Method)

     at com.sun.glass.ui.win.WinApplication.lambda$runLoop$3(WinApplication.java:174)

     at java.base/java.lang.Thread.run(Thread.java:829)

 Caused by: java.lang.OutOfMemoryError

     at java.base/jdk.internal.reflect.GeneratedConstructorAccessor3.newInstance(Unknown Source)

     at java.base/jdk.internal.reflect.DelegatingConstructorAccessorImpl.newInstance(DelegatingConstructorAccessorImpl.java:45)

     at java.base/java.lang.reflect.Constructor.newInstance(Constructor.java:490)

     at java.base/java.util.concurrent.ForkJoinTask.getThrowableException(ForkJoinTask.java:603)

     ... 49 more

 Caused by: java.lang.OutOfMemoryError: Java heap space

 java.util.concurrent.ExecutionException: java.lang.OutOfMemoryError

     at java.base/java.util.concurrent.ForkJoinTask.get(ForkJoinTask.java:1006)

     at fr.univ_nantes.codex_processor.gui.DatasetExtendedDepthOfFieldProcessor.run(DatasetExtendedDepthOfFieldProcessor.java:69)

     at fr.univ_nantes.codex_processor.gui.MainController$1.handle(MainController.java:96)

     at fr.univ_nantes.codex_processor.gui.MainController$1.handle(MainController.java:64)

     at com.sun.javafx.event.CompositeEventHandler.dispatchBubblingEvent(CompositeEventHandler.java:86)

     at com.sun.javafx.event.EventHandlerManager.dispatchBubblingEvent(EventHandlerManager.java:234)

     at com.sun.javafx.event.EventHandlerManager.dispatchBubblingEvent(EventHandlerManager.java:191)

     at com.sun.javafx.event.CompositeEventDispatcher.dispatchBubblingEvent(CompositeEventDispatcher.java:59)

     at com.sun.javafx.event.BasicEventDispatcher.dispatchEvent(BasicEventDispatcher.java:58)

     at com.sun.javafx.event.EventDispatchChainImpl.dispatchEvent(EventDispatchChainImpl.java:114)

     at com.sun.javafx.event.BasicEventDispatcher.dispatchEvent(BasicEventDispatcher.java:56)

     at com.sun.javafx.event.EventDispatchChainImpl.dispatchEvent(EventDispatchChainImpl.java:114)

     at com.sun.javafx.event.BasicEventDispatcher.dispatchEvent(BasicEventDispatcher.java:56)

     at com.sun.javafx.event.EventDispatchChainImpl.dispatchEvent(EventDispatchChainImpl.java:114)

     at com.sun.javafx.event.EventUtil.fireEventImpl(EventUtil.java:74)

     at com.sun.javafx.event.EventUtil.fireEvent(EventUtil.java:49)

     at javafx.event.Event.fireEvent(Event.java:198)

     at javafx.scene.Node.fireEvent(Node.java:8889)

     at javafx.scene.control.Button.fire(Button.java:203)

     at com.sun.javafx.scene.control.behavior.ButtonBehavior.mouseReleased(ButtonBehavior.java:208)

     at com.sun.javafx.scene.control.inputmap.InputMap.handle(InputMap.java:274)

     at com.sun.javafx.event.CompositeEventHandler$NormalEventHandlerRecord.handleBubblingEvent(CompositeEventHandler.java:247)

     at com.sun.javafx.event.CompositeEventHandler.dispatchBubblingEvent(CompositeEventHandler.java:80)

     at com.sun.javafx.event.EventHandlerManager.dispatchBubblingEvent(EventHandlerManager.java:234)

     at com.sun.javafx.event.EventHandlerManager.dispatchBubblingEvent(EventHandlerManager.java:191)

     at com.sun.javafx.event.CompositeEventDispatcher.dispatchBubblingEvent(CompositeEventDispatcher.java:59)

     at com.sun.javafx.event.BasicEventDispatcher.dispatchEvent(BasicEventDispatcher.java:58)

     at com.sun.javafx.event.EventDispatchChainImpl.dispatchEvent(EventDispatchChainImpl.java:114)

     at com.sun.javafx.event.BasicEventDispatcher.dispatchEvent(BasicEventDispatcher.java:56)

     at com.sun.javafx.event.EventDispatchChainImpl.dispatchEvent(EventDispatchChainImpl.java:114)

     at com.sun.javafx.event.BasicEventDispatcher.dispatchEvent(BasicEventDispatcher.java:56)

     at com.sun.javafx.event.EventDispatchChainImpl.dispatchEvent(EventDispatchChainImpl.java:114)

     at com.sun.javafx.event.EventUtil.fireEventImpl(EventUtil.java:74)

     at com.sun.javafx.event.EventUtil.fireEvent(EventUtil.java:54)

     at javafx.event.Event.fireEvent(Event.java:198)

     at javafx.scene.Scene$MouseHandler.process(Scene.java:3856)

     at javafx.scene.Scene.processMouseEvent(Scene.java:1851)

     at javafx.scene.Scene$ScenePeerListener.mouseEvent(Scene.java:2584)

     at com.sun.javafx.tk.quantum.GlassViewEventHandler$MouseEventNotification.run(GlassViewEventHandler.java:409)

     at com.sun.javafx.tk.quantum.GlassViewEventHandler$MouseEventNotification.run(GlassViewEventHandler.java:299)

     at java.base/java.security.AccessController.doPrivileged(Native Method)

     at com.sun.javafx.tk.quantum.GlassViewEventHandler.lambda$handleMouseEvent$2(GlassViewEventHandler.java:447)

     at com.sun.javafx.tk.quantum.QuantumToolkit.runWithoutRenderLock(QuantumToolkit.java:412)

     at com.sun.javafx.tk.quantum.GlassViewEventHandler.handleMouseEvent(GlassViewEventHandler.java:446)

     at com.sun.glass.ui.View.handleMouseEvent(View.java:556)

     at com.sun.glass.ui.View.notifyMouse(View.java:942)

     at com.sun.glass.ui.win.WinApplication._runLoop(Native Method)

     at com.sun.glass.ui.win.WinApplication.lambda$runLoop$3(WinApplication.java:174)

     at java.base/java.lang.Thread.run(Thread.java:829)

 Caused by: java.lang.OutOfMemoryError

     at java.base/jdk.internal.reflect.GeneratedConstructorAccessor3.newInstance(Unknown Source)

     at java.base/jdk.internal.reflect.DelegatingConstructorAccessorImpl.newInstance(DelegatingConstructorAccessorImpl.java:45)

     at java.base/java.lang.reflect.Constructor.newInstance(Constructor.java:490)

     at java.base/java.util.concurrent.ForkJoinTask.getThrowableException(ForkJoinTask.java:603)

     ... 49 more

 Caused by: java.lang.OutOfMemoryError: Java heap space

 java.util.concurrent.ExecutionException: java.lang.OutOfMemoryError

     at java.base/java.util.concurrent.ForkJoinTask.get(ForkJoinTask.java:1006)

     at fr.univ_nantes.codex_processor.gui.DatasetExtendedDepthOfFieldProcessor.run(DatasetExtendedDepthOfFieldProcessor.java:69)

     at fr.univ_nantes.codex_processor.gui.MainController$1.handle(MainController.java:96)

     at fr.univ_nantes.codex_processor.gui.MainController$1.handle(MainController.java:64)

     at com.sun.javafx.event.CompositeEventHandler.dispatchBubblingEvent(CompositeEventHandler.java:86)

     at com.sun.javafx.event.EventHandlerManager.dispatchBubblingEvent(EventHandlerManager.java:234)

     at com.sun.javafx.event.EventHandlerManager.dispatchBubblingEvent(EventHandlerManager.java:191)

     at com.sun.javafx.event.CompositeEventDispatcher.dispatchBubblingEvent(CompositeEventDispatcher.java:59)

     at com.sun.javafx.event.BasicEventDispatcher.dispatchEvent(BasicEventDispatcher.java:58)

     at com.sun.javafx.event.EventDispatchChainImpl.dispatchEvent(EventDispatchChainImpl.java:114)

     at com.sun.javafx.event.BasicEventDispatcher.dispatchEvent(BasicEventDispatcher.java:56)

     at com.sun.javafx.event.EventDispatchChainImpl.dispatchEvent(EventDispatchChainImpl.java:114)

     at com.sun.javafx.event.BasicEventDispatcher.dispatchEvent(BasicEventDispatcher.java:56)

     at com.sun.javafx.event.EventDispatchChainImpl.dispatchEvent(EventDispatchChainImpl.java:114)

     at com.sun.javafx.event.EventUtil.fireEventImpl(EventUtil.java:74)

     at com.sun.javafx.event.EventUtil.fireEvent(EventUtil.java:49)

     at javafx.event.Event.fireEvent(Event.java:198)

     at javafx.scene.Node.fireEvent(Node.java:8889)

     at javafx.scene.control.Button.fire(Button.java:203)

     at com.sun.javafx.scene.control.behavior.ButtonBehavior.mouseReleased(ButtonBehavior.java:208)

     at com.sun.javafx.scene.control.inputmap.InputMap.handle(InputMap.java:274)

     at com.sun.javafx.event.CompositeEventHandler$NormalEventHandlerRecord.handleBubblingEvent(CompositeEventHandler.java:247)

     at com.sun.javafx.event.CompositeEventHandler.dispatchBubblingEvent(CompositeEventHandler.java:80)

     at com.sun.javafx.event.EventHandlerManager.dispatchBubblingEvent(EventHandlerManager.java:234)

     at com.sun.javafx.event.EventHandlerManager.dispatchBubblingEvent(EventHandlerManager.java:191)

     at com.sun.javafx.event.CompositeEventDispatcher.dispatchBubblingEvent(CompositeEventDispatcher.java:59)

     at com.sun.javafx.event.BasicEventDispatcher.dispatchEvent(BasicEventDispatcher.java:58)

     at com.sun.javafx.event.EventDispatchChainImpl.dispatchEvent(EventDispatchChainImpl.java:114)

     at com.sun.javafx.event.BasicEventDispatcher.dispatchEvent(BasicEventDispatcher.java:56)

     at com.sun.javafx.event.EventDispatchChainImpl.dispatchEvent(EventDispatchChainImpl.java:114)

     at com.sun.javafx.event.BasicEventDispatcher.dispatchEvent(BasicEventDispatcher.java:56)

     at com.sun.javafx.event.EventDispatchChainImpl.dispatchEvent(EventDispatchChainImpl.java:114)

     at com.sun.javafx.event.EventUtil.fireEventImpl(EventUtil.java:74)

     at com.sun.javafx.event.EventUtil.fireEvent(EventUtil.java:54)

     at javafx.event.Event.fireEvent(Event.java:198)

     at javafx.scene.Scene$MouseHandler.process(Scene.java:3856)

     at javafx.scene.Scene.processMouseEvent(Scene.java:1851)

     at javafx.scene.Scene$ScenePeerListener.mouseEvent(Scene.java:2584)

     at com.sun.javafx.tk.quantum.GlassViewEventHandler$MouseEventNotification.run(GlassViewEventHandler.java:409)

     at com.sun.javafx.tk.quantum.GlassViewEventHandler$MouseEventNotification.run(GlassViewEventHandler.java:299)

     at java.base/java.security.AccessController.doPrivileged(Native Method)

     at com.sun.javafx.tk.quantum.GlassViewEventHandler.lambda$handleMouseEvent$2(GlassViewEventHandler.java:447)

     at com.sun.javafx.tk.quantum.QuantumToolkit.runWithoutRenderLock(QuantumToolkit.java:412)

     at com.sun.javafx.tk.quantum.GlassViewEventHandler.handleMouseEvent(GlassViewEventHandler.java:446)

     at com.sun.glass.ui.View.handleMouseEvent(View.java:556)

     at com.sun.glass.ui.View.notifyMouse(View.java:942)

     at com.sun.glass.ui.win.WinApplication._runLoop(Native Method)

     at com.sun.glass.ui.win.WinApplication.lambda$runLoop$3(WinApplication.java:174)

     at java.base/java.lang.Thread.run(Thread.java:829)

 Caused by: java.lang.OutOfMemoryError

     at java.base/jdk.internal.reflect.GeneratedConstructorAccessor3.newInstance(Unknown Source)

     at java.base/jdk.internal.reflect.DelegatingConstructorAccessorImpl.newInstance(DelegatingConstructorAccessorImpl.java:45)

     at java.base/java.lang.reflect.Constructor.newInstance(Constructor.java:490)

     at java.base/java.util.concurrent.ForkJoinTask.getThrowableException(ForkJoinTask.java:603)

     ... 49 more

 Caused by: java.lang.OutOfMemoryError: Java heap space

 java.util.concurrent.ExecutionException: java.lang.OutOfMemoryError

     at java.base/java.util.concurrent.ForkJoinTask.get(ForkJoinTask.java:1006)

     at fr.univ_nantes.codex_processor.gui.DatasetExtendedDepthOfFieldProcessor.run(DatasetExtendedDepthOfFieldProcessor.java:69)

     at fr.univ_nantes.codex_processor.gui.MainController$1.handle(MainController.java:96)

     at fr.univ_nantes.codex_processor.gui.MainController$1.handle(MainController.java:64)

     at com.sun.javafx.event.CompositeEventHandler.dispatchBubblingEvent(CompositeEventHandler.java:86)

     at com.sun.javafx.event.EventHandlerManager.dispatchBubblingEvent(EventHandlerManager.java:234)

     at com.sun.javafx.event.EventHandlerManager.dispatchBubblingEvent(EventHandlerManager.java:191)

     at com.sun.javafx.event.CompositeEventDispatcher.dispatchBubblingEvent(CompositeEventDispatcher.java:59)

     at com.sun.javafx.event.BasicEventDispatcher.dispatchEvent(BasicEventDispatcher.java:58)

     at com.sun.javafx.event.EventDispatchChainImpl.dispatchEvent(EventDispatchChainImpl.java:114)

     at com.sun.javafx.event.BasicEventDispatcher.dispatchEvent(BasicEventDispatcher.java:56)

     at com.sun.javafx.event.EventDispatchChainImpl.dispatchEvent(EventDispatchChainImpl.java:114)

     at com.sun.javafx.event.BasicEventDispatcher.dispatchEvent(BasicEventDispatcher.java:56)

     at com.sun.javafx.event.EventDispatchChainImpl.dispatchEvent(EventDispatchChainImpl.java:114)

     at com.sun.javafx.event.EventUtil.fireEventImpl(EventUtil.java:74)

     at com.sun.javafx.event.EventUtil.fireEvent(EventUtil.java:49)

     at javafx.event.Event.fireEvent(Event.java:198)

     at javafx.scene.Node.fireEvent(Node.java:8889)

     at javafx.scene.control.Button.fire(Button.java:203)

     at com.sun.javafx.scene.control.behavior.ButtonBehavior.mouseReleased(ButtonBehavior.java:208)

     at com.sun.javafx.scene.control.inputmap.InputMap.handle(InputMap.java:274)

     at com.sun.javafx.event.CompositeEventHandler$NormalEventHandlerRecord.handleBubblingEvent(CompositeEventHandler.java:247)

     at com.sun.javafx.event.CompositeEventHandler.dispatchBubblingEvent(CompositeEventHandler.java:80)

     at com.sun.javafx.event.EventHandlerManager.dispatchBubblingEvent(EventHandlerManager.java:234)

     at com.sun.javafx.event.EventHandlerManager.dispatchBubblingEvent(EventHandlerManager.java:191)

     at com.sun.javafx.event.CompositeEventDispatcher.dispatchBubblingEvent(CompositeEventDispatcher.java:59)

     at com.sun.javafx.event.BasicEventDispatcher.dispatchEvent(BasicEventDispatcher.java:58)

     at com.sun.javafx.event.EventDispatchChainImpl.dispatchEvent(EventDispatchChainImpl.java:114)

     at com.sun.javafx.event.BasicEventDispatcher.dispatchEvent(BasicEventDispatcher.java:56)

     at com.sun.javafx.event.EventDispatchChainImpl.dispatchEvent(EventDispatchChainImpl.java:114)

     at com.sun.javafx.event.BasicEventDispatcher.dispatchEvent(BasicEventDispatcher.java:56)

     at com.sun.javafx.event.EventDispatchChainImpl.dispatchEvent(EventDispatchChainImpl.java:114)

     at com.sun.javafx.event.EventUtil.fireEventImpl(EventUtil.java:74)

     at com.sun.javafx.event.EventUtil.fireEvent(EventUtil.java:54)

     at javafx.event.Event.fireEvent(Event.java:198)

     at javafx.scene.Scene$MouseHandler.process(Scene.java:3856)

     at javafx.scene.Scene.processMouseEvent(Scene.java:1851)

     at javafx.scene.Scene$ScenePeerListener.mouseEvent(Scene.java:2584)

     at com.sun.javafx.tk.quantum.GlassViewEventHandler$MouseEventNotification.run(GlassViewEventHandler.java:409)

     at com.sun.javafx.tk.quantum.GlassViewEventHandler$MouseEventNotification.run(GlassViewEventHandler.java:299)

     at java.base/java.security.AccessController.doPrivileged(Native Method)

     at com.sun.javafx.tk.quantum.GlassViewEventHandler.lambda$handleMouseEvent$2(GlassViewEventHandler.java:447)

     at com.sun.javafx.tk.quantum.QuantumToolkit.runWithoutRenderLock(QuantumToolkit.java:412)

     at com.sun.javafx.tk.quantum.GlassViewEventHandler.handleMouseEvent(GlassViewEventHandler.java:446)

     at com.sun.glass.ui.View.handleMouseEvent(View.java:556)

     at com.sun.glass.ui.View.notifyMouse(View.java:942)

     at com.sun.glass.ui.win.WinApplication._runLoop(Native Method)

     at com.sun.glass.ui.win.WinApplication.lambda$runLoop$3(WinApplication.java:174)

     at java.base/java.lang.Thread.run(Thread.java:829)

 Caused by: java.lang.OutOfMemoryError

     at java.base/jdk.internal.reflect.GeneratedConstructorAccessor3.newInstance(Unknown Source)

     at java.base/jdk.internal.reflect.DelegatingConstructorAccessorImpl.newInstance(DelegatingConstructorAccessorImpl.java:45)

     at java.base/java.lang.reflect.Constructor.newInstance(Constructor.java:490)

     at java.base/java.util.concurrent.ForkJoinTask.getThrowableException(ForkJoinTask.java:603)

     ... 49 more

 Caused by: java.lang.OutOfMemoryError: Java heap space

 java.util.concurrent.ExecutionException: java.lang.OutOfMemoryError

     at java.base/java.util.concurrent.ForkJoinTask.get(ForkJoinTask.java:1006)

     at fr.univ_nantes.codex_processor.gui.DatasetExtendedDepthOfFieldProcessor.run(DatasetExtendedDepthOfFieldProcessor.java:69)

     at fr.univ_nantes.codex_processor.gui.MainController$1.handle(MainController.java:96)

     at fr.univ_nantes.codex_processor.gui.MainController$1.handle(MainController.java:64)

     at com.sun.javafx.event.CompositeEventHandler.dispatchBubblingEvent(CompositeEventHandler.java:86)

     at com.sun.javafx.event.EventHandlerManager.dispatchBubblingEvent(EventHandlerManager.java:234)

     at com.sun.javafx.event.EventHandlerManager.dispatchBubblingEvent(EventHandlerManager.java:191)

     at com.sun.javafx.event.CompositeEventDispatcher.dispatchBubblingEvent(CompositeEventDispatcher.java:59)

     at com.sun.javafx.event.BasicEventDispatcher.dispatchEvent(BasicEventDispatcher.java:58)

     at com.sun.javafx.event.EventDispatchChainImpl.dispatchEvent(EventDispatchChainImpl.java:114)

     at com.sun.javafx.event.BasicEventDispatcher.dispatchEvent(BasicEventDispatcher.java:56)

     at com.sun.javafx.event.EventDispatchChainImpl.dispatchEvent(EventDispatchChainImpl.java:114)

     at com.sun.javafx.event.BasicEventDispatcher.dispatchEvent(BasicEventDispatcher.java:56)

     at com.sun.javafx.event.EventDispatchChainImpl.dispatchEvent(EventDispatchChainImpl.java:114)

     at com.sun.javafx.event.EventUtil.fireEventImpl(EventUtil.java:74)

     at com.sun.javafx.event.EventUtil.fireEvent(EventUtil.java:49)

     at javafx.event.Event.fireEvent(Event.java:198)

     at javafx.scene.Node.fireEvent(Node.java:8889)

     at javafx.scene.control.Button.fire(Button.java:203)

     at com.sun.javafx.scene.control.behavior.ButtonBehavior.mouseReleased(ButtonBehavior.java:208)

     at com.sun.javafx.scene.control.inputmap.InputMap.handle(InputMap.java:274)

     at com.sun.javafx.event.CompositeEventHandler$NormalEventHandlerRecord.handleBubblingEvent(CompositeEventHandler.java:247)

     at com.sun.javafx.event.CompositeEventHandler.dispatchBubblingEvent(CompositeEventHandler.java:80)

     at com.sun.javafx.event.EventHandlerManager.dispatchBubblingEvent(EventHandlerManager.java:234)

     at com.sun.javafx.event.EventHandlerManager.dispatchBubblingEvent(EventHandlerManager.java:191)

     at com.sun.javafx.event.CompositeEventDispatcher.dispatchBubblingEvent(CompositeEventDispatcher.java:59)

     at com.sun.javafx.event.BasicEventDispatcher.dispatchEvent(BasicEventDispatcher.java:58)

     at com.sun.javafx.event.EventDispatchChainImpl.dispatchEvent(EventDispatchChainImpl.java:114)

     at com.sun.javafx.event.BasicEventDispatcher.dispatchEvent(BasicEventDispatcher.java:56)

     at com.sun.javafx.event.EventDispatchChainImpl.dispatchEvent(EventDispatchChainImpl.java:114)

     at com.sun.javafx.event.BasicEventDispatcher.dispatchEvent(BasicEventDispatcher.java:56)

     at com.sun.javafx.event.EventDispatchChainImpl.dispatchEvent(EventDispatchChainImpl.java:114)

     at com.sun.javafx.event.EventUtil.fireEventImpl(EventUtil.java:74)

     at com.sun.javafx.event.EventUtil.fireEvent(EventUtil.java:54)

     at javafx.event.Event.fireEvent(Event.java:198)

     at javafx.scene.Scene$MouseHandler.process(Scene.java:3856)

     at javafx.scene.Scene.processMouseEvent(Scene.java:1851)

     at javafx.scene.Scene$ScenePeerListener.mouseEvent(Scene.java:2584)

     at com.sun.javafx.tk.quantum.GlassViewEventHandler$MouseEventNotification.run(GlassViewEventHandler.java:409)

     at com.sun.javafx.tk.quantum.GlassViewEventHandler$MouseEventNotification.run(GlassViewEventHandler.java:299)

     at java.base/java.security.AccessController.doPrivileged(Native Method)

     at com.sun.javafx.tk.quantum.GlassViewEventHandler.lambda$handleMouseEvent$2(GlassViewEventHandler.java:447)

     at com.sun.javafx.tk.quantum.QuantumToolkit.runWithoutRenderLock(QuantumToolkit.java:412)

     at com.sun.javafx.tk.quantum.GlassViewEventHandler.handleMouseEvent(GlassViewEventHandler.java:446)

     at com.sun.glass.ui.View.handleMouseEvent(View.java:556)

     at com.sun.glass.ui.View.notifyMouse(View.java:942)

     at com.sun.glass.ui.win.WinApplication._runLoop(Native Method)

     at com.sun.glass.ui.win.WinApplication.lambda$runLoop$3(WinApplication.java:174)

     at java.base/java.lang.Thread.run(Thread.java:829)

 Caused by: java.lang.OutOfMemoryError

     at java.base/jdk.internal.reflect.GeneratedConstructorAccessor3.newInstance(Unknown Source)

     at java.base/jdk.internal.reflect.DelegatingConstructorAccessorImpl.newInstance(DelegatingConstructorAccessorImpl.java:45)

     at java.base/java.lang.reflect.Constructor.newInstance(Constructor.java:490)

     at java.base/java.util.concurrent.ForkJoinTask.getThrowableException(ForkJoinTask.java:603)

     ... 49 more

 Caused by: java.lang.OutOfMemoryError: Java heap space

 java.util.concurrent.ExecutionException: java.lang.OutOfMemoryError

     at java.base/java.util.concurrent.ForkJoinTask.get(ForkJoinTask.java:1006)

     at fr.univ_nantes.codex_processor.gui.DatasetExtendedDepthOfFieldProcessor.run(DatasetExtendedDepthOfFieldProcessor.java:69)

     at fr.univ_nantes.codex_processor.gui.MainController$1.handle(MainController.java:96)

     at fr.univ_nantes.codex_processor.gui.MainController$1.handle(MainController.java:64)

     at com.sun.javafx.event.CompositeEventHandler.dispatchBubblingEvent(CompositeEventHandler.java:86)

     at com.sun.javafx.event.EventHandlerManager.dispatchBubblingEvent(EventHandlerManager.java:234)

     at com.sun.javafx.event.EventHandlerManager.dispatchBubblingEvent(EventHandlerManager.java:191)

     at com.sun.javafx.event.CompositeEventDispatcher.dispatchBubblingEvent(CompositeEventDispatcher.java:59)

     at com.sun.javafx.event.BasicEventDispatcher.dispatchEvent(BasicEventDispatcher.java:58)

     at com.sun.javafx.event.EventDispatchChainImpl.dispatchEvent(EventDispatchChainImpl.java:114)

     at com.sun.javafx.event.BasicEventDispatcher.dispatchEvent(BasicEventDispatcher.java:56)

     at com.sun.javafx.event.EventDispatchChainImpl.dispatchEvent(EventDispatchChainImpl.java:114)

     at com.sun.javafx.event.BasicEventDispatcher.dispatchEvent(BasicEventDispatcher.java:56)

     at com.sun.javafx.event.EventDispatchChainImpl.dispatchEvent(EventDispatchChainImpl.java:114)

     at com.sun.javafx.event.EventUtil.fireEventImpl(EventUtil.java:74)

     at com.sun.javafx.event.EventUtil.fireEvent(EventUtil.java:49)

     at javafx.event.Event.fireEvent(Event.java:198)

     at javafx.scene.Node.fireEvent(Node.java:8889)

     at javafx.scene.control.Button.fire(Button.java:203)

     at com.sun.javafx.scene.control.behavior.ButtonBehavior.mouseReleased(ButtonBehavior.java:208)

     at com.sun.javafx.scene.control.inputmap.InputMap.handle(InputMap.java:274)

     at com.sun.javafx.event.CompositeEventHandler$NormalEventHandlerRecord.handleBubblingEvent(CompositeEventHandler.java:247)

     at com.sun.javafx.event.CompositeEventHandler.dispatchBubblingEvent(CompositeEventHandler.java:80)

     at com.sun.javafx.event.EventHandlerManager.dispatchBubblingEvent(EventHandlerManager.java:234)

     at com.sun.javafx.event.EventHandlerManager.dispatchBubblingEvent(EventHandlerManager.java:191)

     at com.sun.javafx.event.CompositeEventDispatcher.dispatchBubblingEvent(CompositeEventDispatcher.java:59)

     at com.sun.javafx.event.BasicEventDispatcher.dispatchEvent(BasicEventDispatcher.java:58)

     at com.sun.javafx.event.EventDispatchChainImpl.dispatchEvent(EventDispatchChainImpl.java:114)

     at com.sun.javafx.event.BasicEventDispatcher.dispatchEvent(BasicEventDispatcher.java:56)

     at com.sun.javafx.event.EventDispatchChainImpl.dispatchEvent(EventDispatchChainImpl.java:114)

     at com.sun.javafx.event.BasicEventDispatcher.dispatchEvent(BasicEventDispatcher.java:56)

     at com.sun.javafx.event.EventDispatchChainImpl.dispatchEvent(EventDispatchChainImpl.java:114)

     at com.sun.javafx.event.EventUtil.fireEventImpl(EventUtil.java:74)

     at com.sun.javafx.event.EventUtil.fireEvent(EventUtil.java:54)

     at javafx.event.Event.fireEvent(Event.java:198)

     at javafx.scene.Scene$MouseHandler.process(Scene.java:3856)

     at javafx.scene.Scene.processMouseEvent(Scene.java:1851)

     at javafx.scene.Scene$ScenePeerListener.mouseEvent(Scene.java:2584)

     at com.sun.javafx.tk.quantum.GlassViewEventHandler$MouseEventNotification.run(GlassViewEventHandler.java:409)

     at com.sun.javafx.tk.quantum.GlassViewEventHandler$MouseEventNotification.run(GlassViewEventHandler.java:299)

     at java.base/java.security.AccessController.doPrivileged(Native Method)

     at com.sun.javafx.tk.quantum.GlassViewEventHandler.lambda$handleMouseEvent$2(GlassViewEventHandler.java:447)

     at com.sun.javafx.tk.quantum.QuantumToolkit.runWithoutRenderLock(QuantumToolkit.java:412)

     at com.sun.javafx.tk.quantum.GlassViewEventHandler.handleMouseEvent(GlassViewEventHandler.java:446)

     at com.sun.glass.ui.View.handleMouseEvent(View.java:556)

     at com.sun.glass.ui.View.notifyMouse(View.java:942)

     at com.sun.glass.ui.win.WinApplication._runLoop(Native Method)

     at com.sun.glass.ui.win.WinApplication.lambda$runLoop$3(WinApplication.java:174)

     at java.base/java.lang.Thread.run(Thread.java:829)

 Caused by: java.lang.OutOfMemoryError

     at java.base/jdk.internal.reflect.GeneratedConstructorAccessor3.newInstance(Unknown Source)

     at java.base/jdk.internal.reflect.DelegatingConstructorAccessorImpl.newInstance(DelegatingConstructorAccessorImpl.java:45)

     at java.base/java.lang.reflect.Constructor.newInstance(Constructor.java:490)

     at java.base/java.util.concurrent.ForkJoinTask.getThrowableException(ForkJoinTask.java:603)

     ... 49 more

 Caused by: java.lang.OutOfMemoryError: Java heap space

 java.util.concurrent.ExecutionException: java.lang.OutOfMemoryError

     at java.base/java.util.concurrent.ForkJoinTask.get(ForkJoinTask.java:1006)

     at fr.univ_nantes.codex_processor.gui.DatasetExtendedDepthOfFieldProcessor.run(DatasetExtendedDepthOfFieldProcessor.java:69)

     at fr.univ_nantes.codex_processor.gui.MainController$1.handle(MainController.java:96)

     at fr.univ_nantes.codex_processor.gui.MainController$1.handle(MainController.java:64)

     at com.sun.javafx.event.CompositeEventHandler.dispatchBubblingEvent(CompositeEventHandler.java:86)

     at com.sun.javafx.event.EventHandlerManager.dispatchBubblingEvent(EventHandlerManager.java:234)

     at com.sun.javafx.event.EventHandlerManager.dispatchBubblingEvent(EventHandlerManager.java:191)

     at com.sun.javafx.event.CompositeEventDispatcher.dispatchBubblingEvent(CompositeEventDispatcher.java:59)

     at com.sun.javafx.event.BasicEventDispatcher.dispatchEvent(BasicEventDispatcher.java:58)

     at com.sun.javafx.event.EventDispatchChainImpl.dispatchEvent(EventDispatchChainImpl.java:114)

     at com.sun.javafx.event.BasicEventDispatcher.dispatchEvent(BasicEventDispatcher.java:56)

     at com.sun.javafx.event.EventDispatchChainImpl.dispatchEvent(EventDispatchChainImpl.java:114)

     at com.sun.javafx.event.BasicEventDispatcher.dispatchEvent(BasicEventDispatcher.java:56)

     at com.sun.javafx.event.EventDispatchChainImpl.dispatchEvent(EventDispatchChainImpl.java:114)

     at com.sun.javafx.event.EventUtil.fireEventImpl(EventUtil.java:74)

     at com.sun.javafx.event.EventUtil.fireEvent(EventUtil.java:49)

     at javafx.event.Event.fireEvent(Event.java:198)

     at javafx.scene.Node.fireEvent(Node.java:8889)

     at javafx.scene.control.Button.fire(Button.java:203)

     at com.sun.javafx.scene.control.behavior.ButtonBehavior.mouseReleased(ButtonBehavior.java:208)

     at com.sun.javafx.scene.control.inputmap.InputMap.handle(InputMap.java:274)

     at com.sun.javafx.event.CompositeEventHandler$NormalEventHandlerRecord.handleBubblingEvent(CompositeEventHandler.java:247)

     at com.sun.javafx.event.CompositeEventHandler.dispatchBubblingEvent(CompositeEventHandler.java:80)

     at com.sun.javafx.event.EventHandlerManager.dispatchBubblingEvent(EventHandlerManager.java:234)

     at com.sun.javafx.event.EventHandlerManager.dispatchBubblingEvent(EventHandlerManager.java:191)

     at com.sun.javafx.event.CompositeEventDispatcher.dispatchBubblingEvent(CompositeEventDispatcher.java:59)

     at com.sun.javafx.event.BasicEventDispatcher.dispatchEvent(BasicEventDispatcher.java:58)

     at com.sun.javafx.event.EventDispatchChainImpl.dispatchEvent(EventDispatchChainImpl.java:114)

     at com.sun.javafx.event.BasicEventDispatcher.dispatchEvent(BasicEventDispatcher.java:56)

     at com.sun.javafx.event.EventDispatchChainImpl.dispatchEvent(EventDispatchChainImpl.java:114)

     at com.sun.javafx.event.BasicEventDispatcher.dispatchEvent(BasicEventDispatcher.java:56)

     at com.sun.javafx.event.EventDispatchChainImpl.dispatchEvent(EventDispatchChainImpl.java:114)

     at com.sun.javafx.event.EventUtil.fireEventImpl(EventUtil.java:74)

     at com.sun.javafx.event.EventUtil.fireEvent(EventUtil.java:54)

     at javafx.event.Event.fireEvent(Event.java:198)

     at javafx.scene.Scene$MouseHandler.process(Scene.java:3856)

     at javafx.scene.Scene.processMouseEvent(Scene.java:1851)

     at javafx.scene.Scene$ScenePeerListener.mouseEvent(Scene.java:2584)

     at com.sun.javafx.tk.quantum.GlassViewEventHandler$MouseEventNotification.run(GlassViewEventHandler.java:409)

     at com.sun.javafx.tk.quantum.GlassViewEventHandler$MouseEventNotification.run(GlassViewEventHandler.java:299)

     at java.base/java.security.AccessController.doPrivileged(Native Method)

     at com.sun.javafx.tk.quantum.GlassViewEventHandler.lambda$handleMouseEvent$2(GlassViewEventHandler.java:447)

     at com.sun.javafx.tk.quantum.QuantumToolkit.runWithoutRenderLock(QuantumToolkit.java:412)

     at com.sun.javafx.tk.quantum.GlassViewEventHandler.handleMouseEvent(GlassViewEventHandler.java:446)

     at com.sun.glass.ui.View.handleMouseEvent(View.java:556)

     at com.sun.glass.ui.View.notifyMouse(View.java:942)

     at com.sun.glass.ui.win.WinApplication._runLoop(Native Method)

     at com.sun.glass.ui.win.WinApplication.lambda$runLoop$3(WinApplication.java:174)

     at java.base/java.lang.Thread.run(Thread.java:829)

 Caused by: java.lang.OutOfMemoryError

     at java.base/jdk.internal.reflect.GeneratedConstructorAccessor3.newInstance(Unknown Source)

     at java.base/jdk.internal.reflect.DelegatingConstructorAccessorImpl.newInstance(DelegatingConstructorAccessorImpl.java:45)

     at java.base/java.lang.reflect.Constructor.newInstance(Constructor.java:490)

     at java.base/java.util.concurrent.ForkJoinTask.getThrowableException(ForkJoinTask.java:603)

     ... 49 more

 Caused by: java.lang.OutOfMemoryError: Java heap space

 WARNING: An illegal reflective access operation has occurred

 WARNING: Illegal reflective access by gov.nist.isg.mist.lib.libraryloader.LibraryUtils (file:/C:/Users/IT-WL-annkl878/Documents/notes/Codex_Analysis/MultiplexProcessor/gui-0.1.0-SNAPSHOT.jar) to field java.lang.ClassLoader.usr_paths

 WARNING: Please consider reporting this to the maintainers of gov.nist.isg.mist.lib.libraryloader.LibraryUtils

 WARNING: Use --illegal-access=warn to enable warnings of further illegal reflective access operations

 WARNING: All illegal access operations will be denied in a future release

 Python was not found; run without arguments to install from the Microsoft Store, or disable this shortcut from Settings > Manage App Execution Aliases.

 java.nio.file.NoSuchFileException: C:\Users\IT-WL-annkl878\Documents\notes\Codex_Analysis\MultiplexProcessor\labels.tif

     at java.base/sun.nio.fs.WindowsException.translateToIOException(WindowsException.java:85)

     at java.base/sun.nio.fs.WindowsException.rethrowAsIOException(WindowsException.java:103)

     at java.base/sun.nio.fs.WindowsException.rethrowAsIOException(WindowsException.java:108)

     at java.base/sun.nio.fs.WindowsFileCopy.move(WindowsFileCopy.java:326)

     at java.base/sun.nio.fs.WindowsFileSystemProvider.move(WindowsFileSystemProvider.java:292)

     at java.base/java.nio.file.Files.move(Files.java:1422)

     at fr.univ_nantes.codex_processor.gui.StardistDatasetSegmentationProcessor.run(StardistDatasetSegmentationProcessor.java:36)

     at fr.univ_nantes.codex_processor.gui.MainController$1.handle(MainController.java:144)

     at fr.univ_nantes.codex_processor.gui.MainController$1.handle(MainController.java:64)

     at com.sun.javafx.event.CompositeEventHandler.dispatchBubblingEvent(CompositeEventHandler.java:86)

     at com.sun.javafx.event.EventHandlerManager.dispatchBubblingEvent(EventHandlerManager.java:234)

     at com.sun.javafx.event.EventHandlerManager.dispatchBubblingEvent(EventHandlerManager.java:191)

     at com.sun.javafx.event.CompositeEventDispatcher.dispatchBubblingEvent(CompositeEventDispatcher.java:59)

     at com.sun.javafx.event.BasicEventDispatcher.dispatchEvent(BasicEventDispatcher.java:58)

     at com.sun.javafx.event.EventDispatchChainImpl.dispatchEvent(EventDispatchChainImpl.java:114)

     at com.sun.javafx.event.BasicEventDispatcher.dispatchEvent(BasicEventDispatcher.java:56)

     at com.sun.javafx.event.EventDispatchChainImpl.dispatchEvent(EventDispatchChainImpl.java:114)

     at com.sun.javafx.event.BasicEventDispatcher.dispatchEvent(BasicEventDispatcher.java:56)

     at com.sun.javafx.event.EventDispatchChainImpl.dispatchEvent(EventDispatchChainImpl.java:114)

     at com.sun.javafx.event.EventUtil.fireEventImpl(EventUtil.java:74)

     at com.sun.javafx.event.EventUtil.fireEvent(EventUtil.java:49)

     at javafx.event.Event.fireEvent(Event.java:198)

     at javafx.scene.Node.fireEvent(Node.java:8889)

     at javafx.scene.control.Button.fire(Button.java:203)

     at com.sun.javafx.scene.control.behavior.ButtonBehavior.mouseReleased(ButtonBehavior.java:208)

     at com.sun.javafx.scene.control.inputmap.InputMap.handle(InputMap.java:274)

     at com.sun.javafx.event.CompositeEventHandler$NormalEventHandlerRecord.handleBubblingEvent(CompositeEventHandler.java:247)

     at com.sun.javafx.event.CompositeEventHandler.dispatchBubblingEvent(CompositeEventHandler.java:80)

     at com.sun.javafx.event.EventHandlerManager.dispatchBubblingEvent(EventHandlerManager.java:234)

     at com.sun.javafx.event.EventHandlerManager.dispatchBubblingEvent(EventHandlerManager.java:191)

     at com.sun.javafx.event.CompositeEventDispatcher.dispatchBubblingEvent(CompositeEventDispatcher.java:59)

     at com.sun.javafx.event.BasicEventDispatcher.dispatchEvent(BasicEventDispatcher.java:58)

     at com.sun.javafx.event.EventDispatchChainImpl.dispatchEvent(EventDispatchChainImpl.java:114)

     at com.sun.javafx.event.BasicEventDispatcher.dispatchEvent(BasicEventDispatcher.java:56)

     at com.sun.javafx.event.EventDispatchChainImpl.dispatchEvent(EventDispatchChainImpl.java:114)

     at com.sun.javafx.event.BasicEventDispatcher.dispatchEvent(BasicEventDispatcher.java:56)

     at com.sun.javafx.event.EventDispatchChainImpl.dispatchEvent(EventDispatchChainImpl.java:114)

     at com.sun.javafx.event.EventUtil.fireEventImpl(EventUtil.java:74)

     at com.sun.javafx.event.EventUtil.fireEvent(EventUtil.java:54)

     at javafx.event.Event.fireEvent(Event.java:198)

     at javafx.scene.Scene$MouseHandler.process(Scene.java:3856)

     at javafx.scene.Scene.processMouseEvent(Scene.java:1851)

     at javafx.scene.Scene$ScenePeerListener.mouseEvent(Scene.java:2584)

     at com.sun.javafx.tk.quantum.GlassViewEventHandler$MouseEventNotification.run(GlassViewEventHandler.java:409)

     at com.sun.javafx.tk.quantum.GlassViewEventHandler$MouseEventNotification.run(GlassViewEventHandler.java:299)

     at java.base/java.security.AccessController.doPrivileged(Native Method)

     at com.sun.javafx.tk.quantum.GlassViewEventHandler.lambda$handleMouseEvent$2(GlassViewEventHandler.java:447)

     at com.sun.javafx.tk.quantum.QuantumToolkit.runWithoutRenderLock(QuantumToolkit.java:412)

     at com.sun.javafx.tk.quantum.GlassViewEventHandler.handleMouseEvent(GlassViewEventHandler.java:446)

     at com.sun.glass.ui.View.handleMouseEvent(View.java:556)

     at com.sun.glass.ui.View.notifyMouse(View.java:942)

     at com.sun.glass.ui.win.WinApplication._runLoop(Native Method)

     at com.sun.glass.ui.win.WinApplication.lambda$runLoop$3(WinApplication.java:174)

     at java.base/java.lang.Thread.run(Thread.java:829)

 java.nio.file.NoSuchFileException: C:\Users\IT-WL-annkl878\Documents\notes\Codex_Analysis\test_multiplxProcessor\out\rois_cy002_ch1.zip

     at java.base/sun.nio.fs.WindowsException.translateToIOException(WindowsException.java:85)

     at java.base/sun.nio.fs.WindowsException.rethrowAsIOException(WindowsException.java:103)

     at java.base/sun.nio.fs.WindowsException.rethrowAsIOException(WindowsException.java:108)

     at java.base/sun.nio.fs.WindowsFileAttributeViews$Basic.readAttributes(WindowsFileAttributeViews.java:53)

     at java.base/sun.nio.fs.WindowsFileAttributeViews$Basic.readAttributes(WindowsFileAttributeViews.java:38)

     at java.base/sun.nio.fs.WindowsFileSystemProvider.readAttributes(WindowsFileSystemProvider.java:198)

     at java.base/java.nio.file.Files.readAttributes(Files.java:1764)

     at java.base/java.util.zip.ZipFile$Source.get(ZipFile.java:1259)

     at java.base/java.util.zip.ZipFile$CleanableResource.(ZipFile.java:733)

     at java.base/java.util.zip.ZipFile$CleanableResource.get(ZipFile.java:850)

     at java.base/java.util.zip.ZipFile.(ZipFile.java:248)

     at java.base/java.util.zip.ZipFile.(ZipFile.java:177)

     at java.base/java.util.zip.ZipFile.(ZipFile.java:191)

     at fr.univ_nantes.codex_processor.gui.CreateSegmentationMaskFromRois.run(CreateSegmentationMaskFromRois.java:32)

     at fr.univ_nantes.codex_processor.gui.MainController$1.handle(MainController.java:152)

     at fr.univ_nantes.codex_processor.gui.MainController$1.handle(MainController.java:64)

     at com.sun.javafx.event.CompositeEventHandler.dispatchBubblingEvent(CompositeEventHandler.java:86)

     at com.sun.javafx.event.EventHandlerManager.dispatchBubblingEvent(EventHandlerManager.java:234)

     at com.sun.javafx.event.EventHandlerManager.dispatchBubblingEvent(EventHandlerManager.java:191)

     at com.sun.javafx.event.CompositeEventDispatcher.dispatchBubblingEvent(CompositeEventDispatcher.java:59)

     at com.sun.javafx.event.BasicEventDispatcher.dispatchEvent(BasicEventDispatcher.java:58)

     at com.sun.javafx.event.EventDispatchChainImpl.dispatchEvent(EventDispatchChainImpl.java:114)

     at com.sun.javafx.event.BasicEventDispatcher.dispatchEvent(BasicEventDispatcher.java:56)

     at com.sun.javafx.event.EventDispatchChainImpl.dispatchEvent(EventDispatchChainImpl.java:114)

     at com.sun.javafx.event.BasicEventDispatcher.dispatchEvent(BasicEventDispatcher.java:56)

     at com.sun.javafx.event.EventDispatchChainImpl.dispatchEvent(EventDispatchChainImpl.java:114)

     at com.sun.javafx.event.EventUtil.fireEventImpl(EventUtil.java:74)

     at com.sun.javafx.event.EventUtil.fireEvent(EventUtil.java:49)

     at javafx.event.Event.fireEvent(Event.java:198)

     at javafx.scene.Node.fireEvent(Node.java:8889)

     at javafx.scene.control.Button.fire(Button.java:203)

     at com.sun.javafx.scene.control.behavior.ButtonBehavior.mouseReleased(ButtonBehavior.java:208)

     at com.sun.javafx.scene.control.inputmap.InputMap.handle(InputMap.java:274)

     at com.sun.javafx.event.CompositeEventHandler$NormalEventHandlerRecord.handleBubblingEvent(CompositeEventHandler.java:247)

     at com.sun.javafx.event.CompositeEventHandler.dispatchBubblingEvent(CompositeEventHandler.java:80)

     at com.sun.javafx.event.EventHandlerManager.dispatchBubblingEvent(EventHandlerManager.java:234)

     at com.sun.javafx.event.EventHandlerManager.dispatchBubblingEvent(EventHandlerManager.java:191)

     at com.sun.javafx.event.CompositeEventDispatcher.dispatchBubblingEvent(CompositeEventDispatcher.java:59)

     at com.sun.javafx.event.BasicEventDispatcher.dispatchEvent(BasicEventDispatcher.java:58)

     at com.sun.javafx.event.EventDispatchChainImpl.dispatchEvent(EventDispatchChainImpl.java:114)

     at com.sun.javafx.event.BasicEventDispatcher.dispatchEvent(BasicEventDispatcher.java:56)

     at com.sun.javafx.event.EventDispatchChainImpl.dispatchEvent(EventDispatchChainImpl.java:114)

     at com.sun.javafx.event.BasicEventDispatcher.dispatchEvent(BasicEventDispatcher.java:56)

     at com.sun.javafx.event.EventDispatchChainImpl.dispatchEvent(EventDispatchChainImpl.java:114)

     at com.sun.javafx.event.EventUtil.fireEventImpl(EventUtil.java:74)

     at com.sun.javafx.event.EventUtil.fireEvent(EventUtil.java:54)

     at javafx.event.Event.fireEvent(Event.java:198)

     at javafx.scene.Scene$MouseHandler.process(Scene.java:3856)

     at javafx.scene.Scene.processMouseEvent(Scene.java:1851)

     at javafx.scene.Scene$ScenePeerListener.mouseEvent(Scene.java:2584)

     at com.sun.javafx.tk.quantum.GlassViewEventHandler$MouseEventNotification.run(GlassViewEventHandler.java:409)

     at com.sun.javafx.tk.quantum.GlassViewEventHandler$MouseEventNotification.run(GlassViewEventHandler.java:299)

     at java.base/java.security.AccessController.doPrivileged(Native Method)

     at com.sun.javafx.tk.quantum.GlassViewEventHandler.lambda$handleMouseEvent$2(GlassViewEventHandler.java:447)

     at com.sun.javafx.tk.quantum.QuantumToolkit.runWithoutRenderLock(QuantumToolkit.java:412)

     at com.sun.javafx.tk.quantum.GlassViewEventHandler.handleMouseEvent(GlassViewEventHandler.java:446)

     at com.sun.glass.ui.View.handleMouseEvent(View.java:556)

     at com.sun.glass.ui.View.notifyMouse(View.java:942)

     at com.sun.glass.ui.win.WinApplication._runLoop(Native Method)

     at com.sun.glass.ui.win.WinApplication.lambda$runLoop$3(WinApplication.java:174)

     at java.base/java.lang.Thread.run(Thread.java:829)

 java.nio.file.NoSuchFileException: C:\Users\IT-WL-annkl878\Documents\notes\Codex_Analysis\test_multiplxProcessor\out\rois_cy002_ch1.zip

     at java.base/sun.nio.fs.WindowsException.translateToIOException(WindowsException.java:85)

     at java.base/sun.nio.fs.WindowsException.rethrowAsIOException(WindowsException.java:103)

     at java.base/sun.nio.fs.WindowsException.rethrowAsIOException(WindowsException.java:108)

     at java.base/sun.nio.fs.WindowsFileAttributeViews$Basic.readAttributes(WindowsFileAttributeViews.java:53)

     at java.base/sun.nio.fs.WindowsFileAttributeViews$Basic.readAttributes(WindowsFileAttributeViews.java:38)

     at java.base/sun.nio.fs.WindowsFileSystemProvider.readAttributes(WindowsFileSystemProvider.java:198)

     at java.base/java.nio.file.Files.readAttributes(Files.java:1764)

     at java.base/java.util.zip.ZipFile$Source.get(ZipFile.java:1259)

     at java.base/java.util.zip.ZipFile$CleanableResource.(ZipFile.java:733)

     at java.base/java.util.zip.ZipFile$CleanableResource.get(ZipFile.java:850)

     at java.base/java.util.zip.ZipFile.(ZipFile.java:248)

     at java.base/java.util.zip.ZipFile.(ZipFile.java:177)

     at java.base/java.util.zip.ZipFile.(ZipFile.java:191)

     at fr.univ_nantes.codex_processor.core.measure.MeasureProcessor.run(MeasureProcessor.java:43)

     at fr.univ_nantes.codex_processor.gui.DatasetMeasureProcessor.run(DatasetMeasureProcessor.java:45)

     at fr.univ_nantes.codex_processor.gui.MainController$1.handle(MainController.java:160)

     at fr.univ_nantes.codex_processor.gui.MainController$1.handle(MainController.java:64)

     at com.sun.javafx.event.CompositeEventHandler.dispatchBubblingEvent(CompositeEventHandler.java:86)

     at com.sun.javafx.event.EventHandlerManager.dispatchBubblingEvent(EventHandlerManager.java:234)

     at com.sun.javafx.event.EventHandlerManager.dispatchBubblingEvent(EventHandlerManager.java:191)

     at com.sun.javafx.event.CompositeEventDispatcher.dispatchBubblingEvent(CompositeEventDispatcher.java:59)

     at com.sun.javafx.event.BasicEventDispatcher.dispatchEvent(BasicEventDispatcher.java:58)

     at com.sun.javafx.event.EventDispatchChainImpl.dispatchEvent(EventDispatchChainImpl.java:114)

     at com.sun.javafx.event.BasicEventDispatcher.dispatchEvent(BasicEventDispatcher.java:56)

     at com.sun.javafx.event.EventDispatchChainImpl.dispatchEvent(EventDispatchChainImpl.java:114)

     at com.sun.javafx.event.BasicEventDispatcher.dispatchEvent(BasicEventDispatcher.java:56)

     at com.sun.javafx.event.EventDispatchChainImpl.dispatchEvent(EventDispatchChainImpl.java:114)

     at com.sun.javafx.event.EventUtil.fireEventImpl(EventUtil.java:74)

     at com.sun.javafx.event.EventUtil.fireEvent(EventUtil.java:49)

     at javafx.event.Event.fireEvent(Event.java:198)

     at javafx.scene.Node.fireEvent(Node.java:8889)

     at javafx.scene.control.Button.fire(Button.java:203)

     at com.sun.javafx.scene.control.behavior.ButtonBehavior.mouseReleased(ButtonBehavior.java:208)

     at com.sun.javafx.scene.control.inputmap.InputMap.handle(InputMap.java:274)

     at com.sun.javafx.event.CompositeEventHandler$NormalEventHandlerRecord.handleBubblingEvent(CompositeEventHandler.java:247)

     at com.sun.javafx.event.CompositeEventHandler.dispatchBubblingEvent(CompositeEventHandler.java:80)

     at com.sun.javafx.event.EventHandlerManager.dispatchBubblingEvent(EventHandlerManager.java:234)

     at com.sun.javafx.event.EventHandlerManager.dispatchBubblingEvent(EventHandlerManager.java:191)

     at com.sun.javafx.event.CompositeEventDispatcher.dispatchBubblingEvent(CompositeEventDispatcher.java:59)

     at com.sun.javafx.event.BasicEventDispatcher.dispatchEvent(BasicEventDispatcher.java:58)

     at com.sun.javafx.event.EventDispatchChainImpl.dispatchEvent(EventDispatchChainImpl.java:114)

     at com.sun.javafx.event.BasicEventDispatcher.dispatchEvent(BasicEventDispatcher.java:56)

     at com.sun.javafx.event.EventDispatchChainImpl.dispatchEvent(EventDispatchChainImpl.java:114)

     at com.sun.javafx.event.BasicEventDispatcher.dispatchEvent(BasicEventDispatcher.java:56)

     at com.sun.javafx.event.EventDispatchChainImpl.dispatchEvent(EventDispatchChainImpl.java:114)

     at com.sun.javafx.event.EventUtil.fireEventImpl(EventUtil.java:74)

     at com.sun.javafx.event.EventUtil.fireEvent(EventUtil.java:54)

     at javafx.event.Event.fireEvent(Event.java:198)

     at javafx.scene.Scene$MouseHandler.process(Scene.java:3856)

     at javafx.scene.Scene.processMouseEvent(Scene.java:1851)

     at javafx.scene.Scene$ScenePeerListener.mouseEvent(Scene.java:2584)

     at com.sun.javafx.tk.quantum.GlassViewEventHandler$MouseEventNotification.run(GlassViewEventHandler.java:409)

     at com.sun.javafx.tk.quantum.GlassViewEventHandler$MouseEventNotification.run(GlassViewEventHandler.java:299)

     at java.base/java.security.AccessController.doPrivileged(Native Method)

     at com.sun.javafx.tk.quantum.GlassViewEventHandler.lambda$handleMouseEvent$2(GlassViewEventHandler.java:447)

     at com.sun.javafx.tk.quantum.QuantumToolkit.runWithoutRenderLock(QuantumToolkit.java:412)

     at com.sun.javafx.tk.quantum.GlassViewEventHandler.handleMouseEvent(GlassViewEventHandler.java:446)

     at com.sun.glass.ui.View.handleMouseEvent(View.java:556)

     at com.sun.glass.ui.View.notifyMouse(View.java:942)

     at com.sun.glass.ui.win.WinApplication._runLoop(Native Method)

     at com.sun.glass.ui.win.WinApplication.lambda$runLoop$3(WinApplication.java:174)

     at java.base/java.lang.Thread.run(Thread.java:829)

 java.nio.file.NoSuchFileException: C:\Users\IT-WL-annkl878\Documents\notes\Codex_Analysis\test_multiplxProcessor\out\rois_cy002_ch1.zip

     at java.base/sun.nio.fs.WindowsException.translateToIOException(WindowsException.java:85)

     at java.base/sun.nio.fs.WindowsException.rethrowAsIOException(WindowsException.java:103)

     at java.base/sun.nio.fs.WindowsException.rethrowAsIOException(WindowsException.java:108)

     at java.base/sun.nio.fs.WindowsFileAttributeViews$Basic.readAttributes(WindowsFileAttributeViews.java:53)

     at java.base/sun.nio.fs.WindowsFileAttributeViews$Basic.readAttributes(WindowsFileAttributeViews.java:38)

     at java.base/sun.nio.fs.WindowsFileSystemProvider.readAttributes(WindowsFileSystemProvider.java:198)

     at java.base/java.nio.file.Files.readAttributes(Files.java:1764)

     at java.base/java.util.zip.ZipFile$Source.get(ZipFile.java:1259)

     at java.base/java.util.zip.ZipFile$CleanableResource.(ZipFile.java:733)

     at java.base/java.util.zip.ZipFile$CleanableResource.get(ZipFile.java:850)

     at java.base/java.util.zip.ZipFile.(ZipFile.java:248)

     at java.base/java.util.zip.ZipFile.(ZipFile.java:177)

     at java.base/java.util.zip.ZipFile.(ZipFile.java:191)

     at fr.univ_nantes.codex_processor.core.measure.MeasureProcessor.run(MeasureProcessor.java:43)

     at fr.univ_nantes.codex_processor.gui.DatasetMeasureProcessor.run(DatasetMeasureProcessor.java:45)

     at fr.univ_nantes.codex_processor.gui.MainController$1.handle(MainController.java:160)

     at fr.univ_nantes.codex_processor.gui.MainController$1.handle(MainController.java:64)

     at com.sun.javafx.event.CompositeEventHandler.dispatchBubblingEvent(CompositeEventHandler.java:86)

     at com.sun.javafx.event.EventHandlerManager.dispatchBubblingEvent(EventHandlerManager.java:234)

     at com.sun.javafx.event.EventHandlerManager.dispatchBubblingEvent(EventHandlerManager.java:191)

     at com.sun.javafx.event.CompositeEventDispatcher.dispatchBubblingEvent(CompositeEventDispatcher.java:59)

     at com.sun.javafx.event.BasicEventDispatcher.dispatchEvent(BasicEventDispatcher.java:58)

     at com.sun.javafx.event.EventDispatchChainImpl.dispatchEvent(EventDispatchChainImpl.java:114)

     at com.sun.javafx.event.BasicEventDispatcher.dispatchEvent(BasicEventDispatcher.java:56)

     at com.sun.javafx.event.EventDispatchChainImpl.dispatchEvent(EventDispatchChainImpl.java:114)

     at com.sun.javafx.event.BasicEventDispatcher.dispatchEvent(BasicEventDispatcher.java:56)

     at com.sun.javafx.event.EventDispatchChainImpl.dispatchEvent(EventDispatchChainImpl.java:114)

     at com.sun.javafx.event.EventUtil.fireEventImpl(EventUtil.java:74)

     at com.sun.javafx.event.EventUtil.fireEvent(EventUtil.java:49)

     at javafx.event.Event.fireEvent(Event.java:198)

     at javafx.scene.Node.fireEvent(Node.java:8889)

     at javafx.scene.control.Button.fire(Button.java:203)

     at com.sun.javafx.scene.control.behavior.ButtonBehavior.mouseReleased(ButtonBehavior.java:208)

     at com.sun.javafx.scene.control.inputmap.InputMap.handle(InputMap.java:274)

     at com.sun.javafx.event.CompositeEventHandler$NormalEventHandlerRecord.handleBubblingEvent(CompositeEventHandler.java:247)

     at com.sun.javafx.event.CompositeEventHandler.dispatchBubblingEvent(CompositeEventHandler.java:80)

     at com.sun.javafx.event.EventHandlerManager.dispatchBubblingEvent(EventHandlerManager.java:234)

     at com.sun.javafx.event.EventHandlerManager.dispatchBubblingEvent(EventHandlerManager.java:191)

     at com.sun.javafx.event.CompositeEventDispatcher.dispatchBubblingEvent(CompositeEventDispatcher.java:59)

     at com.sun.javafx.event.BasicEventDispatcher.dispatchEvent(BasicEventDispatcher.java:58)

     at com.sun.javafx.event.EventDispatchChainImpl.dispatchEvent(EventDispatchChainImpl.java:114)

     at com.sun.javafx.event.BasicEventDispatcher.dispatchEvent(BasicEventDispatcher.java:56)

     at com.sun.javafx.event.EventDispatchChainImpl.dispatchEvent(EventDispatchChainImpl.java:114)

     at com.sun.javafx.event.BasicEventDispatcher.dispatchEvent(BasicEventDispatcher.java:56)

     at com.sun.javafx.event.EventDispatchChainImpl.dispatchEvent(EventDispatchChainImpl.java:114)

     at com.sun.javafx.event.EventUtil.fireEventImpl(EventUtil.java:74)

     at com.sun.javafx.event.EventUtil.fireEvent(EventUtil.java:54)

     at javafx.event.Event.fireEvent(Event.java:198)

     at javafx.scene.Scene$MouseHandler.process(Scene.java:3856)

     at javafx.scene.Scene.processMouseEvent(Scene.java:1851)

     at javafx.scene.Scene$ScenePeerListener.mouseEvent(Scene.java:2584)

     at com.sun.javafx.tk.quantum.GlassViewEventHandler$MouseEventNotification.run(GlassViewEventHandler.java:409)

     at com.sun.javafx.tk.quantum.GlassViewEventHandler$MouseEventNotification.run(GlassViewEventHandler.java:299)

     at java.base/java.security.AccessController.doPrivileged(Native Method)

     at com.sun.javafx.tk.quantum.GlassViewEventHandler.lambda$handleMouseEvent$2(GlassViewEventHandler.java:447)

     at com.sun.javafx.tk.quantum.QuantumToolkit.runWithoutRenderLock(QuantumToolkit.java:412)

     at com.sun.javafx.tk.quantum.GlassViewEventHandler.handleMouseEvent(GlassViewEventHandler.java:446)

     at com.sun.glass.ui.View.handleMouseEvent(View.java:556)

     at com.sun.glass.ui.View.notifyMouse(View.java:942)

     at com.sun.glass.ui.win.WinApplication._runLoop(Native Method)

     at com.sun.glass.ui.win.WinApplication.lambda$runLoop$3(WinApplication.java:174)

     at java.base/java.lang.Thread.run(Thread.java:829)

 java.nio.file.NoSuchFileException: C:\Users\IT-WL-annkl878\Documents\notes\Codex_Analysis\test_multiplxProcessor\out\rois_cy002_ch1.zip

     at java.base/sun.nio.fs.WindowsException.translateToIOException(WindowsException.java:85)

     at java.base/sun.nio.fs.WindowsException.rethrowAsIOException(WindowsException.java:103)

     at java.base/sun.nio.fs.WindowsException.rethrowAsIOException(WindowsException.java:108)

     at java.base/sun.nio.fs.WindowsFileAttributeViews$Basic.readAttributes(WindowsFileAttributeViews.java:53)

     at java.base/sun.nio.fs.WindowsFileAttributeViews$Basic.readAttributes(WindowsFileAttributeViews.java:38)

     at java.base/sun.nio.fs.WindowsFileSystemProvider.readAttributes(WindowsFileSystemProvider.java:198)

     at java.base/java.nio.file.Files.readAttributes(Files.java:1764)

     at java.base/java.util.zip.ZipFile$Source.get(ZipFile.java:1259)

     at java.base/java.util.zip.ZipFile$CleanableResource.(ZipFile.java:733)

     at java.base/java.util.zip.ZipFile$CleanableResource.get(ZipFile.java:850)

     at java.base/java.util.zip.ZipFile.(ZipFile.java:248)

     at java.base/java.util.zip.ZipFile.(ZipFile.java:177)

     at java.base/java.util.zip.ZipFile.(ZipFile.java:191)

     at fr.univ_nantes.codex_processor.core.measure.MeasureProcessor.run(MeasureProcessor.java:43)

     at fr.univ_nantes.codex_processor.gui.DatasetMeasureProcessor.run(DatasetMeasureProcessor.java:45)

     at fr.univ_nantes.codex_processor.gui.MainController$1.handle(MainController.java:160)

     at fr.univ_nantes.codex_processor.gui.MainController$1.handle(MainController.java:64)

     at com.sun.javafx.event.CompositeEventHandler.dispatchBubblingEvent(CompositeEventHandler.java:86)

     at com.sun.javafx.event.EventHandlerManager.dispatchBubblingEvent(EventHandlerManager.java:234)

     at com.sun.javafx.event.EventHandlerManager.dispatchBubblingEvent(EventHandlerManager.java:191)

     at com.sun.javafx.event.CompositeEventDispatcher.dispatchBubblingEvent(CompositeEventDispatcher.java:59)

     at com.sun.javafx.event.BasicEventDispatcher.dispatchEvent(BasicEventDispatcher.java:58)

     at com.sun.javafx.event.EventDispatchChainImpl.dispatchEvent(EventDispatchChainImpl.java:114)

     at com.sun.javafx.event.BasicEventDispatcher.dispatchEvent(BasicEventDispatcher.java:56)

     at com.sun.javafx.event.EventDispatchChainImpl.dispatchEvent(EventDispatchChainImpl.java:114)

     at com.sun.javafx.event.BasicEventDispatcher.dispatchEvent(BasicEventDispatcher.java:56)

     at com.sun.javafx.event.EventDispatchChainImpl.dispatchEvent(EventDispatchChainImpl.java:114)

     at com.sun.javafx.event.EventUtil.fireEventImpl(EventUtil.java:74)

     at com.sun.javafx.event.EventUtil.fireEvent(EventUtil.java:49)

     at javafx.event.Event.fireEvent(Event.java:198)

     at javafx.scene.Node.fireEvent(Node.java:8889)

     at javafx.scene.control.Button.fire(Button.java:203)

     at com.sun.javafx.scene.control.behavior.ButtonBehavior.mouseReleased(ButtonBehavior.java:208)

     at com.sun.javafx.scene.control.inputmap.InputMap.handle(InputMap.java:274)

     at com.sun.javafx.event.CompositeEventHandler$NormalEventHandlerRecord.handleBubblingEvent(CompositeEventHandler.java:247)

     at com.sun.javafx.event.CompositeEventHandler.dispatchBubblingEvent(CompositeEventHandler.java:80)

     at com.sun.javafx.event.EventHandlerManager.dispatchBubblingEvent(EventHandlerManager.java:234)

     at com.sun.javafx.event.EventHandlerManager.dispatchBubblingEvent(EventHandlerManager.java:191)

     at com.sun.javafx.event.CompositeEventDispatcher.dispatchBubblingEvent(CompositeEventDispatcher.java:59)

     at com.sun.javafx.event.BasicEventDispatcher.dispatchEvent(BasicEventDispatcher.java:58)

     at com.sun.javafx.event.EventDispatchChainImpl.dispatchEvent(EventDispatchChainImpl.java:114)

     at com.sun.javafx.event.BasicEventDispatcher.dispatchEvent(BasicEventDispatcher.java:56)

     at com.sun.javafx.event.EventDispatchChainImpl.dispatchEvent(EventDispatchChainImpl.java:114)

     at com.sun.javafx.event.BasicEventDispatcher.dispatchEvent(BasicEventDispatcher.java:56)

     at com.sun.javafx.event.EventDispatchChainImpl.dispatchEvent(EventDispatchChainImpl.java:114)

     at com.sun.javafx.event.EventUtil.fireEventImpl(EventUtil.java:74)

     at com.sun.javafx.event.EventUtil.fireEvent(EventUtil.java:54)

     at javafx.event.Event.fireEvent(Event.java:198)

     at javafx.scene.Scene$MouseHandler.process(Scene.java:3856)

     at javafx.scene.Scene.processMouseEvent(Scene.java:1851)

     at javafx.scene.Scene$ScenePeerListener.mouseEvent(Scene.java:2584)

     at com.sun.javafx.tk.quantum.GlassViewEventHandler$MouseEventNotification.run(GlassViewEventHandler.java:409)

     at com.sun.javafx.tk.quantum.GlassViewEventHandler$MouseEventNotification.run(GlassViewEventHandler.java:299)

     at java.base/java.security.AccessController.doPrivileged(Native Method)

     at com.sun.javafx.tk.quantum.GlassViewEventHandler.lambda$handleMouseEvent$2(GlassViewEventHandler.java:447)

     at com.sun.javafx.tk.quantum.QuantumToolkit.runWithoutRenderLock(QuantumToolkit.java:412)

     at com.sun.javafx.tk.quantum.GlassViewEventHandler.handleMouseEvent(GlassViewEventHandler.java:446)

     at com.sun.glass.ui.View.handleMouseEvent(View.java:556)

     at com.sun.glass.ui.View.notifyMouse(View.java:942)

     at com.sun.glass.ui.win.WinApplication._runLoop(Native Method)

     at com.sun.glass.ui.win.WinApplication.lambda$runLoop$3(WinApplication.java:174)

     at java.base/java.lang.Thread.run(Thread.java:829)

 java.nio.file.NoSuchFileException: C:\Users\IT-WL-annkl878\Documents\notes\Codex_Analysis\test_multiplxProcessor\out\rois_cy002_ch1.zip

     at java.base/sun.nio.fs.WindowsException.translateToIOException(WindowsException.java:85)

     at java.base/sun.nio.fs.WindowsException.rethrowAsIOException(WindowsException.java:103)

     at java.base/sun.nio.fs.WindowsException.rethrowAsIOException(WindowsException.java:108)

     at java.base/sun.nio.fs.WindowsFileAttributeViews$Basic.readAttributes(WindowsFileAttributeViews.java:53)

     at java.base/sun.nio.fs.WindowsFileAttributeViews$Basic.readAttributes(WindowsFileAttributeViews.java:38)

     at java.base/sun.nio.fs.WindowsFileSystemProvider.readAttributes(WindowsFileSystemProvider.java:198)

     at java.base/java.nio.file.Files.readAttributes(Files.java:1764)

     at java.base/java.util.zip.ZipFile$Source.get(ZipFile.java:1259)

     at java.base/java.util.zip.ZipFile$CleanableResource.(ZipFile.java:733)

     at java.base/java.util.zip.ZipFile$CleanableResource.get(ZipFile.java:850)

     at java.base/java.util.zip.ZipFile.(ZipFile.java:248)

     at java.base/java.util.zip.ZipFile.(ZipFile.java:177)

     at java.base/java.util.zip.ZipFile.(ZipFile.java:191)

     at fr.univ_nantes.codex_processor.core.measure.MeasureProcessor.run(MeasureProcessor.java:43)

     at fr.univ_nantes.codex_processor.gui.DatasetMeasureProcessor.run(DatasetMeasureProcessor.java:45)

     at fr.univ_nantes.codex_processor.gui.MainController$1.handle(MainController.java:160)

     at fr.univ_nantes.codex_processor.gui.MainController$1.handle(MainController.java:64)

     at com.sun.javafx.event.CompositeEventHandler.dispatchBubblingEvent(CompositeEventHandler.java:86)

     at com.sun.javafx.event.EventHandlerManager.dispatchBubblingEvent(EventHandlerManager.java:234)

     at com.sun.javafx.event.EventHandlerManager.dispatchBubblingEvent(EventHandlerManager.java:191)

     at com.sun.javafx.event.CompositeEventDispatcher.dispatchBubblingEvent(CompositeEventDispatcher.java:59)

     at com.sun.javafx.event.BasicEventDispatcher.dispatchEvent(BasicEventDispatcher.java:58)

     at com.sun.javafx.event.EventDispatchChainImpl.dispatchEvent(EventDispatchChainImpl.java:114)

     at com.sun.javafx.event.BasicEventDispatcher.dispatchEvent(BasicEventDispatcher.java:56)

     at com.sun.javafx.event.EventDispatchChainImpl.dispatchEvent(EventDispatchChainImpl.java:114)

     at com.sun.javafx.event.BasicEventDispatcher.dispatchEvent(BasicEventDispatcher.java:56)

     at com.sun.javafx.event.EventDispatchChainImpl.dispatchEvent(EventDispatchChainImpl.java:114)

     at com.sun.javafx.event.EventUtil.fireEventImpl(EventUtil.java:74)

     at com.sun.javafx.event.EventUtil.fireEvent(EventUtil.java:49)

     at javafx.event.Event.fireEvent(Event.java:198)

     at javafx.scene.Node.fireEvent(Node.java:8889)

     at javafx.scene.control.Button.fire(Button.java:203)

     at com.sun.javafx.scene.control.behavior.ButtonBehavior.mouseReleased(ButtonBehavior.java:208)

     at com.sun.javafx.scene.control.inputmap.InputMap.handle(InputMap.java:274)

     at com.sun.javafx.event.CompositeEventHandler$NormalEventHandlerRecord.handleBubblingEvent(CompositeEventHandler.java:247)

     at com.sun.javafx.event.CompositeEventHandler.dispatchBubblingEvent(CompositeEventHandler.java:80)

     at com.sun.javafx.event.EventHandlerManager.dispatchBubblingEvent(EventHandlerManager.java:234)

     at com.sun.javafx.event.EventHandlerManager.dispatchBubblingEvent(EventHandlerManager.java:191)

     at com.sun.javafx.event.CompositeEventDispatcher.dispatchBubblingEvent(CompositeEventDispatcher.java:59)

     at com.sun.javafx.event.BasicEventDispatcher.dispatchEvent(BasicEventDispatcher.java:58)

     at com.sun.javafx.event.EventDispatchChainImpl.dispatchEvent(EventDispatchChainImpl.java:114)

     at com.sun.javafx.event.BasicEventDispatcher.dispatchEvent(BasicEventDispatcher.java:56)

     at com.sun.javafx.event.EventDispatchChainImpl.dispatchEvent(EventDispatchChainImpl.java:114)

     at com.sun.javafx.event.BasicEventDispatcher.dispatchEvent(BasicEventDispatcher.java:56)

     at com.sun.javafx.event.EventDispatchChainImpl.dispatchEvent(EventDispatchChainImpl.java:114)

     at com.sun.javafx.event.EventUtil.fireEventImpl(EventUtil.java:74)

     at com.sun.javafx.event.EventUtil.fireEvent(EventUtil.java:54)

     at javafx.event.Event.fireEvent(Event.java:198)

     at javafx.scene.Scene$MouseHandler.process(Scene.java:3856)

     at javafx.scene.Scene.processMouseEvent(Scene.java:1851)

     at javafx.scene.Scene$ScenePeerListener.mouseEvent(Scene.java:2584)

     at com.sun.javafx.tk.quantum.GlassViewEventHandler$MouseEventNotification.run(GlassViewEventHandler.java:409)

     at com.sun.javafx.tk.quantum.GlassViewEventHandler$MouseEventNotification.run(GlassViewEventHandler.java:299)

     at java.base/java.security.AccessController.doPrivileged(Native Method)

     at com.sun.javafx.tk.quantum.GlassViewEventHandler.lambda$handleMouseEvent$2(GlassViewEventHandler.java:447)

     at com.sun.javafx.tk.quantum.QuantumToolkit.runWithoutRenderLock(QuantumToolkit.java:412)

     at com.sun.javafx.tk.quantum.GlassViewEventHandler.handleMouseEvent(GlassViewEventHandler.java:446)

     at com.sun.glass.ui.View.handleMouseEvent(View.java:556)

     at com.sun.glass.ui.View.notifyMouse(View.java:942)

     at com.sun.glass.ui.win.WinApplication._runLoop(Native Method)

     at com.sun.glass.ui.win.WinApplication.lambda$runLoop$3(WinApplication.java:174)

     at java.base/java.lang.Thread.run(Thread.java:829)

 java.nio.file.NoSuchFileException: C:\Users\IT-WL-annkl878\Documents\notes\Codex_Analysis\test_multiplxProcessor\out\rois_cy002_ch1.zip

     at java.base/sun.nio.fs.WindowsException.translateToIOException(WindowsException.java:85)

     at java.base/sun.nio.fs.WindowsException.rethrowAsIOException(WindowsException.java:103)

     at java.base/sun.nio.fs.WindowsException.rethrowAsIOException(WindowsException.java:108)

     at java.base/sun.nio.fs.WindowsFileAttributeViews$Basic.readAttributes(WindowsFileAttributeViews.java:53)

     at java.base/sun.nio.fs.WindowsFileAttributeViews$Basic.readAttributes(WindowsFileAttributeViews.java:38)

     at java.base/sun.nio.fs.WindowsFileSystemProvider.readAttributes(WindowsFileSystemProvider.java:198)

     at java.base/java.nio.file.Files.readAttributes(Files.java:1764)

     at java.base/java.util.zip.ZipFile$Source.get(ZipFile.java:1259)

     at java.base/java.util.zip.ZipFile$CleanableResource.(ZipFile.java:733)

     at java.base/java.util.zip.ZipFile$CleanableResource.get(ZipFile.java:850)

     at java.base/java.util.zip.ZipFile.(ZipFile.java:248)

     at java.base/java.util.zip.ZipFile.(ZipFile.java:177)

     at java.base/java.util.zip.ZipFile.(ZipFile.java:191)

     at fr.univ_nantes.codex_processor.core.measure.MeasureProcessor.run(MeasureProcessor.java:43)

     at fr.univ_nantes.codex_processor.gui.DatasetMeasureProcessor.run(DatasetMeasureProcessor.java:45)

     at fr.univ_nantes.codex_processor.gui.MainController$1.handle(MainController.java:160)

     at fr.univ_nantes.codex_processor.gui.MainController$1.handle(MainController.java:64)

     at com.sun.javafx.event.CompositeEventHandler.dispatchBubblingEvent(CompositeEventHandler.java:86)

     at com.sun.javafx.event.EventHandlerManager.dispatchBubblingEvent(EventHandlerManager.java:234)

     at com.sun.javafx.event.EventHandlerManager.dispatchBubblingEvent(EventHandlerManager.java:191)

     at com.sun.javafx.event.CompositeEventDispatcher.dispatchBubblingEvent(CompositeEventDispatcher.java:59)

     at com.sun.javafx.event.BasicEventDispatcher.dispatchEvent(BasicEventDispatcher.java:58)

     at com.sun.javafx.event.EventDispatchChainImpl.dispatchEvent(EventDispatchChainImpl.java:114)

     at com.sun.javafx.event.BasicEventDispatcher.dispatchEvent(BasicEventDispatcher.java:56)

     at com.sun.javafx.event.EventDispatchChainImpl.dispatchEvent(EventDispatchChainImpl.java:114)

     at com.sun.javafx.event.BasicEventDispatcher.dispatchEvent(BasicEventDispatcher.java:56)

     at com.sun.javafx.event.EventDispatchChainImpl.dispatchEvent(EventDispatchChainImpl.java:114)

     at com.sun.javafx.event.EventUtil.fireEventImpl(EventUtil.java:74)

     at com.sun.javafx.event.EventUtil.fireEvent(EventUtil.java:49)

     at javafx.event.Event.fireEvent(Event.java:198)

     at javafx.scene.Node.fireEvent(Node.java:8889)

     at javafx.scene.control.Button.fire(Button.java:203)

     at com.sun.javafx.scene.control.behavior.ButtonBehavior.mouseReleased(ButtonBehavior.java:208)

     at com.sun.javafx.scene.control.inputmap.InputMap.handle(InputMap.java:274)

     at com.sun.javafx.event.CompositeEventHandler$NormalEventHandlerRecord.handleBubblingEvent(CompositeEventHandler.java:247)

     at com.sun.javafx.event.CompositeEventHandler.dispatchBubblingEvent(CompositeEventHandler.java:80)

     at com.sun.javafx.event.EventHandlerManager.dispatchBubblingEvent(EventHandlerManager.java:234)

     at com.sun.javafx.event.EventHandlerManager.dispatchBubblingEvent(EventHandlerManager.java:191)

     at com.sun.javafx.event.CompositeEventDispatcher.dispatchBubblingEvent(CompositeEventDispatcher.java:59)

     at com.sun.javafx.event.BasicEventDispatcher.dispatchEvent(BasicEventDispatcher.java:58)

     at com.sun.javafx.event.EventDispatchChainImpl.dispatchEvent(EventDispatchChainImpl.java:114)

     at com.sun.javafx.event.BasicEventDispatcher.dispatchEvent(BasicEventDispatcher.java:56)

     at com.sun.javafx.event.EventDispatchChainImpl.dispatchEvent(EventDispatchChainImpl.java:114)

     at com.sun.javafx.event.BasicEventDispatcher.dispatchEvent(BasicEventDispatcher.java:56)

     at com.sun.javafx.event.EventDispatchChainImpl.dispatchEvent(EventDispatchChainImpl.java:114)

     at com.sun.javafx.event.EventUtil.fireEventImpl(EventUtil.java:74)

     at com.sun.javafx.event.EventUtil.fireEvent(EventUtil.java:54)

     at javafx.event.Event.fireEvent(Event.java:198)

     at javafx.scene.Scene$MouseHandler.process(Scene.java:3856)

     at javafx.scene.Scene.processMouseEvent(Scene.java:1851)

     at javafx.scene.Scene$ScenePeerListener.mouseEvent(Scene.java:2584)

     at com.sun.javafx.tk.quantum.GlassViewEventHandler$MouseEventNotification.run(GlassViewEventHandler.java:409)

     at com.sun.javafx.tk.quantum.GlassViewEventHandler$MouseEventNotification.run(GlassViewEventHandler.java:299)

     at java.base/java.security.AccessController.doPrivileged(Native Method)

     at com.sun.javafx.tk.quantum.GlassViewEventHandler.lambda$handleMouseEvent$2(GlassViewEventHandler.java:447)

     at com.sun.javafx.tk.quantum.QuantumToolkit.runWithoutRenderLock(QuantumToolkit.java:412)

     at com.sun.javafx.tk.quantum.GlassViewEventHandler.handleMouseEvent(GlassViewEventHandler.java:446)

     at com.sun.glass.ui.View.handleMouseEvent(View.java:556)

     at com.sun.glass.ui.View.notifyMouse(View.java:942)

     at com.sun.glass.ui.win.WinApplication._runLoop(Native Method)

     at com.sun.glass.ui.win.WinApplication.lambda$runLoop$3(WinApplication.java:174)

     at java.base/java.lang.Thread.run(Thread.java:829)

 java.nio.file.NoSuchFileException: C:\Users\IT-WL-annkl878\Documents\notes\Codex_Analysis\test_multiplxProcessor\out\rois_cy002_ch1.zip

     at java.base/sun.nio.fs.WindowsException.translateToIOException(WindowsException.java:85)

     at java.base/sun.nio.fs.WindowsException.rethrowAsIOException(WindowsException.java:103)

     at java.base/sun.nio.fs.WindowsException.rethrowAsIOException(WindowsException.java:108)

     at java.base/sun.nio.fs.WindowsFileAttributeViews$Basic.readAttributes(WindowsFileAttributeViews.java:53)

     at java.base/sun.nio.fs.WindowsFileAttributeViews$Basic.readAttributes(WindowsFileAttributeViews.java:38)

     at java.base/sun.nio.fs.WindowsFileSystemProvider.readAttributes(WindowsFileSystemProvider.java:198)

     at java.base/java.nio.file.Files.readAttributes(Files.java:1764)

     at java.base/java.util.zip.ZipFile$Source.get(ZipFile.java:1259)

     at java.base/java.util.zip.ZipFile$CleanableResource.(ZipFile.java:733)

     at java.base/java.util.zip.ZipFile$CleanableResource.get(ZipFile.java:850)

     at java.base/java.util.zip.ZipFile.(ZipFile.java:248)

     at java.base/java.util.zip.ZipFile.(ZipFile.java:177)

     at java.base/java.util.zip.ZipFile.(ZipFile.java:191)

     at fr.univ_nantes.codex_processor.core.measure.MeasureProcessor.run(MeasureProcessor.java:43)

     at fr.univ_nantes.codex_processor.gui.DatasetMeasureProcessor.run(DatasetMeasureProcessor.java:45)

     at fr.univ_nantes.codex_processor.gui.MainController$1.handle(MainController.java:160)

     at fr.univ_nantes.codex_processor.gui.MainController$1.handle(MainController.java:64)

     at com.sun.javafx.event.CompositeEventHandler.dispatchBubblingEvent(CompositeEventHandler.java:86)

     at com.sun.javafx.event.EventHandlerManager.dispatchBubblingEvent(EventHandlerManager.java:234)

     at com.sun.javafx.event.EventHandlerManager.dispatchBubblingEvent(EventHandlerManager.java:191)

     at com.sun.javafx.event.CompositeEventDispatcher.dispatchBubblingEvent(CompositeEventDispatcher.java:59)

     at com.sun.javafx.event.BasicEventDispatcher.dispatchEvent(BasicEventDispatcher.java:58)

     at com.sun.javafx.event.EventDispatchChainImpl.dispatchEvent(EventDispatchChainImpl.java:114)

     at com.sun.javafx.event.BasicEventDispatcher.dispatchEvent(BasicEventDispatcher.java:56)

     at com.sun.javafx.event.EventDispatchChainImpl.dispatchEvent(EventDispatchChainImpl.java:114)

     at com.sun.javafx.event.BasicEventDispatcher.dispatchEvent(BasicEventDispatcher.java:56)

     at com.sun.javafx.event.EventDispatchChainImpl.dispatchEvent(EventDispatchChainImpl.java:114)

     at com.sun.javafx.event.EventUtil.fireEventImpl(EventUtil.java:74)

     at com.sun.javafx.event.EventUtil.fireEvent(EventUtil.java:49)

     at javafx.event.Event.fireEvent(Event.java:198)

     at javafx.scene.Node.fireEvent(Node.java:8889)

     at javafx.scene.control.Button.fire(Button.java:203)

     at com.sun.javafx.scene.control.behavior.ButtonBehavior.mouseReleased(ButtonBehavior.java:208)

     at com.sun.javafx.scene.control.inputmap.InputMap.handle(InputMap.java:274)

     at com.sun.javafx.event.CompositeEventHandler$NormalEventHandlerRecord.handleBubblingEvent(CompositeEventHandler.java:247)

     at com.sun.javafx.event.CompositeEventHandler.dispatchBubblingEvent(CompositeEventHandler.java:80)

     at com.sun.javafx.event.EventHandlerManager.dispatchBubblingEvent(EventHandlerManager.java:234)

     at com.sun.javafx.event.EventHandlerManager.dispatchBubblingEvent(EventHandlerManager.java:191)

     at com.sun.javafx.event.CompositeEventDispatcher.dispatchBubblingEvent(CompositeEventDispatcher.java:59)

     at com.sun.javafx.event.BasicEventDispatcher.dispatchEvent(BasicEventDispatcher.java:58)

     at com.sun.javafx.event.EventDispatchChainImpl.dispatchEvent(EventDispatchChainImpl.java:114)

     at com.sun.javafx.event.BasicEventDispatcher.dispatchEvent(BasicEventDispatcher.java:56)

     at com.sun.javafx.event.EventDispatchChainImpl.dispatchEvent(EventDispatchChainImpl.java:114)

     at com.sun.javafx.event.BasicEventDispatcher.dispatchEvent(BasicEventDispatcher.java:56)

     at com.sun.javafx.event.EventDispatchChainImpl.dispatchEvent(EventDispatchChainImpl.java:114)

     at com.sun.javafx.event.EventUtil.fireEventImpl(EventUtil.java:74)

     at com.sun.javafx.event.EventUtil.fireEvent(EventUtil.java:54)

     at javafx.event.Event.fireEvent(Event.java:198)

     at javafx.scene.Scene$MouseHandler.process(Scene.java:3856)

     at javafx.scene.Scene.processMouseEvent(Scene.java:1851)

     at javafx.scene.Scene$ScenePeerListener.mouseEvent(Scene.java:2584)

     at com.sun.javafx.tk.quantum.GlassViewEventHandler$MouseEventNotification.run(GlassViewEventHandler.java:409)

     at com.sun.javafx.tk.quantum.GlassViewEventHandler$MouseEventNotification.run(GlassViewEventHandler.java:299)

     at java.base/java.security.AccessController.doPrivileged(Native Method)

     at com.sun.javafx.tk.quantum.GlassViewEventHandler.lambda$handleMouseEvent$2(GlassViewEventHandler.java:447)

     at com.sun.javafx.tk.quantum.QuantumToolkit.runWithoutRenderLock(QuantumToolkit.java:412)

     at com.sun.javafx.tk.quantum.GlassViewEventHandler.handleMouseEvent(GlassViewEventHandler.java:446)

     at com.sun.glass.ui.View.handleMouseEvent(View.java:556)

     at com.sun.glass.ui.View.notifyMouse(View.java:942)

     at com.sun.glass.ui.win.WinApplication._runLoop(Native Method)

     at com.sun.glass.ui.win.WinApplication.lambda$runLoop$3(WinApplication.java:174)

     at java.base/java.lang.Thread.run(Thread.java:829)

 java.nio.file.NoSuchFileException: C:\Users\IT-WL-annkl878\Documents\notes\Codex_Analysis\test_multiplxProcessor\out\rois_cy002_ch1.zip

     at java.base/sun.nio.fs.WindowsException.translateToIOException(WindowsException.java:85)

     at java.base/sun.nio.fs.WindowsException.rethrowAsIOException(WindowsException.java:103)

     at java.base/sun.nio.fs.WindowsException.rethrowAsIOException(WindowsException.java:108)

     at java.base/sun.nio.fs.WindowsFileAttributeViews$Basic.readAttributes(WindowsFileAttributeViews.java:53)

     at java.base/sun.nio.fs.WindowsFileAttributeViews$Basic.readAttributes(WindowsFileAttributeViews.java:38)

     at java.base/sun.nio.fs.WindowsFileSystemProvider.readAttributes(WindowsFileSystemProvider.java:198)

     at java.base/java.nio.file.Files.readAttributes(Files.java:1764)

     at java.base/java.util.zip.ZipFile$Source.get(ZipFile.java:1259)

     at java.base/java.util.zip.ZipFile$CleanableResource.(ZipFile.java:733)

     at java.base/java.util.zip.ZipFile$CleanableResource.get(ZipFile.java:850)

     at java.base/java.util.zip.ZipFile.(ZipFile.java:248)

     at java.base/java.util.zip.ZipFile.(ZipFile.java:177)

     at java.base/java.util.zip.ZipFile.(ZipFile.java:191)

     at fr.univ_nantes.codex_processor.core.measure.MeasureProcessor.run(MeasureProcessor.java:43)

     at fr.univ_nantes.codex_processor.gui.DatasetMeasureProcessor.run(DatasetMeasureProcessor.java:45)

     at fr.univ_nantes.codex_processor.gui.MainController$1.handle(MainController.java:160)

     at fr.univ_nantes.codex_processor.gui.MainController$1.handle(MainController.java:64)

     at com.sun.javafx.event.CompositeEventHandler.dispatchBubblingEvent(CompositeEventHandler.java:86)

     at com.sun.javafx.event.EventHandlerManager.dispatchBubblingEvent(EventHandlerManager.java:234)

     at com.sun.javafx.event.EventHandlerManager.dispatchBubblingEvent(EventHandlerManager.java:191)

     at com.sun.javafx.event.CompositeEventDispatcher.dispatchBubblingEvent(CompositeEventDispatcher.java:59)

     at com.sun.javafx.event.BasicEventDispatcher.dispatchEvent(BasicEventDispatcher.java:58)

     at com.sun.javafx.event.EventDispatchChainImpl.dispatchEvent(EventDispatchChainImpl.java:114)

     at com.sun.javafx.event.BasicEventDispatcher.dispatchEvent(BasicEventDispatcher.java:56)

     at com.sun.javafx.event.EventDispatchChainImpl.dispatchEvent(EventDispatchChainImpl.java:114)

     at com.sun.javafx.event.BasicEventDispatcher.dispatchEvent(BasicEventDispatcher.java:56)

     at com.sun.javafx.event.EventDispatchChainImpl.dispatchEvent(EventDispatchChainImpl.java:114)

     at com.sun.javafx.event.EventUtil.fireEventImpl(EventUtil.java:74)

     at com.sun.javafx.event.EventUtil.fireEvent(EventUtil.java:49)

     at javafx.event.Event.fireEvent(Event.java:198)

     at javafx.scene.Node.fireEvent(Node.java:8889)

     at javafx.scene.control.Button.fire(Button.java:203)

     at com.sun.javafx.scene.control.behavior.ButtonBehavior.mouseReleased(ButtonBehavior.java:208)

     at com.sun.javafx.scene.control.inputmap.InputMap.handle(InputMap.java:274)

     at com.sun.javafx.event.CompositeEventHandler$NormalEventHandlerRecord.handleBubblingEvent(CompositeEventHandler.java:247)

     at com.sun.javafx.event.CompositeEventHandler.dispatchBubblingEvent(CompositeEventHandler.java:80)

     at com.sun.javafx.event.EventHandlerManager.dispatchBubblingEvent(EventHandlerManager.java:234)

     at com.sun.javafx.event.EventHandlerManager.dispatchBubblingEvent(EventHandlerManager.java:191)

     at com.sun.javafx.event.CompositeEventDispatcher.dispatchBubblingEvent(CompositeEventDispatcher.java:59)

     at com.sun.javafx.event.BasicEventDispatcher.dispatchEvent(BasicEventDispatcher.java:58)

     at com.sun.javafx.event.EventDispatchChainImpl.dispatchEvent(EventDispatchChainImpl.java:114)

     at com.sun.javafx.event.BasicEventDispatcher.dispatchEvent(BasicEventDispatcher.java:56)

     at com.sun.javafx.event.EventDispatchChainImpl.dispatchEvent(EventDispatchChainImpl.java:114)

     at com.sun.javafx.event.BasicEventDispatcher.dispatchEvent(BasicEventDispatcher.java:56)

     at com.sun.javafx.event.EventDispatchChainImpl.dispatchEvent(EventDispatchChainImpl.java:114)

     at com.sun.javafx.event.EventUtil.fireEventImpl(EventUtil.java:74)

     at com.sun.javafx.event.EventUtil.fireEvent(EventUtil.java:54)

     at javafx.event.Event.fireEvent(Event.java:198)

     at javafx.scene.Scene$MouseHandler.process(Scene.java:3856)

     at javafx.scene.Scene.processMouseEvent(Scene.java:1851)

     at javafx.scene.Scene$ScenePeerListener.mouseEvent(Scene.java:2584)

     at com.sun.javafx.tk.quantum.GlassViewEventHandler$MouseEventNotification.run(GlassViewEventHandler.java:409)

     at com.sun.javafx.tk.quantum.GlassViewEventHandler$MouseEventNotification.run(GlassViewEventHandler.java:299)

     at java.base/java.security.AccessController.doPrivileged(Native Method)

     at com.sun.javafx.tk.quantum.GlassViewEventHandler.lambda$handleMouseEvent$2(GlassViewEventHandler.java:447)

     at com.sun.javafx.tk.quantum.QuantumToolkit.runWithoutRenderLock(QuantumToolkit.java:412)

     at com.sun.javafx.tk.quantum.GlassViewEventHandler.handleMouseEvent(GlassViewEventHandler.java:446)

     at com.sun.glass.ui.View.handleMouseEvent(View.java:556)

     at com.sun.glass.ui.View.notifyMouse(View.java:942)

     at com.sun.glass.ui.win.WinApplication._runLoop(Native Method)

     at com.sun.glass.ui.win.WinApplication.lambda$runLoop$3(WinApplication.java:174)

     at java.base/java.lang.Thread.run(Thread.java:829)

 java.nio.file.NoSuchFileException: C:\Users\IT-WL-annkl878\Documents\notes\Codex_Analysis\test_multiplxProcessor\out\rois_cy002_ch1.zip

     at java.base/sun.nio.fs.WindowsException.translateToIOException(WindowsException.java:85)

     at java.base/sun.nio.fs.WindowsException.rethrowAsIOException(WindowsException.java:103)

     at java.base/sun.nio.fs.WindowsException.rethrowAsIOException(WindowsException.java:108)

     at java.base/sun.nio.fs.WindowsFileAttributeViews$Basic.readAttributes(WindowsFileAttributeViews.java:53)

     at java.base/sun.nio.fs.WindowsFileAttributeViews$Basic.readAttributes(WindowsFileAttributeViews.java:38)

     at java.base/sun.nio.fs.WindowsFileSystemProvider.readAttributes(WindowsFileSystemProvider.java:198)

     at java.base/java.nio.file.Files.readAttributes(Files.java:1764)

     at java.base/java.util.zip.ZipFile$Source.get(ZipFile.java:1259)

     at java.base/java.util.zip.ZipFile$CleanableResource.(ZipFile.java:733)

     at java.base/java.util.zip.ZipFile$CleanableResource.get(ZipFile.java:850)

     at java.base/java.util.zip.ZipFile.(ZipFile.java:248)

     at java.base/java.util.zip.ZipFile.(ZipFile.java:177)

     at java.base/java.util.zip.ZipFile.(ZipFile.java:191)

     at fr.univ_nantes.codex_processor.core.measure.MeasureProcessor.run(MeasureProcessor.java:43)

     at fr.univ_nantes.codex_processor.gui.DatasetMeasureProcessor.run(DatasetMeasureProcessor.java:45)

     at fr.univ_nantes.codex_processor.gui.MainController$1.handle(MainController.java:160)

     at fr.univ_nantes.codex_processor.gui.MainController$1.handle(MainController.java:64)

     at com.sun.javafx.event.CompositeEventHandler.dispatchBubblingEvent(CompositeEventHandler.java:86)

     at com.sun.javafx.event.EventHandlerManager.dispatchBubblingEvent(EventHandlerManager.java:234)

     at com.sun.javafx.event.EventHandlerManager.dispatchBubblingEvent(EventHandlerManager.java:191)

     at com.sun.javafx.event.CompositeEventDispatcher.dispatchBubblingEvent(CompositeEventDispatcher.java:59)

     at com.sun.javafx.event.BasicEventDispatcher.dispatchEvent(BasicEventDispatcher.java:58)

     at com.sun.javafx.event.EventDispatchChainImpl.dispatchEvent(EventDispatchChainImpl.java:114)

     at com.sun.javafx.event.BasicEventDispatcher.dispatchEvent(BasicEventDispatcher.java:56)

     at com.sun.javafx.event.EventDispatchChainImpl.dispatchEvent(EventDispatchChainImpl.java:114)

     at com.sun.javafx.event.BasicEventDispatcher.dispatchEvent(BasicEventDispatcher.java:56)

     at com.sun.javafx.event.EventDispatchChainImpl.dispatchEvent(EventDispatchChainImpl.java:114)

     at com.sun.javafx.event.EventUtil.fireEventImpl(EventUtil.java:74)

     at com.sun.javafx.event.EventUtil.fireEvent(EventUtil.java:49)

     at javafx.event.Event.fireEvent(Event.java:198)

     at javafx.scene.Node.fireEvent(Node.java:8889)

     at javafx.scene.control.Button.fire(Button.java:203)

     at com.sun.javafx.scene.control.behavior.ButtonBehavior.mouseReleased(ButtonBehavior.java:208)

     at com.sun.javafx.scene.control.inputmap.InputMap.handle(InputMap.java:274)

     at com.sun.javafx.event.CompositeEventHandler$NormalEventHandlerRecord.handleBubblingEvent(CompositeEventHandler.java:247)

     at com.sun.javafx.event.CompositeEventHandler.dispatchBubblingEvent(CompositeEventHandler.java:80)

     at com.sun.javafx.event.EventHandlerManager.dispatchBubblingEvent(EventHandlerManager.java:234)

     at com.sun.javafx.event.EventHandlerManager.dispatchBubblingEvent(EventHandlerManager.java:191)

     at com.sun.javafx.event.CompositeEventDispatcher.dispatchBubblingEvent(CompositeEventDispatcher.java:59)

     at com.sun.javafx.event.BasicEventDispatcher.dispatchEvent(BasicEventDispatcher.java:58)

     at com.sun.javafx.event.EventDispatchChainImpl.dispatchEvent(EventDispatchChainImpl.java:114)

     at com.sun.javafx.event.BasicEventDispatcher.dispatchEvent(BasicEventDispatcher.java:56)

     at com.sun.javafx.event.EventDispatchChainImpl.dispatchEvent(EventDispatchChainImpl.java:114)

     at com.sun.javafx.event.BasicEventDispatcher.dispatchEvent(BasicEventDispatcher.java:56)

     at com.sun.javafx.event.EventDispatchChainImpl.dispatchEvent(EventDispatchChainImpl.java:114)

     at com.sun.javafx.event.EventUtil.fireEventImpl(EventUtil.java:74)

     at com.sun.javafx.event.EventUtil.fireEvent(EventUtil.java:54)

     at javafx.event.Event.fireEvent(Event.java:198)

     at javafx.scene.Scene$MouseHandler.process(Scene.java:3856)

     at javafx.scene.Scene.processMouseEvent(Scene.java:1851)

     at javafx.scene.Scene$ScenePeerListener.mouseEvent(Scene.java:2584)

     at com.sun.javafx.tk.quantum.GlassViewEventHandler$MouseEventNotification.run(GlassViewEventHandler.java:409)

     at com.sun.javafx.tk.quantum.GlassViewEventHandler$MouseEventNotification.run(GlassViewEventHandler.java:299)

     at java.base/java.security.AccessController.doPrivileged(Native Method)

     at com.sun.javafx.tk.quantum.GlassViewEventHandler.lambda$handleMouseEvent$2(GlassViewEventHandler.java:447)

     at com.sun.javafx.tk.quantum.QuantumToolkit.runWithoutRenderLock(QuantumToolkit.java:412)

     at com.sun.javafx.tk.quantum.GlassViewEventHandler.handleMouseEvent(GlassViewEventHandler.java:446)

     at com.sun.glass.ui.View.handleMouseEvent(View.java:556)

     at com.sun.glass.ui.View.notifyMouse(View.java:942)

     at com.sun.glass.ui.win.WinApplication._runLoop(Native Method)

     at com.sun.glass.ui.win.WinApplication.lambda$runLoop$3(WinApplication.java:174)

     at java.base/java.lang.Thread.run(Thread.java:829)

 java.nio.file.NoSuchFileException: C:\Users\IT-WL-annkl878\Documents\notes\Codex_Analysis\test_multiplxProcessor\out\rois_cy002_ch1.zip

     at java.base/sun.nio.fs.WindowsException.translateToIOException(WindowsException.java:85)

     at java.base/sun.nio.fs.WindowsException.rethrowAsIOException(WindowsException.java:103)

     at java.base/sun.nio.fs.WindowsException.rethrowAsIOException(WindowsException.java:108)

     at java.base/sun.nio.fs.WindowsFileAttributeViews$Basic.readAttributes(WindowsFileAttributeViews.java:53)

     at java.base/sun.nio.fs.WindowsFileAttributeViews$Basic.readAttributes(WindowsFileAttributeViews.java:38)

     at java.base/sun.nio.fs.WindowsFileSystemProvider.readAttributes(WindowsFileSystemProvider.java:198)

     at java.base/java.nio.file.Files.readAttributes(Files.java:1764)

     at java.base/java.util.zip.ZipFile$Source.get(ZipFile.java:1259)

     at java.base/java.util.zip.ZipFile$CleanableResource.(ZipFile.java:733)

     at java.base/java.util.zip.ZipFile$CleanableResource.get(ZipFile.java:850)

     at java.base/java.util.zip.ZipFile.(ZipFile.java:248)

     at java.base/java.util.zip.ZipFile.(ZipFile.java:177)

     at java.base/java.util.zip.ZipFile.(ZipFile.java:191)

     at fr.univ_nantes.codex_processor.core.measure.MeasureProcessor.run(MeasureProcessor.java:43)

     at fr.univ_nantes.codex_processor.gui.DatasetMeasureProcessor.run(DatasetMeasureProcessor.java:45)

     at fr.univ_nantes.codex_processor.gui.MainController$1.handle(MainController.java:160)

     at fr.univ_nantes.codex_processor.gui.MainController$1.handle(MainController.java:64)

     at com.sun.javafx.event.CompositeEventHandler.dispatchBubblingEvent(CompositeEventHandler.java:86)

     at com.sun.javafx.event.EventHandlerManager.dispatchBubblingEvent(EventHandlerManager.java:234)

     at com.sun.javafx.event.EventHandlerManager.dispatchBubblingEvent(EventHandlerManager.java:191)

     at com.sun.javafx.event.CompositeEventDispatcher.dispatchBubblingEvent(CompositeEventDispatcher.java:59)

     at com.sun.javafx.event.BasicEventDispatcher.dispatchEvent(BasicEventDispatcher.java:58)

     at com.sun.javafx.event.EventDispatchChainImpl.dispatchEvent(EventDispatchChainImpl.java:114)

     at com.sun.javafx.event.BasicEventDispatcher.dispatchEvent(BasicEventDispatcher.java:56)

     at com.sun.javafx.event.EventDispatchChainImpl.dispatchEvent(EventDispatchChainImpl.java:114)

     at com.sun.javafx.event.BasicEventDispatcher.dispatchEvent(BasicEventDispatcher.java:56)

     at com.sun.javafx.event.EventDispatchChainImpl.dispatchEvent(EventDispatchChainImpl.java:114)

     at com.sun.javafx.event.EventUtil.fireEventImpl(EventUtil.java:74)

     at com.sun.javafx.event.EventUtil.fireEvent(EventUtil.java:49)

     at javafx.event.Event.fireEvent(Event.java:198)

     at javafx.scene.Node.fireEvent(Node.java:8889)

     at javafx.scene.control.Button.fire(Button.java:203)

     at com.sun.javafx.scene.control.behavior.ButtonBehavior.mouseReleased(ButtonBehavior.java:208)

     at com.sun.javafx.scene.control.inputmap.InputMap.handle(InputMap.java:274)

     at com.sun.javafx.event.CompositeEventHandler$NormalEventHandlerRecord.handleBubblingEvent(CompositeEventHandler.java:247)

     at com.sun.javafx.event.CompositeEventHandler.dispatchBubblingEvent(CompositeEventHandler.java:80)

     at com.sun.javafx.event.EventHandlerManager.dispatchBubblingEvent(EventHandlerManager.java:234)

     at com.sun.javafx.event.EventHandlerManager.dispatchBubblingEvent(EventHandlerManager.java:191)

     at com.sun.javafx.event.CompositeEventDispatcher.dispatchBubblingEvent(CompositeEventDispatcher.java:59)

     at com.sun.javafx.event.BasicEventDispatcher.dispatchEvent(BasicEventDispatcher.java:58)

     at com.sun.javafx.event.EventDispatchChainImpl.dispatchEvent(EventDispatchChainImpl.java:114)

     at com.sun.javafx.event.BasicEventDispatcher.dispatchEvent(BasicEventDispatcher.java:56)

     at com.sun.javafx.event.EventDispatchChainImpl.dispatchEvent(EventDispatchChainImpl.java:114)

     at com.sun.javafx.event.BasicEventDispatcher.dispatchEvent(BasicEventDispatcher.java:56)

     at com.sun.javafx.event.EventDispatchChainImpl.dispatchEvent(EventDispatchChainImpl.java:114)

     at com.sun.javafx.event.EventUtil.fireEventImpl(EventUtil.java:74)

     at com.sun.javafx.event.EventUtil.fireEvent(EventUtil.java:54)

     at javafx.event.Event.fireEvent(Event.java:198)

     at javafx.scene.Scene$MouseHandler.process(Scene.java:3856)

     at javafx.scene.Scene.processMouseEvent(Scene.java:1851)

     at javafx.scene.Scene$ScenePeerListener.mouseEvent(Scene.java:2584)

     at com.sun.javafx.tk.quantum.GlassViewEventHandler$MouseEventNotification.run(GlassViewEventHandler.java:409)

     at com.sun.javafx.tk.quantum.GlassViewEventHandler$MouseEventNotification.run(GlassViewEventHandler.java:299)

     at java.base/java.security.AccessController.doPrivileged(Native Method)

     at com.sun.javafx.tk.quantum.GlassViewEventHandler.lambda$handleMouseEvent$2(GlassViewEventHandler.java:447)

     at com.sun.javafx.tk.quantum.QuantumToolkit.runWithoutRenderLock(QuantumToolkit.java:412)

     at com.sun.javafx.tk.quantum.GlassViewEventHandler.handleMouseEvent(GlassViewEventHandler.java:446)

     at com.sun.glass.ui.View.handleMouseEvent(View.java:556)

     at com.sun.glass.ui.View.notifyMouse(View.java:942)

     at com.sun.glass.ui.win.WinApplication._runLoop(Native Method)

     at com.sun.glass.ui.win.WinApplication.lambda$runLoop$3(WinApplication.java:174)

     at java.base/java.lang.Thread.run(Thread.java:829)

 java.nio.file.NoSuchFileException: C:\Users\IT-WL-annkl878\Documents\notes\Codex_Analysis\test_multiplxProcessor\out\rois_cy002_ch1.zip

     at java.base/sun.nio.fs.WindowsException.translateToIOException(WindowsException.java:85)

     at java.base/sun.nio.fs.WindowsException.rethrowAsIOException(WindowsException.java:103)

     at java.base/sun.nio.fs.WindowsException.rethrowAsIOException(WindowsException.java:108)

     at java.base/sun.nio.fs.WindowsFileAttributeViews$Basic.readAttributes(WindowsFileAttributeViews.java:53)

     at java.base/sun.nio.fs.WindowsFileAttributeViews$Basic.readAttributes(WindowsFileAttributeViews.java:38)

     at java.base/sun.nio.fs.WindowsFileSystemProvider.readAttributes(WindowsFileSystemProvider.java:198)

     at java.base/java.nio.file.Files.readAttributes(Files.java:1764)

     at java.base/java.util.zip.ZipFile$Source.get(ZipFile.java:1259)

     at java.base/java.util.zip.ZipFile$CleanableResource.(ZipFile.java:733)

     at java.base/java.util.zip.ZipFile$CleanableResource.get(ZipFile.java:850)

     at java.base/java.util.zip.ZipFile.(ZipFile.java:248)

     at java.base/java.util.zip.ZipFile.(ZipFile.java:177)

     at java.base/java.util.zip.ZipFile.(ZipFile.java:191)

     at fr.univ_nantes.codex_processor.core.measure.MeasureProcessor.run(MeasureProcessor.java:43)

     at fr.univ_nantes.codex_processor.gui.DatasetMeasureProcessor.run(DatasetMeasureProcessor.java:45)

     at fr.univ_nantes.codex_processor.gui.MainController$1.handle(MainController.java:160)

     at fr.univ_nantes.codex_processor.gui.MainController$1.handle(MainController.java:64)

     at com.sun.javafx.event.CompositeEventHandler.dispatchBubblingEvent(CompositeEventHandler.java:86)

     at com.sun.javafx.event.EventHandlerManager.dispatchBubblingEvent(EventHandlerManager.java:234)

     at com.sun.javafx.event.EventHandlerManager.dispatchBubblingEvent(EventHandlerManager.java:191)

     at com.sun.javafx.event.CompositeEventDispatcher.dispatchBubblingEvent(CompositeEventDispatcher.java:59)

     at com.sun.javafx.event.BasicEventDispatcher.dispatchEvent(BasicEventDispatcher.java:58)

     at com.sun.javafx.event.EventDispatchChainImpl.dispatchEvent(EventDispatchChainImpl.java:114)

     at com.sun.javafx.event.BasicEventDispatcher.dispatchEvent(BasicEventDispatcher.java:56)

     at com.sun.javafx.event.EventDispatchChainImpl.dispatchEvent(EventDispatchChainImpl.java:114)

     at com.sun.javafx.event.BasicEventDispatcher.dispatchEvent(BasicEventDispatcher.java:56)

     at com.sun.javafx.event.EventDispatchChainImpl.dispatchEvent(EventDispatchChainImpl.java:114)

     at com.sun.javafx.event.EventUtil.fireEventImpl(EventUtil.java:74)

     at com.sun.javafx.event.EventUtil.fireEvent(EventUtil.java:49)

     at javafx.event.Event.fireEvent(Event.java:198)

     at javafx.scene.Node.fireEvent(Node.java:8889)

     at javafx.scene.control.Button.fire(Button.java:203)

     at com.sun.javafx.scene.control.behavior.ButtonBehavior.mouseReleased(ButtonBehavior.java:208)

     at com.sun.javafx.scene.control.inputmap.InputMap.handle(InputMap.java:274)

     at com.sun.javafx.event.CompositeEventHandler$NormalEventHandlerRecord.handleBubblingEvent(CompositeEventHandler.java:247)

     at com.sun.javafx.event.CompositeEventHandler.dispatchBubblingEvent(CompositeEventHandler.java:80)

     at com.sun.javafx.event.EventHandlerManager.dispatchBubblingEvent(EventHandlerManager.java:234)

     at com.sun.javafx.event.EventHandlerManager.dispatchBubblingEvent(EventHandlerManager.java:191)

     at com.sun.javafx.event.CompositeEventDispatcher.dispatchBubblingEvent(CompositeEventDispatcher.java:59)

     at com.sun.javafx.event.BasicEventDispatcher.dispatchEvent(BasicEventDispatcher.java:58)

     at com.sun.javafx.event.EventDispatchChainImpl.dispatchEvent(EventDispatchChainImpl.java:114)

     at com.sun.javafx.event.BasicEventDispatcher.dispatchEvent(BasicEventDispatcher.java:56)

     at com.sun.javafx.event.EventDispatchChainImpl.dispatchEvent(EventDispatchChainImpl.java:114)

     at com.sun.javafx.event.BasicEventDispatcher.dispatchEvent(BasicEventDispatcher.java:56)

     at com.sun.javafx.event.EventDispatchChainImpl.dispatchEvent(EventDispatchChainImpl.java:114)

     at com.sun.javafx.event.EventUtil.fireEventImpl(EventUtil.java:74)

     at com.sun.javafx.event.EventUtil.fireEvent(EventUtil.java:54)

     at javafx.event.Event.fireEvent(Event.java:198)

     at javafx.scene.Scene$MouseHandler.process(Scene.java:3856)

     at javafx.scene.Scene.processMouseEvent(Scene.java:1851)

     at javafx.scene.Scene$ScenePeerListener.mouseEvent(Scene.java:2584)

     at com.sun.javafx.tk.quantum.GlassViewEventHandler$MouseEventNotification.run(GlassViewEventHandler.java:409)

     at com.sun.javafx.tk.quantum.GlassViewEventHandler$MouseEventNotification.run(GlassViewEventHandler.java:299)

     at java.base/java.security.AccessController.doPrivileged(Native Method)

     at com.sun.javafx.tk.quantum.GlassViewEventHandler.lambda$handleMouseEvent$2(GlassViewEventHandler.java:447)

     at com.sun.javafx.tk.quantum.QuantumToolkit.runWithoutRenderLock(QuantumToolkit.java:412)

     at com.sun.javafx.tk.quantum.GlassViewEventHandler.handleMouseEvent(GlassViewEventHandler.java:446)

     at com.sun.glass.ui.View.handleMouseEvent(View.java:556)

     at com.sun.glass.ui.View.notifyMouse(View.java:942)

     at com.sun.glass.ui.win.WinApplication._runLoop(Native Method)

     at com.sun.glass.ui.win.WinApplication.lambda$runLoop$3(WinApplication.java:174)

     at java.base/java.lang.Thread.run(Thread.java:829)

 java.nio.file.NoSuchFileException: C:\Users\IT-WL-annkl878\Documents\notes\Codex_Analysis\test_multiplxProcessor\out\rois_cy002_ch1.zip

     at java.base/sun.nio.fs.WindowsException.translateToIOException(WindowsException.java:85)

     at java.base/sun.nio.fs.WindowsException.rethrowAsIOException(WindowsException.java:103)

     at java.base/sun.nio.fs.WindowsException.rethrowAsIOException(WindowsException.java:108)

     at java.base/sun.nio.fs.WindowsFileAttributeViews$Basic.readAttributes(WindowsFileAttributeViews.java:53)

     at java.base/sun.nio.fs.WindowsFileAttributeViews$Basic.readAttributes(WindowsFileAttributeViews.java:38)

     at java.base/sun.nio.fs.WindowsFileSystemProvider.readAttributes(WindowsFileSystemProvider.java:198)

     at java.base/java.nio.file.Files.readAttributes(Files.java:1764)

     at java.base/java.util.zip.ZipFile$Source.get(ZipFile.java:1259)

     at java.base/java.util.zip.ZipFile$CleanableResource.(ZipFile.java:733)

     at java.base/java.util.zip.ZipFile$CleanableResource.get(ZipFile.java:850)

     at java.base/java.util.zip.ZipFile.(ZipFile.java:248)

     at java.base/java.util.zip.ZipFile.(ZipFile.java:177)

     at java.base/java.util.zip.ZipFile.(ZipFile.java:191)

     at fr.univ_nantes.codex_processor.core.measure.MeasureProcessor.run(MeasureProcessor.java:43)

     at fr.univ_nantes.codex_processor.gui.DatasetMeasureProcessor.run(DatasetMeasureProcessor.java:45)

     at fr.univ_nantes.codex_processor.gui.MainController$1.handle(MainController.java:160)

     at fr.univ_nantes.codex_processor.gui.MainController$1.handle(MainController.java:64)

     at com.sun.javafx.event.CompositeEventHandler.dispatchBubblingEvent(CompositeEventHandler.java:86)

     at com.sun.javafx.event.EventHandlerManager.dispatchBubblingEvent(EventHandlerManager.java:234)

     at com.sun.javafx.event.EventHandlerManager.dispatchBubblingEvent(EventHandlerManager.java:191)

     at com.sun.javafx.event.CompositeEventDispatcher.dispatchBubblingEvent(CompositeEventDispatcher.java:59)

     at com.sun.javafx.event.BasicEventDispatcher.dispatchEvent(BasicEventDispatcher.java:58)

     at com.sun.javafx.event.EventDispatchChainImpl.dispatchEvent(EventDispatchChainImpl.java:114)

     at com.sun.javafx.event.BasicEventDispatcher.dispatchEvent(BasicEventDispatcher.java:56)

     at com.sun.javafx.event.EventDispatchChainImpl.dispatchEvent(EventDispatchChainImpl.java:114)

     at com.sun.javafx.event.BasicEventDispatcher.dispatchEvent(BasicEventDispatcher.java:56)

     at com.sun.javafx.event.EventDispatchChainImpl.dispatchEvent(EventDispatchChainImpl.java:114)

     at com.sun.javafx.event.EventUtil.fireEventImpl(EventUtil.java:74)

     at com.sun.javafx.event.EventUtil.fireEvent(EventUtil.java:49)

     at javafx.event.Event.fireEvent(Event.java:198)

     at javafx.scene.Node.fireEvent(Node.java:8889)

     at javafx.scene.control.Button.fire(Button.java:203)

     at com.sun.javafx.scene.control.behavior.ButtonBehavior.mouseReleased(ButtonBehavior.java:208)

     at com.sun.javafx.scene.control.inputmap.InputMap.handle(InputMap.java:274)

     at com.sun.javafx.event.CompositeEventHandler$NormalEventHandlerRecord.handleBubblingEvent(CompositeEventHandler.java:247)

     at com.sun.javafx.event.CompositeEventHandler.dispatchBubblingEvent(CompositeEventHandler.java:80)

     at com.sun.javafx.event.EventHandlerManager.dispatchBubblingEvent(EventHandlerManager.java:234)

     at com.sun.javafx.event.EventHandlerManager.dispatchBubblingEvent(EventHandlerManager.java:191)

     at com.sun.javafx.event.CompositeEventDispatcher.dispatchBubblingEvent(CompositeEventDispatcher.java:59)

     at com.sun.javafx.event.BasicEventDispatcher.dispatchEvent(BasicEventDispatcher.java:58)

     at com.sun.javafx.event.EventDispatchChainImpl.dispatchEvent(EventDispatchChainImpl.java:114)

     at com.sun.javafx.event.BasicEventDispatcher.dispatchEvent(BasicEventDispatcher.java:56)

     at com.sun.javafx.event.EventDispatchChainImpl.dispatchEvent(EventDispatchChainImpl.java:114)

     at com.sun.javafx.event.BasicEventDispatcher.dispatchEvent(BasicEventDispatcher.java:56)

     at com.sun.javafx.event.EventDispatchChainImpl.dispatchEvent(EventDispatchChainImpl.java:114)

     at com.sun.javafx.event.EventUtil.fireEventImpl(EventUtil.java:74)

     at com.sun.javafx.event.EventUtil.fireEvent(EventUtil.java:54)

     at javafx.event.Event.fireEvent(Event.java:198)

     at javafx.scene.Scene$MouseHandler.process(Scene.java:3856)

     at javafx.scene.Scene.processMouseEvent(Scene.java:1851)

     at javafx.scene.Scene$ScenePeerListener.mouseEvent(Scene.java:2584)

     at com.sun.javafx.tk.quantum.GlassViewEventHandler$MouseEventNotification.run(GlassViewEventHandler.java:409)

     at com.sun.javafx.tk.quantum.GlassViewEventHandler$MouseEventNotification.run(GlassViewEventHandler.java:299)

     at java.base/java.security.AccessController.doPrivileged(Native Method)

     at com.sun.javafx.tk.quantum.GlassViewEventHandler.lambda$handleMouseEvent$2(GlassViewEventHandler.java:447)

     at com.sun.javafx.tk.quantum.QuantumToolkit.runWithoutRenderLock(QuantumToolkit.java:412)

     at com.sun.javafx.tk.quantum.GlassViewEventHandler.handleMouseEvent(GlassViewEventHandler.java:446)

     at com.sun.glass.ui.View.handleMouseEvent(View.java:556)

     at com.sun.glass.ui.View.notifyMouse(View.java:942)

     at com.sun.glass.ui.win.WinApplication._runLoop(Native Method)

     at com.sun.glass.ui.win.WinApplication.lambda$runLoop$3(WinApplication.java:174)

     at java.base/java.lang.Thread.run(Thread.java:829)

 java.nio.file.NoSuchFileException: C:\Users\IT-WL-annkl878\Documents\notes\Codex_Analysis\test_multiplxProcessor\out\rois_cy002_ch1.zip

     at java.base/sun.nio.fs.WindowsException.translateToIOException(WindowsException.java:85)

     at java.base/sun.nio.fs.WindowsException.rethrowAsIOException(WindowsException.java:103)

     at java.base/sun.nio.fs.WindowsException.rethrowAsIOException(WindowsException.java:108)

     at java.base/sun.nio.fs.WindowsFileAttributeViews$Basic.readAttributes(WindowsFileAttributeViews.java:53)

     at java.base/sun.nio.fs.WindowsFileAttributeViews$Basic.readAttributes(WindowsFileAttributeViews.java:38)

     at java.base/sun.nio.fs.WindowsFileSystemProvider.readAttributes(WindowsFileSystemProvider.java:198)

     at java.base/java.nio.file.Files.readAttributes(Files.java:1764)

     at java.base/java.util.zip.ZipFile$Source.get(ZipFile.java:1259)

     at java.base/java.util.zip.ZipFile$CleanableResource.(ZipFile.java:733)

     at java.base/java.util.zip.ZipFile$CleanableResource.get(ZipFile.java:850)

     at java.base/java.util.zip.ZipFile.(ZipFile.java:248)

     at java.base/java.util.zip.ZipFile.(ZipFile.java:177)

     at java.base/java.util.zip.ZipFile.(ZipFile.java:191)

     at fr.univ_nantes.codex_processor.core.measure.MeasureProcessor.run(MeasureProcessor.java:43)

     at fr.univ_nantes.codex_processor.gui.DatasetMeasureProcessor.run(DatasetMeasureProcessor.java:45)

     at fr.univ_nantes.codex_processor.gui.MainController$1.handle(MainController.java:160)

     at fr.univ_nantes.codex_processor.gui.MainController$1.handle(MainController.java:64)

     at com.sun.javafx.event.CompositeEventHandler.dispatchBubblingEvent(CompositeEventHandler.java:86)

     at com.sun.javafx.event.EventHandlerManager.dispatchBubblingEvent(EventHandlerManager.java:234)

     at com.sun.javafx.event.EventHandlerManager.dispatchBubblingEvent(EventHandlerManager.java:191)

     at com.sun.javafx.event.CompositeEventDispatcher.dispatchBubblingEvent(CompositeEventDispatcher.java:59)

     at com.sun.javafx.event.BasicEventDispatcher.dispatchEvent(BasicEventDispatcher.java:58)

     at com.sun.javafx.event.EventDispatchChainImpl.dispatchEvent(EventDispatchChainImpl.java:114)

     at com.sun.javafx.event.BasicEventDispatcher.dispatchEvent(BasicEventDispatcher.java:56)

     at com.sun.javafx.event.EventDispatchChainImpl.dispatchEvent(EventDispatchChainImpl.java:114)

     at com.sun.javafx.event.BasicEventDispatcher.dispatchEvent(BasicEventDispatcher.java:56)

     at com.sun.javafx.event.EventDispatchChainImpl.dispatchEvent(EventDispatchChainImpl.java:114)

     at com.sun.javafx.event.EventUtil.fireEventImpl(EventUtil.java:74)

     at com.sun.javafx.event.EventUtil.fireEvent(EventUtil.java:49)

     at javafx.event.Event.fireEvent(Event.java:198)

     at javafx.scene.Node.fireEvent(Node.java:8889)

     at javafx.scene.control.Button.fire(Button.java:203)

     at com.sun.javafx.scene.control.behavior.ButtonBehavior.mouseReleased(ButtonBehavior.java:208)

     at com.sun.javafx.scene.control.inputmap.InputMap.handle(InputMap.java:274)

     at com.sun.javafx.event.CompositeEventHandler$NormalEventHandlerRecord.handleBubblingEvent(CompositeEventHandler.java:247)

     at com.sun.javafx.event.CompositeEventHandler.dispatchBubblingEvent(CompositeEventHandler.java:80)

     at com.sun.javafx.event.EventHandlerManager.dispatchBubblingEvent(EventHandlerManager.java:234)

     at com.sun.javafx.event.EventHandlerManager.dispatchBubblingEvent(EventHandlerManager.java:191)

     at com.sun.javafx.event.CompositeEventDispatcher.dispatchBubblingEvent(CompositeEventDispatcher.java:59)

     at com.sun.javafx.event.BasicEventDispatcher.dispatchEvent(BasicEventDispatcher.java:58)

     at com.sun.javafx.event.EventDispatchChainImpl.dispatchEvent(EventDispatchChainImpl.java:114)

     at com.sun.javafx.event.BasicEventDispatcher.dispatchEvent(BasicEventDispatcher.java:56)

     at com.sun.javafx.event.EventDispatchChainImpl.dispatchEvent(EventDispatchChainImpl.java:114)

     at com.sun.javafx.event.BasicEventDispatcher.dispatchEvent(BasicEventDispatcher.java:56)

     at com.sun.javafx.event.EventDispatchChainImpl.dispatchEvent(EventDispatchChainImpl.java:114)

     at com.sun.javafx.event.EventUtil.fireEventImpl(EventUtil.java:74)

     at com.sun.javafx.event.EventUtil.fireEvent(EventUtil.java:54)

     at javafx.event.Event.fireEvent(Event.java:198)

     at javafx.scene.Scene$MouseHandler.process(Scene.java:3856)

     at javafx.scene.Scene.processMouseEvent(Scene.java:1851)

     at javafx.scene.Scene$ScenePeerListener.mouseEvent(Scene.java:2584)

     at com.sun.javafx.tk.quantum.GlassViewEventHandler$MouseEventNotification.run(GlassViewEventHandler.java:409)

     at com.sun.javafx.tk.quantum.GlassViewEventHandler$MouseEventNotification.run(GlassViewEventHandler.java:299)

     at java.base/java.security.AccessController.doPrivileged(Native Method)

     at com.sun.javafx.tk.quantum.GlassViewEventHandler.lambda$handleMouseEvent$2(GlassViewEventHandler.java:447)

     at com.sun.javafx.tk.quantum.QuantumToolkit.runWithoutRenderLock(QuantumToolkit.java:412)

     at com.sun.javafx.tk.quantum.GlassViewEventHandler.handleMouseEvent(GlassViewEventHandler.java:446)

     at com.sun.glass.ui.View.handleMouseEvent(View.java:556)

     at com.sun.glass.ui.View.notifyMouse(View.java:942)

     at com.sun.glass.ui.win.WinApplication._runLoop(Native Method)

     at com.sun.glass.ui.win.WinApplication.lambda$runLoop$3(WinApplication.java:174)

     at java.base/java.lang.Thread.run(Thread.java:829)

 java.nio.file.NoSuchFileException: C:\Users\IT-WL-annkl878\Documents\notes\Codex_Analysis\test_multiplxProcessor\out\rois_cy002_ch1.zip

     at java.base/sun.nio.fs.WindowsException.translateToIOException(WindowsException.java:85)

     at java.base/sun.nio.fs.WindowsException.rethrowAsIOException(WindowsException.java:103)

     at java.base/sun.nio.fs.WindowsException.rethrowAsIOException(WindowsException.java:108)

     at java.base/sun.nio.fs.WindowsFileAttributeViews$Basic.readAttributes(WindowsFileAttributeViews.java:53)

     at java.base/sun.nio.fs.WindowsFileAttributeViews$Basic.readAttributes(WindowsFileAttributeViews.java:38)

     at java.base/sun.nio.fs.WindowsFileSystemProvider.readAttributes(WindowsFileSystemProvider.java:198)

     at java.base/java.nio.file.Files.readAttributes(Files.java:1764)

     at java.base/java.util.zip.ZipFile$Source.get(ZipFile.java:1259)

     at java.base/java.util.zip.ZipFile$CleanableResource.(ZipFile.java:733)

     at java.base/java.util.zip.ZipFile$CleanableResource.get(ZipFile.java:850)

     at java.base/java.util.zip.ZipFile.(ZipFile.java:248)

     at java.base/java.util.zip.ZipFile.(ZipFile.java:177)

     at java.base/java.util.zip.ZipFile.(ZipFile.java:191)

     at fr.univ_nantes.codex_processor.core.measure.MeasureProcessor.run(MeasureProcessor.java:43)

     at fr.univ_nantes.codex_processor.gui.DatasetMeasureProcessor.run(DatasetMeasureProcessor.java:45)

     at fr.univ_nantes.codex_processor.gui.MainController$1.handle(MainController.java:160)

     at fr.univ_nantes.codex_processor.gui.MainController$1.handle(MainController.java:64)

     at com.sun.javafx.event.CompositeEventHandler.dispatchBubblingEvent(CompositeEventHandler.java:86)

     at com.sun.javafx.event.EventHandlerManager.dispatchBubblingEvent(EventHandlerManager.java:234)

     at com.sun.javafx.event.EventHandlerManager.dispatchBubblingEvent(EventHandlerManager.java:191)

     at com.sun.javafx.event.CompositeEventDispatcher.dispatchBubblingEvent(CompositeEventDispatcher.java:59)

     at com.sun.javafx.event.BasicEventDispatcher.dispatchEvent(BasicEventDispatcher.java:58)

     at com.sun.javafx.event.EventDispatchChainImpl.dispatchEvent(EventDispatchChainImpl.java:114)

     at com.sun.javafx.event.BasicEventDispatcher.dispatchEvent(BasicEventDispatcher.java:56)

     at com.sun.javafx.event.EventDispatchChainImpl.dispatchEvent(EventDispatchChainImpl.java:114)

     at com.sun.javafx.event.BasicEventDispatcher.dispatchEvent(BasicEventDispatcher.java:56)

     at com.sun.javafx.event.EventDispatchChainImpl.dispatchEvent(EventDispatchChainImpl.java:114)

     at com.sun.javafx.event.EventUtil.fireEventImpl(EventUtil.java:74)

     at com.sun.javafx.event.EventUtil.fireEvent(EventUtil.java:49)

     at javafx.event.Event.fireEvent(Event.java:198)

     at javafx.scene.Node.fireEvent(Node.java:8889)

     at javafx.scene.control.Button.fire(Button.java:203)

     at com.sun.javafx.scene.control.behavior.ButtonBehavior.mouseReleased(ButtonBehavior.java:208)

     at com.sun.javafx.scene.control.inputmap.InputMap.handle(InputMap.java:274)

     at com.sun.javafx.event.CompositeEventHandler$NormalEventHandlerRecord.handleBubblingEvent(CompositeEventHandler.java:247)

     at com.sun.javafx.event.CompositeEventHandler.dispatchBubblingEvent(CompositeEventHandler.java:80)

     at com.sun.javafx.event.EventHandlerManager.dispatchBubblingEvent(EventHandlerManager.java:234)

     at com.sun.javafx.event.EventHandlerManager.dispatchBubblingEvent(EventHandlerManager.java:191)

     at com.sun.javafx.event.CompositeEventDispatcher.dispatchBubblingEvent(CompositeEventDispatcher.java:59)

     at com.sun.javafx.event.BasicEventDispatcher.dispatchEvent(BasicEventDispatcher.java:58)

     at com.sun.javafx.event.EventDispatchChainImpl.dispatchEvent(EventDispatchChainImpl.java:114)

     at com.sun.javafx.event.BasicEventDispatcher.dispatchEvent(BasicEventDispatcher.java:56)

     at com.sun.javafx.event.EventDispatchChainImpl.dispatchEvent(EventDispatchChainImpl.java:114)

     at com.sun.javafx.event.BasicEventDispatcher.dispatchEvent(BasicEventDispatcher.java:56)

     at com.sun.javafx.event.EventDispatchChainImpl.dispatchEvent(EventDispatchChainImpl.java:114)

     at com.sun.javafx.event.EventUtil.fireEventImpl(EventUtil.java:74)

     at com.sun.javafx.event.EventUtil.fireEvent(EventUtil.java:54)

     at javafx.event.Event.fireEvent(Event.java:198)

     at javafx.scene.Scene$MouseHandler.process(Scene.java:3856)

     at javafx.scene.Scene.processMouseEvent(Scene.java:1851)

     at javafx.scene.Scene$ScenePeerListener.mouseEvent(Scene.java:2584)

     at com.sun.javafx.tk.quantum.GlassViewEventHandler$MouseEventNotification.run(GlassViewEventHandler.java:409)

     at com.sun.javafx.tk.quantum.GlassViewEventHandler$MouseEventNotification.run(GlassViewEventHandler.java:299)

     at java.base/java.security.AccessController.doPrivileged(Native Method)

     at com.sun.javafx.tk.quantum.GlassViewEventHandler.lambda$handleMouseEvent$2(GlassViewEventHandler.java:447)

     at com.sun.javafx.tk.quantum.QuantumToolkit.runWithoutRenderLock(QuantumToolkit.java:412)

     at com.sun.javafx.tk.quantum.GlassViewEventHandler.handleMouseEvent(GlassViewEventHandler.java:446)

     at com.sun.glass.ui.View.handleMouseEvent(View.java:556)

     at com.sun.glass.ui.View.notifyMouse(View.java:942)

     at com.sun.glass.ui.win.WinApplication._runLoop(Native Method)

     at com.sun.glass.ui.win.WinApplication.lambda$runLoop$3(WinApplication.java:174)

     at java.base/java.lang.Thread.run(Thread.java:829)

 java.nio.file.NoSuchFileException: C:\Users\IT-WL-annkl878\Documents\notes\Codex_Analysis\test_multiplxProcessor\out\rois_cy002_ch1.zip

     at java.base/sun.nio.fs.WindowsException.translateToIOException(WindowsException.java:85)

     at java.base/sun.nio.fs.WindowsException.rethrowAsIOException(WindowsException.java:103)

     at java.base/sun.nio.fs.WindowsException.rethrowAsIOException(WindowsException.java:108)

     at java.base/sun.nio.fs.WindowsFileAttributeViews$Basic.readAttributes(WindowsFileAttributeViews.java:53)

     at java.base/sun.nio.fs.WindowsFileAttributeViews$Basic.readAttributes(WindowsFileAttributeViews.java:38)

     at java.base/sun.nio.fs.WindowsFileSystemProvider.readAttributes(WindowsFileSystemProvider.java:198)

     at java.base/java.nio.file.Files.readAttributes(Files.java:1764)

     at java.base/java.util.zip.ZipFile$Source.get(ZipFile.java:1259)

     at java.base/java.util.zip.ZipFile$CleanableResource.(ZipFile.java:733)

     at java.base/java.util.zip.ZipFile$CleanableResource.get(ZipFile.java:850)

     at java.base/java.util.zip.ZipFile.(ZipFile.java:248)

     at java.base/java.util.zip.ZipFile.(ZipFile.java:177)

     at java.base/java.util.zip.ZipFile.(ZipFile.java:191)

     at fr.univ_nantes.codex_processor.core.measure.MeasureProcessor.run(MeasureProcessor.java:43)

     at fr.univ_nantes.codex_processor.gui.DatasetMeasureProcessor.run(DatasetMeasureProcessor.java:45)

     at fr.univ_nantes.codex_processor.gui.MainController$1.handle(MainController.java:160)

     at fr.univ_nantes.codex_processor.gui.MainController$1.handle(MainController.java:64)

     at com.sun.javafx.event.CompositeEventHandler.dispatchBubblingEvent(CompositeEventHandler.java:86)

     at com.sun.javafx.event.EventHandlerManager.dispatchBubblingEvent(EventHandlerManager.java:234)

     at com.sun.javafx.event.EventHandlerManager.dispatchBubblingEvent(EventHandlerManager.java:191)

     at com.sun.javafx.event.CompositeEventDispatcher.dispatchBubblingEvent(CompositeEventDispatcher.java:59)

     at com.sun.javafx.event.BasicEventDispatcher.dispatchEvent(BasicEventDispatcher.java:58)

     at com.sun.javafx.event.EventDispatchChainImpl.dispatchEvent(EventDispatchChainImpl.java:114)

     at com.sun.javafx.event.BasicEventDispatcher.dispatchEvent(BasicEventDispatcher.java:56)

     at com.sun.javafx.event.EventDispatchChainImpl.dispatchEvent(EventDispatchChainImpl.java:114)

     at com.sun.javafx.event.BasicEventDispatcher.dispatchEvent(BasicEventDispatcher.java:56)

     at com.sun.javafx.event.EventDispatchChainImpl.dispatchEvent(EventDispatchChainImpl.java:114)

     at com.sun.javafx.event.EventUtil.fireEventImpl(EventUtil.java:74)

     at com.sun.javafx.event.EventUtil.fireEvent(EventUtil.java:49)

     at javafx.event.Event.fireEvent(Event.java:198)

     at javafx.scene.Node.fireEvent(Node.java:8889)

     at javafx.scene.control.Button.fire(Button.java:203)

     at com.sun.javafx.scene.control.behavior.ButtonBehavior.mouseReleased(ButtonBehavior.java:208)

     at com.sun.javafx.scene.control.inputmap.InputMap.handle(InputMap.java:274)

     at com.sun.javafx.event.CompositeEventHandler$NormalEventHandlerRecord.handleBubblingEvent(CompositeEventHandler.java:247)

     at com.sun.javafx.event.CompositeEventHandler.dispatchBubblingEvent(CompositeEventHandler.java:80)

     at com.sun.javafx.event.EventHandlerManager.dispatchBubblingEvent(EventHandlerManager.java:234)

     at com.sun.javafx.event.EventHandlerManager.dispatchBubblingEvent(EventHandlerManager.java:191)

     at com.sun.javafx.event.CompositeEventDispatcher.dispatchBubblingEvent(CompositeEventDispatcher.java:59)

     at com.sun.javafx.event.BasicEventDispatcher.dispatchEvent(BasicEventDispatcher.java:58)

     at com.sun.javafx.event.EventDispatchChainImpl.dispatchEvent(EventDispatchChainImpl.java:114)

     at com.sun.javafx.event.BasicEventDispatcher.dispatchEvent(BasicEventDispatcher.java:56)

     at com.sun.javafx.event.EventDispatchChainImpl.dispatchEvent(EventDispatchChainImpl.java:114)

     at com.sun.javafx.event.BasicEventDispatcher.dispatchEvent(BasicEventDispatcher.java:56)

     at com.sun.javafx.event.EventDispatchChainImpl.dispatchEvent(EventDispatchChainImpl.java:114)

     at com.sun.javafx.event.EventUtil.fireEventImpl(EventUtil.java:74)

     at com.sun.javafx.event.EventUtil.fireEvent(EventUtil.java:54)

     at javafx.event.Event.fireEvent(Event.java:198)

     at javafx.scene.Scene$MouseHandler.process(Scene.java:3856)

     at javafx.scene.Scene.processMouseEvent(Scene.java:1851)

     at javafx.scene.Scene$ScenePeerListener.mouseEvent(Scene.java:2584)

     at com.sun.javafx.tk.quantum.GlassViewEventHandler$MouseEventNotification.run(GlassViewEventHandler.java:409)

     at com.sun.javafx.tk.quantum.GlassViewEventHandler$MouseEventNotification.run(GlassViewEventHandler.java:299)

     at java.base/java.security.AccessController.doPrivileged(Native Method)

     at com.sun.javafx.tk.quantum.GlassViewEventHandler.lambda$handleMouseEvent$2(GlassViewEventHandler.java:447)

     at com.sun.javafx.tk.quantum.QuantumToolkit.runWithoutRenderLock(QuantumToolkit.java:412)

     at com.sun.javafx.tk.quantum.GlassViewEventHandler.handleMouseEvent(GlassViewEventHandler.java:446)

     at com.sun.glass.ui.View.handleMouseEvent(View.java:556)

     at com.sun.glass.ui.View.notifyMouse(View.java:942)

     at com.sun.glass.ui.win.WinApplication._runLoop(Native Method)

     at com.sun.glass.ui.win.WinApplication.lambda$runLoop$3(WinApplication.java:174)

     at java.base/java.lang.Thread.run(Thread.java:829)

 java.nio.file.NoSuchFileException: C:\Users\IT-WL-annkl878\Documents\notes\Codex_Analysis\test_multiplxProcessor\out\rois_cy002_ch1.zip

     at java.base/sun.nio.fs.WindowsException.translateToIOException(WindowsException.java:85)

     at java.base/sun.nio.fs.WindowsException.rethrowAsIOException(WindowsException.java:103)

     at java.base/sun.nio.fs.WindowsException.rethrowAsIOException(WindowsException.java:108)

     at java.base/sun.nio.fs.WindowsFileAttributeViews$Basic.readAttributes(WindowsFileAttributeViews.java:53)

     at java.base/sun.nio.fs.WindowsFileAttributeViews$Basic.readAttributes(WindowsFileAttributeViews.java:38)

     at java.base/sun.nio.fs.WindowsFileSystemProvider.readAttributes(WindowsFileSystemProvider.java:198)

     at java.base/java.nio.file.Files.readAttributes(Files.java:1764)

     at java.base/java.util.zip.ZipFile$Source.get(ZipFile.java:1259)

     at java.base/java.util.zip.ZipFile$CleanableResource.(ZipFile.java:733)

     at java.base/java.util.zip.ZipFile$CleanableResource.get(ZipFile.java:850)

     at java.base/java.util.zip.ZipFile.(ZipFile.java:248)

     at java.base/java.util.zip.ZipFile.(ZipFile.java:177)

     at java.base/java.util.zip.ZipFile.(ZipFile.java:191)

     at fr.univ_nantes.codex_processor.core.measure.MeasureProcessor.run(MeasureProcessor.java:43)

     at fr.univ_nantes.codex_processor.gui.DatasetMeasureProcessor.run(DatasetMeasureProcessor.java:45)

     at fr.univ_nantes.codex_processor.gui.MainController$1.handle(MainController.java:160)

     at fr.univ_nantes.codex_processor.gui.MainController$1.handle(MainController.java:64)

     at com.sun.javafx.event.CompositeEventHandler.dispatchBubblingEvent(CompositeEventHandler.java:86)

     at com.sun.javafx.event.EventHandlerManager.dispatchBubblingEvent(EventHandlerManager.java:234)

     at com.sun.javafx.event.EventHandlerManager.dispatchBubblingEvent(EventHandlerManager.java:191)

     at com.sun.javafx.event.CompositeEventDispatcher.dispatchBubblingEvent(CompositeEventDispatcher.java:59)

     at com.sun.javafx.event.BasicEventDispatcher.dispatchEvent(BasicEventDispatcher.java:58)

     at com.sun.javafx.event.EventDispatchChainImpl.dispatchEvent(EventDispatchChainImpl.java:114)

     at com.sun.javafx.event.BasicEventDispatcher.dispatchEvent(BasicEventDispatcher.java:56)

     at com.sun.javafx.event.EventDispatchChainImpl.dispatchEvent(EventDispatchChainImpl.java:114)

     at com.sun.javafx.event.BasicEventDispatcher.dispatchEvent(BasicEventDispatcher.java:56)

     at com.sun.javafx.event.EventDispatchChainImpl.dispatchEvent(EventDispatchChainImpl.java:114)

     at com.sun.javafx.event.EventUtil.fireEventImpl(EventUtil.java:74)

     at com.sun.javafx.event.EventUtil.fireEvent(EventUtil.java:49)

     at javafx.event.Event.fireEvent(Event.java:198)

     at javafx.scene.Node.fireEvent(Node.java:8889)

     at javafx.scene.control.Button.fire(Button.java:203)

     at com.sun.javafx.scene.control.behavior.ButtonBehavior.mouseReleased(ButtonBehavior.java:208)

     at com.sun.javafx.scene.control.inputmap.InputMap.handle(InputMap.java:274)

     at com.sun.javafx.event.CompositeEventHandler$NormalEventHandlerRecord.handleBubblingEvent(CompositeEventHandler.java:247)

     at com.sun.javafx.event.CompositeEventHandler.dispatchBubblingEvent(CompositeEventHandler.java:80)

     at com.sun.javafx.event.EventHandlerManager.dispatchBubblingEvent(EventHandlerManager.java:234)

     at com.sun.javafx.event.EventHandlerManager.dispatchBubblingEvent(EventHandlerManager.java:191)

     at com.sun.javafx.event.CompositeEventDispatcher.dispatchBubblingEvent(CompositeEventDispatcher.java:59)

     at com.sun.javafx.event.BasicEventDispatcher.dispatchEvent(BasicEventDispatcher.java:58)

     at com.sun.javafx.event.EventDispatchChainImpl.dispatchEvent(EventDispatchChainImpl.java:114)

     at com.sun.javafx.event.BasicEventDispatcher.dispatchEvent(BasicEventDispatcher.java:56)

     at com.sun.javafx.event.EventDispatchChainImpl.dispatchEvent(EventDispatchChainImpl.java:114)

     at com.sun.javafx.event.BasicEventDispatcher.dispatchEvent(BasicEventDispatcher.java:56)

     at com.sun.javafx.event.EventDispatchChainImpl.dispatchEvent(EventDispatchChainImpl.java:114)

     at com.sun.javafx.event.EventUtil.fireEventImpl(EventUtil.java:74)

     at com.sun.javafx.event.EventUtil.fireEvent(EventUtil.java:54)

     at javafx.event.Event.fireEvent(Event.java:198)

     at javafx.scene.Scene$MouseHandler.process(Scene.java:3856)

     at javafx.scene.Scene.processMouseEvent(Scene.java:1851)

     at javafx.scene.Scene$ScenePeerListener.mouseEvent(Scene.java:2584)

     at com.sun.javafx.tk.quantum.GlassViewEventHandler$MouseEventNotification.run(GlassViewEventHandler.java:409)

     at com.sun.javafx.tk.quantum.GlassViewEventHandler$MouseEventNotification.run(GlassViewEventHandler.java:299)

     at java.base/java.security.AccessController.doPrivileged(Native Method)

     at com.sun.javafx.tk.quantum.GlassViewEventHandler.lambda$handleMouseEvent$2(GlassViewEventHandler.java:447)

     at com.sun.javafx.tk.quantum.QuantumToolkit.runWithoutRenderLock(QuantumToolkit.java:412)

     at com.sun.javafx.tk.quantum.GlassViewEventHandler.handleMouseEvent(GlassViewEventHandler.java:446)

     at com.sun.glass.ui.View.handleMouseEvent(View.java:556)

     at com.sun.glass.ui.View.notifyMouse(View.java:942)

     at com.sun.glass.ui.win.WinApplication._runLoop(Native Method)

     at com.sun.glass.ui.win.WinApplication.lambda$runLoop$3(WinApplication.java:174)

     at java.base/java.lang.Thread.run(Thread.java:829)

 java.nio.file.NoSuchFileException: C:\Users\IT-WL-annkl878\Documents\notes\Codex_Analysis\test_multiplxProcessor\out\rois_cy002_ch1.zip

     at java.base/sun.nio.fs.WindowsException.translateToIOException(WindowsException.java:85)

     at java.base/sun.nio.fs.WindowsException.rethrowAsIOException(WindowsException.java:103)

     at java.base/sun.nio.fs.WindowsException.rethrowAsIOException(WindowsException.java:108)

     at java.base/sun.nio.fs.WindowsFileAttributeViews$Basic.readAttributes(WindowsFileAttributeViews.java:53)

     at java.base/sun.nio.fs.WindowsFileAttributeViews$Basic.readAttributes(WindowsFileAttributeViews.java:38)

     at java.base/sun.nio.fs.WindowsFileSystemProvider.readAttributes(WindowsFileSystemProvider.java:198)

     at java.base/java.nio.file.Files.readAttributes(Files.java:1764)

     at java.base/java.util.zip.ZipFile$Source.get(ZipFile.java:1259)

     at java.base/java.util.zip.ZipFile$CleanableResource.(ZipFile.java:733)

     at java.base/java.util.zip.ZipFile$CleanableResource.get(ZipFile.java:850)

     at java.base/java.util.zip.ZipFile.(ZipFile.java:248)

     at java.base/java.util.zip.ZipFile.(ZipFile.java:177)

     at java.base/java.util.zip.ZipFile.(ZipFile.java:191)

     at fr.univ_nantes.codex_processor.core.measure.MeasureProcessor.run(MeasureProcessor.java:43)

     at fr.univ_nantes.codex_processor.gui.DatasetMeasureProcessor.run(DatasetMeasureProcessor.java:45)

     at fr.univ_nantes.codex_processor.gui.MainController$1.handle(MainController.java:160)

     at fr.univ_nantes.codex_processor.gui.MainController$1.handle(MainController.java:64)

     at com.sun.javafx.event.CompositeEventHandler.dispatchBubblingEvent(CompositeEventHandler.java:86)

     at com.sun.javafx.event.EventHandlerManager.dispatchBubblingEvent(EventHandlerManager.java:234)

     at com.sun.javafx.event.EventHandlerManager.dispatchBubblingEvent(EventHandlerManager.java:191)

     at com.sun.javafx.event.CompositeEventDispatcher.dispatchBubblingEvent(CompositeEventDispatcher.java:59)

     at com.sun.javafx.event.BasicEventDispatcher.dispatchEvent(BasicEventDispatcher.java:58)

     at com.sun.javafx.event.EventDispatchChainImpl.dispatchEvent(EventDispatchChainImpl.java:114)

     at com.sun.javafx.event.BasicEventDispatcher.dispatchEvent(BasicEventDispatcher.java:56)

     at com.sun.javafx.event.EventDispatchChainImpl.dispatchEvent(EventDispatchChainImpl.java:114)

     at com.sun.javafx.event.BasicEventDispatcher.dispatchEvent(BasicEventDispatcher.java:56)

     at com.sun.javafx.event.EventDispatchChainImpl.dispatchEvent(EventDispatchChainImpl.java:114)

     at com.sun.javafx.event.EventUtil.fireEventImpl(EventUtil.java:74)

     at com.sun.javafx.event.EventUtil.fireEvent(EventUtil.java:49)

     at javafx.event.Event.fireEvent(Event.java:198)

     at javafx.scene.Node.fireEvent(Node.java:8889)

     at javafx.scene.control.Button.fire(Button.java:203)

     at com.sun.javafx.scene.control.behavior.ButtonBehavior.mouseReleased(ButtonBehavior.java:208)

     at com.sun.javafx.scene.control.inputmap.InputMap.handle(InputMap.java:274)

     at com.sun.javafx.event.CompositeEventHandler$NormalEventHandlerRecord.handleBubblingEvent(CompositeEventHandler.java:247)

     at com.sun.javafx.event.CompositeEventHandler.dispatchBubblingEvent(CompositeEventHandler.java:80)

     at com.sun.javafx.event.EventHandlerManager.dispatchBubblingEvent(EventHandlerManager.java:234)

     at com.sun.javafx.event.EventHandlerManager.dispatchBubblingEvent(EventHandlerManager.java:191)

     at com.sun.javafx.event.CompositeEventDispatcher.dispatchBubblingEvent(CompositeEventDispatcher.java:59)

     at com.sun.javafx.event.BasicEventDispatcher.dispatchEvent(BasicEventDispatcher.java:58)

     at com.sun.javafx.event.EventDispatchChainImpl.dispatchEvent(EventDispatchChainImpl.java:114)

     at com.sun.javafx.event.BasicEventDispatcher.dispatchEvent(BasicEventDispatcher.java:56)

     at com.sun.javafx.event.EventDispatchChainImpl.dispatchEvent(EventDispatchChainImpl.java:114)

     at com.sun.javafx.event.BasicEventDispatcher.dispatchEvent(BasicEventDispatcher.java:56)

     at com.sun.javafx.event.EventDispatchChainImpl.dispatchEvent(EventDispatchChainImpl.java:114)

     at com.sun.javafx.event.EventUtil.fireEventImpl(EventUtil.java:74)

     at com.sun.javafx.event.EventUtil.fireEvent(EventUtil.java:54)

     at javafx.event.Event.fireEvent(Event.java:198)

     at javafx.scene.Scene$MouseHandler.process(Scene.java:3856)

     at javafx.scene.Scene.processMouseEvent(Scene.java:1851)

     at javafx.scene.Scene$ScenePeerListener.mouseEvent(Scene.java:2584)

     at com.sun.javafx.tk.quantum.GlassViewEventHandler$MouseEventNotification.run(GlassViewEventHandler.java:409)

     at com.sun.javafx.tk.quantum.GlassViewEventHandler$MouseEventNotification.run(GlassViewEventHandler.java:299)

     at java.base/java.security.AccessController.doPrivileged(Native Method)

     at com.sun.javafx.tk.quantum.GlassViewEventHandler.lambda$handleMouseEvent$2(GlassViewEventHandler.java:447)

     at com.sun.javafx.tk.quantum.QuantumToolkit.runWithoutRenderLock(QuantumToolkit.java:412)

     at com.sun.javafx.tk.quantum.GlassViewEventHandler.handleMouseEvent(GlassViewEventHandler.java:446)

     at com.sun.glass.ui.View.handleMouseEvent(View.java:556)

     at com.sun.glass.ui.View.notifyMouse(View.java:942)

     at com.sun.glass.ui.win.WinApplication._runLoop(Native Method)

     at com.sun.glass.ui.win.WinApplication.lambda$runLoop$3(WinApplication.java:174)

     at java.base/java.lang.Thread.run(Thread.java:829)

 java.nio.file.NoSuchFileException: C:\Users\IT-WL-annkl878\Documents\notes\Codex_Analysis\test_multiplxProcessor\out\rois_cy002_ch1.zip

     at java.base/sun.nio.fs.WindowsException.translateToIOException(WindowsException.java:85)

     at java.base/sun.nio.fs.WindowsException.rethrowAsIOException(WindowsException.java:103)

     at java.base/sun.nio.fs.WindowsException.rethrowAsIOException(WindowsException.java:108)

     at java.base/sun.nio.fs.WindowsFileAttributeViews$Basic.readAttributes(WindowsFileAttributeViews.java:53)

     at java.base/sun.nio.fs.WindowsFileAttributeViews$Basic.readAttributes(WindowsFileAttributeViews.java:38)

     at java.base/sun.nio.fs.WindowsFileSystemProvider.readAttributes(WindowsFileSystemProvider.java:198)

     at java.base/java.nio.file.Files.readAttributes(Files.java:1764)

     at java.base/java.util.zip.ZipFile$Source.get(ZipFile.java:1259)

     at java.base/java.util.zip.ZipFile$CleanableResource.(ZipFile.java:733)

     at java.base/java.util.zip.ZipFile$CleanableResource.get(ZipFile.java:850)

     at java.base/java.util.zip.ZipFile.(ZipFile.java:248)

     at java.base/java.util.zip.ZipFile.(ZipFile.java:177)

     at java.base/java.util.zip.ZipFile.(ZipFile.java:191)

     at fr.univ_nantes.codex_processor.core.measure.MeasureProcessor.run(MeasureProcessor.java:43)

     at fr.univ_nantes.codex_processor.gui.DatasetMeasureProcessor.run(DatasetMeasureProcessor.java:45)

     at fr.univ_nantes.codex_processor.gui.MainController$1.handle(MainController.java:160)

     at fr.univ_nantes.codex_processor.gui.MainController$1.handle(MainController.java:64)

     at com.sun.javafx.event.CompositeEventHandler.dispatchBubblingEvent(CompositeEventHandler.java:86)

     at com.sun.javafx.event.EventHandlerManager.dispatchBubblingEvent(EventHandlerManager.java:234)

     at com.sun.javafx.event.EventHandlerManager.dispatchBubblingEvent(EventHandlerManager.java:191)

     at com.sun.javafx.event.CompositeEventDispatcher.dispatchBubblingEvent(CompositeEventDispatcher.java:59)

     at com.sun.javafx.event.BasicEventDispatcher.dispatchEvent(BasicEventDispatcher.java:58)

     at com.sun.javafx.event.EventDispatchChainImpl.dispatchEvent(EventDispatchChainImpl.java:114)

     at com.sun.javafx.event.BasicEventDispatcher.dispatchEvent(BasicEventDispatcher.java:56)

     at com.sun.javafx.event.EventDispatchChainImpl.dispatchEvent(EventDispatchChainImpl.java:114)

     at com.sun.javafx.event.BasicEventDispatcher.dispatchEvent(BasicEventDispatcher.java:56)

     at com.sun.javafx.event.EventDispatchChainImpl.dispatchEvent(EventDispatchChainImpl.java:114)

     at com.sun.javafx.event.EventUtil.fireEventImpl(EventUtil.java:74)

     at com.sun.javafx.event.EventUtil.fireEvent(EventUtil.java:49)

     at javafx.event.Event.fireEvent(Event.java:198)

     at javafx.scene.Node.fireEvent(Node.java:8889)

     at javafx.scene.control.Button.fire(Button.java:203)

     at com.sun.javafx.scene.control.behavior.ButtonBehavior.mouseReleased(ButtonBehavior.java:208)

     at com.sun.javafx.scene.control.inputmap.InputMap.handle(InputMap.java:274)

     at com.sun.javafx.event.CompositeEventHandler$NormalEventHandlerRecord.handleBubblingEvent(CompositeEventHandler.java:247)

     at com.sun.javafx.event.CompositeEventHandler.dispatchBubblingEvent(CompositeEventHandler.java:80)

     at com.sun.javafx.event.EventHandlerManager.dispatchBubblingEvent(EventHandlerManager.java:234)

     at com.sun.javafx.event.EventHandlerManager.dispatchBubblingEvent(EventHandlerManager.java:191)

     at com.sun.javafx.event.CompositeEventDispatcher.dispatchBubblingEvent(CompositeEventDispatcher.java:59)

     at com.sun.javafx.event.BasicEventDispatcher.dispatchEvent(BasicEventDispatcher.java:58)

     at com.sun.javafx.event.EventDispatchChainImpl.dispatchEvent(EventDispatchChainImpl.java:114)

     at com.sun.javafx.event.BasicEventDispatcher.dispatchEvent(BasicEventDispatcher.java:56)

     at com.sun.javafx.event.EventDispatchChainImpl.dispatchEvent(EventDispatchChainImpl.java:114)

     at com.sun.javafx.event.BasicEventDispatcher.dispatchEvent(BasicEventDispatcher.java:56)

     at com.sun.javafx.event.EventDispatchChainImpl.dispatchEvent(EventDispatchChainImpl.java:114)

     at com.sun.javafx.event.EventUtil.fireEventImpl(EventUtil.java:74)

     at com.sun.javafx.event.EventUtil.fireEvent(EventUtil.java:54)

     at javafx.event.Event.fireEvent(Event.java:198)

     at javafx.scene.Scene$MouseHandler.process(Scene.java:3856)

     at javafx.scene.Scene.processMouseEvent(Scene.java:1851)

     at javafx.scene.Scene$ScenePeerListener.mouseEvent(Scene.java:2584)

     at com.sun.javafx.tk.quantum.GlassViewEventHandler$MouseEventNotification.run(GlassViewEventHandler.java:409)

     at com.sun.javafx.tk.quantum.GlassViewEventHandler$MouseEventNotification.run(GlassViewEventHandler.java:299)

     at java.base/java.security.AccessController.doPrivileged(Native Method)

     at com.sun.javafx.tk.quantum.GlassViewEventHandler.lambda$handleMouseEvent$2(GlassViewEventHandler.java:447)

     at com.sun.javafx.tk.quantum.QuantumToolkit.runWithoutRenderLock(QuantumToolkit.java:412)

     at com.sun.javafx.tk.quantum.GlassViewEventHandler.handleMouseEvent(GlassViewEventHandler.java:446)

     at com.sun.glass.ui.View.handleMouseEvent(View.java:556)

     at com.sun.glass.ui.View.notifyMouse(View.java:942)

     at com.sun.glass.ui.win.WinApplication._runLoop(Native Method)

     at com.sun.glass.ui.win.WinApplication.lambda$runLoop$3(WinApplication.java:174)

     at java.base/java.lang.Thread.run(Thread.java:829)

 java.nio.file.NoSuchFileException: C:\Users\IT-WL-annkl878\Documents\notes\Codex_Analysis\test_multiplxProcessor\out\rois_cy002_ch1.zip

     at java.base/sun.nio.fs.WindowsException.translateToIOException(WindowsException.java:85)

     at java.base/sun.nio.fs.WindowsException.rethrowAsIOException(WindowsException.java:103)

     at java.base/sun.nio.fs.WindowsException.rethrowAsIOException(WindowsException.java:108)

     at java.base/sun.nio.fs.WindowsFileAttributeViews$Basic.readAttributes(WindowsFileAttributeViews.java:53)

     at java.base/sun.nio.fs.WindowsFileAttributeViews$Basic.readAttributes(WindowsFileAttributeViews.java:38)

     at java.base/sun.nio.fs.WindowsFileSystemProvider.readAttributes(WindowsFileSystemProvider.java:198)

     at java.base/java.nio.file.Files.readAttributes(Files.java:1764)

     at java.base/java.util.zip.ZipFile$Source.get(ZipFile.java:1259)

     at java.base/java.util.zip.ZipFile$CleanableResource.(ZipFile.java:733)

     at java.base/java.util.zip.ZipFile$CleanableResource.get(ZipFile.java:850)

     at java.base/java.util.zip.ZipFile.(ZipFile.java:248)

     at java.base/java.util.zip.ZipFile.(ZipFile.java:177)

     at java.base/java.util.zip.ZipFile.(ZipFile.java:191)

     at fr.univ_nantes.codex_processor.core.measure.MeasureProcessor.run(MeasureProcessor.java:43)

     at fr.univ_nantes.codex_processor.gui.DatasetMeasureProcessor.run(DatasetMeasureProcessor.java:45)

     at fr.univ_nantes.codex_processor.gui.MainController$1.handle(MainController.java:160)

     at fr.univ_nantes.codex_processor.gui.MainController$1.handle(MainController.java:64)

     at com.sun.javafx.event.CompositeEventHandler.dispatchBubblingEvent(CompositeEventHandler.java:86)

     at com.sun.javafx.event.EventHandlerManager.dispatchBubblingEvent(EventHandlerManager.java:234)

     at com.sun.javafx.event.EventHandlerManager.dispatchBubblingEvent(EventHandlerManager.java:191)

     at com.sun.javafx.event.CompositeEventDispatcher.dispatchBubblingEvent(CompositeEventDispatcher.java:59)

     at com.sun.javafx.event.BasicEventDispatcher.dispatchEvent(BasicEventDispatcher.java:58)

     at com.sun.javafx.event.EventDispatchChainImpl.dispatchEvent(EventDispatchChainImpl.java:114)

     at com.sun.javafx.event.BasicEventDispatcher.dispatchEvent(BasicEventDispatcher.java:56)

     at com.sun.javafx.event.EventDispatchChainImpl.dispatchEvent(EventDispatchChainImpl.java:114)

     at com.sun.javafx.event.BasicEventDispatcher.dispatchEvent(BasicEventDispatcher.java:56)

     at com.sun.javafx.event.EventDispatchChainImpl.dispatchEvent(EventDispatchChainImpl.java:114)

     at com.sun.javafx.event.EventUtil.fireEventImpl(EventUtil.java:74)

     at com.sun.javafx.event.EventUtil.fireEvent(EventUtil.java:49)

     at javafx.event.Event.fireEvent(Event.java:198)

     at javafx.scene.Node.fireEvent(Node.java:8889)

     at javafx.scene.control.Button.fire(Button.java:203)

     at com.sun.javafx.scene.control.behavior.ButtonBehavior.mouseReleased(ButtonBehavior.java:208)

     at com.sun.javafx.scene.control.inputmap.InputMap.handle(InputMap.java:274)

     at com.sun.javafx.event.CompositeEventHandler$NormalEventHandlerRecord.handleBubblingEvent(CompositeEventHandler.java:247)

     at com.sun.javafx.event.CompositeEventHandler.dispatchBubblingEvent(CompositeEventHandler.java:80)

     at com.sun.javafx.event.EventHandlerManager.dispatchBubblingEvent(EventHandlerManager.java:234)

     at com.sun.javafx.event.EventHandlerManager.dispatchBubblingEvent(EventHandlerManager.java:191)

     at com.sun.javafx.event.CompositeEventDispatcher.dispatchBubblingEvent(CompositeEventDispatcher.java:59)

     at com.sun.javafx.event.BasicEventDispatcher.dispatchEvent(BasicEventDispatcher.java:58)

     at com.sun.javafx.event.EventDispatchChainImpl.dispatchEvent(EventDispatchChainImpl.java:114)

     at com.sun.javafx.event.BasicEventDispatcher.dispatchEvent(BasicEventDispatcher.java:56)

     at com.sun.javafx.event.EventDispatchChainImpl.dispatchEvent(EventDispatchChainImpl.java:114)

     at com.sun.javafx.event.BasicEventDispatcher.dispatchEvent(BasicEventDispatcher.java:56)

     at com.sun.javafx.event.EventDispatchChainImpl.dispatchEvent(EventDispatchChainImpl.java:114)

     at com.sun.javafx.event.EventUtil.fireEventImpl(EventUtil.java:74)

     at com.sun.javafx.event.EventUtil.fireEvent(EventUtil.java:54)

     at javafx.event.Event.fireEvent(Event.java:198)

     at javafx.scene.Scene$MouseHandler.process(Scene.java:3856)

     at javafx.scene.Scene.processMouseEvent(Scene.java:1851)

     at javafx.scene.Scene$ScenePeerListener.mouseEvent(Scene.java:2584)

     at com.sun.javafx.tk.quantum.GlassViewEventHandler$MouseEventNotification.run(GlassViewEventHandler.java:409)

     at com.sun.javafx.tk.quantum.GlassViewEventHandler$MouseEventNotification.run(GlassViewEventHandler.java:299)

     at java.base/java.security.AccessController.doPrivileged(Native Method)

     at com.sun.javafx.tk.quantum.GlassViewEventHandler.lambda$handleMouseEvent$2(GlassViewEventHandler.java:447)

     at com.sun.javafx.tk.quantum.QuantumToolkit.runWithoutRenderLock(QuantumToolkit.java:412)

     at com.sun.javafx.tk.quantum.GlassViewEventHandler.handleMouseEvent(GlassViewEventHandler.java:446)

     at com.sun.glass.ui.View.handleMouseEvent(View.java:556)

     at com.sun.glass.ui.View.notifyMouse(View.java:942)

     at com.sun.glass.ui.win.WinApplication._runLoop(Native Method)

     at com.sun.glass.ui.win.WinApplication.lambda$runLoop$3(WinApplication.java:174)

     at java.base/java.lang.Thread.run(Thread.java:829)

 java.nio.file.NoSuchFileException: C:\Users\IT-WL-annkl878\Documents\notes\Codex_Analysis\test_multiplxProcessor\out\rois_cy002_ch1.zip

     at java.base/sun.nio.fs.WindowsException.translateToIOException(WindowsException.java:85)

     at java.base/sun.nio.fs.WindowsException.rethrowAsIOException(WindowsException.java:103)

     at java.base/sun.nio.fs.WindowsException.rethrowAsIOException(WindowsException.java:108)

     at java.base/sun.nio.fs.WindowsFileAttributeViews$Basic.readAttributes(WindowsFileAttributeViews.java:53)

     at java.base/sun.nio.fs.WindowsFileAttributeViews$Basic.readAttributes(WindowsFileAttributeViews.java:38)

     at java.base/sun.nio.fs.WindowsFileSystemProvider.readAttributes(WindowsFileSystemProvider.java:198)

     at java.base/java.nio.file.Files.readAttributes(Files.java:1764)

     at java.base/java.util.zip.ZipFile$Source.get(ZipFile.java:1259)

     at java.base/java.util.zip.ZipFile$CleanableResource.(ZipFile.java:733)

     at java.base/java.util.zip.ZipFile$CleanableResource.get(ZipFile.java:850)

     at java.base/java.util.zip.ZipFile.(ZipFile.java:248)

     at java.base/java.util.zip.ZipFile.(ZipFile.java:177)

     at java.base/java.util.zip.ZipFile.(ZipFile.java:191)

     at fr.univ_nantes.codex_processor.core.measure.MeasureProcessor.run(MeasureProcessor.java:43)

     at fr.univ_nantes.codex_processor.gui.DatasetMeasureProcessor.run(DatasetMeasureProcessor.java:45)

     at fr.univ_nantes.codex_processor.gui.MainController$1.handle(MainController.java:160)

     at fr.univ_nantes.codex_processor.gui.MainController$1.handle(MainController.java:64)

     at com.sun.javafx.event.CompositeEventHandler.dispatchBubblingEvent(CompositeEventHandler.java:86)

     at com.sun.javafx.event.EventHandlerManager.dispatchBubblingEvent(EventHandlerManager.java:234)

     at com.sun.javafx.event.EventHandlerManager.dispatchBubblingEvent(EventHandlerManager.java:191)

     at com.sun.javafx.event.CompositeEventDispatcher.dispatchBubblingEvent(CompositeEventDispatcher.java:59)

     at com.sun.javafx.event.BasicEventDispatcher.dispatchEvent(BasicEventDispatcher.java:58)

     at com.sun.javafx.event.EventDispatchChainImpl.dispatchEvent(EventDispatchChainImpl.java:114)

     at com.sun.javafx.event.BasicEventDispatcher.dispatchEvent(BasicEventDispatcher.java:56)

     at com.sun.javafx.event.EventDispatchChainImpl.dispatchEvent(EventDispatchChainImpl.java:114)

     at com.sun.javafx.event.BasicEventDispatcher.dispatchEvent(BasicEventDispatcher.java:56)

     at com.sun.javafx.event.EventDispatchChainImpl.dispatchEvent(EventDispatchChainImpl.java:114)

     at com.sun.javafx.event.EventUtil.fireEventImpl(EventUtil.java:74)

     at com.sun.javafx.event.EventUtil.fireEvent(EventUtil.java:49)

     at javafx.event.Event.fireEvent(Event.java:198)

     at javafx.scene.Node.fireEvent(Node.java:8889)

     at javafx.scene.control.Button.fire(Button.java:203)

     at com.sun.javafx.scene.control.behavior.ButtonBehavior.mouseReleased(ButtonBehavior.java:208)

     at com.sun.javafx.scene.control.inputmap.InputMap.handle(InputMap.java:274)

     at com.sun.javafx.event.CompositeEventHandler$NormalEventHandlerRecord.handleBubblingEvent(CompositeEventHandler.java:247)

     at com.sun.javafx.event.CompositeEventHandler.dispatchBubblingEvent(CompositeEventHandler.java:80)

     at com.sun.javafx.event.EventHandlerManager.dispatchBubblingEvent(EventHandlerManager.java:234)

     at com.sun.javafx.event.EventHandlerManager.dispatchBubblingEvent(EventHandlerManager.java:191)

     at com.sun.javafx.event.CompositeEventDispatcher.dispatchBubblingEvent(CompositeEventDispatcher.java:59)

     at com.sun.javafx.event.BasicEventDispatcher.dispatchEvent(BasicEventDispatcher.java:58)

     at com.sun.javafx.event.EventDispatchChainImpl.dispatchEvent(EventDispatchChainImpl.java:114)

     at com.sun.javafx.event.BasicEventDispatcher.dispatchEvent(BasicEventDispatcher.java:56)

     at com.sun.javafx.event.EventDispatchChainImpl.dispatchEvent(EventDispatchChainImpl.java:114)

     at com.sun.javafx.event.BasicEventDispatcher.dispatchEvent(BasicEventDispatcher.java:56)

     at com.sun.javafx.event.EventDispatchChainImpl.dispatchEvent(EventDispatchChainImpl.java:114)

     at com.sun.javafx.event.EventUtil.fireEventImpl(EventUtil.java:74)

     at com.sun.javafx.event.EventUtil.fireEvent(EventUtil.java:54)

     at javafx.event.Event.fireEvent(Event.java:198)

     at javafx.scene.Scene$MouseHandler.process(Scene.java:3856)

     at javafx.scene.Scene.processMouseEvent(Scene.java:1851)

     at javafx.scene.Scene$ScenePeerListener.mouseEvent(Scene.java:2584)

     at com.sun.javafx.tk.quantum.GlassViewEventHandler$MouseEventNotification.run(GlassViewEventHandler.java:409)

     at com.sun.javafx.tk.quantum.GlassViewEventHandler$MouseEventNotification.run(GlassViewEventHandler.java:299)

     at java.base/java.security.AccessController.doPrivileged(Native Method)

     at com.sun.javafx.tk.quantum.GlassViewEventHandler.lambda$handleMouseEvent$2(GlassViewEventHandler.java:447)

     at com.sun.javafx.tk.quantum.QuantumToolkit.runWithoutRenderLock(QuantumToolkit.java:412)

     at com.sun.javafx.tk.quantum.GlassViewEventHandler.handleMouseEvent(GlassViewEventHandler.java:446)

     at com.sun.glass.ui.View.handleMouseEvent(View.java:556)

     at com.sun.glass.ui.View.notifyMouse(View.java:942)

     at com.sun.glass.ui.win.WinApplication._runLoop(Native Method)

     at com.sun.glass.ui.win.WinApplication.lambda$runLoop$3(WinApplication.java:174)

     at java.base/java.lang.Thread.run(Thread.java:829)

 java.nio.file.NoSuchFileException: C:\Users\IT-WL-annkl878\Documents\notes\Codex_Analysis\test_multiplxProcessor\out\rois_cy002_ch1.zip

     at java.base/sun.nio.fs.WindowsException.translateToIOException(WindowsException.java:85)

     at java.base/sun.nio.fs.WindowsException.rethrowAsIOException(WindowsException.java:103)

     at java.base/sun.nio.fs.WindowsException.rethrowAsIOException(WindowsException.java:108)

     at java.base/sun.nio.fs.WindowsFileAttributeViews$Basic.readAttributes(WindowsFileAttributeViews.java:53)

     at java.base/sun.nio.fs.WindowsFileAttributeViews$Basic.readAttributes(WindowsFileAttributeViews.java:38)

     at java.base/sun.nio.fs.WindowsFileSystemProvider.readAttributes(WindowsFileSystemProvider.java:198)

     at java.base/java.nio.file.Files.readAttributes(Files.java:1764)

     at java.base/java.util.zip.ZipFile$Source.get(ZipFile.java:1259)

     at java.base/java.util.zip.ZipFile$CleanableResource.(ZipFile.java:733)

     at java.base/java.util.zip.ZipFile$CleanableResource.get(ZipFile.java:850)

     at java.base/java.util.zip.ZipFile.(ZipFile.java:248)

     at java.base/java.util.zip.ZipFile.(ZipFile.java:177)

     at java.base/java.util.zip.ZipFile.(ZipFile.java:191)

     at fr.univ_nantes.codex_processor.core.measure.MeasureProcessor.run(MeasureProcessor.java:43)

     at fr.univ_nantes.codex_processor.gui.DatasetMeasureProcessor.run(DatasetMeasureProcessor.java:45)

     at fr.univ_nantes.codex_processor.gui.MainController$1.handle(MainController.java:160)

     at fr.univ_nantes.codex_processor.gui.MainController$1.handle(MainController.java:64)

     at com.sun.javafx.event.CompositeEventHandler.dispatchBubblingEvent(CompositeEventHandler.java:86)

     at com.sun.javafx.event.EventHandlerManager.dispatchBubblingEvent(EventHandlerManager.java:234)

     at com.sun.javafx.event.EventHandlerManager.dispatchBubblingEvent(EventHandlerManager.java:191)

     at com.sun.javafx.event.CompositeEventDispatcher.dispatchBubblingEvent(CompositeEventDispatcher.java:59)

     at com.sun.javafx.event.BasicEventDispatcher.dispatchEvent(BasicEventDispatcher.java:58)

     at com.sun.javafx.event.EventDispatchChainImpl.dispatchEvent(EventDispatchChainImpl.java:114)

     at com.sun.javafx.event.BasicEventDispatcher.dispatchEvent(BasicEventDispatcher.java:56)

     at com.sun.javafx.event.EventDispatchChainImpl.dispatchEvent(EventDispatchChainImpl.java:114)

     at com.sun.javafx.event.BasicEventDispatcher.dispatchEvent(BasicEventDispatcher.java:56)

     at com.sun.javafx.event.EventDispatchChainImpl.dispatchEvent(EventDispatchChainImpl.java:114)

     at com.sun.javafx.event.EventUtil.fireEventImpl(EventUtil.java:74)

     at com.sun.javafx.event.EventUtil.fireEvent(EventUtil.java:49)

     at javafx.event.Event.fireEvent(Event.java:198)

     at javafx.scene.Node.fireEvent(Node.java:8889)

     at javafx.scene.control.Button.fire(Button.java:203)

     at com.sun.javafx.scene.control.behavior.ButtonBehavior.mouseReleased(ButtonBehavior.java:208)

     at com.sun.javafx.scene.control.inputmap.InputMap.handle(InputMap.java:274)

     at com.sun.javafx.event.CompositeEventHandler$NormalEventHandlerRecord.handleBubblingEvent(CompositeEventHandler.java:247)

     at com.sun.javafx.event.CompositeEventHandler.dispatchBubblingEvent(CompositeEventHandler.java:80)

     at com.sun.javafx.event.EventHandlerManager.dispatchBubblingEvent(EventHandlerManager.java:234)

     at com.sun.javafx.event.EventHandlerManager.dispatchBubblingEvent(EventHandlerManager.java:191)

     at com.sun.javafx.event.CompositeEventDispatcher.dispatchBubblingEvent(CompositeEventDispatcher.java:59)

     at com.sun.javafx.event.BasicEventDispatcher.dispatchEvent(BasicEventDispatcher.java:58)

     at com.sun.javafx.event.EventDispatchChainImpl.dispatchEvent(EventDispatchChainImpl.java:114)

     at com.sun.javafx.event.BasicEventDispatcher.dispatchEvent(BasicEventDispatcher.java:56)

     at com.sun.javafx.event.EventDispatchChainImpl.dispatchEvent(EventDispatchChainImpl.java:114)

     at com.sun.javafx.event.BasicEventDispatcher.dispatchEvent(BasicEventDispatcher.java:56)

     at com.sun.javafx.event.EventDispatchChainImpl.dispatchEvent(EventDispatchChainImpl.java:114)

     at com.sun.javafx.event.EventUtil.fireEventImpl(EventUtil.java:74)

     at com.sun.javafx.event.EventUtil.fireEvent(EventUtil.java:54)

     at javafx.event.Event.fireEvent(Event.java:198)

     at javafx.scene.Scene$MouseHandler.process(Scene.java:3856)

     at javafx.scene.Scene.processMouseEvent(Scene.java:1851)

     at javafx.scene.Scene$ScenePeerListener.mouseEvent(Scene.java:2584)

     at com.sun.javafx.tk.quantum.GlassViewEventHandler$MouseEventNotification.run(GlassViewEventHandler.java:409)

     at com.sun.javafx.tk.quantum.GlassViewEventHandler$MouseEventNotification.run(GlassViewEventHandler.java:299)

     at java.base/java.security.AccessController.doPrivileged(Native Method)

     at com.sun.javafx.tk.quantum.GlassViewEventHandler.lambda$handleMouseEvent$2(GlassViewEventHandler.java:447)

     at com.sun.javafx.tk.quantum.QuantumToolkit.runWithoutRenderLock(QuantumToolkit.java:412)

     at com.sun.javafx.tk.quantum.GlassViewEventHandler.handleMouseEvent(GlassViewEventHandler.java:446)

     at com.sun.glass.ui.View.handleMouseEvent(View.java:556)

     at com.sun.glass.ui.View.notifyMouse(View.java:942)

     at com.sun.glass.ui.win.WinApplication._runLoop(Native Method)

     at com.sun.glass.ui.win.WinApplication.lambda$runLoop$3(WinApplication.java:174)

     at java.base/java.lang.Thread.run(Thread.java:829)

 java.nio.file.NoSuchFileException: C:\Users\IT-WL-annkl878\Documents\notes\Codex_Analysis\test_multiplxProcessor\out\rois_cy002_ch1.zip

     at java.base/sun.nio.fs.WindowsException.translateToIOException(WindowsException.java:85)

     at java.base/sun.nio.fs.WindowsException.rethrowAsIOException(WindowsException.java:103)

     at java.base/sun.nio.fs.WindowsException.rethrowAsIOException(WindowsException.java:108)

     at java.base/sun.nio.fs.WindowsFileAttributeViews$Basic.readAttributes(WindowsFileAttributeViews.java:53)

     at java.base/sun.nio.fs.WindowsFileAttributeViews$Basic.readAttributes(WindowsFileAttributeViews.java:38)

     at java.base/sun.nio.fs.WindowsFileSystemProvider.readAttributes(WindowsFileSystemProvider.java:198)

     at java.base/java.nio.file.Files.readAttributes(Files.java:1764)

     at java.base/java.util.zip.ZipFile$Source.get(ZipFile.java:1259)

     at java.base/java.util.zip.ZipFile$CleanableResource.(ZipFile.java:733)

     at java.base/java.util.zip.ZipFile$CleanableResource.get(ZipFile.java:850)

     at java.base/java.util.zip.ZipFile.(ZipFile.java:248)

     at java.base/java.util.zip.ZipFile.(ZipFile.java:177)

     at java.base/java.util.zip.ZipFile.(ZipFile.java:191)

     at fr.univ_nantes.codex_processor.core.measure.MeasureProcessor.run(MeasureProcessor.java:43)

     at fr.univ_nantes.codex_processor.gui.DatasetMeasureProcessor.run(DatasetMeasureProcessor.java:45)

     at fr.univ_nantes.codex_processor.gui.MainController$1.handle(MainController.java:160)

     at fr.univ_nantes.codex_processor.gui.MainController$1.handle(MainController.java:64)

     at com.sun.javafx.event.CompositeEventHandler.dispatchBubblingEvent(CompositeEventHandler.java:86)

     at com.sun.javafx.event.EventHandlerManager.dispatchBubblingEvent(EventHandlerManager.java:234)

     at com.sun.javafx.event.EventHandlerManager.dispatchBubblingEvent(EventHandlerManager.java:191)

     at com.sun.javafx.event.CompositeEventDispatcher.dispatchBubblingEvent(CompositeEventDispatcher.java:59)

     at com.sun.javafx.event.BasicEventDispatcher.dispatchEvent(BasicEventDispatcher.java:58)

     at com.sun.javafx.event.EventDispatchChainImpl.dispatchEvent(EventDispatchChainImpl.java:114)

     at com.sun.javafx.event.BasicEventDispatcher.dispatchEvent(BasicEventDispatcher.java:56)

     at com.sun.javafx.event.EventDispatchChainImpl.dispatchEvent(EventDispatchChainImpl.java:114)

     at com.sun.javafx.event.BasicEventDispatcher.dispatchEvent(BasicEventDispatcher.java:56)

     at com.sun.javafx.event.EventDispatchChainImpl.dispatchEvent(EventDispatchChainImpl.java:114)

     at com.sun.javafx.event.EventUtil.fireEventImpl(EventUtil.java:74)

     at com.sun.javafx.event.EventUtil.fireEvent(EventUtil.java:49)

     at javafx.event.Event.fireEvent(Event.java:198)

     at javafx.scene.Node.fireEvent(Node.java:8889)

     at javafx.scene.control.Button.fire(Button.java:203)

     at com.sun.javafx.scene.control.behavior.ButtonBehavior.mouseReleased(ButtonBehavior.java:208)

     at com.sun.javafx.scene.control.inputmap.InputMap.handle(InputMap.java:274)

     at com.sun.javafx.event.CompositeEventHandler$NormalEventHandlerRecord.handleBubblingEvent(CompositeEventHandler.java:247)

     at com.sun.javafx.event.CompositeEventHandler.dispatchBubblingEvent(CompositeEventHandler.java:80)

     at com.sun.javafx.event.EventHandlerManager.dispatchBubblingEvent(EventHandlerManager.java:234)

     at com.sun.javafx.event.EventHandlerManager.dispatchBubblingEvent(EventHandlerManager.java:191)

     at com.sun.javafx.event.CompositeEventDispatcher.dispatchBubblingEvent(CompositeEventDispatcher.java:59)

     at com.sun.javafx.event.BasicEventDispatcher.dispatchEvent(BasicEventDispatcher.java:58)

     at com.sun.javafx.event.EventDispatchChainImpl.dispatchEvent(EventDispatchChainImpl.java:114)

     at com.sun.javafx.event.BasicEventDispatcher.dispatchEvent(BasicEventDispatcher.java:56)

     at com.sun.javafx.event.EventDispatchChainImpl.dispatchEvent(EventDispatchChainImpl.java:114)

     at com.sun.javafx.event.BasicEventDispatcher.dispatchEvent(BasicEventDispatcher.java:56)

     at com.sun.javafx.event.EventDispatchChainImpl.dispatchEvent(EventDispatchChainImpl.java:114)

     at com.sun.javafx.event.EventUtil.fireEventImpl(EventUtil.java:74)

     at com.sun.javafx.event.EventUtil.fireEvent(EventUtil.java:54)

     at javafx.event.Event.fireEvent(Event.java:198)

     at javafx.scene.Scene$MouseHandler.process(Scene.java:3856)

     at javafx.scene.Scene.processMouseEvent(Scene.java:1851)

     at javafx.scene.Scene$ScenePeerListener.mouseEvent(Scene.java:2584)

     at com.sun.javafx.tk.quantum.GlassViewEventHandler$MouseEventNotification.run(GlassViewEventHandler.java:409)

     at com.sun.javafx.tk.quantum.GlassViewEventHandler$MouseEventNotification.run(GlassViewEventHandler.java:299)

     at java.base/java.security.AccessController.doPrivileged(Native Method)

     at com.sun.javafx.tk.quantum.GlassViewEventHandler.lambda$handleMouseEvent$2(GlassViewEventHandler.java:447)

     at com.sun.javafx.tk.quantum.QuantumToolkit.runWithoutRenderLock(QuantumToolkit.java:412)

     at com.sun.javafx.tk.quantum.GlassViewEventHandler.handleMouseEvent(GlassViewEventHandler.java:446)

     at com.sun.glass.ui.View.handleMouseEvent(View.java:556)

     at com.sun.glass.ui.View.notifyMouse(View.java:942)

     at com.sun.glass.ui.win.WinApplication._runLoop(Native Method)

     at com.sun.glass.ui.win.WinApplication.lambda$runLoop$3(WinApplication.java:174)

     at java.base/java.lang.Thread.run(Thread.java:829)

 java.nio.file.NoSuchFileException: C:\Users\IT-WL-annkl878\Documents\notes\Codex_Analysis\test_multiplxProcessor\out\rois_cy002_ch1.zip

     at java.base/sun.nio.fs.WindowsException.translateToIOException(WindowsException.java:85)

     at java.base/sun.nio.fs.WindowsException.rethrowAsIOException(WindowsException.java:103)

     at java.base/sun.nio.fs.WindowsException.rethrowAsIOException(WindowsException.java:108)

     at java.base/sun.nio.fs.WindowsFileAttributeViews$Basic.readAttributes(WindowsFileAttributeViews.java:53)

     at java.base/sun.nio.fs.WindowsFileAttributeViews$Basic.readAttributes(WindowsFileAttributeViews.java:38)

     at java.base/sun.nio.fs.WindowsFileSystemProvider.readAttributes(WindowsFileSystemProvider.java:198)

     at java.base/java.nio.file.Files.readAttributes(Files.java:1764)

     at java.base/java.util.zip.ZipFile$Source.get(ZipFile.java:1259)

     at java.base/java.util.zip.ZipFile$CleanableResource.(ZipFile.java:733)

     at java.base/java.util.zip.ZipFile$CleanableResource.get(ZipFile.java:850)

     at java.base/java.util.zip.ZipFile.(ZipFile.java:248)

     at java.base/java.util.zip.ZipFile.(ZipFile.java:177)

     at java.base/java.util.zip.ZipFile.(ZipFile.java:191)

     at fr.univ_nantes.codex_processor.core.measure.MeasureProcessor.run(MeasureProcessor.java:43)

     at fr.univ_nantes.codex_processor.gui.DatasetMeasureProcessor.run(DatasetMeasureProcessor.java:45)

     at fr.univ_nantes.codex_processor.gui.MainController$1.handle(MainController.java:160)

     at fr.univ_nantes.codex_processor.gui.MainController$1.handle(MainController.java:64)

     at com.sun.javafx.event.CompositeEventHandler.dispatchBubblingEvent(CompositeEventHandler.java:86)

     at com.sun.javafx.event.EventHandlerManager.dispatchBubblingEvent(EventHandlerManager.java:234)

     at com.sun.javafx.event.EventHandlerManager.dispatchBubblingEvent(EventHandlerManager.java:191)

     at com.sun.javafx.event.CompositeEventDispatcher.dispatchBubblingEvent(CompositeEventDispatcher.java:59)

     at com.sun.javafx.event.BasicEventDispatcher.dispatchEvent(BasicEventDispatcher.java:58)

     at com.sun.javafx.event.EventDispatchChainImpl.dispatchEvent(EventDispatchChainImpl.java:114)

     at com.sun.javafx.event.BasicEventDispatcher.dispatchEvent(BasicEventDispatcher.java:56)

     at com.sun.javafx.event.EventDispatchChainImpl.dispatchEvent(EventDispatchChainImpl.java:114)

     at com.sun.javafx.event.BasicEventDispatcher.dispatchEvent(BasicEventDispatcher.java:56)

     at com.sun.javafx.event.EventDispatchChainImpl.dispatchEvent(EventDispatchChainImpl.java:114)

     at com.sun.javafx.event.EventUtil.fireEventImpl(EventUtil.java:74)

     at com.sun.javafx.event.EventUtil.fireEvent(EventUtil.java:49)

     at javafx.event.Event.fireEvent(Event.java:198)

     at javafx.scene.Node.fireEvent(Node.java:8889)

     at javafx.scene.control.Button.fire(Button.java:203)

     at com.sun.javafx.scene.control.behavior.ButtonBehavior.mouseReleased(ButtonBehavior.java:208)

     at com.sun.javafx.scene.control.inputmap.InputMap.handle(InputMap.java:274)

     at com.sun.javafx.event.CompositeEventHandler$NormalEventHandlerRecord.handleBubblingEvent(CompositeEventHandler.java:247)

     at com.sun.javafx.event.CompositeEventHandler.dispatchBubblingEvent(CompositeEventHandler.java:80)

     at com.sun.javafx.event.EventHandlerManager.dispatchBubblingEvent(EventHandlerManager.java:234)

     at com.sun.javafx.event.EventHandlerManager.dispatchBubblingEvent(EventHandlerManager.java:191)

     at com.sun.javafx.event.CompositeEventDispatcher.dispatchBubblingEvent(CompositeEventDispatcher.java:59)

     at com.sun.javafx.event.BasicEventDispatcher.dispatchEvent(BasicEventDispatcher.java:58)

     at com.sun.javafx.event.EventDispatchChainImpl.dispatchEvent(EventDispatchChainImpl.java:114)

     at com.sun.javafx.event.BasicEventDispatcher.dispatchEvent(BasicEventDispatcher.java:56)

     at com.sun.javafx.event.EventDispatchChainImpl.dispatchEvent(EventDispatchChainImpl.java:114)

     at com.sun.javafx.event.BasicEventDispatcher.dispatchEvent(BasicEventDispatcher.java:56)

     at com.sun.javafx.event.EventDispatchChainImpl.dispatchEvent(EventDispatchChainImpl.java:114)

     at com.sun.javafx.event.EventUtil.fireEventImpl(EventUtil.java:74)

     at com.sun.javafx.event.EventUtil.fireEvent(EventUtil.java:54)

     at javafx.event.Event.fireEvent(Event.java:198)

     at javafx.scene.Scene$MouseHandler.process(Scene.java:3856)

     at javafx.scene.Scene.processMouseEvent(Scene.java:1851)

     at javafx.scene.Scene$ScenePeerListener.mouseEvent(Scene.java:2584)

     at com.sun.javafx.tk.quantum.GlassViewEventHandler$MouseEventNotification.run(GlassViewEventHandler.java:409)

     at com.sun.javafx.tk.quantum.GlassViewEventHandler$MouseEventNotification.run(GlassViewEventHandler.java:299)

     at java.base/java.security.AccessController.doPrivileged(Native Method)

     at com.sun.javafx.tk.quantum.GlassViewEventHandler.lambda$handleMouseEvent$2(GlassViewEventHandler.java:447)

     at com.sun.javafx.tk.quantum.QuantumToolkit.runWithoutRenderLock(QuantumToolkit.java:412)

     at com.sun.javafx.tk.quantum.GlassViewEventHandler.handleMouseEvent(GlassViewEventHandler.java:446)

     at com.sun.glass.ui.View.handleMouseEvent(View.java:556)

     at com.sun.glass.ui.View.notifyMouse(View.java:942)

     at com.sun.glass.ui.win.WinApplication._runLoop(Native Method)

     at com.sun.glass.ui.win.WinApplication.lambda$runLoop$3(WinApplication.java:174)

     at java.base/java.lang.Thread.run(Thread.java:829)

 java.nio.file.NoSuchFileException: C:\Users\IT-WL-annkl878\Documents\notes\Codex_Analysis\test_multiplxProcessor\out\rois_cy002_ch1.zip

     at java.base/sun.nio.fs.WindowsException.translateToIOException(WindowsException.java:85)

     at java.base/sun.nio.fs.WindowsException.rethrowAsIOException(WindowsException.java:103)

     at java.base/sun.nio.fs.WindowsException.rethrowAsIOException(WindowsException.java:108)

     at java.base/sun.nio.fs.WindowsFileAttributeViews$Basic.readAttributes(WindowsFileAttributeViews.java:53)

     at java.base/sun.nio.fs.WindowsFileAttributeViews$Basic.readAttributes(WindowsFileAttributeViews.java:38)

     at java.base/sun.nio.fs.WindowsFileSystemProvider.readAttributes(WindowsFileSystemProvider.java:198)

     at java.base/java.nio.file.Files.readAttributes(Files.java:1764)

     at java.base/java.util.zip.ZipFile$Source.get(ZipFile.java:1259)

     at java.base/java.util.zip.ZipFile$CleanableResource.(ZipFile.java:733)

     at java.base/java.util.zip.ZipFile$CleanableResource.get(ZipFile.java:850)

     at java.base/java.util.zip.ZipFile.(ZipFile.java:248)

     at java.base/java.util.zip.ZipFile.(ZipFile.java:177)

     at java.base/java.util.zip.ZipFile.(ZipFile.java:191)

     at fr.univ_nantes.codex_processor.core.measure.MeasureProcessor.run(MeasureProcessor.java:43)

     at fr.univ_nantes.codex_processor.gui.DatasetMeasureProcessor.run(DatasetMeasureProcessor.java:45)

     at fr.univ_nantes.codex_processor.gui.MainController$1.handle(MainController.java:160)

     at fr.univ_nantes.codex_processor.gui.MainController$1.handle(MainController.java:64)

     at com.sun.javafx.event.CompositeEventHandler.dispatchBubblingEvent(CompositeEventHandler.java:86)

     at com.sun.javafx.event.EventHandlerManager.dispatchBubblingEvent(EventHandlerManager.java:234)

     at com.sun.javafx.event.EventHandlerManager.dispatchBubblingEvent(EventHandlerManager.java:191)

     at com.sun.javafx.event.CompositeEventDispatcher.dispatchBubblingEvent(CompositeEventDispatcher.java:59)

     at com.sun.javafx.event.BasicEventDispatcher.dispatchEvent(BasicEventDispatcher.java:58)

     at com.sun.javafx.event.EventDispatchChainImpl.dispatchEvent(EventDispatchChainImpl.java:114)

     at com.sun.javafx.event.BasicEventDispatcher.dispatchEvent(BasicEventDispatcher.java:56)

     at com.sun.javafx.event.EventDispatchChainImpl.dispatchEvent(EventDispatchChainImpl.java:114)

     at com.sun.javafx.event.BasicEventDispatcher.dispatchEvent(BasicEventDispatcher.java:56)

     at com.sun.javafx.event.EventDispatchChainImpl.dispatchEvent(EventDispatchChainImpl.java:114)

     at com.sun.javafx.event.EventUtil.fireEventImpl(EventUtil.java:74)

     at com.sun.javafx.event.EventUtil.fireEvent(EventUtil.java:49)

     at javafx.event.Event.fireEvent(Event.java:198)

     at javafx.scene.Node.fireEvent(Node.java:8889)

     at javafx.scene.control.Button.fire(Button.java:203)

     at com.sun.javafx.scene.control.behavior.ButtonBehavior.mouseReleased(ButtonBehavior.java:208)

     at com.sun.javafx.scene.control.inputmap.InputMap.handle(InputMap.java:274)

     at com.sun.javafx.event.CompositeEventHandler$NormalEventHandlerRecord.handleBubblingEvent(CompositeEventHandler.java:247)

     at com.sun.javafx.event.CompositeEventHandler.dispatchBubblingEvent(CompositeEventHandler.java:80)

     at com.sun.javafx.event.EventHandlerManager.dispatchBubblingEvent(EventHandlerManager.java:234)

     at com.sun.javafx.event.EventHandlerManager.dispatchBubblingEvent(EventHandlerManager.java:191)

     at com.sun.javafx.event.CompositeEventDispatcher.dispatchBubblingEvent(CompositeEventDispatcher.java:59)

     at com.sun.javafx.event.BasicEventDispatcher.dispatchEvent(BasicEventDispatcher.java:58)

     at com.sun.javafx.event.EventDispatchChainImpl.dispatchEvent(EventDispatchChainImpl.java:114)

     at com.sun.javafx.event.BasicEventDispatcher.dispatchEvent(BasicEventDispatcher.java:56)

     at com.sun.javafx.event.EventDispatchChainImpl.dispatchEvent(EventDispatchChainImpl.java:114)

     at com.sun.javafx.event.BasicEventDispatcher.dispatchEvent(BasicEventDispatcher.java:56)

     at com.sun.javafx.event.EventDispatchChainImpl.dispatchEvent(EventDispatchChainImpl.java:114)

     at com.sun.javafx.event.EventUtil.fireEventImpl(EventUtil.java:74)

     at com.sun.javafx.event.EventUtil.fireEvent(EventUtil.java:54)

     at javafx.event.Event.fireEvent(Event.java:198)

     at javafx.scene.Scene$MouseHandler.process(Scene.java:3856)

     at javafx.scene.Scene.processMouseEvent(Scene.java:1851)

     at javafx.scene.Scene$ScenePeerListener.mouseEvent(Scene.java:2584)

     at com.sun.javafx.tk.quantum.GlassViewEventHandler$MouseEventNotification.run(GlassViewEventHandler.java:409)

     at com.sun.javafx.tk.quantum.GlassViewEventHandler$MouseEventNotification.run(GlassViewEventHandler.java:299)

     at java.base/java.security.AccessController.doPrivileged(Native Method)

     at com.sun.javafx.tk.quantum.GlassViewEventHandler.lambda$handleMouseEvent$2(GlassViewEventHandler.java:447)

     at com.sun.javafx.tk.quantum.QuantumToolkit.runWithoutRenderLock(QuantumToolkit.java:412)

     at com.sun.javafx.tk.quantum.GlassViewEventHandler.handleMouseEvent(GlassViewEventHandler.java:446)

     at com.sun.glass.ui.View.handleMouseEvent(View.java:556)

     at com.sun.glass.ui.View.notifyMouse(View.java:942)

     at com.sun.glass.ui.win.WinApplication._runLoop(Native Method)

     at com.sun.glass.ui.win.WinApplication.lambda$runLoop$3(WinApplication.java:174)

     at java.base/java.lang.Thread.run(Thread.java:829)

 java.nio.file.NoSuchFileException: C:\Users\IT-WL-annkl878\Documents\notes\Codex_Analysis\test_multiplxProcessor\out\rois_cy002_ch1.zip

     at java.base/sun.nio.fs.WindowsException.translateToIOException(WindowsException.java:85)

     at java.base/sun.nio.fs.WindowsException.rethrowAsIOException(WindowsException.java:103)

     at java.base/sun.nio.fs.WindowsException.rethrowAsIOException(WindowsException.java:108)

     at java.base/sun.nio.fs.WindowsFileAttributeViews$Basic.readAttributes(WindowsFileAttributeViews.java:53)

     at java.base/sun.nio.fs.WindowsFileAttributeViews$Basic.readAttributes(WindowsFileAttributeViews.java:38)

     at java.base/sun.nio.fs.WindowsFileSystemProvider.readAttributes(WindowsFileSystemProvider.java:198)

     at java.base/java.nio.file.Files.readAttributes(Files.java:1764)

     at java.base/java.util.zip.ZipFile$Source.get(ZipFile.java:1259)

     at java.base/java.util.zip.ZipFile$CleanableResource.(ZipFile.java:733)

     at java.base/java.util.zip.ZipFile$CleanableResource.get(ZipFile.java:850)

     at java.base/java.util.zip.ZipFile.(ZipFile.java:248)

     at java.base/java.util.zip.ZipFile.(ZipFile.java:177)

     at java.base/java.util.zip.ZipFile.(ZipFile.java:191)

     at fr.univ_nantes.codex_processor.core.measure.MeasureProcessor.run(MeasureProcessor.java:43)

     at fr.univ_nantes.codex_processor.gui.DatasetMeasureProcessor.run(DatasetMeasureProcessor.java:45)

     at fr.univ_nantes.codex_processor.gui.MainController$1.handle(MainController.java:160)

     at fr.univ_nantes.codex_processor.gui.MainController$1.handle(MainController.java:64)

     at com.sun.javafx.event.CompositeEventHandler.dispatchBubblingEvent(CompositeEventHandler.java:86)

     at com.sun.javafx.event.EventHandlerManager.dispatchBubblingEvent(EventHandlerManager.java:234)

     at com.sun.javafx.event.EventHandlerManager.dispatchBubblingEvent(EventHandlerManager.java:191)

     at com.sun.javafx.event.CompositeEventDispatcher.dispatchBubblingEvent(CompositeEventDispatcher.java:59)

     at com.sun.javafx.event.BasicEventDispatcher.dispatchEvent(BasicEventDispatcher.java:58)

     at com.sun.javafx.event.EventDispatchChainImpl.dispatchEvent(EventDispatchChainImpl.java:114)

     at com.sun.javafx.event.BasicEventDispatcher.dispatchEvent(BasicEventDispatcher.java:56)

     at com.sun.javafx.event.EventDispatchChainImpl.dispatchEvent(EventDispatchChainImpl.java:114)

     at com.sun.javafx.event.BasicEventDispatcher.dispatchEvent(BasicEventDispatcher.java:56)

     at com.sun.javafx.event.EventDispatchChainImpl.dispatchEvent(EventDispatchChainImpl.java:114)

     at com.sun.javafx.event.EventUtil.fireEventImpl(EventUtil.java:74)

     at com.sun.javafx.event.EventUtil.fireEvent(EventUtil.java:49)

     at javafx.event.Event.fireEvent(Event.java:198)

     at javafx.scene.Node.fireEvent(Node.java:8889)

     at javafx.scene.control.Button.fire(Button.java:203)

     at com.sun.javafx.scene.control.behavior.ButtonBehavior.mouseReleased(ButtonBehavior.java:208)

     at com.sun.javafx.scene.control.inputmap.InputMap.handle(InputMap.java:274)

     at com.sun.javafx.event.CompositeEventHandler$NormalEventHandlerRecord.handleBubblingEvent(CompositeEventHandler.java:247)

     at com.sun.javafx.event.CompositeEventHandler.dispatchBubblingEvent(CompositeEventHandler.java:80)

     at com.sun.javafx.event.EventHandlerManager.dispatchBubblingEvent(EventHandlerManager.java:234)

     at com.sun.javafx.event.EventHandlerManager.dispatchBubblingEvent(EventHandlerManager.java:191)

     at com.sun.javafx.event.CompositeEventDispatcher.dispatchBubblingEvent(CompositeEventDispatcher.java:59)

     at com.sun.javafx.event.BasicEventDispatcher.dispatchEvent(BasicEventDispatcher.java:58)

     at com.sun.javafx.event.EventDispatchChainImpl.dispatchEvent(EventDispatchChainImpl.java:114)

     at com.sun.javafx.event.BasicEventDispatcher.dispatchEvent(BasicEventDispatcher.java:56)

     at com.sun.javafx.event.EventDispatchChainImpl.dispatchEvent(EventDispatchChainImpl.java:114)

     at com.sun.javafx.event.BasicEventDispatcher.dispatchEvent(BasicEventDispatcher.java:56)

     at com.sun.javafx.event.EventDispatchChainImpl.dispatchEvent(EventDispatchChainImpl.java:114)

     at com.sun.javafx.event.EventUtil.fireEventImpl(EventUtil.java:74)

     at com.sun.javafx.event.EventUtil.fireEvent(EventUtil.java:54)

     at javafx.event.Event.fireEvent(Event.java:198)

     at javafx.scene.Scene$MouseHandler.process(Scene.java:3856)

     at javafx.scene.Scene.processMouseEvent(Scene.java:1851)

     at javafx.scene.Scene$ScenePeerListener.mouseEvent(Scene.java:2584)

     at com.sun.javafx.tk.quantum.GlassViewEventHandler$MouseEventNotification.run(GlassViewEventHandler.java:409)

     at com.sun.javafx.tk.quantum.GlassViewEventHandler$MouseEventNotification.run(GlassViewEventHandler.java:299)

     at java.base/java.security.AccessController.doPrivileged(Native Method)

     at com.sun.javafx.tk.quantum.GlassViewEventHandler.lambda$handleMouseEvent$2(GlassViewEventHandler.java:447)

     at com.sun.javafx.tk.quantum.QuantumToolkit.runWithoutRenderLock(QuantumToolkit.java:412)

     at com.sun.javafx.tk.quantum.GlassViewEventHandler.handleMouseEvent(GlassViewEventHandler.java:446)

     at com.sun.glass.ui.View.handleMouseEvent(View.java:556)

     at com.sun.glass.ui.View.notifyMouse(View.java:942)

     at com.sun.glass.ui.win.WinApplication._runLoop(Native Method)

     at com.sun.glass.ui.win.WinApplication.lambda$runLoop$3(WinApplication.java:174)

     at java.base/java.lang.Thread.run(Thread.java:829)

 Exception in thread "JavaFX Application Thread" java.lang.IllegalArgumentException: Column cy001_ch1_DAPI_Area is not present in table Measure results

     at tech.tablesaw.api.Table.columnIndex(Table.java:437)

     at tech.tablesaw.table.Relation.doubleColumn(Relation.java:379)

     at fr.univ_nantes.codex_processor.gui.DatasetMeasureProcessor.run(DatasetMeasureProcessor.java:62)

     at fr.univ_nantes.codex_processor.gui.MainController$1.handle(MainController.java:160)

     at fr.univ_nantes.codex_processor.gui.MainController$1.handle(MainController.java:64)

     at com.sun.javafx.event.CompositeEventHandler.dispatchBubblingEvent(CompositeEventHandler.java:86)

     at com.sun.javafx.event.EventHandlerManager.dispatchBubblingEvent(EventHandlerManager.java:234)

     at com.sun.javafx.event.EventHandlerManager.dispatchBubblingEvent(EventHandlerManager.java:191)

     at com.sun.javafx.event.CompositeEventDispatcher.dispatchBubblingEvent(CompositeEventDispatcher.java:59)

     at com.sun.javafx.event.BasicEventDispatcher.dispatchEvent(BasicEventDispatcher.java:58)

     at com.sun.javafx.event.EventDispatchChainImpl.dispatchEvent(EventDispatchChainImpl.java:114)

     at com.sun.javafx.event.BasicEventDispatcher.dispatchEvent(BasicEventDispatcher.java:56)

     at com.sun.javafx.event.EventDispatchChainImpl.dispatchEvent(EventDispatchChainImpl.java:114)

     at com.sun.javafx.event.BasicEventDispatcher.dispatchEvent(BasicEventDispatcher.java:56)

     at com.sun.javafx.event.EventDispatchChainImpl.dispatchEvent(EventDispatchChainImpl.java:114)

     at com.sun.javafx.event.EventUtil.fireEventImpl(EventUtil.java:74)

     at com.sun.javafx.event.EventUtil.fireEvent(EventUtil.java:49)

     at javafx.event.Event.fireEvent(Event.java:198)

     at javafx.scene.Node.fireEvent(Node.java:8889)

     at javafx.scene.control.Button.fire(Button.java:203)

     at com.sun.javafx.scene.control.behavior.ButtonBehavior.mouseReleased(ButtonBehavior.java:208)

     at com.sun.javafx.scene.control.inputmap.InputMap.handle(InputMap.java:274)

     at com.sun.javafx.event.CompositeEventHandler$NormalEventHandlerRecord.handleBubblingEvent(CompositeEventHandler.java:247)

     at com.sun.javafx.event.CompositeEventHandler.dispatchBubblingEvent(CompositeEventHandler.java:80)

     at com.sun.javafx.event.EventHandlerManager.dispatchBubblingEvent(EventHandlerManager.java:234)

     at com.sun.javafx.event.EventHandlerManager.dispatchBubblingEvent(EventHandlerManager.java:191)

     at com.sun.javafx.event.CompositeEventDispatcher.dispatchBubblingEvent(CompositeEventDispatcher.java:59)

     at com.sun.javafx.event.BasicEventDispatcher.dispatchEvent(BasicEventDispatcher.java:58)

     at com.sun.javafx.event.EventDispatchChainImpl.dispatchEvent(EventDispatchChainImpl.java:114)

     at com.sun.javafx.event.BasicEventDispatcher.dispatchEvent(BasicEventDispatcher.java:56)

     at com.sun.javafx.event.EventDispatchChainImpl.dispatchEvent(EventDispatchChainImpl.java:114)

     at com.sun.javafx.event.BasicEventDispatcher.dispatchEvent(BasicEventDispatcher.java:56)

     at com.sun.javafx.event.EventDispatchChainImpl.dispatchEvent(EventDispatchChainImpl.java:114)

     at com.sun.javafx.event.EventUtil.fireEventImpl(EventUtil.java:74)

     at com.sun.javafx.event.EventUtil.fireEvent(EventUtil.java:54)

     at javafx.event.Event.fireEvent(Event.java:198)

     at javafx.scene.Scene$MouseHandler.process(Scene.java:3856)

     at javafx.scene.Scene.processMouseEvent(Scene.java:1851)

     at javafx.scene.Scene$ScenePeerListener.mouseEvent(Scene.java:2584)

     at com.sun.javafx.tk.quantum.GlassViewEventHandler$MouseEventNotification.run(GlassViewEventHandler.java:409)

     at com.sun.javafx.tk.quantum.GlassViewEventHandler$MouseEventNotification.run(GlassViewEventHandler.java:299)

     at java.base/java.security.AccessController.doPrivileged(Native Method)

     at com.sun.javafx.tk.quantum.GlassViewEventHandler.lambda$handleMouseEvent$2(GlassViewEventHandler.java:447)

     at com.sun.javafx.tk.quantum.QuantumToolkit.runWithoutRenderLock(QuantumToolkit.java:412)

     at com.sun.javafx.tk.quantum.GlassViewEventHandler.handleMouseEvent(GlassViewEventHandler.java:446)

     at com.sun.glass.ui.View.handleMouseEvent(View.java:556)

     at com.sun.glass.ui.View.notifyMouse(View.java:942)

     at com.sun.glass.ui.win.WinApplication._runLoop(Native Method)

     at com.sun.glass.ui.win.WinApplication.lambda$runLoop$3(WinApplication.java:174)

     at java.base/java.lang.Thread.run(Thread.java:829)

 ^C

# Response

**Published:** 2022-11-08

**Authors:** Anna Klemm

output_gui9

 Extended depth of field - completed : 0 / 96 Extended depth of field - completed : 1 / 96 Extended depth of field - completed : 2 / 96 Extended depth of field - completed : 3 / 96 Extended depth of field - completed : 4 / 96 Extended depth of field - completed : 5 / 96 Extended depth of field - completed : 6 / 96 Extended depth of field - completed : 7 / 96 Shading correction - completed : 0 / 72 Shading correction - completed : 1 / 72 Shading correction - completed : 2 / 72 Shading correction - completed : 3 / 72 Shading correction - completed : 4 / 72 Shading correction - completed : 5 / 72 Shading correction - completed : 6 / 72 Shading correction - completed : 7 / 72 Shading correction - completed : 8 / 72 Shading correction - completed : 9 / 72 Shading correction - completed : 10 / 72 Shading correction - completed : 11 / 72 Shading correction - completed : 12 / 72 Shading correction - completed : 13 / 72 Shading correction - completed : 14 / 72 Shading correction - completed : 15 / 72 Shading correction - completed : 16 / 72 Shading correction - completed : 17 / 72 Shading correction - completed : 18 / 72 Shading correction - completed : 19 / 72 Shading correction - completed : 20 / 72 Shading correction - completed : 21 / 72 Shading correction - completed : 22 / 72 Shading correction - completed : 23 / 72 Shading correction - completed : 24 / 72 Shading correction - completed : 25 / 72 Shading correction - completed : 26 / 72 Shading correction - completed : 27 / 72 Shading correction - completed : 28 / 72 Shading correction - completed : 29 / 72 Shading correction - completed : 30 / 72 Shading correction - completed : 31 / 72 Shading correction - completed : 32 / 72 Shading correction - completed : 33 / 72 Shading correction - completed : 34 / 72 Shading correction - completed : 35 / 72 Shading correction - completed : 36 / 72 Shading correction - completed : 37 / 72 Shading correction - completed : 38 / 72 Shading correction - completed : 39 / 72 Shading correction - completed : 40 / 72 Shading correction - completed : 41 / 72 Shading correction - completed : 42 / 72 Shading correction - completed : 43 / 72 Shading correction - completed : 44 / 72 Shading correction - completed : 45 / 72 Shading correction - completed : 46 / 72 Shading correction - completed : 47 / 72 Shading correction - completed : 48 / 72 Shading correction - completed : 49 / 72 Shading correction - completed : 50 / 72 Shading correction - completed : 51 / 72 Shading correction - completed : 52 / 72 Shading correction - completed : 53 / 72 Shading correction - completed : 54 / 72 Shading correction - completed : 55 / 72 Shading correction - completed : 56 / 72 Shading correction - completed : 57 / 72 Shading correction - completed : 58 / 72 Shading correction - completed : 59 / 72 Shading correction - completed : 60 / 72 Shading correction - completed : 61 / 72 Shading correction - completed : 62 / 72 Shading correction - completed : 63 / 72 Shading correction - completed : 64 / 72 Shading correction - completed : 65 / 72 Shading correction - completed : 66 / 72 Shading correction - completed : 67 / 72 Shading correction - completed : 68 / 72 Shading correction - completed : 69 / 72 Shading correction - completed : 70 / 72 Shading correction - completed : 71 / 72 Shading correction - completed : 72 / 72 Background subtraction - completed : 0 / 48 Background subtraction - completed : 1 / 48 Background subtraction - completed : 2 / 48 Background subtraction - completed : 3 / 48 Background subtraction - completed : 4 / 48 Background subtraction - completed : 5 / 48 Background subtraction - completed : 6 / 48 Background subtraction - completed : 7 / 48 Background subtraction - completed : 8 / 48 Background subtraction - completed : 9 / 48 Background subtraction - completed : 10 / 48 Background subtraction - completed : 11 / 48 Background subtraction - completed : 12 / 48 Background subtraction - completed : 13 / 48 Background subtraction - completed : 14 / 48 Background subtraction - completed : 15 / 48 Background subtraction - completed : 16 / 48 Background subtraction - completed : 17 / 48 Background subtraction - completed : 18 / 48 Background subtraction - completed : 19 / 48 Background subtraction - completed : 20 / 48 Background subtraction - completed : 21 / 48 Background subtraction - completed : 22 / 48 Background subtraction - completed : 23 / 48 Background subtraction - completed : 24 / 48 Background subtraction - completed : 25 / 48 Background subtraction - completed : 26 / 48 Background subtraction - completed : 27 / 48 Background subtraction - completed : 28 / 48 Background subtraction - completed : 29 / 48 Background subtraction - completed : 30 / 48 Background subtraction - completed : 31 / 48 Background subtraction - completed : 32 / 48 Background subtraction - completed : 33 / 48 Background subtraction - completed : 34 / 48 Background subtraction - completed : 35 / 48 Background subtraction - completed : 36 / 48 Background subtraction - completed : 37 / 48 Background subtraction - completed : 38 / 48 Background subtraction - completed : 39 / 48 Background subtraction - completed : 40 / 48 Background subtraction - completed : 41 / 48 Background subtraction - completed : 42 / 48 Background subtraction - completed : 43 / 48 Background subtraction - completed : 44 / 48 Background subtraction - completed : 45 / 48 Background subtraction - completed : 46 / 48 Background subtraction - completed : 47 / 48 Background subtraction - completed : 48 / 48 Cycle registration compute - completed : 0 / 20 Cycle registration compute - completed : 1 / 20 Cycle registration compute - completed : 2 / 20 Cycle registration compute - completed : 3 / 20 Cycle registration compute - completed : 4 / 20 Cycle registration compute - completed : 5 / 20 Cycle registration compute - completed : 6 / 20 Cycle registration compute - completed : 7 / 20 Cycle registration compute - completed : 8 / 20 Cycle registration compute - completed : 9 / 20 Cycle registration compute - completed : 10 / 20 Cycle registration compute - completed : 11 / 20 Cycle registration compute - completed : 12 / 20 Cycle registration compute - completed : 13 / 20 Cycle registration compute - completed : 14 / 20 Cycle registration compute - completed : 15 / 20 Cycle registration compute - completed : 16 / 20 Cycle registration compute - completed : 17 / 20 Cycle registration compute - completed : 18 / 20 Cycle registration compute - completed : 19 / 20 Cycle registration compute - completed : 20 / 20 Cycle registration apply - completed : 0 / 96 Cycle registration apply - completed : 1 / 96 Cycle registration apply - completed : 2 / 96 Cycle registration apply - completed : 3 / 96 Cycle registration apply - completed : 4 / 96 Cycle registration apply - completed : 5 / 96 Cycle registration apply - completed : 6 / 96 Cycle registration apply - completed : 7 / 96 Cycle registration apply - completed : 8 / 96 Cycle registration apply - completed : 9 / 96 Cycle registration apply - completed : 10 / 96 Cycle registration apply - completed : 11 / 96 Cycle registration apply - completed : 12 / 96 Cycle registration apply - completed : 13 / 96 Cycle registration apply - completed : 14 / 96 Cycle registration apply - completed : 15 / 96 Cycle registration apply - completed : 16 / 96 Cycle registration apply - completed : 17 / 96 Cycle registration apply - completed : 18 / 96 Cycle registration apply - completed : 19 / 96 Cycle registration apply - completed : 20 / 96 Cycle registration apply - completed : 21 / 96 Cycle registration apply - completed : 22 / 96 Cycle registration apply - completed : 23 / 96 Cycle registration apply - completed : 24 / 96 Cycle registration apply - completed : 25 / 96 Cycle registration apply - completed : 26 / 96 Cycle registration apply - completed : 27 / 96 Cycle registration apply - completed : 28 / 96 Cycle registration apply - completed : 29 / 96 Cycle registration apply - completed : 30 / 96 Cycle registration apply - completed : 31 / 96 Cycle registration apply - completed : 32 / 96 Cycle registration apply - completed : 33 / 96 Cycle registration apply - completed : 34 / 96 Cycle registration apply - completed : 35 / 96 Cycle registration apply - completed : 36 / 96 Cycle registration apply - completed : 37 / 96 Cycle registration apply - completed : 38 / 96 Cycle registration apply - completed : 39 / 96 Cycle registration apply - completed : 40 / 96 Cycle registration apply - completed : 41 / 96 Cycle registration apply - completed : 42 / 96 Cycle registration apply - completed : 43 / 96 Cycle registration apply - completed : 44 / 96 Cycle registration apply - completed : 45 / 96 Cycle registration apply - completed : 46 / 96 Cycle registration apply - completed : 47 / 96 Cycle registration apply - completed : 48 / 96 Cycle registration apply - completed : 49 / 96 Cycle registration apply - completed : 50 / 96 Cycle registration apply - completed : 51 / 96 Cycle registration apply - completed : 52 / 96 Cycle registration apply - completed : 53 / 96 Cycle registration apply - completed : 54 / 96 Cycle registration apply - completed : 55 / 96 Cycle registration apply - completed : 56 / 96 Cycle registration apply - completed : 57 / 96 Cycle registration apply - completed : 58 / 96 Cycle registration apply - completed : 59 / 96 Cycle registration apply - completed : 60 / 96 Cycle registration apply - completed : 61 / 96 Cycle registration apply - completed : 62 / 96 Cycle registration apply - completed : 63 / 96 Cycle registration apply - completed : 64 / 96 Cycle registration apply - completed : 65 / 96 Cycle registration apply - completed : 66 / 96 Cycle registration apply - completed : 67 / 96 Cycle registration apply - completed : 68 / 96 Cycle registration apply - completed : 69 / 96 Cycle registration apply - completed : 70 / 96 Cycle registration apply - completed : 71 / 96 Cycle registration apply - completed : 72 / 96 Cycle registration apply - completed : 73 / 96 Cycle registration apply - completed : 74 / 96 Cycle registration apply - completed : 75 / 96 Cycle registration apply - completed : 76 / 96 Cycle registration apply - completed : 77 / 96 Cycle registration apply - completed : 78 / 96 Cycle registration apply - completed : 79 / 96 Cycle registration apply - completed : 80 / 96 Cycle registration apply - completed : 81 / 96 Cycle registration apply - completed : 82 / 96 Cycle registration apply - completed : 83 / 96 Cycle registration apply - completed : 84 / 96 Cycle registration apply - completed : 85 / 96 Cycle registration apply - completed : 86 / 96 Cycle registration apply - completed : 87 / 96 Cycle registration apply - completed : 88 / 96 Cycle registration apply - completed : 89 / 96 Cycle registration apply - completed : 90 / 96 Cycle registration apply - completed : 91 / 96 Cycle registration apply - completed : 92 / 96 Cycle registration apply - completed : 93 / 96 Cycle registration apply - completed : 94 / 96 Cycle registration apply - completed : 95 / 96 Cycle registration apply - completed : 96 / 96 Remove negative values - completed : 0 / 24 Remove negative values - completed : 1 / 24 Remove negative values - completed : 2 / 24 Remove negative values - completed : 3 / 24 Remove negative values - completed : 4 / 24 Remove negative values - completed : 5 / 24 Remove negative values - completed : 6 / 24 Remove negative values - completed : 7 / 24 Remove negative values - completed : 8 / 24 Remove negative values - completed : 9 / 24 Remove negative values - completed : 10 / 24 Remove negative values - completed : 11 / 24 Remove negative values - completed : 12 / 24 Remove negative values - completed : 13 / 24 Remove negative values - completed : 14 / 24 Remove negative values - completed : 15 / 24 Remove negative values - completed : 16 / 24 Remove negative values - completed : 17 / 24 Remove negative values - completed : 18 / 24 Remove negative values - completed : 19 / 24 Remove negative values - completed : 20 / 24 Remove negative values - completed : 21 / 24 Remove negative values - completed : 22 / 24 Remove negative values - completed : 23 / 24 Remove negative values - completed : 24 / 24 gridwidth=2 gridheight=2 starttile=1 imagedir='C:\Users\IT-WL-annkl878\Documents\notes\Codex_Analysis\test_multiplxProcessor\out' filenamepatterntype=SEQUENTIAL filenamepattern={ppppp}_cy001_ch1.tif gridorigin=UL numberingpattern=HORIZONTALCONTINUOUS assemblefrommetadata=false globalpositionsfile='C:\Users\IT-WL-annkl878\Documents\notes\Codex_Analysis\test_multiplxProcessor\out\global-positions-0.txt' assemblenooverlap=false startrow=0 startcol=0 extentwidth=2 extentheight=2 timeslices=0 istimeslicesenabled=false outputpath='C:\Users\IT-WL-annkl878\Documents\notes\Codex_Analysis\test_multiplxProcessor\out' displaystitching=false outputfullimage=true outputmeta=true outputimgpyramid=false outfileprefix= blendingmode=LINEAR blendingalpha=1 numfftpeaks=100 isusedoubleprecision=true translationrefinementmethod=EXHAUSTIVE programtype=FFTW headless=true STITCHING BEGINS! Successfully loaded plan from file Loading FFTW Plan... Saving plan to file: C:\Users\IT-WL-annkl878\Documents\notes\Codex_Analysis\MultiplexProcessor\lib\fftw\fftPlans\2032x2032MeasurePlan.dat Finished loading/saving FFTW plan. Commencing stitching. memory pool size: 16 Writing relative positions (no optimization) to: C:\Users\IT-WL-annkl878\Documents\notes\Codex_Analysis\test_multiplxProcessor\out\relative-positions-no-optimization-0.txt Computed North overlap: 5.0% Computed West overlap: 4.0% Warning: no good translations found for North direction. Estimated translations generated from the overlap. Warning: no good translations found for North direction. Repeatability has been set to zero. Please check the statistics file for more details. Repeatability for North: 0 pixels Repeatability for West: 0 pixels Calculated Repeatability: 3 pixels Completed Stitching in 3864ms Writing global positions to: C:\Users\IT-WL-annkl878\Documents\notes\Codex_Analysis\test_multiplxProcessor\out\global-positions-0.txt Writing relative positions to: C:\Users\IT-WL-annkl878\Documents\notes\Codex_Analysis\test_multiplxProcessor\out\relative-positions-0.txt Writing relative positions (no optimization) to: C:\Users\IT-WL-annkl878\Documents\notes\Codex_Analysis\test_multiplxProcessor\out\relative-positions-no-optimization-0.txt Writing full image to: C:\Users\IT-WL-annkl878\Documents\notes\Codex_Analysis\test_multiplxProcessor\out\stitched-0.tif  Width: 3929 Height: 3965 Saving tiles to file: C:\Users\IT-WL-annkl878\Documents\notes\Codex_Analysis\test_multiplxProcessor\out\stitched-0.tif Finished saving full image: C:\Users\IT-WL-annkl878\Documents\notes\Codex_Analysis\test_multiplxProcessor\out\stitched-0.tif Completed output options for slice 0. Saving Statistics to "C:\Users\IT-WL-annkl878\Documents\notes\Codex_Analysis\test_multiplxProcessor\out\statistics.txt" Saving Log to "C:\Users\IT-WL-annkl878\Documents\notes\Codex_Analysis\test_multiplxProcessor\out\log.txt" Done gridwidth=2 gridheight=2 starttile=1 imagedir='C:\Users\IT-WL-annkl878\Documents\notes\Codex_Analysis\test_multiplxProcessor\out' filenamepatterntype=SEQUENTIAL filenamepattern={ppppp}_cy001_ch2.tif gridorigin=UL numberingpattern=HORIZONTALCONTINUOUS assemblefrommetadata=true globalpositionsfile='C:\Users\IT-WL-annkl878\Documents\notes\Codex_Analysis\test_multiplxProcessor\out\global-positions-0.txt' assemblenooverlap=false startrow=0 startcol=0 extentwidth=2 extentheight=2 timeslices=0 istimeslicesenabled=false outputpath='C:\Users\IT-WL-annkl878\Documents\notes\Codex_Analysis\test_multiplxProcessor\out' displaystitching=false outputfullimage=true outputmeta=true outputimgpyramid=false outfileprefix= blendingmode=LINEAR blendingalpha=1 numfftpeaks=100 isusedoubleprecision=true translationrefinementmethod=EXHAUSTIVE programtype=FFTW headless=true Checking args for stitching: Arg check passed Warning: the following files will be overwritten: C:\Users\IT-WL-annkl878\Documents\notes\Codex_Analysis\test_multiplxProcessor\out\log.txt C:\Users\IT-WL-annkl878\Documents\notes\Codex_Analysis\test_multiplxProcessor\out\statistics.txt STITCHING BEGINS! Completed Stitching in 13ms Writing full image to: C:\Users\IT-WL-annkl878\Documents\notes\Codex_Analysis\test_multiplxProcessor\out\stitched-0.tif  Width: 3929 Height: 3965 Saving tiles to file: C:\Users\IT-WL-annkl878\Documents\notes\Codex_Analysis\test_multiplxProcessor\out\stitched-0.tif Finished saving full image: C:\Users\IT-WL-annkl878\Documents\notes\Codex_Analysis\test_multiplxProcessor\out\stitched-0.tif Completed output options for slice 0. Done gridwidth=2 gridheight=2 starttile=1 imagedir='C:\Users\IT-WL-annkl878\Documents\notes\Codex_Analysis\test_multiplxProcessor\out' filenamepatterntype=SEQUENTIAL filenamepattern={ppppp}_cy001_ch3.tif gridorigin=UL numberingpattern=HORIZONTALCONTINUOUS assemblefrommetadata=true globalpositionsfile='C:\Users\IT-WL-annkl878\Documents\notes\Codex_Analysis\test_multiplxProcessor\out\global-positions-0.txt' assemblenooverlap=false startrow=0 startcol=0 extentwidth=2 extentheight=2 timeslices=0 istimeslicesenabled=false outputpath='C:\Users\IT-WL-annkl878\Documents\notes\Codex_Analysis\test_multiplxProcessor\out' displaystitching=false outputfullimage=true outputmeta=true outputimgpyramid=false outfileprefix= blendingmode=LINEAR blendingalpha=1 numfftpeaks=100 isusedoubleprecision=true translationrefinementmethod=EXHAUSTIVE programtype=FFTW headless=true Checking args for stitching: Arg check passed Warning: the following files will be overwritten: C:\Users\IT-WL-annkl878\Documents\notes\Codex_Analysis\test_multiplxProcessor\out\log.txt C:\Users\IT-WL-annkl878\Documents\notes\Codex_Analysis\test_multiplxProcessor\out\statistics.txt STITCHING BEGINS! Completed Stitching in 1ms Writing full image to: C:\Users\IT-WL-annkl878\Documents\notes\Codex_Analysis\test_multiplxProcessor\out\stitched-0.tif  Width: 3929 Height: 3965 Saving tiles to file: C:\Users\IT-WL-annkl878\Documents\notes\Codex_Analysis\test_multiplxProcessor\out\stitched-0.tif Finished saving full image: C:\Users\IT-WL-annkl878\Documents\notes\Codex_Analysis\test_multiplxProcessor\out\stitched-0.tif Completed output options for slice 0. Done gridwidth=2 gridheight=2 starttile=1 imagedir='C:\Users\IT-WL-annkl878\Documents\notes\Codex_Analysis\test_multiplxProcessor\out' filenamepatterntype=SEQUENTIAL filenamepattern={ppppp}_cy001_ch4.tif gridorigin=UL numberingpattern=HORIZONTALCONTINUOUS assemblefrommetadata=true globalpositionsfile='C:\Users\IT-WL-annkl878\Documents\notes\Codex_Analysis\test_multiplxProcessor\out\global-positions-0.txt' assemblenooverlap=false startrow=0 startcol=0 extentwidth=2 extentheight=2 timeslices=0 istimeslicesenabled=false outputpath='C:\Users\IT-WL-annkl878\Documents\notes\Codex_Analysis\test_multiplxProcessor\out' displaystitching=false outputfullimage=true outputmeta=true outputimgpyramid=false outfileprefix= blendingmode=LINEAR blendingalpha=1 numfftpeaks=100 isusedoubleprecision=true translationrefinementmethod=EXHAUSTIVE programtype=FFTW headless=true Checking args for stitching: Arg check passed Warning: the following files will be overwritten: C:\Users\IT-WL-annkl878\Documents\notes\Codex_Analysis\test_multiplxProcessor\out\log.txt C:\Users\IT-WL-annkl878\Documents\notes\Codex_Analysis\test_multiplxProcessor\out\statistics.txt STITCHING BEGINS! Completed Stitching in 0ms Writing full image to: C:\Users\IT-WL-annkl878\Documents\notes\Codex_Analysis\test_multiplxProcessor\out\stitched-0.tif  Width: 3929 Height: 3965 Saving tiles to file: C:\Users\IT-WL-annkl878\Documents\notes\Codex_Analysis\test_multiplxProcessor\out\stitched-0.tif Finished saving full image: C:\Users\IT-WL-annkl878\Documents\notes\Codex_Analysis\test_multiplxProcessor\out\stitched-0.tif Completed output options for slice 0. Done gridwidth=2 gridheight=2 starttile=1 imagedir='C:\Users\IT-WL-annkl878\Documents\notes\Codex_Analysis\test_multiplxProcessor\out' filenamepatterntype=SEQUENTIAL filenamepattern={ppppp}_cy002_ch1.tif gridorigin=UL numberingpattern=HORIZONTALCONTINUOUS assemblefrommetadata=true globalpositionsfile='C:\Users\IT-WL-annkl878\Documents\notes\Codex_Analysis\test_multiplxProcessor\out\global-positions-0.txt' assemblenooverlap=false startrow=0 startcol=0 extentwidth=2 extentheight=2 timeslices=0 istimeslicesenabled=false outputpath='C:\Users\IT-WL-annkl878\Documents\notes\Codex_Analysis\test_multiplxProcessor\out' displaystitching=false outputfullimage=true outputmeta=true outputimgpyramid=false outfileprefix= blendingmode=LINEAR blendingalpha=1 numfftpeaks=100 isusedoubleprecision=true translationrefinementmethod=EXHAUSTIVE programtype=FFTW headless=true Checking args for stitching: Arg check passed Warning: the following files will be overwritten: C:\Users\IT-WL-annkl878\Documents\notes\Codex_Analysis\test_multiplxProcessor\out\log.txt C:\Users\IT-WL-annkl878\Documents\notes\Codex_Analysis\test_multiplxProcessor\out\statistics.txt STITCHING BEGINS! Completed Stitching in 1ms Writing full image to: C:\Users\IT-WL-annkl878\Documents\notes\Codex_Analysis\test_multiplxProcessor\out\stitched-0.tif  Width: 3929 Height: 3965 Saving tiles to file: C:\Users\IT-WL-annkl878\Documents\notes\Codex_Analysis\test_multiplxProcessor\out\stitched-0.tif Finished saving full image: C:\Users\IT-WL-annkl878\Documents\notes\Codex_Analysis\test_multiplxProcessor\out\stitched-0.tif Completed output options for slice 0. Done gridwidth=2 gridheight=2 starttile=1 imagedir='C:\Users\IT-WL-annkl878\Documents\notes\Codex_Analysis\test_multiplxProcessor\out' filenamepatterntype=SEQUENTIAL filenamepattern={ppppp}_cy002_ch2.tif gridorigin=UL numberingpattern=HORIZONTALCONTINUOUS assemblefrommetadata=true globalpositionsfile='C:\Users\IT-WL-annkl878\Documents\notes\Codex_Analysis\test_multiplxProcessor\out\global-positions-0.txt' assemblenooverlap=false startrow=0 startcol=0 extentwidth=2 extentheight=2 timeslices=0 istimeslicesenabled=false outputpath='C:\Users\IT-WL-annkl878\Documents\notes\Codex_Analysis\test_multiplxProcessor\out' displaystitching=false outputfullimage=true outputmeta=true outputimgpyramid=false outfileprefix= blendingmode=LINEAR blendingalpha=1 numfftpeaks=100 isusedoubleprecision=true translationrefinementmethod=EXHAUSTIVE programtype=FFTW headless=true Checking args for stitching: Arg check passed Warning: the following files will be overwritten: C:\Users\IT-WL-annkl878\Documents\notes\Codex_Analysis\test_multiplxProcessor\out\log.txt C:\Users\IT-WL-annkl878\Documents\notes\Codex_Analysis\test_multiplxProcessor\out\statistics.txt STITCHING BEGINS! Completed Stitching in 2ms Writing full image to: C:\Users\IT-WL-annkl878\Documents\notes\Codex_Analysis\test_multiplxProcessor\out\stitched-0.tif  Width: 3929 Height: 3965 Saving tiles to file: C:\Users\IT-WL-annkl878\Documents\notes\Codex_Analysis\test_multiplxProcessor\out\stitched-0.tif Finished saving full image: C:\Users\IT-WL-annkl878\Documents\notes\Codex_Analysis\test_multiplxProcessor\out\stitched-0.tif Completed output options for slice 0. Done gridwidth=2 gridheight=2 starttile=1 imagedir='C:\Users\IT-WL-annkl878\Documents\notes\Codex_Analysis\test_multiplxProcessor\out' filenamepatterntype=SEQUENTIAL filenamepattern={ppppp}_cy002_ch3.tif gridorigin=UL numberingpattern=HORIZONTALCONTINUOUS assemblefrommetadata=true globalpositionsfile='C:\Users\IT-WL-annkl878\Documents\notes\Codex_Analysis\test_multiplxProcessor\out\global-positions-0.txt' assemblenooverlap=false startrow=0 startcol=0 extentwidth=2 extentheight=2 timeslices=0 istimeslicesenabled=false outputpath='C:\Users\IT-WL-annkl878\Documents\notes\Codex_Analysis\test_multiplxProcessor\out' displaystitching=false outputfullimage=true outputmeta=true outputimgpyramid=false outfileprefix= blendingmode=LINEAR blendingalpha=1 numfftpeaks=100 isusedoubleprecision=true translationrefinementmethod=EXHAUSTIVE programtype=FFTW headless=true Checking args for stitching: Arg check passed Warning: the following files will be overwritten: C:\Users\IT-WL-annkl878\Documents\notes\Codex_Analysis\test_multiplxProcessor\out\log.txt C:\Users\IT-WL-annkl878\Documents\notes\Codex_Analysis\test_multiplxProcessor\out\statistics.txt STITCHING BEGINS! Completed Stitching in 2ms Writing full image to: C:\Users\IT-WL-annkl878\Documents\notes\Codex_Analysis\test_multiplxProcessor\out\stitched-0.tif  Width: 3929 Height: 3965 Saving tiles to file: C:\Users\IT-WL-annkl878\Documents\notes\Codex_Analysis\test_multiplxProcessor\out\stitched-0.tif Finished saving full image: C:\Users\IT-WL-annkl878\Documents\notes\Codex_Analysis\test_multiplxProcessor\out\stitched-0.tif Completed output options for slice 0. Done gridwidth=2 gridheight=2 starttile=1 imagedir='C:\Users\IT-WL-annkl878\Documents\notes\Codex_Analysis\test_multiplxProcessor\out' filenamepatterntype=SEQUENTIAL filenamepattern={ppppp}_cy002_ch4.tif gridorigin=UL numberingpattern=HORIZONTALCONTINUOUS assemblefrommetadata=true globalpositionsfile='C:\Users\IT-WL-annkl878\Documents\notes\Codex_Analysis\test_multiplxProcessor\out\global-positions-0.txt' assemblenooverlap=false startrow=0 startcol=0 extentwidth=2 extentheight=2 timeslices=0 istimeslicesenabled=false outputpath='C:\Users\IT-WL-annkl878\Documents\notes\Codex_Analysis\test_multiplxProcessor\out' displaystitching=false outputfullimage=true outputmeta=true outputimgpyramid=false outfileprefix= blendingmode=LINEAR blendingalpha=1 numfftpeaks=100 isusedoubleprecision=true translationrefinementmethod=EXHAUSTIVE programtype=FFTW headless=true Checking args for stitching: Arg check passed Warning: the following files will be overwritten: C:\Users\IT-WL-annkl878\Documents\notes\Codex_Analysis\test_multiplxProcessor\out\log.txt C:\Users\IT-WL-annkl878\Documents\notes\Codex_Analysis\test_multiplxProcessor\out\statistics.txt STITCHING BEGINS! Completed Stitching in 0ms Writing full image to: C:\Users\IT-WL-annkl878\Documents\notes\Codex_Analysis\test_multiplxProcessor\out\stitched-0.tif  Width: 3929 Height: 3965 Saving tiles to file: C:\Users\IT-WL-annkl878\Documents\notes\Codex_Analysis\test_multiplxProcessor\out\stitched-0.tif Finished saving full image: C:\Users\IT-WL-annkl878\Documents\notes\Codex_Analysis\test_multiplxProcessor\out\stitched-0.tif Completed output options for slice 0. Done gridwidth=2 gridheight=2 starttile=1 imagedir='C:\Users\IT-WL-annkl878\Documents\notes\Codex_Analysis\test_multiplxProcessor\out' filenamepatterntype=SEQUENTIAL filenamepattern={ppppp}_cy003_ch1.tif gridorigin=UL numberingpattern=HORIZONTALCONTINUOUS assemblefrommetadata=true globalpositionsfile='C:\Users\IT-WL-annkl878\Documents\notes\Codex_Analysis\test_multiplxProcessor\out\global-positions-0.txt' assemblenooverlap=false startrow=0 startcol=0 extentwidth=2 extentheight=2 timeslices=0 istimeslicesenabled=false outputpath='C:\Users\IT-WL-annkl878\Documents\notes\Codex_Analysis\test_multiplxProcessor\out' displaystitching=false outputfullimage=true outputmeta=true outputimgpyramid=false outfileprefix= blendingmode=LINEAR blendingalpha=1 numfftpeaks=100 isusedoubleprecision=true translationrefinementmethod=EXHAUSTIVE programtype=FFTW headless=true Checking args for stitching: Arg check passed Warning: the following files will be overwritten: C:\Users\IT-WL-annkl878\Documents\notes\Codex_Analysis\test_multiplxProcessor\out\log.txt C:\Users\IT-WL-annkl878\Documents\notes\Codex_Analysis\test_multiplxProcessor\out\statistics.txt STITCHING BEGINS! Completed Stitching in 1ms Writing full image to: C:\Users\IT-WL-annkl878\Documents\notes\Codex_Analysis\test_multiplxProcessor\out\stitched-0.tif  Width: 3929 Height: 3965 Saving tiles to file: C:\Users\IT-WL-annkl878\Documents\notes\Codex_Analysis\test_multiplxProcessor\out\stitched-0.tif Finished saving full image: C:\Users\IT-WL-annkl878\Documents\notes\Codex_Analysis\test_multiplxProcessor\out\stitched-0.tif Completed output options for slice 0. Done gridwidth=2 gridheight=2 starttile=1 imagedir='C:\Users\IT-WL-annkl878\Documents\notes\Codex_Analysis\test_multiplxProcessor\out' filenamepatterntype=SEQUENTIAL filenamepattern={ppppp}_cy003_ch2.tif gridorigin=UL numberingpattern=HORIZONTALCONTINUOUS assemblefrommetadata=true globalpositionsfile='C:\Users\IT-WL-annkl878\Documents\notes\Codex_Analysis\test_multiplxProcessor\out\global-positions-0.txt' assemblenooverlap=false startrow=0 startcol=0 extentwidth=2 extentheight=2 timeslices=0 istimeslicesenabled=false outputpath='C:\Users\IT-WL-annkl878\Documents\notes\Codex_Analysis\test_multiplxProcessor\out' displaystitching=false outputfullimage=true outputmeta=true outputimgpyramid=false outfileprefix= blendingmode=LINEAR blendingalpha=1 numfftpeaks=100 isusedoubleprecision=true translationrefinementmethod=EXHAUSTIVE programtype=FFTW headless=true Checking args for stitching: Arg check passed Warning: the following files will be overwritten: C:\Users\IT-WL-annkl878\Documents\notes\Codex_Analysis\test_multiplxProcessor\out\log.txt C:\Users\IT-WL-annkl878\Documents\notes\Codex_Analysis\test_multiplxProcessor\out\statistics.txt STITCHING BEGINS! Completed Stitching in 1ms Writing full image to: C:\Users\IT-WL-annkl878\Documents\notes\Codex_Analysis\test_multiplxProcessor\out\stitched-0.tif  Width: 3929 Height: 3965 Saving tiles to file: C:\Users\IT-WL-annkl878\Documents\notes\Codex_Analysis\test_multiplxProcessor\out\stitched-0.tif Finished saving full image: C:\Users\IT-WL-annkl878\Documents\notes\Codex_Analysis\test_multiplxProcessor\out\stitched-0.tif Completed output options for slice 0. Done gridwidth=2 gridheight=2 starttile=1 imagedir='C:\Users\IT-WL-annkl878\Documents\notes\Codex_Analysis\test_multiplxProcessor\out' filenamepatterntype=SEQUENTIAL filenamepattern={ppppp}_cy003_ch3.tif gridorigin=UL numberingpattern=HORIZONTALCONTINUOUS assemblefrommetadata=true globalpositionsfile='C:\Users\IT-WL-annkl878\Documents\notes\Codex_Analysis\test_multiplxProcessor\out\global-positions-0.txt' assemblenooverlap=false startrow=0 startcol=0 extentwidth=2 extentheight=2 timeslices=0 istimeslicesenabled=false outputpath='C:\Users\IT-WL-annkl878\Documents\notes\Codex_Analysis\test_multiplxProcessor\out' displaystitching=false outputfullimage=true outputmeta=true outputimgpyramid=false outfileprefix= blendingmode=LINEAR blendingalpha=1 numfftpeaks=100 isusedoubleprecision=true translationrefinementmethod=EXHAUSTIVE programtype=FFTW headless=true Checking args for stitching: Arg check passed Warning: the following files will be overwritten: C:\Users\IT-WL-annkl878\Documents\notes\Codex_Analysis\test_multiplxProcessor\out\log.txt C:\Users\IT-WL-annkl878\Documents\notes\Codex_Analysis\test_multiplxProcessor\out\statistics.txt STITCHING BEGINS! Completed Stitching in 1ms Writing full image to: C:\Users\IT-WL-annkl878\Documents\notes\Codex_Analysis\test_multiplxProcessor\out\stitched-0.tif  Width: 3929 Height: 3965 Saving tiles to file: C:\Users\IT-WL-annkl878\Documents\notes\Codex_Analysis\test_multiplxProcessor\out\stitched-0.tif Finished saving full image: C:\Users\IT-WL-annkl878\Documents\notes\Codex_Analysis\test_multiplxProcessor\out\stitched-0.tif Completed output options for slice 0. Done gridwidth=2 gridheight=2 starttile=1 imagedir='C:\Users\IT-WL-annkl878\Documents\notes\Codex_Analysis\test_multiplxProcessor\out' filenamepatterntype=SEQUENTIAL filenamepattern={ppppp}_cy003_ch4.tif gridorigin=UL numberingpattern=HORIZONTALCONTINUOUS assemblefrommetadata=true globalpositionsfile='C:\Users\IT-WL-annkl878\Documents\notes\Codex_Analysis\test_multiplxProcessor\out\global-positions-0.txt' assemblenooverlap=false startrow=0 startcol=0 extentwidth=2 extentheight=2 timeslices=0 istimeslicesenabled=false outputpath='C:\Users\IT-WL-annkl878\Documents\notes\Codex_Analysis\test_multiplxProcessor\out' displaystitching=false outputfullimage=true outputmeta=true outputimgpyramid=false outfileprefix= blendingmode=LINEAR blendingalpha=1 numfftpeaks=100 isusedoubleprecision=true translationrefinementmethod=EXHAUSTIVE programtype=FFTW headless=true Checking args for stitching: Arg check passed Warning: the following files will be overwritten: C:\Users\IT-WL-annkl878\Documents\notes\Codex_Analysis\test_multiplxProcessor\out\log.txt C:\Users\IT-WL-annkl878\Documents\notes\Codex_Analysis\test_multiplxProcessor\out\statistics.txt STITCHING BEGINS! Completed Stitching in 1ms Writing full image to: C:\Users\IT-WL-annkl878\Documents\notes\Codex_Analysis\test_multiplxProcessor\out\stitched-0.tif  Width: 3929 Height: 3965 Saving tiles to file: C:\Users\IT-WL-annkl878\Documents\notes\Codex_Analysis\test_multiplxProcessor\out\stitched-0.tif Finished saving full image: C:\Users\IT-WL-annkl878\Documents\notes\Codex_Analysis\test_multiplxProcessor\out\stitched-0.tif Completed output options for slice 0. Done gridwidth=2 gridheight=2 starttile=1 imagedir='C:\Users\IT-WL-annkl878\Documents\notes\Codex_Analysis\test_multiplxProcessor\out' filenamepatterntype=SEQUENTIAL filenamepattern={ppppp}_cy004_ch1.tif gridorigin=UL numberingpattern=HORIZONTALCONTINUOUS assemblefrommetadata=true globalpositionsfile='C:\Users\IT-WL-annkl878\Documents\notes\Codex_Analysis\test_multiplxProcessor\out\global-positions-0.txt' assemblenooverlap=false startrow=0 startcol=0 extentwidth=2 extentheight=2 timeslices=0 istimeslicesenabled=false outputpath='C:\Users\IT-WL-annkl878\Documents\notes\Codex_Analysis\test_multiplxProcessor\out' displaystitching=false outputfullimage=true outputmeta=true outputimgpyramid=false outfileprefix= blendingmode=LINEAR blendingalpha=1 numfftpeaks=100 isusedoubleprecision=true translationrefinementmethod=EXHAUSTIVE programtype=FFTW headless=true Checking args for stitching: Arg check passed Warning: the following files will be overwritten: C:\Users\IT-WL-annkl878\Documents\notes\Codex_Analysis\test_multiplxProcessor\out\log.txt C:\Users\IT-WL-annkl878\Documents\notes\Codex_Analysis\test_multiplxProcessor\out\statistics.txt STITCHING BEGINS! Completed Stitching in 1ms Writing full image to: C:\Users\IT-WL-annkl878\Documents\notes\Codex_Analysis\test_multiplxProcessor\out\stitched-0.tif  Width: 3929 Height: 3965 Saving tiles to file: C:\Users\IT-WL-annkl878\Documents\notes\Codex_Analysis\test_multiplxProcessor\out\stitched-0.tif Finished saving full image: C:\Users\IT-WL-annkl878\Documents\notes\Codex_Analysis\test_multiplxProcessor\out\stitched-0.tif Completed output options for slice 0. Done gridwidth=2 gridheight=2 starttile=1 imagedir='C:\Users\IT-WL-annkl878\Documents\notes\Codex_Analysis\test_multiplxProcessor\out' filenamepatterntype=SEQUENTIAL filenamepattern={ppppp}_cy004_ch2.tif gridorigin=UL numberingpattern=HORIZONTALCONTINUOUS assemblefrommetadata=true globalpositionsfile='C:\Users\IT-WL-annkl878\Documents\notes\Codex_Analysis\test_multiplxProcessor\out\global-positions-0.txt' assemblenooverlap=false startrow=0 startcol=0 extentwidth=2 extentheight=2 timeslices=0 istimeslicesenabled=false outputpath='C:\Users\IT-WL-annkl878\Documents\notes\Codex_Analysis\test_multiplxProcessor\out' displaystitching=false outputfullimage=true outputmeta=true outputimgpyramid=false outfileprefix= blendingmode=LINEAR blendingalpha=1 numfftpeaks=100 isusedoubleprecision=true translationrefinementmethod=EXHAUSTIVE programtype=FFTW headless=true Checking args for stitching: Arg check passed Warning: the following files will be overwritten: C:\Users\IT-WL-annkl878\Documents\notes\Codex_Analysis\test_multiplxProcessor\out\log.txt C:\Users\IT-WL-annkl878\Documents\notes\Codex_Analysis\test_multiplxProcessor\out\statistics.txt STITCHING BEGINS! Completed Stitching in 1ms Writing full image to: C:\Users\IT-WL-annkl878\Documents\notes\Codex_Analysis\test_multiplxProcessor\out\stitched-0.tif  Width: 3929 Height: 3965 Saving tiles to file: C:\Users\IT-WL-annkl878\Documents\notes\Codex_Analysis\test_multiplxProcessor\out\stitched-0.tif Finished saving full image: C:\Users\IT-WL-annkl878\Documents\notes\Codex_Analysis\test_multiplxProcessor\out\stitched-0.tif Completed output options for slice 0. Done gridwidth=2 gridheight=2 starttile=1 imagedir='C:\Users\IT-WL-annkl878\Documents\notes\Codex_Analysis\test_multiplxProcessor\out' filenamepatterntype=SEQUENTIAL filenamepattern={ppppp}_cy004_ch3.tif gridorigin=UL numberingpattern=HORIZONTALCONTINUOUS assemblefrommetadata=true globalpositionsfile='C:\Users\IT-WL-annkl878\Documents\notes\Codex_Analysis\test_multiplxProcessor\out\global-positions-0.txt' assemblenooverlap=false startrow=0 startcol=0 extentwidth=2 extentheight=2 timeslices=0 istimeslicesenabled=false outputpath='C:\Users\IT-WL-annkl878\Documents\notes\Codex_Analysis\test_multiplxProcessor\out' displaystitching=false outputfullimage=true outputmeta=true outputimgpyramid=false outfileprefix= blendingmode=LINEAR blendingalpha=1 numfftpeaks=100 isusedoubleprecision=true translationrefinementmethod=EXHAUSTIVE programtype=FFTW headless=true Checking args for stitching: Arg check passed Warning: the following files will be overwritten: C:\Users\IT-WL-annkl878\Documents\notes\Codex_Analysis\test_multiplxProcessor\out\log.txt C:\Users\IT-WL-annkl878\Documents\notes\Codex_Analysis\test_multiplxProcessor\out\statistics.txt STITCHING BEGINS! Completed Stitching in 1ms Writing full image to: C:\Users\IT-WL-annkl878\Documents\notes\Codex_Analysis\test_multiplxProcessor\out\stitched-0.tif  Width: 3929 Height: 3965 Saving tiles to file: C:\Users\IT-WL-annkl878\Documents\notes\Codex_Analysis\test_multiplxProcessor\out\stitched-0.tif Finished saving full image: C:\Users\IT-WL-annkl878\Documents\notes\Codex_Analysis\test_multiplxProcessor\out\stitched-0.tif Completed output options for slice 0. Done gridwidth=2 gridheight=2 starttile=1 imagedir='C:\Users\IT-WL-annkl878\Documents\notes\Codex_Analysis\test_multiplxProcessor\out' filenamepatterntype=SEQUENTIAL filenamepattern={ppppp}_cy004_ch4.tif gridorigin=UL numberingpattern=HORIZONTALCONTINUOUS assemblefrommetadata=true globalpositionsfile='C:\Users\IT-WL-annkl878\Documents\notes\Codex_Analysis\test_multiplxProcessor\out\global-positions-0.txt' assemblenooverlap=false startrow=0 startcol=0 extentwidth=2 extentheight=2 timeslices=0 istimeslicesenabled=false outputpath='C:\Users\IT-WL-annkl878\Documents\notes\Codex_Analysis\test_multiplxProcessor\out' displaystitching=false outputfullimage=true outputmeta=true outputimgpyramid=false outfileprefix= blendingmode=LINEAR blendingalpha=1 numfftpeaks=100 isusedoubleprecision=true translationrefinementmethod=EXHAUSTIVE programtype=FFTW headless=true Checking args for stitching: Arg check passed Warning: the following files will be overwritten: C:\Users\IT-WL-annkl878\Documents\notes\Codex_Analysis\test_multiplxProcessor\out\log.txt C:\Users\IT-WL-annkl878\Documents\notes\Codex_Analysis\test_multiplxProcessor\out\statistics.txt STITCHING BEGINS! Completed Stitching in 1ms Writing full image to: C:\Users\IT-WL-annkl878\Documents\notes\Codex_Analysis\test_multiplxProcessor\out\stitched-0.tif  Width: 3929 Height: 3965 Saving tiles to file: C:\Users\IT-WL-annkl878\Documents\notes\Codex_Analysis\test_multiplxProcessor\out\stitched-0.tif Finished saving full image: C:\Users\IT-WL-annkl878\Documents\notes\Codex_Analysis\test_multiplxProcessor\out\stitched-0.tif Completed output options for slice 0. Done gridwidth=2 gridheight=2 starttile=1 imagedir='C:\Users\IT-WL-annkl878\Documents\notes\Codex_Analysis\test_multiplxProcessor\out' filenamepatterntype=SEQUENTIAL filenamepattern={ppppp}_cy005_ch1.tif gridorigin=UL numberingpattern=HORIZONTALCONTINUOUS assemblefrommetadata=true globalpositionsfile='C:\Users\IT-WL-annkl878\Documents\notes\Codex_Analysis\test_multiplxProcessor\out\global-positions-0.txt' assemblenooverlap=false startrow=0 startcol=0 extentwidth=2 extentheight=2 timeslices=0 istimeslicesenabled=false outputpath='C:\Users\IT-WL-annkl878\Documents\notes\Codex_Analysis\test_multiplxProcessor\out' displaystitching=false outputfullimage=true outputmeta=true outputimgpyramid=false outfileprefix= blendingmode=LINEAR blendingalpha=1 numfftpeaks=100 isusedoubleprecision=true translationrefinementmethod=EXHAUSTIVE programtype=FFTW headless=true Checking args for stitching: Arg check passed Warning: the following files will be overwritten: C:\Users\IT-WL-annkl878\Documents\notes\Codex_Analysis\test_multiplxProcessor\out\log.txt C:\Users\IT-WL-annkl878\Documents\notes\Codex_Analysis\test_multiplxProcessor\out\statistics.txt STITCHING BEGINS! Completed Stitching in 0ms Writing full image to: C:\Users\IT-WL-annkl878\Documents\notes\Codex_Analysis\test_multiplxProcessor\out\stitched-0.tif  Width: 3929 Height: 3965 Saving tiles to file: C:\Users\IT-WL-annkl878\Documents\notes\Codex_Analysis\test_multiplxProcessor\out\stitched-0.tif Finished saving full image: C:\Users\IT-WL-annkl878\Documents\notes\Codex_Analysis\test_multiplxProcessor\out\stitched-0.tif Completed output options for slice 0. Done gridwidth=2 gridheight=2 starttile=1 imagedir='C:\Users\IT-WL-annkl878\Documents\notes\Codex_Analysis\test_multiplxProcessor\out' filenamepatterntype=SEQUENTIAL filenamepattern={ppppp}_cy005_ch2.tif gridorigin=UL numberingpattern=HORIZONTALCONTINUOUS assemblefrommetadata=true globalpositionsfile='C:\Users\IT-WL-annkl878\Documents\notes\Codex_Analysis\test_multiplxProcessor\out\global-positions-0.txt' assemblenooverlap=false startrow=0 startcol=0 extentwidth=2 extentheight=2 timeslices=0 istimeslicesenabled=false outputpath='C:\Users\IT-WL-annkl878\Documents\notes\Codex_Analysis\test_multiplxProcessor\out' displaystitching=false outputfullimage=true outputmeta=true outputimgpyramid=false outfileprefix= blendingmode=LINEAR blendingalpha=1 numfftpeaks=100 isusedoubleprecision=true translationrefinementmethod=EXHAUSTIVE programtype=FFTW headless=true Checking args for stitching: Arg check passed Warning: the following files will be overwritten: C:\Users\IT-WL-annkl878\Documents\notes\Codex_Analysis\test_multiplxProcessor\out\log.txt C:\Users\IT-WL-annkl878\Documents\notes\Codex_Analysis\test_multiplxProcessor\out\statistics.txt STITCHING BEGINS! Completed Stitching in 1ms Writing full image to: C:\Users\IT-WL-annkl878\Documents\notes\Codex_Analysis\test_multiplxProcessor\out\stitched-0.tif  Width: 3929 Height: 3965 Saving tiles to file: C:\Users\IT-WL-annkl878\Documents\notes\Codex_Analysis\test_multiplxProcessor\out\stitched-0.tif Finished saving full image: C:\Users\IT-WL-annkl878\Documents\notes\Codex_Analysis\test_multiplxProcessor\out\stitched-0.tif Completed output options for slice 0. Done gridwidth=2 gridheight=2 starttile=1 imagedir='C:\Users\IT-WL-annkl878\Documents\notes\Codex_Analysis\test_multiplxProcessor\out' filenamepatterntype=SEQUENTIAL filenamepattern={ppppp}_cy005_ch3.tif gridorigin=UL numberingpattern=HORIZONTALCONTINUOUS assemblefrommetadata=true globalpositionsfile='C:\Users\IT-WL-annkl878\Documents\notes\Codex_Analysis\test_multiplxProcessor\out\global-positions-0.txt' assemblenooverlap=false startrow=0 startcol=0 extentwidth=2 extentheight=2 timeslices=0 istimeslicesenabled=false outputpath='C:\Users\IT-WL-annkl878\Documents\notes\Codex_Analysis\test_multiplxProcessor\out' displaystitching=false outputfullimage=true outputmeta=true outputimgpyramid=false outfileprefix= blendingmode=LINEAR blendingalpha=1 numfftpeaks=100 isusedoubleprecision=true translationrefinementmethod=EXHAUSTIVE programtype=FFTW headless=true Checking args for stitching: Arg check passed Warning: the following files will be overwritten: C:\Users\IT-WL-annkl878\Documents\notes\Codex_Analysis\test_multiplxProcessor\out\log.txt C:\Users\IT-WL-annkl878\Documents\notes\Codex_Analysis\test_multiplxProcessor\out\statistics.txt STITCHING BEGINS! Completed Stitching in 1ms Writing full image to: C:\Users\IT-WL-annkl878\Documents\notes\Codex_Analysis\test_multiplxProcessor\out\stitched-0.tif  Width: 3929 Height: 3965 Saving tiles to file: C:\Users\IT-WL-annkl878\Documents\notes\Codex_Analysis\test_multiplxProcessor\out\stitched-0.tif Finished saving full image: C:\Users\IT-WL-annkl878\Documents\notes\Codex_Analysis\test_multiplxProcessor\out\stitched-0.tif Completed output options for slice 0. Done gridwidth=2 gridheight=2 starttile=1 imagedir='C:\Users\IT-WL-annkl878\Documents\notes\Codex_Analysis\test_multiplxProcessor\out' filenamepatterntype=SEQUENTIAL filenamepattern={ppppp}_cy005_ch4.tif gridorigin=UL numberingpattern=HORIZONTALCONTINUOUS assemblefrommetadata=true globalpositionsfile='C:\Users\IT-WL-annkl878\Documents\notes\Codex_Analysis\test_multiplxProcessor\out\global-positions-0.txt' assemblenooverlap=false startrow=0 startcol=0 extentwidth=2 extentheight=2 timeslices=0 istimeslicesenabled=false outputpath='C:\Users\IT-WL-annkl878\Documents\notes\Codex_Analysis\test_multiplxProcessor\out' displaystitching=false outputfullimage=true outputmeta=true outputimgpyramid=false outfileprefix= blendingmode=LINEAR blendingalpha=1 numfftpeaks=100 isusedoubleprecision=true translationrefinementmethod=EXHAUSTIVE programtype=FFTW headless=true Checking args for stitching: Arg check passed Warning: the following files will be overwritten: C:\Users\IT-WL-annkl878\Documents\notes\Codex_Analysis\test_multiplxProcessor\out\log.txt C:\Users\IT-WL-annkl878\Documents\notes\Codex_Analysis\test_multiplxProcessor\out\statistics.txt STITCHING BEGINS! Completed Stitching in 0ms Writing full image to: C:\Users\IT-WL-annkl878\Documents\notes\Codex_Analysis\test_multiplxProcessor\out\stitched-0.tif  Width: 3929 Height: 3965 Saving tiles to file: C:\Users\IT-WL-annkl878\Documents\notes\Codex_Analysis\test_multiplxProcessor\out\stitched-0.tif Finished saving full image: C:\Users\IT-WL-annkl878\Documents\notes\Codex_Analysis\test_multiplxProcessor\out\stitched-0.tif Completed output options for slice 0. Done gridwidth=2 gridheight=2 starttile=1 imagedir='C:\Users\IT-WL-annkl878\Documents\notes\Codex_Analysis\test_multiplxProcessor\out' filenamepatterntype=SEQUENTIAL filenamepattern={ppppp}_cy006_ch1.tif gridorigin=UL numberingpattern=HORIZONTALCONTINUOUS assemblefrommetadata=true globalpositionsfile='C:\Users\IT-WL-annkl878\Documents\notes\Codex_Analysis\test_multiplxProcessor\out\global-positions-0.txt' assemblenooverlap=false startrow=0 startcol=0 extentwidth=2 extentheight=2 timeslices=0 istimeslicesenabled=false outputpath='C:\Users\IT-WL-annkl878\Documents\notes\Codex_Analysis\test_multiplxProcessor\out' displaystitching=false outputfullimage=true outputmeta=true outputimgpyramid=false outfileprefix= blendingmode=LINEAR blendingalpha=1 numfftpeaks=100 isusedoubleprecision=true translationrefinementmethod=EXHAUSTIVE programtype=FFTW headless=true Checking args for stitching: Arg check passed Warning: the following files will be overwritten: C:\Users\IT-WL-annkl878\Documents\notes\Codex_Analysis\test_multiplxProcessor\out\log.txt C:\Users\IT-WL-annkl878\Documents\notes\Codex_Analysis\test_multiplxProcessor\out\statistics.txt STITCHING BEGINS! Completed Stitching in 0ms Writing full image to: C:\Users\IT-WL-annkl878\Documents\notes\Codex_Analysis\test_multiplxProcessor\out\stitched-0.tif  Width: 3929 Height: 3965 Saving tiles to file: C:\Users\IT-WL-annkl878\Documents\notes\Codex_Analysis\test_multiplxProcessor\out\stitched-0.tif Finished saving full image: C:\Users\IT-WL-annkl878\Documents\notes\Codex_Analysis\test_multiplxProcessor\out\stitched-0.tif Completed output options for slice 0. Done gridwidth=2 gridheight=2 starttile=1 imagedir='C:\Users\IT-WL-annkl878\Documents\notes\Codex_Analysis\test_multiplxProcessor\out' filenamepatterntype=SEQUENTIAL filenamepattern={ppppp}_cy006_ch2.tif gridorigin=UL numberingpattern=HORIZONTALCONTINUOUS assemblefrommetadata=true globalpositionsfile='C:\Users\IT-WL-annkl878\Documents\notes\Codex_Analysis\test_multiplxProcessor\out\global-positions-0.txt' assemblenooverlap=false startrow=0 startcol=0 extentwidth=2 extentheight=2 timeslices=0 istimeslicesenabled=false outputpath='C:\Users\IT-WL-annkl878\Documents\notes\Codex_Analysis\test_multiplxProcessor\out' displaystitching=false outputfullimage=true outputmeta=true outputimgpyramid=false outfileprefix= blendingmode=LINEAR blendingalpha=1 numfftpeaks=100 isusedoubleprecision=true translationrefinementmethod=EXHAUSTIVE programtype=FFTW headless=true Checking args for stitching: Arg check passed Warning: the following files will be overwritten: C:\Users\IT-WL-annkl878\Documents\notes\Codex_Analysis\test_multiplxProcessor\out\log.txt C:\Users\IT-WL-annkl878\Documents\notes\Codex_Analysis\test_multiplxProcessor\out\statistics.txt STITCHING BEGINS! Completed Stitching in 1ms Writing full image to: C:\Users\IT-WL-annkl878\Documents\notes\Codex_Analysis\test_multiplxProcessor\out\stitched-0.tif  Width: 3929 Height: 3965 Saving tiles to file: C:\Users\IT-WL-annkl878\Documents\notes\Codex_Analysis\test_multiplxProcessor\out\stitched-0.tif Finished saving full image: C:\Users\IT-WL-annkl878\Documents\notes\Codex_Analysis\test_multiplxProcessor\out\stitched-0.tif Completed output options for slice 0. Done gridwidth=2 gridheight=2 starttile=1 imagedir='C:\Users\IT-WL-annkl878\Documents\notes\Codex_Analysis\test_multiplxProcessor\out' filenamepatterntype=SEQUENTIAL filenamepattern={ppppp}_cy006_ch3.tif gridorigin=UL numberingpattern=HORIZONTALCONTINUOUS assemblefrommetadata=true globalpositionsfile='C:\Users\IT-WL-annkl878\Documents\notes\Codex_Analysis\test_multiplxProcessor\out\global-positions-0.txt' assemblenooverlap=false startrow=0 startcol=0 extentwidth=2 extentheight=2 timeslices=0 istimeslicesenabled=false outputpath='C:\Users\IT-WL-annkl878\Documents\notes\Codex_Analysis\test_multiplxProcessor\out' displaystitching=false outputfullimage=true outputmeta=true outputimgpyramid=false outfileprefix= blendingmode=LINEAR blendingalpha=1 numfftpeaks=100 isusedoubleprecision=true translationrefinementmethod=EXHAUSTIVE programtype=FFTW headless=true Checking args for stitching: Arg check passed Warning: the following files will be overwritten: C:\Users\IT-WL-annkl878\Documents\notes\Codex_Analysis\test_multiplxProcessor\out\log.txt C:\Users\IT-WL-annkl878\Documents\notes\Codex_Analysis\test_multiplxProcessor\out\statistics.txt STITCHING BEGINS! Completed Stitching in 0ms Writing full image to: C:\Users\IT-WL-annkl878\Documents\notes\Codex_Analysis\test_multiplxProcessor\out\stitched-0.tif  Width: 3929 Height: 3965 Saving tiles to file: C:\Users\IT-WL-annkl878\Documents\notes\Codex_Analysis\test_multiplxProcessor\out\stitched-0.tif Finished saving full image: C:\Users\IT-WL-annkl878\Documents\notes\Codex_Analysis\test_multiplxProcessor\out\stitched-0.tif Completed output options for slice 0. Done gridwidth=2 gridheight=2 starttile=1 imagedir='C:\Users\IT-WL-annkl878\Documents\notes\Codex_Analysis\test_multiplxProcessor\out' filenamepatterntype=SEQUENTIAL filenamepattern={ppppp}_cy006_ch4.tif gridorigin=UL numberingpattern=HORIZONTALCONTINUOUS assemblefrommetadata=true globalpositionsfile='C:\Users\IT-WL-annkl878\Documents\notes\Codex_Analysis\test_multiplxProcessor\out\global-positions-0.txt' assemblenooverlap=false startrow=0 startcol=0 extentwidth=2 extentheight=2 timeslices=0 istimeslicesenabled=false outputpath='C:\Users\IT-WL-annkl878\Documents\notes\Codex_Analysis\test_multiplxProcessor\out' displaystitching=false outputfullimage=true outputmeta=true outputimgpyramid=false outfileprefix= blendingmode=LINEAR blendingalpha=1 numfftpeaks=100 isusedoubleprecision=true translationrefinementmethod=EXHAUSTIVE programtype=FFTW headless=true Checking args for stitching: Arg check passed Warning: the following files will be overwritten: C:\Users\IT-WL-annkl878\Documents\notes\Codex_Analysis\test_multiplxProcessor\out\log.txt C:\Users\IT-WL-annkl878\Documents\notes\Codex_Analysis\test_multiplxProcessor\out\statistics.txt STITCHING BEGINS! Completed Stitching in 1ms Writing full image to: C:\Users\IT-WL-annkl878\Documents\notes\Codex_Analysis\test_multiplxProcessor\out\stitched-0.tif  Width: 3929 Height: 3965 Saving tiles to file: C:\Users\IT-WL-annkl878\Documents\notes\Codex_Analysis\test_multiplxProcessor\out\stitched-0.tif Finished saving full image: C:\Users\IT-WL-annkl878\Documents\notes\Codex_Analysis\test_multiplxProcessor\out\stitched-0.tif Completed output options for slice 0. Done Measure - completed : 0 / 24 Measure - completed : 1 / 24 Measure - completed : 2 / 24 Measure - completed : 3 / 24 Measure - completed : 4 / 24 Measure - completed : 5 / 24 Measure - completed : 6 / 24 Measure - completed : 7 / 24 Measure - completed : 8 / 24 Measure - completed : 9 / 24 Measure - completed : 10 / 24 Measure - completed : 11 / 24 Measure - completed : 12 / 24 Measure - completed : 13 / 24 Measure - completed : 14 / 24 Measure - completed : 15 / 24 Measure - completed : 16 / 24 Measure - completed : 17 / 24 Measure - completed : 18 / 24 Measure - completed : 19 / 24 Measure - completed : 20 / 24 Measure - completed : 21 / 24 Measure - completed : 22 / 24 Measure - completed : 23 / 24 Measure - completed : 24 / 24
